# Substitution-Induced Mechanistic Switching in S_N_Ar-Warheads for Cysteine Proteases

**DOI:** 10.3390/molecules29112660

**Published:** 2024-06-04

**Authors:** Collin Zimmer, Jan Brauer, Dorota Ferenc, Jessica Meyr, Patrick Müller, Hans-Joachim Räder, Bernd Engels, Till Opatz, Tanja Schirmeister

**Affiliations:** 1 Institute of Pharmaceutical and Biomedical Sciences, University of Mainz, Staudingerweg 5, 55128 Mainz, Germany; cozimmer@uni-mainz.de (C.Z.); muelpat@uni-mainz.de (P.M.); 2 Department of Chemistry, University of Mainz, Duesbergweg 10-14, 55128 Mainz, Germany; j.brauer@uni-mainz.de (J.B.); ferenc@uni-mainz.de (D.F.); 3 Institute of Physical and Theoretical Chemistry, Julius-Maximilians-University Würzburg, Am Hubland, 97074 Würzburg, Germany; jessica.meyr@uni-wuerzburg.de (J.M.); bernd.engels@uni-wuerzburg.de (B.E.); 4 Max Planck Institute for Polymer Research, Ackermannweg 10, 55128 Mainz, Germany; raeder@mpip-mainz.mpg.de

**Keywords:** electrophilic warhead, S_N_Ar, rhodesain, covalency, reversibility, permeability

## Abstract

The aim of this study was to investigate the transition from non-covalent reversible over covalent reversible to covalent irreversible inhibition of cysteine proteases by making delicate structural changes to the warhead scaffold. To this end, dipeptidic rhodesain inhibitors with different *N*-terminal electrophilic arenes as warheads relying on the S_N_Ar mechanism were synthesized and investigated. Strong structure–activity relationships of the inhibition potency, the degree of covalency, and the reversibility of binding on the arene substitution pattern were found. The studies were complemented and substantiated by molecular docking and quantum-mechanical calculations of model systems. Furthermore, the improvement in the membrane permeability of peptide esters in comparison to their corresponding carboxylic acids was exemplified.

## 1. Introduction

Rhodesain (*Tb*CatL), a key cysteine protease of the deadly human parasite *Trypanosoma brucei*, is considered a validated drug target for the treatment of human African trypanosomiasis [1,2,3]. This infectious disease, caused by two regional subspecies of the pathogen, still poses a burden to countries, mainly in rural central Africa, where 500–1000 people are newly diagnosed per year and three million people are at risk of contracting the disease [4]. In the past decades, the WHO has taken significant efforts to tackle this problem: while chemotherapy has proven effective on the individual level, vector control targeting the transmitting tsetse fly has been effective in controlling the disease spread. This progressed its “elimination as a public health problem” with the goal of “elimination of transmission” by 2030 [5]. The ongoing research and clinical trials on this disease still result in the discovery and even approval of novel drugs, and remarkable advances have been achieved in the last five years in the context of efficacy, application, and side effects (fexinidazole, approved in 2021, and acoziborole, in phase II/III clinical trials) [6,7].

Protease inhibition in general is a relevant contributor to clinical disease management, especially for viral infectious diseases such as hepatitis C, acquired immune deficiency syndrome (AIDS), and COVID-19 [8,9]. In research, the concept is also employed for autoimmune diseases, and different types of cancer [10]. Due to the high similarity of the binding sites of cysteine proteases of the papain family (e.g., cathepsins L and S), rhodesain is an interesting model system for mechanistic investigations on other targets. It can be produced in large quantities in a straightforward fashion using standard molecular biology methods [11], and in contrast to many commercially available enzymes, it is exceptionally stable and retains catalytic activity for extended periods and under a variety of conditions. On a structural level, it possesses the precatalytically deprotonated active-site cysteine of the CA clan proteases, a highly reactive nucleophile [12].

To address this active-site nucleophile, there are numerous reports on covalent rhodesain inhibitors, but data on non-covalent ones are scarce, especially on a mechanistic level [13,14,15]. In a previous project on this topic, fluorine- and nitro-substituted arenes as warheads in combination with a dipeptidic binding motif suitable for rhodesain [16,17] were identified as a novel class of inhibitors. The S_N_Ar-reaction path—the underlying principle of the inhibition mode of these compounds—is characterized by multiple stages, starting with the formation of a non-covalent π-complex between nucleophile and electrophile, followed by the formation of a covalent Meisenheimer-type anion (σ-complex) [18]. The anion can be oxidized, yielding the so-called Zimmermann product; the substitution reaction can take place by elimination of a leaving group, or the addition can revert back to the original reactants via the π-complex. The application of electron-deficient arenes in the context of addressing (non-)catalytic cysteines irreversibly through a substitution reaction has been described on different targets, e.g., peroxisome proliferator-activated receptors (PPARs) [19], the bacterial enzyme sortase A (SrtA) [20], the fibroblast growth factor receptor 4 (FGFR4) [21], S6 kinase β2 (S6K2) [22], and DNA methyltransferase 2 (DNMT2) [23]. While the electrophiles look similar to the ones described for rhodesain, the inhibition mechanism differs substantially. The previously identified arene-based rhodesain inhibitors surprisingly act via a non-covalent mechanism [17,24]. One of these compounds, specifically with a free C-terminus, was identified as a highly affine and selective inhibitor of rhodesain [17]. Initial mechanistic experiments and theoretical investigations pointed to a nucleophilic attack onto the arene that only progresses until the π-, rather than the σ-complex [17]. MALDI-TOF mass spectrometry (MS) later provided further experimental validation that the reaction stops at the non-covalent π-complex [24]. Its effect is supported by anti-trypanosomal data from a cell-based assay and has been explained by the prodrug concept in the context of cell permeability [17]. 

As a follow-up, we now conducted a systematic structure activity relationship (SAR) study to further elucidate the criteria for affinity and to assess the prerequisite electrophilicity necessary to stop the described reaction on either intermediate on the reaction path to a complete (and irreversible) S_N_Ar reaction. The inhibition of rhodesain was assessed using classical fluorimetric substrate displacement assays. The covalency of binding was investigated using matrix-assisted laser desorption/ionization time-of-flight mass spectrometry (MALDI-TOF-MS) with an acidic matrix to suppress the formation of non-covalent complexes. The (ir)reversibility of inhibition was shown, and the experimental findings on covalency and reversibility were substantiated by quantum-mechanical (QM) calculations and docking to suggest explanations on a molecular level. For the most interesting compounds, selectivity against related human cysteine proteases was investigated. Furthermore, experimental reasoning for the observed anti-trypanosomal activity of ester derivates in a cellular context is given with results from a parallel artificial membrane permeability assay (PAMPA).

## 2. Results

### 2.1. Design and Synthesis

**Figure 1:**
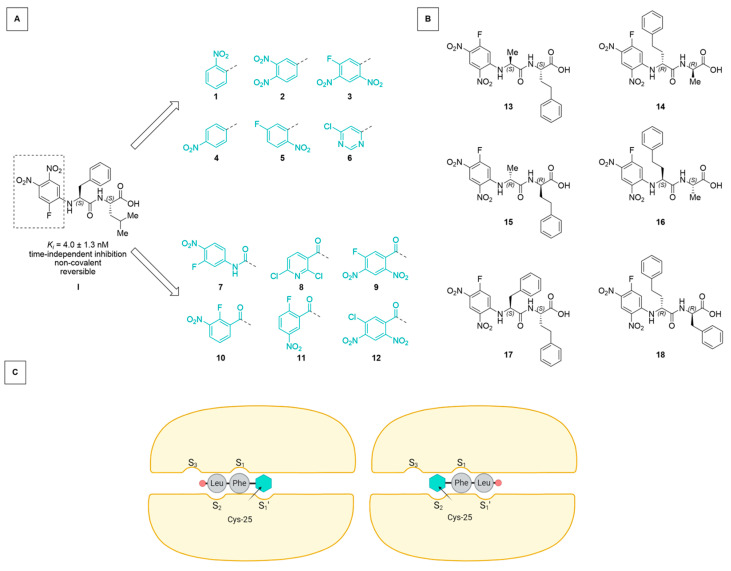
(**A**) Starting from the lead compound **I** [17], an SAR study was performed investigating the effect of the electrophilicity and leaving group properties of the *N*-terminal arene “Ar” of the Ar-Phe-Leu-OH scaffold. (**B**) Dipeptidic motifs (based on Ala-hPhe and Phe-hPhe) with inversed sequence and/or chirality (retro-inverso) were employed to assess the binding directionality. (**C**) Schematic representation of two possible orientations in which the electrophilic arene interacts with the catalytic Cys-25 (inhibitor *C*-terminus as red circle, arene as cyan hexagon). Protein subpocket nomenclature after Schechter and Berger [28] (created using BioRender.com).

To investigate binding orientation and inhibition modes, two strategies were followed:

(A) We used the general structure of Ar-Phe-Leu-OH, relating to the dipeptide phenylalanine-leucine, with “Ar” being an electron-deficient arene attached to the Phe-*N*-terminus, and “-OH” signifying the free carboxylic acid at the *C*-terminal leucine (Figure 1a). With this, we expanded the SAR study for the arene moiety of the not-yet-explored acid counterparts of the previously described esters (compounds **1–12**) [17]. Starting from an arene with only one electron-withdrawing substituent, the influence of substituent number, identity, and position, and the presence of an adequate leaving group were investigated in an effort to observe differences in interaction with the nucleophilic thiolate in rhodesain.
(B) Employing the concept of retro-inverso peptides [25,26], we combined homophenylalanine (hPhe, a strong interactor with rhodesain’s S1 subpocket) and Ala (an expected weak interactor) in an inverted sequence and chirality. With this, we wanted to further probe the directionality of ligand binding (compounds **13–16**). Additionally, we transferred the motif of *N*-terminal, electron-deficient arene and unprotected *C*-terminus onto the dipeptide of the known irreversible pan-cathepsin inhibitor K11777 [27] (Phe-hPhe) and the respective retro-inverso derivate (compounds **17–18**).

**Scheme 1:**
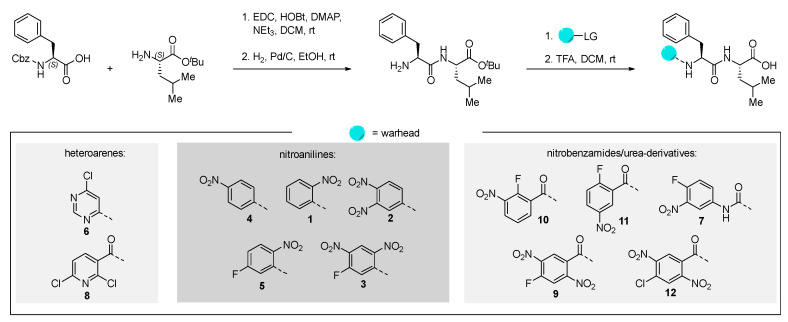
Synthesis of the tested peptides. Yields and detailed reaction conditions can be found in the SI.

All inhibitor candidates were synthesized using known strategies [17]. We started from the commercially available *N*-(carbobenzyloxy)-L-phenylalanine and L-leucine *tert*-butyl ester hydrochloride, which were linked using standard coupling procedures. After hydrogenolytic deprotection of the *N*-terminus, the warheads were attached using different reaction conditions (for further information, we refer to the Supporting Information (SI)). The free carboxylic acid was then obtained via trifluoroacetic acid-promoted ester cleavage (Scheme 1).

We tried to utilize solid-phase synthesis for the preparation of the homophenylalanine-bearing dipeptides. However, this strategy suffered from low yields for two substrates and failed to deliver the envisioned product in one case. We therefore reverted to the previous approach, which worked for all depicted molecules in good yields over four steps. The detailed procedures for the preparation of these compounds can be found in the SI.

### 2.2. Inhibition Assay

Inhibition of rhodesain was assessed using Z-Phe-Arg-AMC as a fluorogenic substrate [29]. IC_50_ or *K*_I_^app^ were determined depending on the time dependency of the progress curves. Because a competitive mode of inhibition can be assumed [17], *K*_i_ (for reversible inhibition) and *K*_I_ (for irreversible inhibition) were calculated using the Cheng–Prusoff equation [30]. Selectivity towards human cathepsins L and B (*Hs*CatL, *Hs*CatB) was assessed for the most active compounds. The results can be found in Table 1.

**Figure 2:**
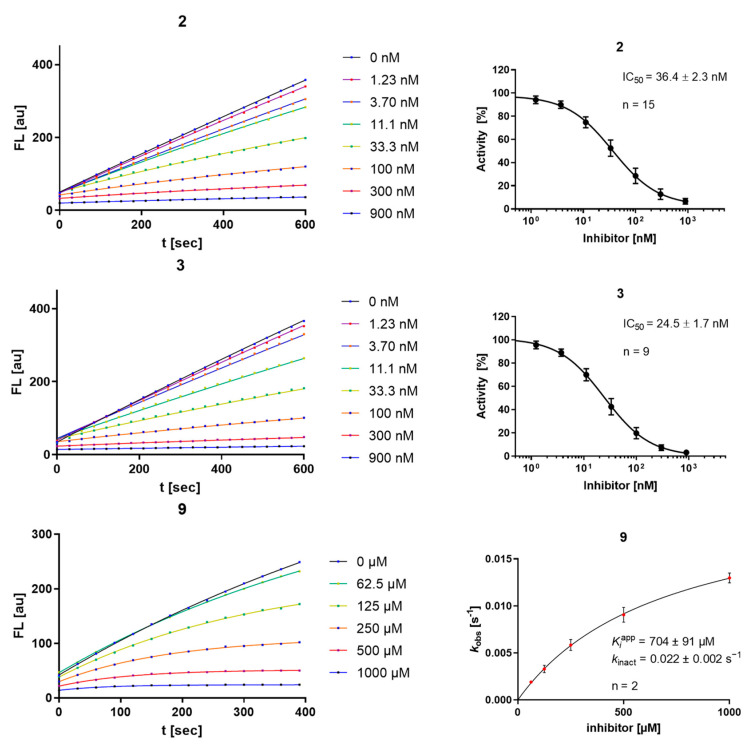
Progress curves (**left**) and IC_50_ determination for the time-independent inhibitors **2** and **3**, and *k*_obs_-[I] diagram, *K*_I_^app^, and *k*_inact_ for the irreversible inhibitor **9** (**right**); “n” is the number of replicate measurements for each depicted concentration.

An interesting observation is that the number of strongly inhibiting arene motifs is highly limited. The strongest inhibition was displayed by compounds **2** and **3**, showing *K*_i_ values in the single-digit nM range. They showed the previously described [17] high degree of selectivity towards the investigated human off-target cathepsins, which seems to be characteristic of the most active inhibitors. All but compound **9** showed linear progress curves, indicating fast-reversible inhibition (Figure 2). The irreversibility of inhibition for compound **9** was proven by dilution assays (SI-Figure S6) as described in the literature [29]. The degree of covalency and therefore a distinction between π- or σ-complex formation in the inhibited state of the complex between rhodesain and **2** or **3** cannot be made with this data alone, but is further assessed below (Section 2.3 and Section 2.5). Consequently, the results in Table 1 indicate that from an electrostatic view, aniline derivatives with two nitro groups as strongly electron-withdrawing substituents in the absence or presence of an additional fluorine substituent as a suitable leaving group are necessary for strong reversible inhibition (e.g., cpd. **2** and **3**). From the benzamide derivatives, compound **9** with fluoro-dinitro-substitution shows time-dependent progress curves in the inhibition assay and undergoes an irreversible reaction with rhodesain.

The explanation for high-affinity binding with strong inhibition and a distinction between covalent or non-covalent modes of inhibition is naturally based on multiple aspects. Reasoning can, for example, be based on electrostatic properties as prerequisites for the nucleophilic attack, but also on the presence of a suitable leaving group, favorable positioning inside the active site, and optimal bond geometries during transition states. All are important factors in deciding about stable complexes, covalent bond formation, and substitution. By discussing electrostatic and leaving group properties, the following structure–activity relationships can be inferred. Starting from aniline as the arene portion, substituents with increasing electron-withdrawing properties were employed. First, the mono-nitro-substituted products (*ortho*- (**1**) and *para*- (**4**); *meta*- was not easily accessible) were evaluated; they did not show inhibition. Second, an additional fluorine atom was installed as a −I substituent and a potential leaving group (**5**), which also did not result in inhibition. Following the analogy of replacing nitro groups with ring nitrogen [31], while simultaneously exchanging fluorine with chlorine (**6**), did not produce an active inhibitor either. Only after transitioning to the dinitro-substituted anilines with or without an additional fluorine substituent (**2** and **3**) was strong inhibition observed again with a time-independent character. The *K*_i_ values obtained were in the single-digit nM range as for the previously reported fluoro-dinitro-bearing inhibitor **I** (compare Table 1 and Figure 2). Exchanging the fluoro-dinitro-aniline with a benzamide motif (**9**), thereby exchanging the +M-amine nitrogen with a −M-carbonyl substituent, resulted in a drop in affinity, but interestingly in the only compound showing time-dependent inhibition in the series. This is due to irreversible inhibition of rhodesain (compare Figure 2 and SI-Figure S6). A similar substitution of the aniline with a benzamide motif while retaining a monofluoro-mononitro substitution (**10** and **11**) was ineffective. Likewise, limiting the electron-donating effect of the aniline nitrogen by transformation to a urea motif on a monofluoro-mononitro scaffold did not produce an active inhibitor (**7**). A chlorine-substituted hetarene carboxamide (**8**) also did not yield relevant inhibition. Moreover, the exchange of fluorine with chlorine on the fluoro-dinitrobenzamide scaffold (**12**) also abolished the time-dependent mode of inhibition, indicating that a substitution reaction with chloride as leaving group does not take place.

Validating experiments regarding the inner-filter effect and reactivity towards unspecific nucleophiles are depicted in the SI (SI-Figures S1–S5). Most compounds of the aniline series show a relevant inner-filter effect at concentrations exceeding 10 µM, which was considered in the performed calculations. In contrast to compounds **2** and **3**, compound **9** shows relevant reactivity towards unspecific nucleophiles (water and DTT) over the course of minutes to hours, which underlines its high electrophilicity.

### 2.3. Mass Spectrometric Analysis

Using MALDI-TOF-MS, differentiation between non-covalent and covalent ligand-target complexes is possible if acidic matrices are used that interfere with ionic and dipole interactions (i.e., suppress non-covalent interactions) [32]. Using this method, the non-covalent inhibition mechanism of lead compound **I** was proven, as no adduct could be detected by MALDI-TOF MS, but a respective adduct peak was detected using the native ESI-MS technique, which allows for the detection of non-covalent complexes [17,24].

**Figure 3:**
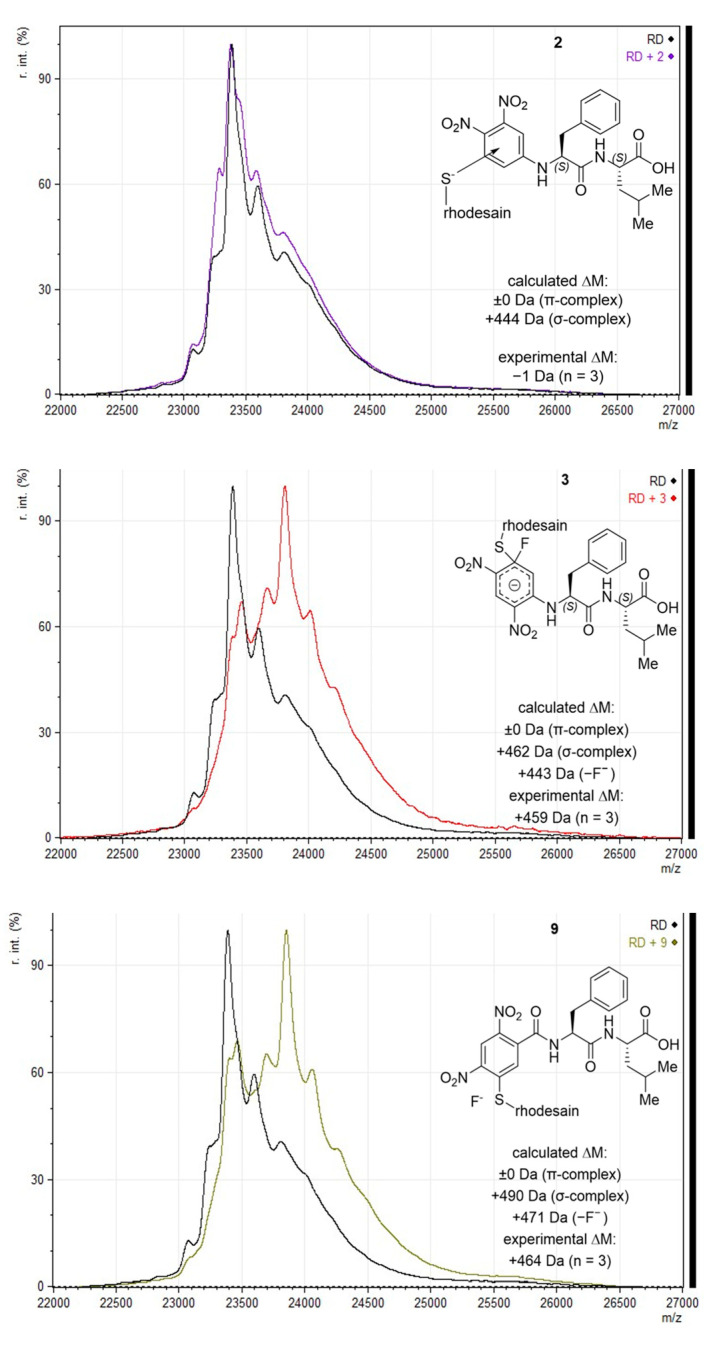
MALDI-TOF-MS (sinapinic acid matrix) with calculated and experimentally determined mass shifts, and depiction of assumed reaction products; “calculated ΔM” states mass shifts for possible S_N_Ar products. For “experimental ΔM”, mean m/z of the most dominant shifted peak for each of the compounds was subtracted from mean m/z of rhodesain [M+H]^+^; replicate measurements for each compound are indicated as “n = x”.

The MALDI method was applied to the novel compounds (Figure 3). For **2**, no adduct was found using MALDI-TOF-MS, advocating for the dominant formation of the non-covalent π-complex. For **3**, the constitutional isomer of **I**, an adduct corresponding to the mass of the inhibitor was observed, indicating the formation of a covalent σ-complex, but not the elimination of any leaving group. This difference can be explained by the relative substitution pattern with the assumption that the *ipso*-carbon of the fluorine is the most electron-deficient one (as shown by the reaction of **3** and **9** with other nucleophiles; SI-Figures S2–S4). In **I**, the electron density on the fluorine-substituted aryl-carbon is expected to be higher than in the novel compound with a changed substitution pattern. The *o*,*p*-dinitro pattern in **3** can more effectively withdraw electron density than the *m*,*p*-dinitro pattern in **I**, leading to the conclusion that the predominant inhibition product of **3** under assay conditions is the σ-complex depicted below. In addition to differences in electron distribution, unfavorable geometries can affect σ-complex formation or later prohibit the full progression of the substitution. An adduct was also found for **9**, albeit with a shift lower than the mass of the inhibitor, indicating both the formation of the covalent bond and the full substitution of fluoride, which is in line with its irreversible inhibition data (Figure 3), its reactivity towards other nucleophiles (SI-Figures S2–S4), and the quantum-mechanical calculations explained below (Section 2.5).

### 2.4. Docking

All tested compounds were docked into the active site of rhodesain (pdb: 2p7u [33]) using LeadIT. For each compound, the 10 poses ranked highest were manually inspected to identify common binding features. Besides general positioning, the minimal distance between the catalytic thiolate of rhodesain’s Cys-25 and any arene carbon (as proxy for π-complex formation) or halogen-bound carbon (expected point of attack for σ-complex formation) was calculated using Pymol.

**Figure 4:**
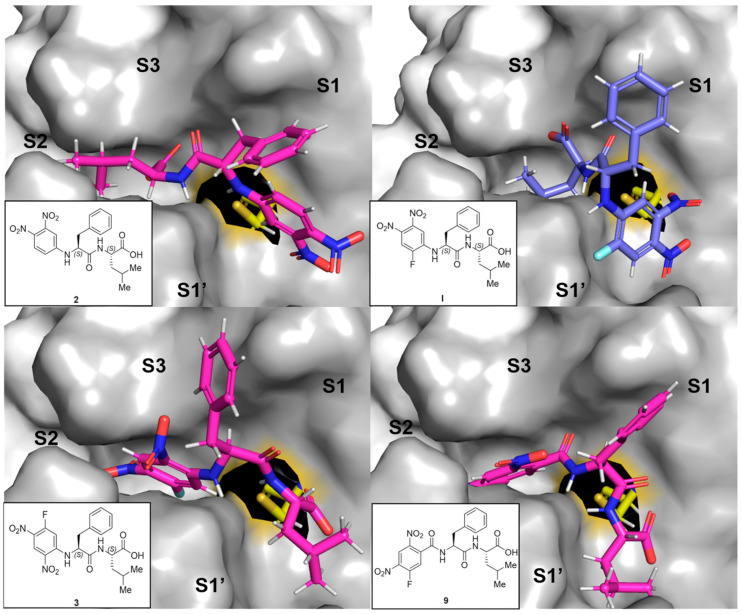
Representative predicted docking poses for compounds **2**, **3**, **9** (in pink), and **I** (in blue) in the active site of rhodesain (pdb 2p7u [27]). Catalytic Cys-25 is depicted as sticks (yellow), and the rest of the binding site as a surface with labeled subpockets (gray).

As expected, two major orientations can be described (substrate or inverse-substrate orientation), with the arene positioned in either the S2 or S1′ pocket, respectively (shown for **I**, **2**, **3**, and **9** in Figure 4). In all benzamide-type and urea-containing inhibitors, the arene was reliably placed in the S2 pocket with substrate orientation. The aniline-derived series can adopt both orientations with different preferences depending on the arene employed. For **2** with an arene warhead in S1′, the Phe side chain is positioned into the S1 pocket, while Leu is placed into the S2 pocket. The smallest predicted distance between Cys-25 and an arene-carbon is 3.0 Å. The same general orientation is adopted by **I**, with the smallest distance of 3.5 Å, and the distance between Cys-25 and the fluorine-bound carbon is 4.8 Å (oriented away). For **3**, the arene is in the S2 pocket with a more substrate-like binding orientation of side chains with a frontal C-terminus. The smallest Cys-25/arene distance here is 3.2 Å and the distance to the fluorine-bound carbon is 4.2 Å, oriented more towards the cysteine compared to **I**. The predicted unfavorable arene position in terms of C-F to Cys-25 distance in **I** compared to **3** can be another argument for the observed difference in the covalency of the inhibition complex (π- vs. σ-complex). Compound **9** is also positioned in the substrate-like orientation with arene in S2, but with larger distances to Cys-25 (3.9 Å as minimal distance to the arene and 4.9 Å to the fluorine-bound carbon), which might be a reason for the observed drop in affinity. The same argument holds true for chlorine analog **12** (SI-Figure S7B).

Inferring from the docking poses, high degrees of inhibition can be achieved by engaging the active site in both orientations, but it seems like covalently addressing the catalytic cysteine is more easily achieved from the S2 pocket in the substrate orientation than from the S1′ site in inverse orientation (with a sufficiently electrophilic arene). This hypothesis requires verification by crystallographic experiments and/or simulations that take the enzyme flexibility into account, but it indicates a second layer of reason in addition to electron deficiency of the arene as to why some compounds react covalently, while others bind non-covalently. Interestingly, the compounds with *N*-terminal (*R*)-hPhe-based peptidic sequences might be unfavorable in general, as they can force the neighboring arene away from Cys-25 (as depicted for **18** in SI-Figure S7A), indicating why they were inactive in the assays.

### 2.5. Quantum-Chemical Calculations

**Figure 5:**
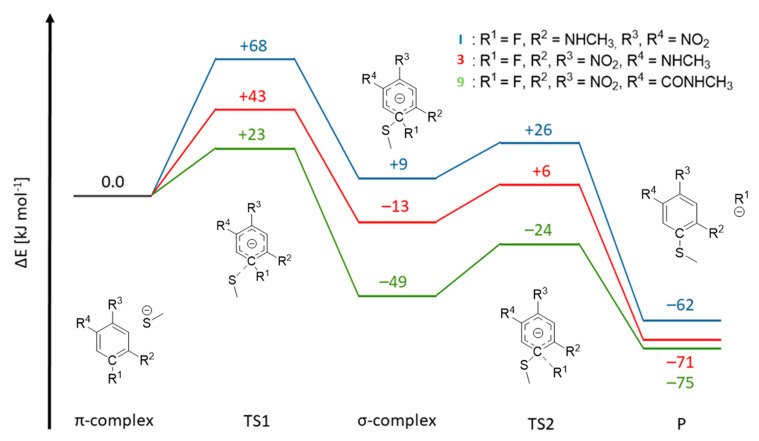
Reaction paths for model compounds **I**, **3**, and **9**. The π-complex of the reactants, the transition state for the formation of the Meisenheimer complex (TS1), the Meisenheimer/σ-complex and the transition state for the elimination of the fluoride (TS2) were calculated, yielding the substitution product (P). The respective C-R1 and S-CR1 distances are given in SI-Table S2.

To explain the inhibition mechanisms observed using mass spectrometric analysis, we performed quantum-chemical calculations on compounds **I**, **2**, **3**, and **9** reacting with a methyl thiolate nucleophile. As shown in Figure 5, we first calculated the π-complexes of the compounds, which were all very similar in energy (compared to the separated reactants). We chose to compare the electronic energies obtained in the calculation because in an enzymatic environment a non-covalent complex is formed prior to the reaction and the bond formation occurs without the entropic penalty. The corresponding free energy reaction paths are shown in SI-Figure S8. Compounds **I**, **3**, and **9** are able to react in the defined S_N_Ar reaction, while compound **2**, which does not contain a fluorine substituent, remains in the non-covalent π-complex. The energy barrier associated with the nucleophilic attack (TS1), leading to the formation of the Meisenheimer/σ-complex, is highest for compound **I**, followed by compound **3**. Additionally, the σ-complex of **I** is less stable than the π-complex (ΔE = +9 kJ∙mol^−1^), causing this inhibitor to remain in a non-covalent complex with rhodesain. The arene with the highest number of electron-withdrawing groups, compound **9**, yields the lowest energy barrier for the formation of the σ-complex (ΔE = +23 kJ mol^−1^). The resulting Meisenheimer complex is exothermic, with ΔE = −49 kJ mol^−1^, and by crossing a small barrier (TS2, ΔE = −24 kJ mol^−1^), the strongly exergonic substitution product is obtained. Thus, our calculations closely match the experimental observations of an irreversible reaction of **9** with rhodesain. For compound **3**, the σ-complex is calculated to be slightly exothermic, with ΔE = −13 kJ mol^−1^, with a barrier of ΔE = +6 kJ mol^−1^ for TS2. Our QM model calculations suggest that both compounds **3** and **9** would react irreversibly. However, the enzymatic environment can strongly influence warhead reactivity patterns, as seen for (fluoro)vinylsulfones [24]. Consequently, for **3**, the substitution might not be able to proceed completely due to enzymatic interactions, as discussed in the mass spectrometry section. In conclusion, although our QM calculations are not able to depict the influence of the enzymatic environment, they can provide insights into the intricate reactivity patterns of electron-deficient arenes with a thiolate.

### 2.6. Parallel Artificial Membrane Permeability Assay (PAMPA)

For the presented class of inhibitors, cell data are available for carboxylic acid **I** and its ester [17]. While being highly active against rhodesain, the free acid did not show strong effects in cell-based assays, in contrast to its ester, which did not inhibit isolated rhodesain but exhibited notable anti-trypanosomal activity [17]. Due to its higher lipophilicity, the ester was reasoned to be cell-permeable, releasing free acid as a highly potent rhodesain inhibitor after hydrolysis. The carboxylic acid is then trapped inside the cell due to its charge (cpK_A_ is 4.8 for **2** and 5.2 for **3** [33]). By the same argument, the free acid cannot enter the cells efficiently under cell culture conditions (pH = 7.4). To experimentally substantiate this hypothesis, we compared the passive permeability of the compounds as carboxylic acids (**2** and **3**) and as *tert*-butyl esters (**20** and **21**) using a standard PAMPA setup with propranolol and methotrexate as suitably well- and poorly permeable control substances, respectively, and candesartan and candesartan cilexetil as a comparative model for an acid–ester drug/prodrug pair (Table 2).

As expected, all esters (candesartan cilexetil, **20** and **21**) clearly show increased permeability over their acid counterparts. Generally, limited permeability is not uncommon for peptidic compounds, even with blocked termini [38,39]. In this context, the strong increase in permeability from **2** to **20** is remarkable. These findings experimentally clarify the previous observation in cell cultures for the acid–ester pair of **I** (EC_50_ of 20 µM vs. 100 nM) [17].

## 3. Discussion

To further investigate the mode of inhibition for dipeptidic rhodesain inhibitors carrying an *N*-terminal electrophilic arene as a warhead and an unprotected *C*-terminus, we investigated 18 different compounds following two strategies. By modulating the arene warhead (compounds **1–12**), we identified two highly affine and selective reversible inhibitors with *K*_i_ values of 2.8 and 1.9 nM (**2** and **3**) and an irreversible inhibitor with a *k*2nd value of 4.1 × 10^2^ M^−1^s^−1^ (**9**). Focusing on a distinction between the S_N_Ar intermediates of non-covalent π-complex (**2**), covalent reversible σ-complex (**3**), and irreversible substitution (**9**), we identified a representative substitution pattern for the warhead that mediates each of these steps as the predominant binding state, which was experimentally proven by MALDI-TOF mass spectrometry, clarifying binding stability in the context of covalent bond formation. This was underlined by quantum-mechanical calculations, indicating that these are indeed the energetically most favorable states in each case. While they all form an energetically similar π-complex, only **3** and **9** showed an exothermic σ-complex for the attack at the fluorine-bound carbon. The retro-inverso approach (compounds **13–18**) underlined that the peptidic sequence and exact positioning of the arene is also a sensitive deciding factor for affinity. Molecular docking simulations elucidated favorable orientations that also suggested two different engagement possibilities between arene and Cys-25 through either the S2 or S1′ pocket, adding indications for covalent engagement of the catalytic cysteine. Finally, PAMPA was used to prove that the *C*-terminal esters have markedly increased membrane permeability than their acid homologs, providing explanation for the difference in anti-trypanosomal effect described previously.

## 4. Materials and Methods

See also Supplementary Materials.

### 4.1. Enzyme Sources

Rhodesain was heterologously expressed from *P. pastoris* as described in the literature [11,29], which is described in detail in the SI. Human cathepsins L and B were commercially available from EMD Millipore (Burlington, MA, USA).

### 4.2. Enzyme Assays

Fluorimetric enzyme assays were performed as described in the literature [29]. Information regarding buffers, concentrations, the employed substrate, as well as data analysis for linear and non-linear regression is given in the SI. In short, the cleavage of a fluorogenic substrate by the protease of interest is detected in the presence of different concentrations of inhibitors. After regression, apparent values (IC_50_ and *K*_I_^app^) were obtained, which were then mathematically corrected to comparable affinity values (*K*_i_ and *K*_I_). For the irreversible compound with non-linear regression, the kinetic *k*_inact_ value was also plotted, from which *k*2nd was calculated.

### 4.3. MALDI-TOF Mass Spectrometry

Protein mass spectrometry was performed as described before [24], but also described in detail in the SI. In short, rhodesain was incubated with a surplus of inhibitor under conditions similar to those of the enzyme assays described above to enable complex formation. This sample was desalted and then incorporated into a sinapinic acid matrix, which was then subjected to mass spectrometry. Data analysis was performed using mMass [40].

### 4.4. PAMPA

The principle is described in the literature [41], and the details are described in the SI. An artificial membrane consisting of phosphatidylcholine dissolved in *n*-dodecane was employed to control equilibration between two connected aqueous compartments, of which one contained the compound of interest. This model for compound permeation through a cell membrane was incubated, and the resulting concentration in the acceptor compartment was quantified spectroscopically.

### 4.5. Docking

The non-covalent docking was performed according to the literature [17] using FlexX, with details described in the SI.

### 4.6. QM Calculations

All calculations were performed using the ORCA 5.0.4 program package [42]. Geometry optimizations were performed using ωB97X-D3 [43,44] \ma-def2-SVP [45,46] with AutoAux auxiliary basis sets [47]. All stationary points were confirmed by frequency analysis, and implicit solvation in water was included using the CPCM solvation method [48]. Free energies included a concentration correction resulting from the change in standard states going from gas phase to condensed phase [49,50]. The structures depicted in SI-Figure S9 were utilized as model compounds, with methyl thiolate as the model nucleophile.

### 4.7. Synthetic Procedures

The general procedure for the synthesis of these compounds is shown here. For the detailed procedures as well as the analytical data, we refer to the Supporting Information. Unless stated otherwise, all solvents and reagents were obtained from commercial suppliers and used without prior purification. 

### 4.8. General Procedure for Amide Coupling (GP1) 

To a round-bottom flask, benzyloxycarbonyl (Cbz)-protected amino acid (1.01 Eq.), hydroxybenzotriazole monohydrate (HOBt·H_2_O 1.01 Eq.), 1-ethyl-3-(3-dimethylaminopropyl)carbodiimide hydrochloride (EDC·HCl, 1.01 Eq.), 4-dimethylaminopyridine (DMAP, 1.01 Eq.), and the hydrochloride of the corresponding tert-butyl-protected amino acid (1.0 Eq.) were added and dissolved in dry dichloromethane (DCM, ca. 0.1 m). Triethylamine (2.0 Eq.) was added, and the mixture was stirred for 12 h. After the addition of water (20 mL), the phases were separated, and the organic phase was washed with saturated ammonium chloride solution. Afterwards, the organic phase was dried over sodium sulfate (Na_2_SO_4_), filtered, and the solvent removed under reduced pressure. The product was used without further purification. 

### 4.9. General Procedure (GP2) for the Deprotection of Cbz-Protected Amines 

In a round-bottom flask, the Cbz-protected amine was dissolved in ethanol (EtOH) or tetrahydrofurane (THF), and 10 wt% of Pd/C (5 wt%) was added. The flask was evacuated and flushed with hydrogen three times and then stirred under a slight overpressure of hydrogen until TLC showed full conversion of the starting material (roughly one hour in ethanol and 24 h in THF). The reaction mixture was filtered through celite, and the solvent evaporated. 

### 4.10. General Procedure (GP3) for the S_N_Ar 

To a 0.1 m solution of the amine in ethanol, the corresponding aromatic compound (1.0 Eq.) was added. *N*,*N*-Diisopropylethylamine (DIPEA, 2.0 Eq.) was added, and the reaction mixture was stirred until TLC showed full conversion. Twice as much water as ethanol was added, and the resulting mixture was extracted three times with ethylacetate (EtOAc, roughly the same volume as the ethanol used). The combined organic extracts were dried over Na_2_SO_4_, and the solvent was removed under reduced pressure. The crude product was purified using flash column chromatography. 

### 4.11. General Procedure (GP4) for the Deprotection of Tert-Butyl-Protected Acids 

To an ice-cold solution of the corresponding ester in DCM (1 m), trifluoroacetic acid (TFA, 50 Eq.) was added. The reaction mixture was stirred for 3 h at rt, and the solvent was removed under reduced pressure. Traces of TFA were removed via co-evaporation with toluene. The crude product was purified using either column chromatography or preparative HPLC. 

### 4.12. General Procedure (GP5) for Solid-Phase Synthesis 

#### 4.12.1. Loading and Capping 

In total, 3.7 g chlorotriethylchloride resin was washed with DCM for 30 min and the DCM was filtered. The fluorenylmethoxycarbonyl (Fmoc)-protected amino acid (0.81 mmol) was dissolved in 100 mL of a 4% collidine/DCM mixture and added to the resin. The mixture was shaken for 12 h, the solvent filtered, and the resin washed three times with 25 mL DCM. 

Then, 20 mL of a capping solution (DCM/MeOH/DIPEA, 17:2:1) was added and stirred for 1 h at rt. The solvent was filtered, and the resin was washed four times with 20 mL DCM and dimethylformamide (DMF). 

#### 4.12.2. Determination of the Loading 

Here, 1.5 mg of the resin was shaken in 1 mL of a 20% piperidine/DMF mixture for 30 min. The solution was filtered and diluted with 5 mL of methanol (MeOH). The mixture was then transferred to a cuvette. The absorption spectrum was measured, and the loading B was calculated using the following equation:

(1) $$B=\frac{A_{289nm}\times V}{\varepsilon_{289nm}\times d\times m}$$

#### 4.12.3. Coupling on the Solid Phase 

The resin was washed three times with 20 mL DMF and then shaken for 1 h with 20 mL of a 20% piperidine/DMF solution. The solvent was filtered and a solution consisting of the corresponding amino acid (5.0 Eq.), HATU (4.5 Eq.), and HOAt (4.5 Eq.) in 20 mL of a 20% piperidine/DMF was added, and the mixture was shaken for 12 h. 

#### 4.12.4. Capping and Fmoc Deprotection 

The resin was filtered from the solvent and washed three times with the same volume of DMF and then three times with 25 mL DCM. Then, 20 mL of the capping solution was added and shaken for 1 h at rt. The solution was filtered, and the resin was washed four times with 20 mL DCM and DMF. In total, 20 mL of a 20% piperidine/DMF mixture was added and shaken for 1 h. The resin was again washed five times with 20 mL DMF and three times with DCM. 

#### 4.12.5. Aromatic Substitution and Cleavage from the Resin

1,3-Difluoro-4,6-dinitrobenzene (363 mg, 1.78 mmol, 2.2 Eq.) in 20 mL EtOH was added to the resin. DIPEA (0.28 mL, 1.62 mmol, 2.0 Eq.) was added and shaken overnight at rt. The solution was filtered, and the resin washed three times with 20 mL DCM. The resin was dried for 1 h and shaken with a mixture of 18 mL TFA, 1 mL H_2_O, and 1 mL triisopropylsilane for 3 h. The mixture was filtered and removed under reduced pressure. The crude product was purified using preparative HPLC.

## Figures and Tables

**Table 1:** Overview of inhibition data (rhodesain) for synthesized compounds. Results are averages including standard deviation of at least two replicates for all compounds.

Compound	*K*_i_ or *K*_I_ [nM]	Compound	*K*_i_ or *K*_I_ [nM]
**1**	(39 ± 4) × 10^3^	**10**	(14 ± 2) × 10^3^
**2** ^#^	2.8 ± 0.2	**11**	(13 ± 2) × 10^3^
**3** ^#^	1.9 ± 0.1	**12**	(25 ± 4) × 10^3^
**4**	(17 ± 2) × 10^3^	**13**	(13 ± 1) × 10^3^
**5**	(44 ± 5) × 10^3^	**14**	(27 ± 2) × 10^3^
**6**	(102 ± 9) × 10^3^	**15**	(22 ± 4) × 10^3^
**7**	(50 ± 4) × 10^3^	**16**	(13 ± 2) × 10^3^
**8**	(30 ± 3) × 10^3^	**17**	(6 ± 0) × 10^3^
**9** *	(53 ± 7) × 10^3^	**18**	(12 ± 1) × 10^3^
**I** ^#^	4.0 ± 1.3 [17]		

# = Selectivity indices calculated as [*K*_i_ (off-target)/*K*_i_ (main target)] towards both *Hs*CatL and *Hs*CatB were >1800 and >2600 for **2** and **3**, respectively, and >375 for **I** (published data [17]); * = irreversible inhibition, *k*_inact_ = 0.022 ± 0.002 s^−1^, and *k*2nd = (4.1 ± 0.6) × 10^2^ M^−1^s^−1^.

**Table 2:** Determined and literature permeabilities for the compounds under investigation. “P_app_ = apparent permeability”. Error is only given for compounds that displayed detectable permeation (n for all compounds was ≥2). “-” = No literature value available.

Compound		P_app_ [×10^−6^ cm/s]	Lit. P_app_ [×10^−6^ cm/s]
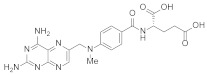	Methotrexate	0.0	0.0 [34]
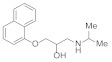	Propranolol	9.9 ± 1.0	8–14 [35,36]
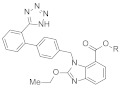	Candesartan, R = H	0.0	0.0 [37]
	Candesartan cilexetil, R = 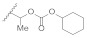	1.3 ± 0.2	0.6 [37]
** 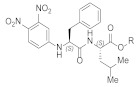 **	**2**, R = H	0.0	-
	**20**, R = *t*Bu	9.3 ± 0.7	-
** 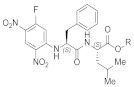 **	**3**, R = H	0.0	-
	**21**, R = *t*Bu	3.4 ± 1.8	-

## Data Availability

Data are contained within the article and Supplementary Materials.

## Supplementary Materials

The following supporting information can be downloaded at https://www.mdpi.com/article/10.3390/molecules29112660/s1: Inner-filter-effect analysis and spectroscopic characteristics (SI-Figure S1); Stability measurements towards general substitution reactions (SI-Figures S2–S5); Reversibility assessment (SI-Figure S6); Discussion on inactive compounds from strategies A and B with a focus on positional reasoning (SI-Figure S7); Quantum-mechanical free-energy calculations (SI-Figure S8); Methods for assays and QM calculations (SI-Table S1, SI-Figure S9, SI-Table S2); Synthetic procedures and compound characterization (NMR Spectra 1–115), Cartesian coordinates of QM calculation structures. References [40,41,42,43,44,45,46,47,48,49,50,51,52,53,54,55,56,57] are also cited in Supplementary Materials.
