# Supplementary material for: Substitution-Induced Mechanistic Switching in SNAr-Warheads for Cysteine Proteases
Source: Molecules. 2024 Jun 4;29(11):2660. doi: 10.3390/molecules29112660 (PMC11173422; doi:10.3390/molecules29112660)
Supplement: Supplementary file 1 [file molecules-29-02660-s001.zip › Supporting Information_molecules-3005998.pdf]

Supporting Information

Substitution-induced Mechanistic Switching in S_NAr-Warheads for Cysteine Proteases

Collin Zimmer[†,1], Jan Brauer[†,2], Dorota Ferenc[2], Jessica Meyr[3], Patrick Müller[1], Hans-Joachim Räder[4], Bernd Engels[3], Till Opatz[*,2] and Tanja Schirmeister[*,1]

[†] Both authors contributed equally

[1] Institute of Pharmaceutical and Biomedical Sciences, University of Mainz, Staudingerweg 5, 55128, Mainz, Germany

[2] Department of Chemistry, University of Mainz, Duesbergweg 10–14, 55128 Mainz, Germany

[3] Institute of Physical and Theoretical Chemistry, Julius-Maximilians-University, Am Hubland, 97074 Würzburg, Germany

[4]Max Plack Institute for Polymer Research, Ackermannweg 10, 55128 Mainz

*Corresponding authors

TABLE OF CONTENTS

INNER-FILTER EFFECT AND SPECTROSCOPIC CHARACTERISTICS 3

STABILITY MEASUREMENTS TOWARDS GENERAL SUBSTITUTION REACTIONS 4

REVERSIBILITY ASSESSMENT FOR TIME-DEPENDENT INHIBITOR 9 8

DISCUSSION ON INACTIVE COMPOUNDS FROM STRATEGIES A AND B WITH A FOCUS ON POSITIONAL REASONING 10

QUANTUM-MECHANICAL FREE ENERGY CALCULATIONS 11

METHODS 12

RHODESAIN EXPRESSION 12

RHODESAIN, CATL, CATB ASSAYS 13

MALDI-TOF EXPERIMENT 15

PAMPA 15

DOCKING 17

QM CALCULATIONS 17

SYNTHETIC PROCEDURE AND COMPOUND CHARACTERIZATION 19

REFERENCES 68

SPECTRA 70

CARTESIAN COORDINATES OF QM CALCULATION STRUCTURES SEPARATE FILE

Inner-filter effect and spectroscopic characteristics

Since especially the aniline-derivatives were found to have relatively low-energy absorption maxima, e.g., due to a *p*-nitro-aniline push-pull system (compare **SI-Figure S1A**), the inner filter effect must be ruled out as an influencing factor for the inhibition data.

To this end, fluorescence of free AMC (λ_{ex} = 380 nm, λ_{em} = 460 nm; 10 µM in assay buffer) was measured in absence and presence of inhibitor and the resulting reduction in fluorescence was analyzed for the potential to influence kinetic measurements of inhibition. The effect is independent on the concentration of AMC, but dependent on the concentration of the inhibitor, therefore different concentrations of the latter were assessed (compare **SI-Figure S1B**) [1].

It is evident that while the nitro-anilines have the potential for assay interference, this problem does only occur at high concentrations (≥10 µM), which was taken into account for affinity calculations (see the method for the cathepsin assays). For compounds **2** and **3**, the inhibition results were not mathematically corrected since the correction of assay output ([FU/s]) for each inhibitor concentration <10 µM would only have been <5 %. By analysis of the UV-spectra of **2** and **3**, it is evident that their influence at higher concentrations is mainly by primary inner-filter effect (absorption of exciting light), and only to a lesser extent by secondary inner-filter effect (absorption of emitted light) [2]. Even though **9** causes fluorescence interference at highest concentrations (8% at 500 µM and 23% at 1000 µM), this does not need to be corrected for in the utilized time-dependent mode of evaluation (therefore not depicted).

A

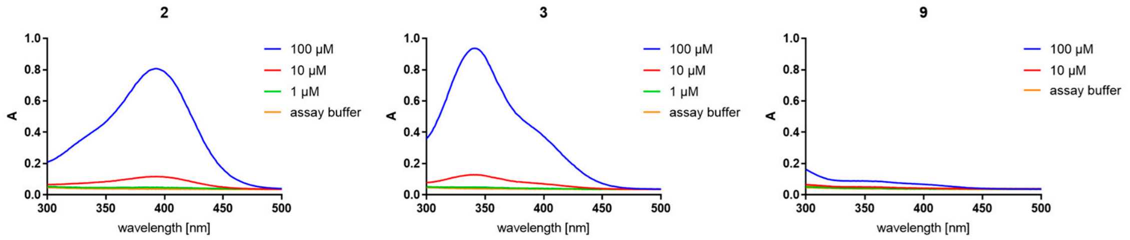

B

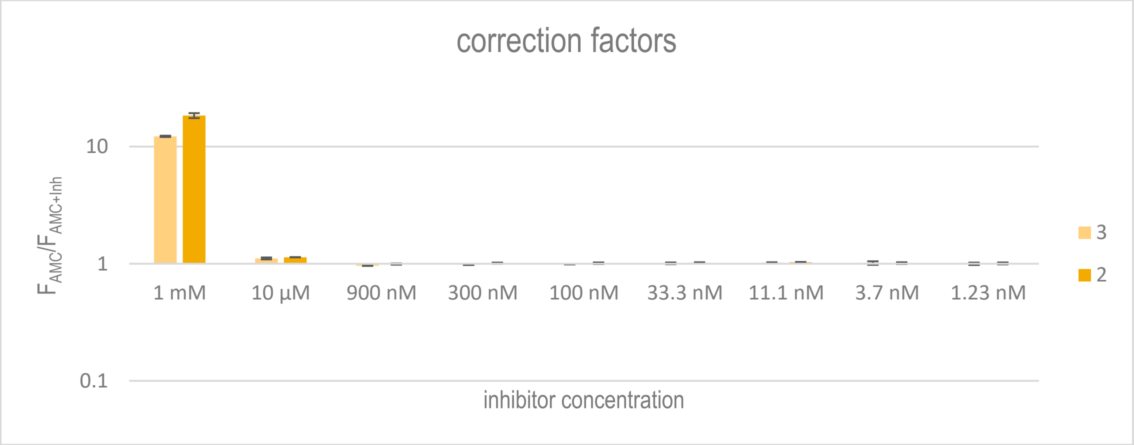

SI-Figure S1: (**A**) Absorption spectra of **2**, **3** and **9** with different concentrations, visualizing a potential need for mathematical correction at high concentrations for nitroanilines. (**B**) Experimental proof that mathematical correction of attenuation of AMC fluorescence is only necessary for inhibitor concentrations ≥10 µM. For **2** and **3** at 10 µM attenuation of fluorescence signal is 14 and 11 %, respectively. At concentrations relevant for IC_{50} evaluation, no relevant interference is detected.

Stability measurements towards general substitution reactions

2, **3** and **9** (from 20 mM stock solutions in DMSO) were diluted into NH_4OAc buffer (pH 5.5) to final concentrations of 100 µM, 125 µM or 1 mM and incubated in buffer alone or in presence of as much DTT as present in the fluorimetric rhodesain assay and the MALDI-TOF experiment, respectively. This was recorded with absorption spectroscopy (results depicted in **SI-Figure S2**) on a Tecan Spark 10M plate reader in UV-transparent 96-well plates (Greiner UV-Star®, 655801, 200 µL volume per sample) or with LCMS (see “measurement setup for LCMS” as described under the method for PAMPA; results depicted in **SI-Figure S3**).

The aniline derivates **2** and **3** show no relevant tendency towards either side reaction in an aqueous system at pH 5.5 over the course of the time necessary for the employed assays. There is a small difference detectable in reactivity of **3** with 5 mM DTT: a slight but time-dependent change in UV-spectrum was found where the maximum at 265 nm decreased in intensity, indicating a small degree of degradation

that is not observed with lower DTT concentration or with **2** at all. The formation takes place on a much larger timescale as the time for detection in the assays (minutes vs. hours) and is therefore irrelevant to the performed evaluations.

However, **9** shows a clear time-dependent change in its UV-spectrum and analysis of the reaction by repeated LCMS analysis indicates the formation of the phenol (substitution of fluoride) over the course of hours. This also seems to happen in the DMSO stock solution to a small degree after some usage (explains the presence of phenol at 0 min). Accordingly, in presence of DTT, both the phenol and the thioether are detected by MS for this compound.

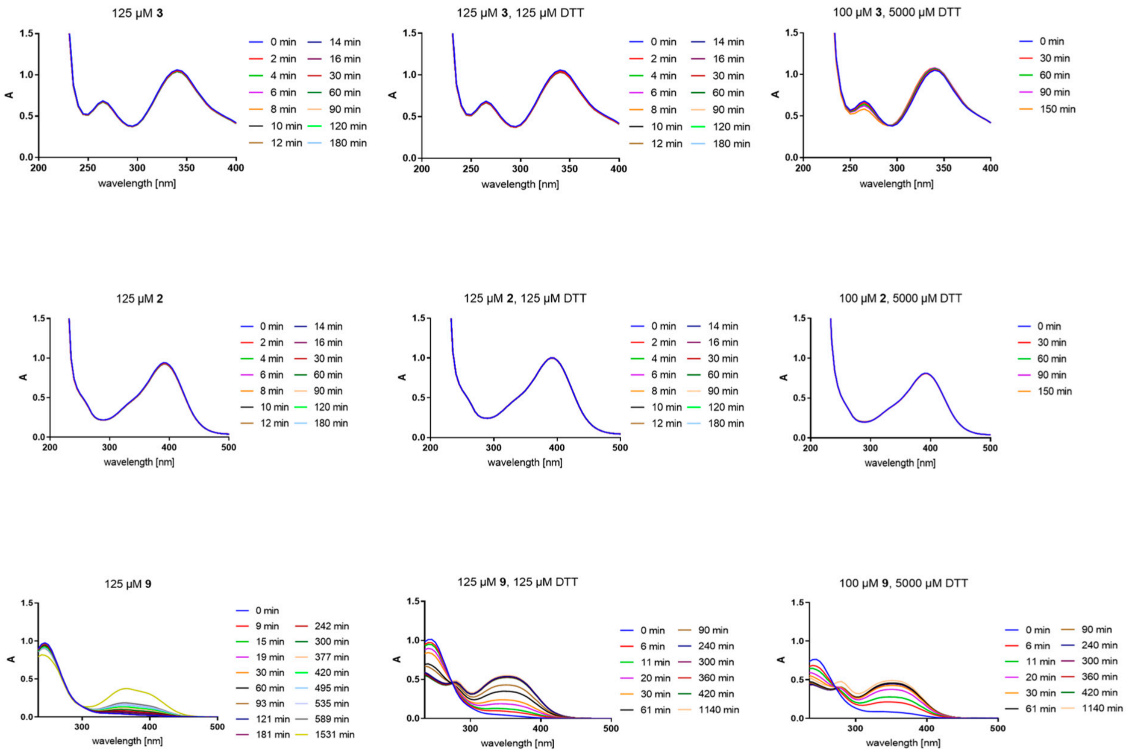

SI-Figure S2: Time-dependent absorption spectra for **2**, **3** and **9** indicating the instability of **9** towards both water and DTT even at slightly acidic pH = 5.5. Spectra for **9** were buffer-corrected prior to display.

A

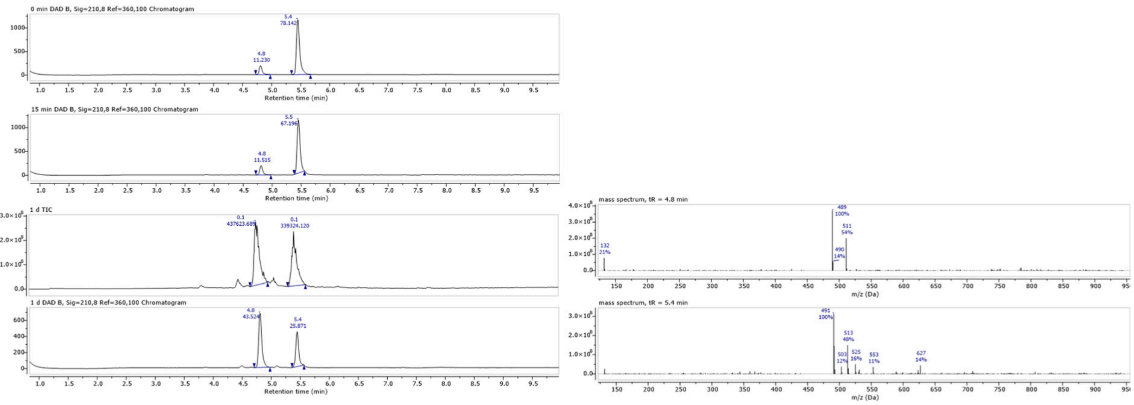

B

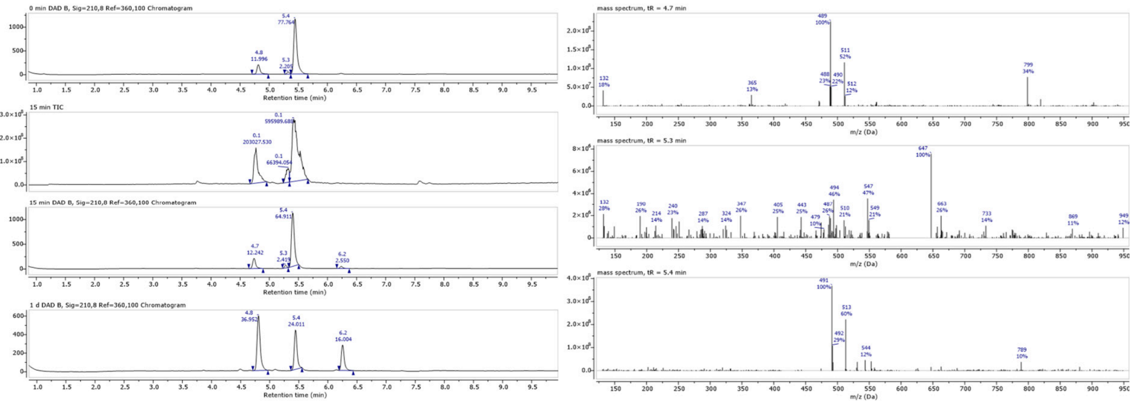

C

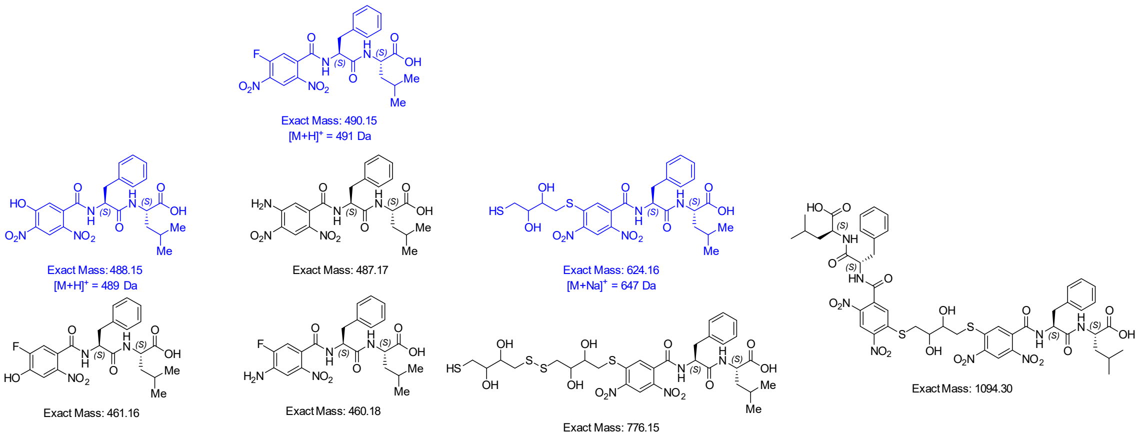

SI-Figure S3: Time-dependent degradation of 1 mM **9** to the putative phenol ($\Delta m/z = -2$ Da was interpreted as a F⟶OH exchange) in presence of ammonium acetate buffer pH 5.5 (**A**), and to the putative phenol and a putative DTT adduct in the mentioned buffer + 125 µM DTT (**B**). Some possible reaction products are shown (**C**) with the suggested structures of detected m/z (in either A or B) in blue. Alignment of TIC and DAD chromatograms was performed manually due to a known instrumental error, retention time for column dead volume with DMSO peak was excluded for clarity.

For **3** a substitution experiment in an organic solvent with an *in situ* generated thiolate was also performed: The peptide (46 mg, 0.1 mmol) was dissolved in dry methanol and a mixture of triethylamine (38 µL, 0.3 mmol) and 2-phenylethanethiol (27 µL, 0.2 mmol) was added. The reaction mixture was stirred for one minute and LCMS analysis of the sample was performed. The reaction control only showed one product, resulting from the substitution of fluoride from the aromatic system. No further reaction was observed, even after stirring for prolonged time (up to 3 h).

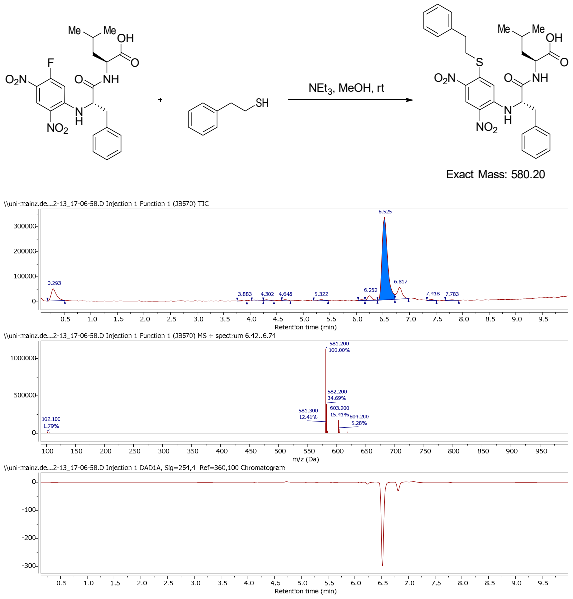

SI-Figure S4: LCMS analysis of the reaction of **3** with low-MW thiol in the presence of a base indicated almost immediate quantitative conversion to the substitution product.

It is worth noting that in our analysis of the novel compounds, for both the aniline and the benzamide, the substitution of fluoride, which is expected to be the best leaving group of the system, seems to be largely preferred over the substitution of the nitro group reported under forced conditions on **I** in the literature [3]. In this regard, the compounds are therefore expected to react preferentially with the catalytic cysteine in rhodesain under covalent binding (if it occurs) to the fluorine-bearing carbon.

The benzamide is not stable in water in both the presence and absence of DTT. In the presence of DTT, this might already be relevant on a minute-timescale. To investigate this influence, its inhibition of rhodesain was reassessed in DTT-free conditions (tris(2-carboxyethyl)phosphine = TCEP used as a non-nucleophilic reducing agent for enzyme activation). Only slightly improved affinity (K_I^{app} = 0.2 mM instead of 0.7 mM) was recorded as depicted in **SI-Figure S5**. The reactivity towards water should not be seriously relevant on that timescale, even though it is expected to complicate all analysis towards this compound. Reduced affinity due to the elongation of the scaffold by the carbonyl group and the concomitant changes in conformation and binding interactions is expected to be the main reason for the high observed K_I^{app}.

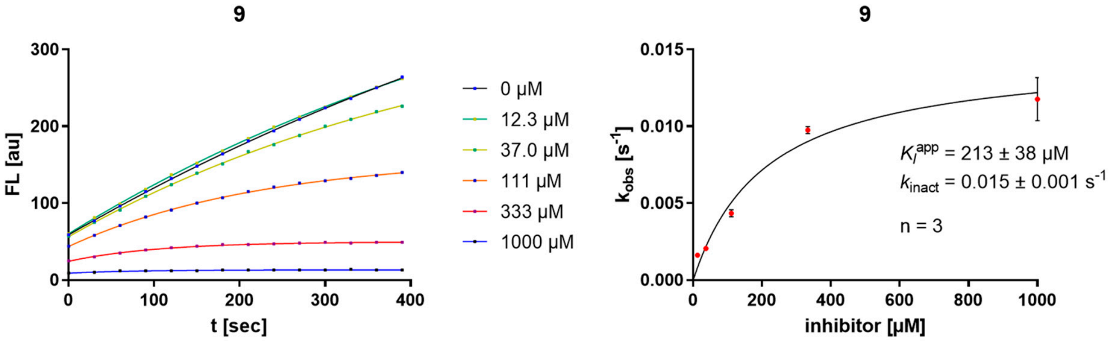

SI-Figure S5: Example progress curves, k_{obs}-[I]-diagram, K_I^{app}, and k_{inact} for the time-dependent inhibitor **9** using TCEP for rhodesain activation to enable inhibitor assessment in absence of low-MW thiol.

Reversibility assessment for time-dependent inhibitor 9

To assess reversibility of the time-dependent inhibitor [4], and to therefore discriminate between slow-reversible and irreversible inhibition, rhodesain was incubated with compound **9** for 1.5 h, at an inhibitor concentration of 400 μM (7.5x K_I) to enable the quantitative progression of a covalent irreversible reaction if one is possible. The same incubation was performed with DMSO alone as a negative control and in presence of 500 nM K11777 as a positive irreversible control. After this incubation period, the samples were diluted 1:100 (to 4 μM of compound **9** (0.075x K_I)) into assay buffer containing substrate to record the degree of recovery of enzymatic activity over the course of 10 min. It is evident that compound **9** could not be displaced by the substrate after incubation and behaved like the irreversible control K11777. The results are depicted in **SI-Figure S6**.

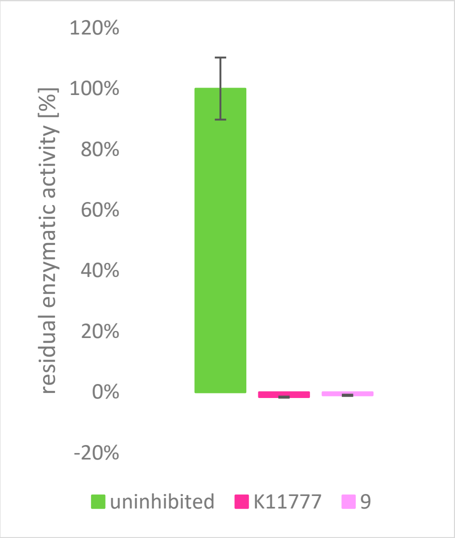

SI-Figure S6: Results of dilution assay to analyze reversibility of inhibition (n = 2). Raw activity of rhodesain after dilution was 0.68 ± 0.05 FU/s which is in the expected range for the concentration and instrument settings. Activity of rhodesain after incubation with either K11777 or **9** and subsequent dilution was −0.01 ±0.00 FU/s in both cases, indicating no recovery of enzymatic activity as expected from irreversible inhibitors.

Discussion on inactive compounds from strategies A and B with a focus on positional reasoning

The compounds containing a hPhe were designed to mimic K11777's binding interactions but overall did not show strong inhibition in the *in vitro* assay. The docking elucidated that this positioning can indeed be unfavorable for the used warhead as it greatly increased its distance from Cys-25. This is also reflected in their assay data: the *N*-terminal (*R)*-hPhe-containing peptides (K_i(**14**) = 27 µM, and K_i(**18**) = 12 µM; docking: arene positioned in S1') are weak inhibitors, probably through forcing the arene away from the catalytic Cys-25 if the (*R)*-hPhe side-chain is positioned optimally (as depicted for **18** in **SI-Figure S7-A**). In contrast, compounds with C-terminal (*S)*-hPhe are predicted to guide the *N*-terminal arene to the S2 pocket, similarly to **3** or **9**, but with different side-chain orientations (K_i(**17**) = 6 µM , and K_i(**13**) = 13 µM). However, since none of the hPhe-containing compounds showed striking inhibition, and the differences between them were marginal, this amino acid is unfavorable when employing *N*-terminal electrophilic arenes as warheads against rhodesain.

A B

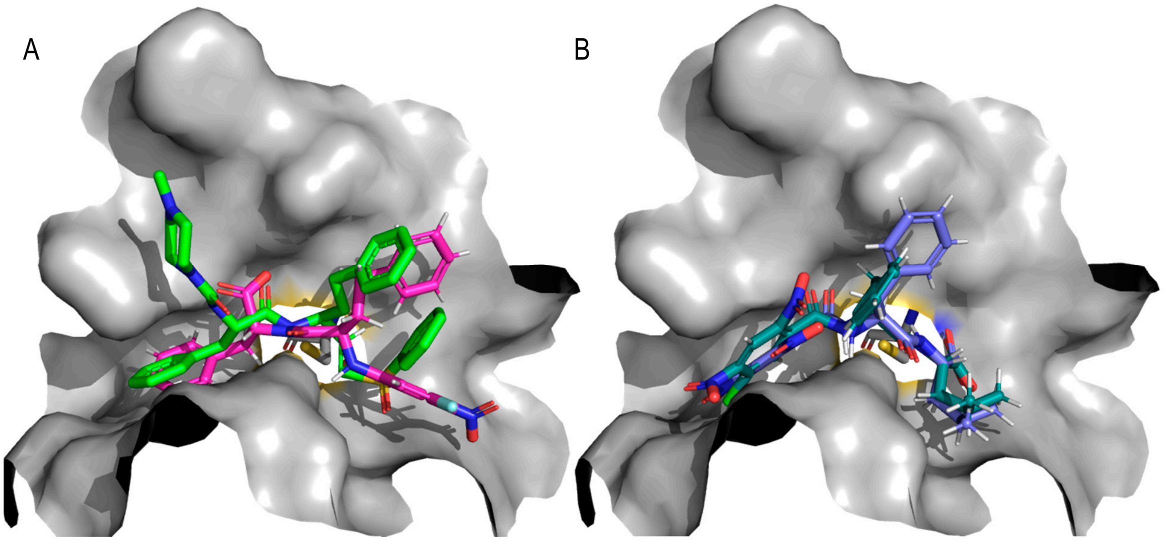

SI-Figure S7: **A**) Predicted binding mode for **18** (pink), overlayed with redocked K11777 (light green) in the active site of rhodesain (pdb: 2p7u). The positioning of Phe and hPhe side chains results in an unfavorable orientation of the electrophilic arene away from Cys-25. **B**) Predicted binding mode for **12** (dark green), and **9** (blue) in the active site of rhodesain (pdb: 2p7u). Both are predicted to be able to assume similar positioning of the arene but with larger distance to Cys-25 for the chlorine-substituted **12** (5.8 Å and 7.0 Å, as smallest distance, and distance to C-Cl, respectively).

Quantum-mechanical free energy calculations

The reaction paths include separately optimized reactants (R), the π-complex of the thiolate and the aromatic compound, transition states (TS), Meisenheimer/σ-complex and the product following the elimination of a fluoride anion (P). The free energies of the reactions are shown in **SI-Figure S8**.

These calculations are in line with the reactivity experiments for **3** and **9** described above and the published reactivity of **I**. Compound **I** slowly reacts with thiolate at room temperature [3], corresponding to the higher energy barrier, and the endergonic σ-complex. Compound **3** performs this reaction more readily, reflecting the lower energy barriers compared to **I**, and the exergonic progression. Finally, compound **9** with the most favorable energy profile for the reaction was shown to undergo substitution with the less nucleophilic thiol already.

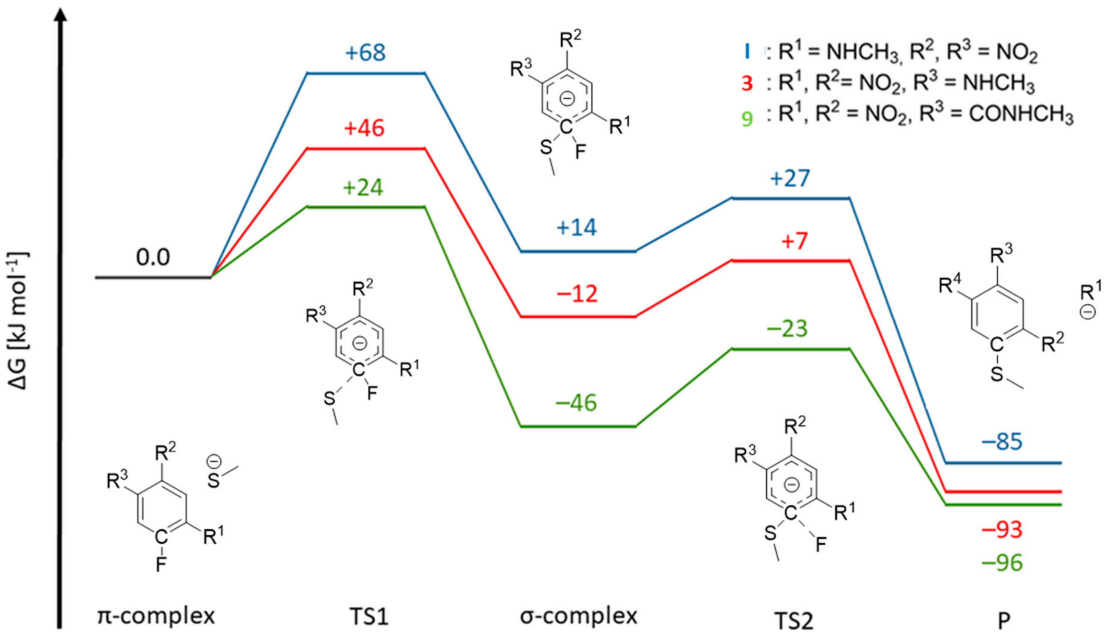

SI Figure S8: Free energy reaction paths for model compounds **I**, **3**, and **9.** The π-complex of the reactants, the transition state for the formation of the Meisenheimer complex (TS1), the Meisenheimer/σ-complex and the transition state for the elimination of the fluoride (TS2) were calculated, yielding the substitution product (P). The respective C-R^1 and S-CR^1 distances are given in **SI-Table S2**.

Methods

Rhodesain expression

Rhodesain was recombinantly expressed in *P. pastoris* according to a method adapted from literature [5,6]: The *Pichia pastoris* X-33 mutant, stably transformed with the rhodesain ΔC gene cloned into the pPICZαA vector, was cultured in buffered minimal glycerol supplemented with ampicillin (BMG_{Amp}) at 30 °C to an optical density (λ = 600 nm) of 2–3. To induce AOX1-controlled expression, BMG_{Amp} was exchanged for buffered minimal methanol with ampicillin (BMM_{Amp}). Incubation was continued for 72 h, and 0.5 % (V/V) methanol was added every 12 h. After this period, cells were pelleted by centrifugation and discarded. The culture supernatant containing secreted rhodesain was filtered (0.2 µm filter, cellulose acetate), adjusted to 2 M sodium chloride and loaded overnight onto a Phenylsepharose FF (high sub) column (V = 20 mL), equilibrated to chromatography conditions described below, attached to an ÄKTA start system. Hydrophobic interaction chromatography was performed by gradient elution with falling concentrations of sodium chloride (2 M to 0 M) in 20 mM sodium citrate buffer (pH 5.5). Rhodesain was eluted afterwards with MQ-water. The eluate was concentrated with centrifugal filter units (10 kDa MWCO, regenerated cellulose) to a volume ≤ 5 mL. Subsequently, size exclusion chromatography was performed with 20 mM sodium citrate buffer with 200 mM sodium chloride (pH 5.5) on an ÄKTA start system equipped with a HiLoad 16/600 Superdex 75 pg column. Rhodesain-containing fractions were pooled and then dialyzed against MQ-water for 4 h (dialysis tubing, 6 kDa MWCO, regenerated cellulose). The desalted rhodesain solution was lyophilized overnight and stored at ≤5 °C.

BMG_{Amp} / BMM_{Amp}:
1 % (V/V) glycerol OR 0.5 % (V/V) methanol
100 mM potassium phosphate buffer pH 6
3.4 g/L yeast nitrogen base without amino acids, without ammonium sulphate
10 g/L ammonium sulphate
0.4 mg/L biotin
100 mg/L ampicillin

Rhodesain, CatL, CatB Assays

Continuous fluorometric measurements with a Tecan Spark 10M reader in a 96 well plate format was used to evaluate activity [6]. Rhodesain, *Hs*CatB and *Hs*CatL were diluted in their respective activation buffer prior to use. Rhodesain was incubated in this for 30–60 min prior to use. The enzyme of interest (5 µL) was incubated with the inhibitor in question (10 µL), and its specific substrate (5 µL) in the respective assay buffer. The fluorescence signal was measured every 30 s for 10 min at room temperature with the corresponding excitation/emission wavelengths (λ_{ex} = 380 nm / λ_{em} = 460 nm). The final concentrations and buffer compositions are depicted in **SI-Table S1**. Inner-filter effect corrections were performed as described in literature [1] if deviations >5% were detected.

SI-Table S1: Information on assay conditions. [E] = final enzyme concentration, [S] = final substrate concentration in well.

Enzyme	***Hs*CatB**	***Hs*CatL**	**Rhodesain (*Tb*CatL)**
Enzyme storage buffer	50 mM NaOAc, 1 mM EDTA, pH = 5.0	20 mM malonate, 400 mM NaCl, 1 mM EDTA, pH = 5.5	50 mM NaOAc, 200 mM NaCl, 5 mM EDTA, pH = 5.5
Activation buffer	50 mM TRIS, 200 mM NaCl, 5 mM EDTA, 2 mM DTT, pH = 6.5		50 mM NaOAc, 200 mM NaCl, 5 mM EDTA, 5 mM DTT, pH = 5.5
Assay buffer	50 mM TRIS, 200 mM NaCl, 5 mM EDTA, pH = 6.5		50 mM NaOAc, 200 mM NaCl, 5 mM EDTA, 0.005 % Brij 35, pH = 5.5
Manufacturer	EMD	EMD	In-house expression
[E]	3 nM	5 nM	0.5 nM
Substrate structure	Z-Phe-Arg-AMC		
[S]	100 µM	6.25 µM	10 µM
K_M	150 µM	6.5 µM	0.827 µM

For the evaluation of **9** in absence of DTT (depicted in **SI-Figure S5**): 5 nM final rhodesain concentration that was activated before in presence of TCEP (alternative activation buffer: 5 mM DTT → 5 mM TCEP) was used to generate a similar slope in a control measurement in absence of inhibitor as for the measurements with DTT.

Data evaluation.

Time-independent behavior

For inhibitors that behaved time-independently during the measurements (i.e., F-t-diagrams show constant slope), IC_{50} values using defined dilution series for each compound were calculated with GraphPad PRISM by fitting the remaining enzymatic activity to a four parameter IC_{50} equation with Y [%] as the residual enzyme activity, Y_{max} as the maximum value of the dose response curve at inhibitor concentrations [I] = 0 µM, Y_{min} as the minimum value at high inhibitor concentrations and s as the Hill coefficient.

$$y\ [\%residual\ activity] = \frac{y_{\max}-y_{\min}}{1+\left(\frac{[\mathrm{I}]}{\mathrm{IC}_{50}}\right)^{s}} + y_{\min} \qquad \textbf{eq. 1}$$

It is assumed that the inhibitors bind competitively in respect to the substrate [7]. Due to the dependence of the IC_{50} value on the substrate affinity and concentration, the K_i values were calculated with the Cheng-Prusoff equation, using the final substrate concentration [S] and the Michaelis-Menten constant K_M to generate comparable data [8].

$$K_{\mathrm{i}} = \frac{\mathrm{IC}_{50}}{1+\frac{[S]}{K_{\mathrm{M}}}} \qquad \textbf{eq. 2}$$

Time-dependent behavior

For inhibitors that behaved time-dependently during the measurements (i.e., F-t-diagrams show reducing slopes over time), K_I^{app} and k_{inact} values using defined dilution series for each compound were calculated with GraphPad PRISM by fitting the k_{obs} values (derived from plotting the measured F-t-diagram to **eq. 3**) to each concentration. The irreversible reaction necessary for this type of evaluation was shown with rhodesain (**SI-Figure 6**).

$$F_t = F_0 + (F_{max} - F_0) * (1 - e^{-k_{obs}*t}) \qquad \textbf{eq. 3}$$

$$k_{obs} = \frac{k_{inact}[I]}{K_I^{app}+[I]} \qquad \textbf{eq. 4}$$

It is assumed that the inhibitors bind competitively in respect to the substrate [7]. Due to the dependence of the K_I^{app} value on the substrate affinity and concentration, the K_I values were calculated with the Cheng-Prusoff equation, using the final substrate concentration [S] and the Michaelis-Menten constant K_M to generate comparable data.

$$K_{\mathrm{I}} = \frac{K_I^{app}}{1+\frac{[S]}{K_{\mathrm{M}}}} \qquad \textbf{eq. 5}$$

MALDI-TOF experiment

Mass spectrometric experiments were performed as described before [9]. Lyophilized rhodesain was first reconstituted at 10 µM in buffer containing reducing agent (pH = 5.5, 50 mM NaOAc, 200 mM NaCl, 5 mM EDTA, 5 mM DTT) and incubated at r.t. for 30–60 min to ensure full activation of the enzyme. Afterwards, each inhibitor was added to 100 µL of 10 µM activated rhodesain solution (final concentrations: 100 µM inhibitor, 2.5 % DMSO).

Prior to MS analysis, protein alone or protein-inhibitor complexes were desalted by using Zeba Spin Desalting Columns (7 kDa MWCO, 0.5 mL; Thermo Fisher Scientific) in accordance with the manufacturer's instructions. On the target, desalted sample solutions were mixed 1:1 with a MALDI-matrix: Sinapinic acid, saturated solution in ACN/water 1:1 with 0.1 % TFA. Then, the mixtures were left in the fume hood for cocrystallization until dry.

After evaporation of the solvents (ca. 15 min), measurements were carried out on a rapifleX MALDI-TOF/TOF mass spectrometer (Bruker Daltonik GmbH, Bremen, Germany). The instrument is equipped with a scanning smart beam 10 kHz Nd:YAG laser at a wavelength of 355 nm and a 10 bit 5 GHz digitizer. The acceleration voltage was set to 20 kV and the mass spectra were recorded in positive ion linear mode. Calibration was done with the Bruker protein calibration standard II in a mass range from 10 to 70 kDa. Samples were measured at a laser power of 70–100 % with random walk ionization across the sample spot. As control sample, rhodesain incubated with compound K11777, a known covalent irreversible inhibitor, was used which showed the expected mass shift representative of covalent adduct formation (data now shown).

Data analysis was performed using the open-source software mMass [10]. $[M+H]^+$ was evaluated, so cropping from 22–27 kDa was performed. Signal intensity was normalized to highest signal in this range. Baseline was corrected with standard settings. Mass shifts were calculated from rhodesain signal in negative control (m/z was 23383 ± 22 Da (n = 5)) subtracted from adduct peak in the respective sample, if present.

PAMPA

A general literature-known principle was used [11]. Propranolol hydrochloride was sourced from Changzou Yabang Pharmaceutical (Fagron GmbH & Co. KG). Candesartan cilexetil was sourced as a chemical reference standard from the EDQM (catalogue code Y0001388). Candesartan was liberated and purified from this as described at the end of the synthesis section.

Incubation setup consisted of donor (top) plate (Sigma Aldrich, MAIPNTR10), 5 µL artificial membrane (1 % (w/v) L-α-phosphatidyl choline, Sigma Aldrich P3556, in *n*-dodecane, Sigma Aldrich 8205430100), acceptor (bottom) plate (Greiner, 655074). Measurement setup for direct spectroscopy consisted of UV-transparent measurement plate (Greiner UV-Star®, 655801), Tecan Spark 10M® well plate reader, 200 µL sample volume, λ = 200–650 nm. Analysis of absorption spectra was performed with AUC function in GraphPad Prism. Measurement setup for LCMS consisted of an Agilent 1100 series HPLC system coupled to an Agilent 1100 series LC/MSD Trap with electron spray ionization (ESI). An Agilent Poroshell 120 EC-C18, 150x2.10 mm, 4 µm column or an Agilent Zorbax SB-Aq 4.6 × 150 mm, 5 µm column was used. A linear gradient was used for elution with a ternary pump using [water/ACN/water + 0.1 % formic acid] that changes ratios from 80/10/10 to 0/90/10 over the course of 6 min, followed by 4 min of isocratic elution (0.7 mL/min). Injection volume was 100 µL. Areas of peaks in the chromatogram (detection-λ = 210 nm) were used to calculate the AUCs. Retention time and mass spectrum recorded in positive ionization mode were used to assign species. Analysis was performed using MestreNova.

Compound solutions were diluted from 20 mM stock solution in DMSO to 100 µM in a buffered (DPBS pH 7.4 or TRIS 50 mM pH 7.4) aqueous solution with 5 % (TRIS) or 50 % (DPBS) final content of DMSO ("donor solution"). Similarly prepared solutions (buffer + solvent) were used as "acceptor solutions". 150 µL of donor solution was applied onto the artificial membrane which had been applied first to the donor plate. This was sealed (Greiner, 676070, Viewseal sealer). 400 µL of acceptor solution was applied to the acceptor plate. Incubation setup was assembled and left for 7 h. After this time, acceptor and reference solutions were analyzed.

The experiment was performed in duplicates on each day. The acceptor solutions of those were measured separately (direct spectroscopic detection) or mixed 1:1 prior to analysis (LCMS detection). Reference solutions were prepared by simply mixing the indicated volumes of donor and acceptor solutions at the start of the incubation period and analyzed later with the acceptor solutions.

Calculations of apparent permeability P_{app} were performed using the following equation with V_D and V_A as volumes of donor and acceptor solutions (0.15 cm^3 and 0.4 cm^3), AUC_A and AUC_{Eq} as the area of the measured and baseline-corrected spectrum of sample and reference solutions, A as the porosity-corrected filter area (0.3019 cm^2*0.7 = 0.2113 cm^2) and t as the incubation time given in seconds.

$$P_{app} = -\frac{V_D * V_A * \ln\left(1 - \frac{AUC_A}{AUC_{Eq}}\right)}{(V_D + V_A) * A * t} \qquad \textbf{eq. 6}$$

All compounds except for esters **20** and **21** were evaluated under standard conditions suitable for both LC-based and direct spectroscopic quantification (5% DMSO, TRIS buffer pH 7.4). Since these esters were found to have poor aqueous solubility to an extent that complicated detection, 50 % DMSO in DPBS

was used for these compounds to ensure dissolution and spectroscopy was used for quantification (**2**, **3**, propranolol and methotrexate were evaluated like this additionally and behaved identically in both conditions).

<u>Docking</u>

Docking was performed as described previously [7]. In short, the crystal structure containing K11777 (pdb: 2p7u) [12] was used for docking without retention of crystallized water. The ligands were energy minimized with MMFF94x forcefield [13] in MOE (v.2022.02, Molecular Operating Environment, Chemical Computing Group Inc., Canada). Docking calculations were performed with FlexX (v.2.3.2, LeadIT/FlexX, BiosolveIT GmbH, Germany) and the top 10 poses were manually inspected for binding interactions in Pymol (v.2.4.0, Schrödinger).

<u>QM calculations</u>

All calculations were performed with the ORCA 5.0.4 program package [14]. Geometry optimizations were performed with ωB97X-D3 [15,16] \ma-def2-SVP [17,18] with the AutoAux auxiliary basis sets [19]. All stationary points were confirmed by frequency analysis and implicit solvation in water was included with the CPCM solvation method [20]. Free energies included a concentration correction resulting from the change in standard states going from gas phase to condensed phase [21,22]. The structures depicted in **SI-Figure S9** were utilized as model compounds with methyl thiolate as the model nucleophile. Starting in the π-complex, the S-CR^1 distances were gradually shortened in a relaxed scan. Subsequently, the highest energy point was subjected to a TS optimization to confirm a true transition state. The same procedure was conducted for TS2, elongating the C-R^1 distances starting from the σ-complex. The C-R^1 and S-CR^1 distances for all calculated structures are given in **SI-Table S2**.

SI Figure S9: Model compounds utilized for QM reaction path calculations.

SI-Table S2: Distances of the leaving group to the attacked aromatic carbon atom (C-R^1) as well as from the thiolate to the carbon atom (S-CR^1) for all computed structures of compounds **I**, **3**, and **9**.

[Å]	Cpd **I**		Cpd **3**		Cpd **9**	
	C-R^1	S-CR^1	C-R^1	S-CR^1	C-R^1	S-CR^1
π-complex	1.34	4.26	1.42	4.68	1.32	4.21
TS1	1.36	2.43	1.35	2.47	1.33	2.57
σ-complex	1.43	1.85	1.44	1.86	1.44	1.84
TS2	1.75	1.80	1.81	1.80	1.85	1.77
P	-	1.76	-	1.75	-	1.74

Synthetic Procedure and Compound Characterization

Solvents and reagents

Unless stated otherwise, all solvents and reagents were obtained from commercial suppliers and used without prior purification.

Chromatography

Preparative column chromatography was performed using an Isolera One automatic flash chromatography system (Biotage) using either cyclohexane and ethyl acetate (35–70 µm, Acros Organics normal phase) or water and acetonitrile (C18 modified silica, reverse phase).
Thin-layer chromatography (TLC) was carried out on silica plates (TLC Silica 60 F254, Merck, Darmstadt). Visualization of the compounds was accomplished by illumination with UV-light of the developed plates and by staining with potassium permanganate reagent.
Preparative HPLC was performed on an Agilent Technologies 1290 Infinity II system with two high-pressure gradient K-1800 pumps and an S-260-UV-DAD detector. The separation took place on ACE 5 C18-PFP (particle size: 5 µm, length: 150 mm, diameter: 30 mm, flow rate: 42.5 mL/min^{-1}) or Macherey-Nagel Nucleodur C18-HTEC (particle size: 5 µm, length: 150 mm, diameter: 32 mm, flow rate: 42.5 mL/min^{-1}) columns. For both analytical and preparative HPLC, the eluent mixtures of solvents A: H_2O (LCMS grade and Milli-Q) + 0.1 % formic acid (LCMS grade) and B: MeCN (HPLC and LCMS grade) were given in the v/v ratio.

NMR spectra

NMR spectra were recorded on a Bruker Avance-III HD (^{1}H-NMR: 300 MHz, ^{13}C-NMR: 75.5 MHz, ^{19}F-NMR: 282 MHz) or a Bruker Avance-II (^{1}H-NMR: 400 MHz, ^{13}C-NMR: 100.6 MHz) spectrometer. Chemical shifts are referenced to residual solvent signals ($CDCl_3$: 7.26 ppm and 77.16 ppm, DMSO-d_6: 2.50 ppm and 39.52 ppm for ^{1}H-NMR and ^{13}C-NMR, acetonitrile-d_3: ^{1}H-NMR δ/ppm = 1.94, ^{13}C-NMR δ/ppm = 118.7 respectively) and reported in parts per million (ppm) relative to tetramethylsilane (TMS). Multiplicities of NMR signals are abbreviated as follows: br = broad, s = singlet, d = doublet, t = triplet, q = quartet, m = multiplet and combinations thereof, app = apparent.

Mass spectra

Electron spray ionization (ESI) mass spectra were recorded on a 1200-series HPLC- system (Agilent-Technologies) with binary pump and integrated diode array detector coupled to an LC/MSD-Trap-XTC-mass spectrometer (Agilent-Technologies) or on a Micromass-Q-TOF-Ultima-3-mass spectrometer (Waters). High resolution mass spectra were recorded on a Micromass-Q-TOF-Ultima-3-mass spectrometer (Waters) with LockSpray-interface and a suitable external calibrant.

IR-Spectroscopy

Infrared spectroscopy was performed on a Bruker Tensor 27 FT-IR spectrometer including a diamond ATR unit.

Optical rotation measurements

Optical rotation measurements were accomplished with a PerkinElmer 241MC polarimeter at 589 nm.

Melting Points

Melting points were determined in open capillary tubes using a Krüss-Optronic (Hamburg, Germany) KSP 1 N thermoelectric melting point meter

General procedure for amide-coupling (GP1)

To a round bottom flask benzyloxycarbonyl (Cbz) protected amino acid (1.01 Eq.), hydroxybenzotriazole monohydrate (HOBt·H_2O 1.01 Eq.), 1-ethyl-3-(3-dimethylaminopropyl)carbodiimide hydrochloride (EDC·HCl, 1.01 Eq.), 4-dimethylaminopyridine (DMAP, 1.01 Eq.) and the hydrochloride of the corresponding *tert*-butyl protected amino acid (1.0 Eq.) were added and dissolved in dry dichloromethane (DCM, ca. 0.1 M). Triethylamine (2.0 Eq.) was added, and the reaction stirred for 12 h. After the addition of water (20 mL) the phases were separated, and the organic phase was washed with saturated ammonium chloride solution. Afterwards, the organic phase was dried over sodium sulfate (Na_2SO_4), filtered and the solvent removed under reduced pressure. The product was used without further purification.

General procedure (GP2) for the deprotection of Cbz-protected amines

In a round bottom flask, the Cbz-protected amine was dissolved in ethanol (EtOH) or tetrahydrofurane (THF) and 10 wt% of Pd/C (5 wt%) was added. The flask was evacuated and flushed with hydrogen three times and then stirred under a slight overpressure of hydrogen until TLC showed full conversion of the starting material (roughly one hour in ethanol, 24 h in THF). The reaction mixture was filtered through celite, and the solvent evaporated.

General procedure (GP3) for the S_NAr

To a 0.1 M solution of the amine in ethanol the corresponding aromatic compound (1.0 Eq.) was added. *N,N*-Diisopropylethylamine (DIPEA, 2.0 Eq.) was added, and the reaction mixture was stirred until TLC showed full conversion. Twice as much water as ethanol was added and the resulting mixture was extracted three times with ethylacetate (EtOAc, roughly the same volume as the used ethanol). The combined organic extracts were dried over Na_2SO_4, and the solvent was removed under reduced pressure. The crude product was purified using flash column chromatography.

General procedure (GP4) for the deprotection of *tert*-butyl protected acids

To an ice-cold solution of the corresponding ester in DCM (1 M), trifluoroacetic acid (TFA, 50 Eq.) was added. The reaction mixture was stirred for 3 h at rt and the solvent was removed under reduced pressure.

Traces of TFA were removed via co-evaporation with toluene. The crude product was purified either via column chromatography or preparative HPLC.

General procedure (GP5) for solid-phase synthesis

1. Loading and Capping

3.7 g Chlorotriethylchloride resin was washed with DCM for 30 min and the DCM was filtered. The fluorenylmethoxycarbonyl (Fmoc) protected amino acid (0.81 mmol) was dissolved in 100 mL of a 4 % collidine/DCM mixture and added to the resin. The mixture was shaken for 12 h, the solvent filtered, and the resin washed three times with 25 mL DCM.
20 mL of a capping solution (DCM/MeOH/DIPEA, 17:2:1) was added and stirred for 1 h at rt. The solvent was filtered, and the resin was washed four times with 20 mL DCM and dimethylformamide (DMF).

2. Determination of the loading

1.5 mg of the resin were shaken in 1 mL of a 20 % piperidine/DMF mixture for 30 min. The solution was filtered and diluted with 5 mL of methanol (MeOH). The mixture was then transferred to a cuvette. An absorption spectrum was measured, and the loading B was calculated with the following equation:

$$B = \frac{A_{289nm} \times V}{\varepsilon_{289nm} \times d \times m} \quad \textbf{eq. 7}$$

3. Coupling on the solid phase

The resin was washed three times with 20 mL DMF and then shaken for 1 h with 20 mL of a 20 % piperidine/DMF solution. The solvent was filtered and a solution consisting of the corresponding amino acid (5.0 Eq.), HATU (4.5 Eq.) and HOAt (4.5 Eq.) in 20 mL of a 20 % piperidine/DMF was added, and the mixture was shaken for 12 h.

4. Capping and Fmoc-deprotection

The resin was filtered from the solvent and washed three times with the same volume of DMF and then three times with 25 mL DCM. 20 mL of the capping solution was added and shaken for 1 h at rt. The solution was filtered, and the resin was washed four times with 20 mL DCM and DMF. 20 mL of a 20 % piperidine/DMF mixture was added and shaken for 1 h. The resin was again washed five times with 20 mL of DMF and three times with DCM.

5. Aromatic substitution and cleavage from the resin

1,3-Difluoro-4,6-dinitrobenzene (363 mg, 1.78 mmol, 2.2 Eq.) in 20 mL EtOH was added to the resin. DIPEA (0.28 mL, 1.62 mmol, 2.0 Eq.) was added and shaken over night at rt. The solution was filtered, and the resin washed three times with 20 mL DCM. The resin was dried for 1 h and shaken with a mixture of 18 mL TFA, 1 mL H_2O and 1 mL of triisopropylsilane for 3 h. The mixture was filtered and removed under reduced pressure. The crude product was purified by preparative HPLC.

Final compounds, strategy A

1 Synthesis of *N*-(2-nitrophenyl)-L-phenylalanyl-L-leucine

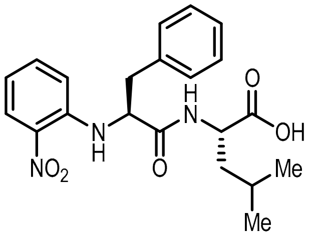

This compound was prepared starting from **19** (106 mg, 0.23 mmol) following GP4. The product was obtained as a yellow solid (80 mg, 0.20 mmol, 86 %).

Rf. = 0.16 (DCM/MeOH, 5:0.3).

MS (ESI): m/z (%) = 400.2 (100) $[M+H]^+$

HRMS (ESI): 400.1858 ($[M+H]^+$, calc. for $C_{21}H_{26}N_3O_5^+$: 400.1867).

Mp. = 72.1 °C (decomp.)

$[\alpha]_D^{22}$ = +76.2° (c = 1.00 g/mL, CH_2Cl_2).

^{1}H-NMR, COSY (600 MHz, DMSO-d_6) δ/ppm = 12.72 (s, 1H, OH), 8.62 (d, J = 8.2 Hz, 1H, NH^{Leu}), 8.17 (d, J = 7.5 Hz, 1H, NH^{Phe}), 8.04 (dd, J = 8.6, 1.6 Hz, 1H, H-3), 7.47 (ddd, J = 8.6, 6.9, 1.6 Hz, 1H, H-5), 7.30 – 7.23 (m, 4H, o,m-H), 7.23 – 7.17 (m, 1H, m-H), 6.79 (dd, J = 8.9, 1.2 Hz, 1H, H-6), 6.70 (ddd, J = 8.4, 6.9, 1.2 Hz, 1H, H-4), 4.56 (td, J = 7.7, 4.8 Hz, 1H, α-CH^{Phe}), 4.27 (ddd, J = 10.1, 8.1, 4.8 Hz, 1H, α-CH^{Leu}), 3.21 (dd, J = 13.9, 4.8 Hz, 1H, β-CH_2^{Phe}), 3.05 (dd, J = 13.9, 8.0 Hz, 1H, β-CH_2^{Phe}), 1.68 – 1.47 (m, 3H, β-CH_2^{Leu} and γ-CH^{Leu}), 0.89 (d, J = 6.4 Hz, 3H, δ-CH_3^{Leu}), 0.80 (d, J = 6.4 Hz, 3H, δ-CH_3^{Leu}).

^{13}C-NMR, HSQC, HMBC (151 MHz, DMSO) δ/ppm = 174.3 (CO^{Leu}), 171.0 (CO^{Phe}), 144.4 (C1), 137.1 (C_q^{Ar}), 137.0 (C5), 132.0 (C2), 129.8 (2C, m-CO^{Phe}), 128.8 (2C, o-C^{Pher}), 127.2 (p-C^{Phe}), 126.7 (C3), 116.6 (C4), 115.1 (C6), 57.5 (α-CH^{Phe}), 50.7 (α-CH^{Leu}), 40.3 (β-CH_2^{Leu}), 38.6 (β-CH_2^{Phe}), 24.8 (γ-CH^{Leu}), 23.4 (δ-CH_3^{Leu}), 21.5 (δ-CH_3^{Leu}).

IR (ATR): $\tilde{\nu}$ (cm^{-1}) = 3363, 2957, 2871, 1723, 1640, 1615, 1574, 1498, 1265, 1149.

2 Synthesis of *N*-(3,4-dinitrophenyl)-L-phenylalanyl-L-leucine

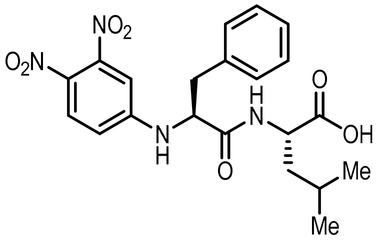

This compound was prepared starting from **20** (107 mg, 0.21 mmol) following GP4. The product was obtained as a colorless solid (86 mg, 0.15 mmol, 71 %).

Rf. = 0.17 (DCM/MeOH, 5:0.2).

MS (ESI): m/z (%) = 445.2 (100) $[M+H]^+$

HRMS (ESI): 445.1717 ($[M+H]^+$, calc. for $C_{21}H_{25}N_4O_7^+$: 445.1717).

Mp. = 98.8–100.1 °C

$[\alpha]_D^{22}$ = -7.6° (c = 1.00 g/mL, CH_2Cl_2).

^{1}H-NMR, COSY (400 MHz, DMSO) δ/ppm = 12.57 (s, 1H, OH), 8.55 (d, J = 8.2 Hz, 1H. NH^{Phe}), 8.16 (d, J = 8.0 Hz, 1H, NH^{Leu}), 7.97 (d, J = 9.3 Hz, 1H, H-5), 7.34 – 7.14 (m, 5H, H^{Ar}), 6.98 (s, 1H, H-2), 6.82 (s, 1H, H-6), 4.43 (td, J = 8.9, 4.8 Hz, 1H, α-CH^{Phe}), 4.32 – 4.20 (m, 1H, α-CH^{Leu}), 3.09 (dd, J = 13.9, 4.7 Hz, 1H, β-CH_2^{Phe}), 2.91 (dd, J = 14.0, 9.2 Hz, 1H, β-CH_2^{Phe}), 1.59 – 1.45 (m, 3H, β-CH_2^{Leu} and γ-CH^{Leu}), 0.86 (d, J = 5.7 Hz, 3H, δ-CH_3^{Phe}), 0.75 (d, J = 5.8 Hz, 3H, δ-CH_3^{Leu}).

^{13}C-NMR HSQC, HMBC (101 MHz, DMSO) δ/ppm = 174.1 (CO^{Leu}), 170.9 (CO^{Phe}), 154.3 (C^{Ar}), 147.3 (C^{Ar}), 137.7 (C^{Ar}), 129.6 (C^{Ar}), 128.7 (C^{Ar} and C-5), 127.4 (C^{Ar}), 127.0 (C^{Ar}), 57.8 (α-CH^{Phe}), 50.7 (α-CH^{Leu}),40.5 (β-CH_2^{Leu}), 38.2 (β-CH_2^{Phe}), 24.8 (γ-CH^{Leu}), 23.3 (δ-CH_3^{Leu}), 21.1 (δ-CH_3^{Leu}).

IR (ATR): $\tilde{\nu}$ (cm^{-1}) = 3277, 2925, 1718, 1659, 1604, 1543, 1369, 1319, 1255, 744.

3 Synthesis of *N*-(5-fluoro-2,4-dinitrophenyl)-L-phenylalanyl-L-leucine

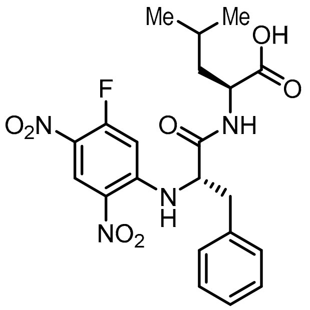

This compound was prepared starting from **21** (193 mg, 0.37 mmol) following GP4. The product was obtained as an intensively yellow solid (158 mg, 0.34 mmol, 92 %).

Rf. = 0.49 (DCM/MeOH, 5:0.2).

MS (ESI): m/z (%) = 463.1(100) $[M+H]^+$

HRMS (ESI): 463.1618 ($[M+H]^+$, calc. for $C_{21}H_{24}FN_4O_7^+$:463.1623).

Mp. = 200.1–201.3 °C

$[\alpha]_D^{22}$ = -14.1° (c = 1.00 g/mL, $CHCl_3$/MeCN, 1:1).

^{1}H-NMR, COSY (400 MHz, DMSO) δ/ppm = 12.75 (s, 1H, COOH), 8.84 (d, J = 8.1 Hz, 1H, H-3$^{Ar\text{-}F}$), 8.77 (dd, J = 7.6, 1.6 Hz, 1H, NH^{Phe}), 8.62 (d, J = 8.1 Hz, 1H, NH^{Leu}), 7.34 – 7.08 (m, 5H, H^{Ar}), 6.69 (d, J = 14.4 Hz, 1H, H-6$^{Ar\text{-}F}$), 4.74 (td, J = 7.6, 4.8 Hz, 1H, α-CH^{Phe}), 4.30 (ddd, J = 9.9, 8.0, 4.9 Hz, 1H, α-CH^{Leu}), 3.28 (dd, J = 13.9, 4.8 Hz, 1H, β-CH$_2^{Phe}$), 3.13 (dd, J = 13.9, 7.7 Hz, 1H, β-CH$_2^{Phe}$), 1.68 – 1.50 (m, 3H, γ-CH^{Leu} and β-CH$_2^{Leu}$), 0.90 (d, J = 6.2 Hz, 3H, δ-CH$_3^{Leu}$), 0.83 (d, J = 6.2 Hz, 3H, δ-CH$_3^{Leu}$).

^{19}F-NMR (377 MHz, DMSO) δ/ppm = -108.19 (dd, J = 14.4, 8.2 Hz).

^{13}C-NMR, HSQC, HMBC (101 MHz, DMSO) δ/ppm = 173.5 (CO^{Leu}), 169.1 (CO^{Phe}), 160.0 (C-5$^{Ar\text{-}F}$), 157.4 (C-4$^{Ar\text{-}F}$), 148.0 (d, J = 14.0 Hz, C-2$^{Ar\text{-}F}$), 136.0 (C^{Ar}), 129.5 (C^{Ar}), 128.3 (C^{Ar}), 127.2 (C^{Ar}), 127.0 (d, J = 30.9 Hz, C-3$^{Ar\text{-}F}$), 125.2 (d, J = 9.6 Hz, C-1$^{Ar\text{-}F}$), 102.3 (d, J = 27.5 Hz, C-6$^{Ar\text{-}F}$), 57.4 (α-CH^{Phe}), 50.3 (α-CH^{Leu}), 39.6 (β-CH$_2^{Leu}$), 37.7 (β-CH$_2^{Phe}$), 24.2 (γ-CH^{Leu}), 22.8 (δ-CH$_3^{Leu}$), 21.0 (δ-CH$_3^{Leu}$).

IR (ATR): $\tilde{\nu}$ (cm^{-1}) = 3352, 2959, 1725, 1629, 1578, 1504, 1261, 1227, 1072, 751.

4 Synthesis of *N*-(4-nitrophenyl)-L-phenylalanyl-L-leucine

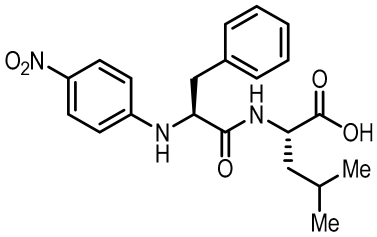

This compound was prepared starting from **22** (166 mg, 0.36 mmol) following GP4. The product was obtained as a yellow solid (123 mg, 0.23 mmol, 65 %).

Rf. = 0.16 (DCM/MeOH, 5:0.3).

MS (ESI): m/z (%) = 400.2 (100) $[M+H]^+$

HRMS (ESI): 400.1864 ($[M+H]^+$, calc. for $C_{21}H_{26}N_3O_5^+$: 400.1867).

Mp. = 95.6 °C (decomp.)

$[\alpha]_D^{22}$ = -+32.0° (c = 1.00 g/mL, CH_2Cl_2).

^{1}H-NMR, COSY (600 MHz, DMSO-d_6) δ/ppm = 12.66 (s, 1H, OH), 8.57 (d, J = 8.2 Hz, 1H, NH^{Leu}), 7.94 – 7.89 (m, 2H, H-3,5), 7.54 (d, J = 8.5 Hz, 1H, NH^{Phe}), 7.38 – 7.31 (m, 2H, o-H), 7.29 – 7.23 (m, 2H, m-H), 7.21 – 7.13 (m, 1H, p-H), 6.63 (d, J = 8.9 Hz, 2H, H-2,6), 4.36 (td, J = 9.1, 4.5 Hz, 1H, α-CH^{Phe}), 4.24 (ddd, J = 10.1, 8.1, 4.9 Hz, 1H, α-CH^{Leu}), 3.07 (dd, J = 14.0, 4.3 Hz, 1H, β-CH_2^{Phe}), 2.90 (dd, J = 14.0, 9.6 Hz, 1H, β-CH_2^{Phe}), 1.67 – 1.48 (m, 3H, β-CH_2^{Leu} and γ-CH^{Leu}), 0.88 (d, J = 6.5 Hz, 3H, δ-CH_3^{Leu}), 0.77 (d, J = 6.4 Hz, 3H, δ-CH_3^{Leu}).

^{13}C-NMR, HSQC, HMBC (151 MHz, DMSO) δ/ppm = 174.3 (CO^{Leu}), 171.8 (CO^{Ala}), 154.4 (C-2,6), 138.2 (C_q^{Ar}), 136.6 (2C, C-1,4), 129.6 (o-C), 128.6 (m-C), 126.9 (p-C), 126.4 (2C, C-3,5), 79.7 (chloroform impurity), 57.8 (α-CH^{Phe}), 50.7 (α-CH^{Leu}), 40.5 (β-CH_2^{Leu}), 38.4 (β-CH_2^{Phe}), 24.8 (γ-CH^{Leu}), 23.4 (δ-CH_3^{Leu}), 21.6 (δ-CH_3^{Leu}).

IR (ATR): $\tilde{\nu}$ (cm^{-1}) = 3335, 2958, 1719, 1648, 1597, 1472, 1305, 1109, 751, 696.

5 Synthesis of *N*-(5-fluoro-2-nitrophenyl)-L-phenylalanyl-L-leucine

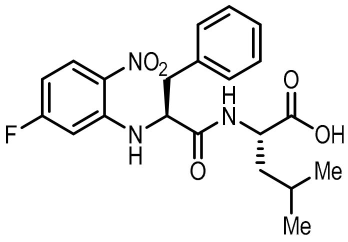

This compound was prepared starting from **23** (179 mg, 0.38 mmol) following GP4. The product was obtained as a colorless solid (164 mg, 0.41 mmol, quant.).

Rf. = 0.14 (DCM/MeOH, 5:0.2).

MS (ESI): m/z (%) = 418.2 (100) $[M+H]^+$

HRMS (ESI): 418.1765 ($[M+H]^+$, calc. for $C_{21}H_{25}FN_3O_5^+$: 418.1773).

Mp. = 162.3–164.0 °C

$[\alpha]_D^{22}$ = +62.9° (c = 1.00 g/mL, $CHCl_3$).

^{1}H-NMR, COSY (400 MHz, DMSO) δ/ppm = 12.73 (s, 1H, OH), 8.60 (d, J = 8.2 Hz, 1H, NH^{Leu}), 8.29 (dd, J = 7.6, 2.0 Hz, 1H, NH^{Phe}), 8.14 (dd, J = 9.4, 6.2 Hz, 1H, H-$3^{Ar\text{-}F}$), 7.33 – 7.16 (m, 5H, H^{Ar}), 6.61 – 6.47 (m, 2H, H-$4^{Ar\text{-}F}$ and H-$6^{Ar\text{-}F}$), 4.55 (td, J = 7.8, 4.7 Hz, 1H, α-CH^{Phe}), 4.28 (ddd, J = 10.0, 8.1, 4.7 Hz, 1H, α-CH^{Leu}), 3.21 (dd, J = 13.9, 4.7 Hz, 1H, β-CH_2^{Phe}), 3.05 (dd, J = 13.9, 8.2 Hz, 1H, β-CH_2^{Phe}), 1.68 – 1.47 (m, 3H, β-CH_2^{Leu} and γ-CH^{Leu}), 1.03 – 0.60 (m, 6H, δ-CH_3^{Leu}).

^{19}F-NMR (377 MHz, DMSO) δ/ppm = -101.66 (dt, J = 13.0, 7.0 Hz).

^{13}C-NMR, HSQC, HMBC (101 MHz, DMSO) δ/ppm = 174.2 (CO^{Leu}), 170.6 (CO^{Phe}), 167.1 (d, J = 253.3 Hz, C-$5^{Ar\text{-}F}$), 146.5 (d, J = 13.6 Hz, C-$1^{Ar\text{-}F}$), 137.0 (C^{Ar}), 130.3 (d, J = 12.5 Hz, C-$3^{Ar\text{-}F}$), 129.8 (C^{Ar}), 129.2 (C-$2^{Ar\text{-}F}$) 128.8 (C^{Ar}), 127.3 (C^{Ar}), 104.9 (d, J = 24.9 Hz, C-$4^{Ar\text{-}F}$), 100.67 (d, J = 27.9 Hz, C-$6^{Ar\text{-}F}$), 57.7 (α-CH^{Phe}), 50.7 (α-CH^{Leu}), 40.3 (β-CH_2^{Leu}), 38.5 (β-CH_2^{Phe}), 24.8 (γ-CH^{Leu}), 23.4 (δ-CH_3^{Leu}), 21.4 (δ-CH_3^{Leu}).

IR (ATR): ṽ (cm^{-1}) =3352, 2959, 2931, 1725, 1629, 1579, 1415, 1261, 1227, 751.

6 Synthesis of *N*-(6-chloropyrimidin-4-yl)-L-phenylalanyl-L-leucine

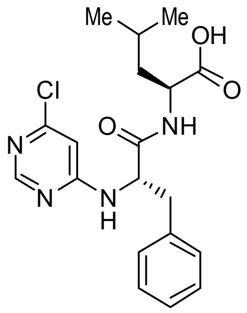

This compound was prepared starting from **24** (193 mg, 0.43 mmol) following GP4. The product was obtained as a colorless solid (156 mg, 0.40 mmol, 92 %).

Rf. = 0.15 (DCM/MeOH, 5:0.2).

MS (ESI): m/z (%) = 391.1(100) $[M+H]^+$

HRMS (ESI): 389.1367 ($[M-H]^-$, calc. for $C_{19}H_{22}ClN_4O_3{}^-$: 389.1386).

Mp. = 98.2–99.3 °C

$[\alpha]_D^{22}$ = -17.6° (c = 1.00 g/mL, $CHCl_3$).

^{1}H-NMR, COSY (600 MHz, Acetonitrile-d_3) δ/ppm = 8.21 (d, J = 1.0 Hz, 1H, H-2^{pyr}), 7.26 (d, J = 5.3 Hz, 4H, H^{Ar}), 7.25 – 7.18 (m, 1H, H^{Ar}), 7.10 (d, J = 8.0 Hz, 1H, NH^{Leu}), 6.51 (d, J = 7.9 Hz, 1H, NH^{Phe}), 6.44 (s, 1H, H-5^{pyr}), 4.84 (s, 1H, α-CH^{Phe}), 4.39 (ddd, J = 9.6, 8.0, 5.2 Hz, 1H, α-CH^{Leu}), 3.22 (dd, J = 14.1, 5.0 Hz, 1H, β-$CH_2{}^{Phe}$), 2.96 (dd, J = 14.1, 8.8 Hz, 1H, β-$CH_2{}^{Phe}$), 1.69 – 1.54 (m, 3H, β-$CH_2{}^{Leu}$ and γ-CH^{Leu}), 0.91 (d, J = 6.3 Hz, 3H, δ-$CH_3{}^{Leu}$), 0.86 (d, J = 6.3 Hz, 3H, δ-$CH_3{}^{Leu}$).

^{13}C-NMR, HSQC, HMBC (151 MHz, CD_3CN) δ/ppm = 172.2 (CO^{Leu}), 162.1 (CO^{Phe}), 157.3 (C-2^{pyr}), 136.3 (C-4^{pyr}), 128.6 (C^{Ar}), 127.5 (C^{Ar}), 125.9 (C^{Ar}), 103.6 (C-5^{pyr}), 54.9 (α-CH^{Phe}), 49.8 (α-CH^{Leu}), 39.1 (β-$CH_2{}^{Leu}$), 36.8 (β-$CH_2{}^{Phe}$), 23.6 (γ-CH^{Leu}), 21.4 (δ-$CH_3{}^{Leu}$), 19.7 (δ-$CH_3{}^{Leu}$).

IR (ATR): ṽ (cm^{-1}) = 3279, 2956, 1727, 1664, 1589, 1498, 1440, 1204, 753, 698.

7 Synthesis of *N*-((4-Fluoro-3-nitrophenyl)carbamoyl)-L-phenylalanyl-L-leucine

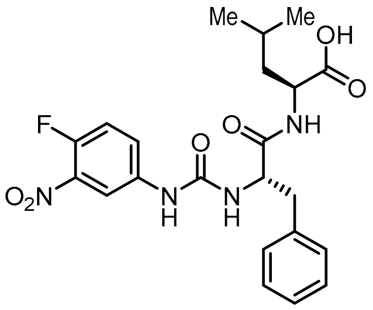

This compound was prepared starting from **25** (216 mg, 0.42 mmol) following GP4. The product was obtained as a colorless solid (190 mg, 0.41 mmol, 98 %).

Rf. = 0.18 (DCM/MeOH, 5:0.2).

MS (ESI): m/z (%) = 461.2 (100) $[M+H]^+$

HRMS (ESI): 459.1686 ($[M-H]^-$, calc. for $C_{22}H_{24}FN_4O_6^{--}$: 459.1685).

Mp. = 178.1–178.5 °C

$[\alpha]_D^{22}$ = -11.2° (c = 1.00 g/mL, CH_2Cl_2).

^{1}H-NMR, COSY (600 MHz, DMSO-d_6) δ/ppm = 12.69 (s, 1H, OH), 9.22 (s, 1H, H-2^{Ar-F}), 8.46 (d, J = 8.0 Hz, 1H, NH^{Leu}), 8.38 – 8.21 (m, 1H), 7.51 (ddd, J = 9.1, 3.8, 2.7 Hz, 1H, H-5^{Ar-F}), 7.43 (dd, J = 11.0, 9.1 Hz, 1H, H-6^{Ar-F}), 7.30 – 7.11 (m, 5H, H^{Ar}), 6.45 (d, J = 8.3 Hz, 1H, NH^{Phe}), 4.56 (td, J = 8.1, 4.6 Hz, 1H, α-CH^{Phe}), 4.27 (ddd, J = 9.8, 8.0, 5.2 Hz, 1H, α-CH^{Leu}), 3.07 (dd, J = 13.9, 4.6 Hz, 1H, β-CH_2^{Phe}), 2.83 (dd, J = 13.9, 8.0 Hz, 1H, β-CH_2^{Phe}), 1.65 (tdd, J = 13.1, 9.2, 5.9 Hz, 1H, β-CH_2^{Leu}), 1.61 – 1.49 (m, 2H, β-CH_2^{Leu} and γ-CH^{Leu}), 0.90 (d, J = 6.6 Hz, 3H, δ-CH_3^{Leu}), 0.85 (d, J = 6.5 Hz, 3H, δ-CH_3^{Leu}).

^{19}F-NMR (377 MHz, $CDCl_3$) δ/ppm = -129.79 (ddd, *J* = 10.9, 6.8, 4.0 Hz).

^{13}C-NMR, HSQC, HMBC (151 MHz, DMSO) δ/ppm = 174.0 (CO^{Leu}), 171.2 (CO^{Phe}), 154.2 (CO), 149.9 (C-4^{Ar-F}), 148.2 (C-3^{Ar-F}), 137.2 (C^{Ar}), 137.2 – 136.2 (m, C-1^{Ar-F}), 129.4 (C^{Ar}), 128.0 (C^{Ar}), 126.2 (C^{Ar}), 124.6 (d, J = 7.6 Hz, C-5^{Ar-F}), 118.5 (d, J = 21.9 Hz, C-6^{Ar-F}), 113.3 (d, J = 3.0 Hz, C-1^{Ar-F}), 79.1 (Chloroform), 53.4 (α-CH^{Phe}), 50.1 (α-CH^{Leu}), 40.0 (β-CH_2^{Leu}), 38.2 (β-CH_2^{Phe}), 24.2 (γ-CH^{Leu}), 22.8 (δ-CH_3^{Leu}), 21.2 (δ-CH_3^{Leu}).

IR (ATR): ṽ (cm^{-1}) = 3326, 3024, 1717, 1645, 1537, 1498, 1349, 1215, 752, 667.

8 Synthesis of *N*-(2,6-dichloronicotinoyl)-L-phenylalanyl-L-leucine

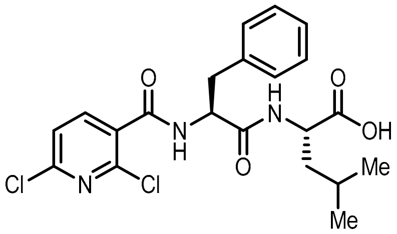

This compound was prepared starting from **26** (282 mg, 0.55 mmol) following GP4. The product was obtained as a colorless solid (225 mg, 0.50 mmol, 90 %).

Rf. = 0.15 (DCM/MeOH, 5:0.3).

MS (ESI): m/z (%) = 452.1 (100) $[M+H]^+$

HRMS (ESI): 452.1144 ($[M+H]^+$, calc. for $C_{21}H_{24}Cl_2N_3O_4^+$: 452.1139).

$[\alpha]_D^{22}$ = -2.7° (c = 1.00 g/mL, $CHCl_3$).

Mp. = 172.7–174.6 °C

^{1}H-NMR, COSY (600 MHz, DMSO) δ/ppm = 12.65 (s, 1H, OH), 8.92 (d, J = 8.6 Hz, 1H, NH^{Phe}), 8.41 (d, J = 7.9 Hz, 1H, NH^{Leu}), 7.66 – 7.55 (m, 2H, H^{Pyr}), 7.34 – 7.25 (m, 4H, o-H^{Ar} and m-H^{Ar}), 7.23 – 7.18 (m, 1H, p-H^{Ar}), 4.77 (ddd, J = 10.6, 8.6, 4.1 Hz, 1H, α-CH^{Phe}), 4.27 (ddd, J = 10.0, 7.9, 5.1 Hz, 1H, α-CH^{Leu}), 3.18 – 3.09 (m, 1H, β-CH_2^{Phe}), 2.81 (dd, J = 14.0, 10.6 Hz, 1H, β-CH_2^{Phe}), 1.69 (tdd, J = 13.2, 9.3, 5.9 Hz, 1H, γ-CH^{Leu}), 1.63 – 1.51 (m, 2H, β-CH_2^{Leu}), 1.00 – 0.70 (m, 6H, δ-CH_3^{Leu}).

^{13}C-NMR, HSQC, HMBC (151 MHz, DMSO) δ/ppm = 174.4 (CO^{Leu}), 171.1 (CO^{Phe}), 164.3 (CO), 149.6 (C-6^{Pyr}), 146.3 (C-2^{Pyr}), 141.4 (C-4^{Pyr}), 138.1 (C_q^{Ar}), 132.3 (C-5^{Pyr}), 129.7 (2C, C^{Ar}), 128.5 (2C, C^{Ar}), 126.8 (p-C^{Ar}), 123.9 (C-3^{Pyr}), 54.5 (α-CH^{Phe}), 50.8 (α-CH^{Leu}), 40.4 (β-CH_2^{Leu}), 37.9 (β-CH_2^{Phe}), 24.8 (γ-CH^{Leu}), 23.4 (δ-CH_3^{Leu}), 21.8 (δ-CH_3^{Leu}).

IR (ATR): $\tilde{\nu}$ (cm^{-1}) = 3260, 2958, 2931, 1719, 1640, 1577, 1424, 1341, 1143, 753.

9 Synthesis of *N*-(5-fluoro-2,4-dinitrobenzoyl)-L-phenylalanyl-L-leucine

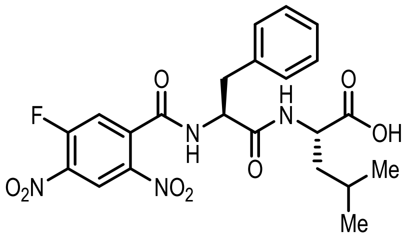

This compound was prepared starting from **27** (103 mg, 0.19 mmol, 1.0 Eq.) following GP4. The compound was obtained after preparative HPLC (HTec-C18, isocratic 50:50 MeCN/H_2O, product peak after 8.50 min) as a colorless solid (58 mg, 0.12 mmol, 63 %).

Rf. = 0.42 (1:9, MeOH/DCM).

MS (ESI): m/z (%) = 491.2 (100) $[M+H]^+$

HRMS (ESI): 491.1568 ($[M+H]^+$, calc. $C_{22}H_{24}FN_4O_8^+$: 491,1573).

Mp. = 196.5–201.0 °C (decomp.)

$[\alpha]_D^{22}$ = -18.8° (c = 1.00 g/mL, MeOH).

^{1}H-NMR, COSY (400 MHz, DMSO-d_6) δ/ppm = 12.62 (s, 1H, OH), 9.22 (d, J = 8.5 Hz, 1H, NH^{Phe}), 8.76 (d, J = 6.5 Hz, 1H, H-3^{ArF}), 8.41 (d, J = 7.9 Hz, 1H, NH^{Leu}), 7.42 (d, J = 10.3 Hz, 1H, H-6^{ArF}), 7.35 – 7.26 (m, 4H,H^{Ar}), 7.22 (ddt, J = 6.7, 5.1, 2.9 Hz, 1H, p-H^{Ar}), 4.86 – 4.72 (m, 1H, α-CH^{Phe}), 4.28 (ddd, J = 9.5, 7.8, 5.4 Hz, 1H, α-CH^{Leu}), 3.13 (dd, J = 14.0, 4.6 Hz, 1H, β-CH_2^{Phe}), 2.87 (dd, J = 14.0, 9.7 Hz, 1H, β-CH_2^{Phe}), 1.75 – 1.63 (m, 1H, γ-CH_2^{Leu}), 1.63 – 1.49 (m, 2H, β-CH^{Leu}), 0.90 (dd, J = 18.8, 6.5 Hz, 6H, δ-CH_3^{Leu}).

^{19}F-NMR (282 MHz, DMSO-d_6) δ/ppm = -109.20 (dd, J = 10.4, 6.6 Hz).

^{13}C-NMR, HSQC, HMBC (101 MHz, DMSO) δ/ppm 173.9 (CO^{Leu}), 170.3 (CO^{Phe}), 162.3 (CO^{ArF}), 156.2 (d, J = 271.3 Hz, C-5^{ArF}), 142.1 (d, J = 3.6 Hz, C-2^{ArF}), 138.5 (d, J = 9.1 Hz, C-1^{ArF}), 137.4 (C-1^{Ar}), 136.7 (d, J = 8.7 Hz, C-4^{ArF}), 129.3 (2C, C^{Ar}), 128.1 (2C, C^{Ar}), 126.4 (C-4^{Ar}), 123.3 (C-3^{ArF}), 119.5 (d, J = 24.6 Hz, C-6^{ArF}), 54.0 (α-CH^{Phe}), 50.4 (α-CH^{Leu}), 39.8 (β-CH^{Leu}), 37.5 (β-CH_2^{Phe}), 24.3 (γ-CH_2^{Leu}), 22.9 (δ-CH_3^{Leu}), 21.4 (δ-CH_3^{Leu}).

IR (ATR): $\tilde{\nu}$ (cm^{-1}) = 3268, 3039, 2962, 1720, 1649, 1543, 1345, 1255, 745, 700.

10 Synthesis of *N*-(2-fluoro-3-nitrobenzoyl)-L-phenylalanyl-L-leucine

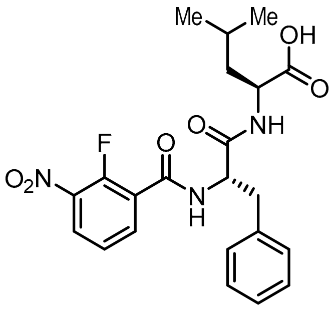

This compound was prepared starting from **28** (300 mg, 0.60 mmol) following GP4. The product was obtained as a colorless solid (252 mg, 0.57 mmol, 95 %).

Rf. = 0.19 (DCM/MeOH, 5:0.3).

MS (ESI): m/z (%) = 446.2 (100) $[M+H]^+$

HRMS (ESI): 446.1719 ($[M+H]^+$, calc. for $C_{22}H_{25}FN_3O_6^+$: 446.1721).

Mp. = 180.8–182.1 °C

$[\alpha]_D^{22}$ = -71.0° (c = 1.00 g/mL, $CHCl_3$/MeCN, 1:1).

^{1}H-NMR, COSY (400 MHz, DMSO) δ/ppm = 12.63 (s, 1H, OH), 8.81 (d, J = 8.4 Hz, 1H, NH^{Phe}), 8.42 (d, J = 7.9 Hz, 1H, NH^{Leu}), 8.20 (ddd, J = 8.5, 7.0, 1.8 Hz, 1H, H-4^{Ar-F}), 7.69 (ddd, J = 7.7, 5.8, 1.8 Hz, 1H, H-6^{Ar-F}), 7.51 – 7.42 (m, 1H, H-5^{Ar-F}), 7.38 – 7.24 (m, 4H, o-H^{Ar} and m-H^{Ar}), 7.24 – 7.15 (m, 1H, p-H^{Ar}), 4.78 (ddd, J = 10.3, 8.5, 4.0 Hz, 1H, α-CH^{Phe}), 4.28 (ddd, J = 9.4, 7.9, 5.6 Hz, 1H, α-CH^{Leu}), 3.13 (dd, J = 13.9, 4.0 Hz, 1H, β-CH_2^{Phe}), 2.86 (dd, J = 13.9, 10.4 Hz, 1H, , β-CH_2^{Phe}), 1.77 – 1.62 (m, 1H, γ-CH^{Leu}), 1.57 (dt, J = 8.5, 5.5 Hz, 2H, β-CH_2^{Leu}), 0.90 (dd, J = 20.6, 6.5 Hz, 6H, δ-CH_3^{Leu}).

^{19}F-NMR (377 MHz, DMSO) δ/ppm = -122.47 (t, J = 6.5 Hz).

^{13}C-NMR, HSQC, HMBC (101 MHz, DMSO) δ/ppm = 173.9 (CO^{Leu}), 170.7 (CO^{Phe}), 161.9 (CO), 151.59 (d, J = 266.3 Hz, C-2^{Ar-F}), 137.6 (C_q^{Ar}), 137.4 (d, J = 8.2 Hz, C-3^{Ar-F}), 135.2 (d, J = 4.0 Hz, C-6^{Ar-F}), 129.2 (2C, C^{Ar}), 128.0 (2C, C^{Ar}), 127.6 (C-4^{Ar-F}), 126.7 (C-1^{Ar-F}), 126.3 (p-C^{Ar}), 124.8 (d, J = 4.7 Hz, C-5^{Ar-F}), 54.4 (α-CH^{Phe}), 50.3 (α-CH^{Leu}), 39.6 (β-CH_2^{Leu}), 37.3 (β-CH_2^{Phe}), 24.2 (γ-CH^{Leu}), 22.8 (δ-CH_3^{Leu}), 21.3 (δ-CH_3^{Leu}).

IR (ATR): ṽ (cm^{-1}) = 3284, 2961, 1718, 1644, 1614, 1537, 1455, 1352, 744, 697.

11 Synthesis of *N*-(2-fluoro-5-nitrobenzoyl)-L-phenylalanyl-L-leucine

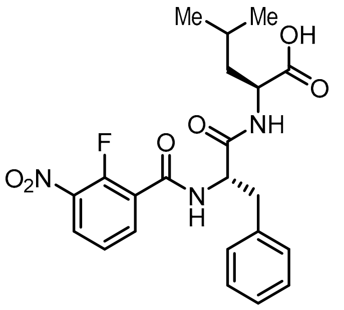

This compound was prepared starting from **29** (116 mg, 0.23 mmol) following GP4. The product was obtained as a colorless solid (85 mg, 0.19 mmol, 83 %).

Rf. = 0.19 (DCM/MeOH, 5:0.3).

MS (ESI): m/z (%) = 446.2 (100) $[M+H]^+$

HRMS (ESI): 444.1557 ($[M-H]^-$, calc. for $C_{22}H_{23}FN_3O_6{}^-$: 444,1576).

Mp. = 147.6–148.9 °C

$[\alpha]_D^{22}$ = -8.1° (c = 1.00 g/mL, CH_2Cl_2).

^{1}H-NMR, COSY (400 MHz, DMSO) δ/ppm = 12.64 (s, 1H, OH), 8.78 (dd, J = 8.4, 1.6 Hz, 1H, NH^{Leu}), 8.43 (d, J = 7.9 Hz, 1H, NH^{Phe}), 8.38 (ddd, J = 9.1, 4.2, 3.0 Hz, 1H, H-$6^{Ar\text{-}F}$), 8.21 (dd, J = 5.9, 3.0 Hz, 1H, H-$4^{Ar\text{-}F}$), 7.56 (t, J = 9.2 Hz, 1H, H-$3^{Ar\text{-}F}$), 7.37 – 7.30 (m, 2H, o-H^{Ar}), 7.31 – 7.24 (m, 2H, m-H^{Ar}), 7.24 – 7.17 (m, 1H, p-H^{Ar}), 4.77 (ddd, J = 10.3, 8.4, 4.0 Hz, 1H, α-CH^{Phe}), 4.28 (ddd, J = 9.5, 7.9, 5.4 Hz, 1H, α-CH^{Phe}), 3.16 (dd, J = 13.8, 4.0 Hz, 1H, β-$CH_2{}^{Phe}$), 2.88 (dd, J = 13.8, 10.3 Hz, 1H, β-$CH_2{}^{Phe}$), 1.79 – 1.63 (m, 1H, γ-CH^{Leu}), 1.63 – 1.49 (m, 2H, β-$CH_2{}^{Leu}$), 0.90 (dd, J = 21.0, 6.5 Hz, 6H, δ-$CH_3{}^{Leu}$).

^{19}F-NMR (377 MHz, DMSO) δ/ppm = -104.03 (dt, J = 9.9, 5.2 Hz).

^{13}C-NMR, HSQC, HMBC (101 MHz, DMSO) δ/ppm = 173.9 (CO^{Leu}), 170.6 (CO^{Phe}), 161.5 (CO), 161.5 (d, J = 262.0 Hz, C-$2^{Ar\text{-}F}$), 143.5 (C-5), 137.6 ($C_q{}^{Ar}$), 129.2 (2C, o-C^{Ar}), 128.0 (3C, m-C^{Ar} and C-6), 126.3 (p-H^{Ar}), 125.6 (d, J = 5.5 Hz, C-$4^{Ar\text{-}F}$), 124.7 (d, J = 17.0 Hz, C-$1^{Ar\text{-}F}$), 118.0 (d, J = 25.1 Hz, C-$3^{Ar\text{-}F}$), 54.4 (α-CH^{Phe}), 50.3 (α-CH^{Leu}), 39.8 (β-$CH_2{}^{Leu}$) 37.2 (β-$CH_2{}^{Phe}$), 24.2 (γ-CH^{Leu}), 22.8 (δ-$CH_3{}^{Leu}$), 21.3 (δ-$CH_3{}^{Leu}$).

IR (ATR): ṽ (cm^{-1}) = 3300, 3080, 2957, 1716, 1629, 1529, 1349, 1195, 742, 448.

12 Synthesis of *N*-(5-chloro-2,4-dinitrobenzoyl)-L-phenylalanyl-L-leucine

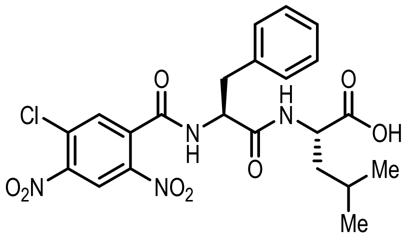

This compound was prepared starting from **30** (80 mg, 0.14 mmol, 1.0 Eq.) following GP4. The compound was obtained after preparative HPLC (HTec-C18, isocratic 55:45 MeCN/H_2O, product peak after 7.32 min) as a colorless solid (69 mg, 0.136 mmol, 96 %).

Rf. = 0.19 (1:9, MeOH/DCM).

MS (ESI): m/z (%) = 507.2 (100) $[M+H]^+$

HRMS (ESI): 507.1279 ($[M+H]^+$, calc. $C_{22}H_{24}ClN_4O_8^+$: 507.1277).

Mp. = 198.4–201.4 °C (decomp.)

$[\alpha]_D^{22}$ = -9.9° (c = 1.00 g/mL, MeOH).

^{1}H-NMR, COSY (600 MHz, DMSO-d_6) δ/ppm = 12.65 (s, 1H, OH), 9.20 (d, J = 8.6 Hz, 1H, NH^{Phe}), 8.79 (s, 1H, H-3^{ArF}), 8.44 (d, J = 7.9 Hz, 1H, NH^{Leu}), 7.55 (s, 1H, H-6^{ArF}), 7.39 – 7.27 (m, 4H, o,m-H^{Ar}), 7.23 (ddt, J = 8.5, 5.9, 2.1 Hz, 1H, p-H^{Ar}), 4.80 (ddd, J = 9.9, 8.6, 4.6 Hz, 1H, α-CH^{Phe}), 4.28 (ddd, J = 10.0, 7.9, 5.1 Hz, 1H, α-CH^{Leu}), 3.14 (dd, J = 14.0, 4.6 Hz, 1H, β-CH_2^{Phe}), 2.86 (dd, J = 14.0, 9.8 Hz, 1H, β-CH_2^{Phe}), 1.69 (tdd, J = 13.2, 9.3, 6.0 Hz, 1H, γ-CH_2^{Leu}), 1.64 – 1.49 (m, 2H, β-CH^{Leu}), 0.90 (dd, J = 28.9, 6.5 Hz, 6H, δ-CH_3^{Leu}).

^{13}C-NMR, HSQC, HMBC (151 MHz, DMSO) δ/ppm = 173.9 (CO^{Leu}), 170.4 (CO^{Phe}), 162.3 (CO^{ArF}), 147.2 (C-5^{ArF}), 145.0 (C-4^{ArF}), 137.5 (C-2^{ArF}), 135.7 (C_{ipso}^{Ar}), 132.3 (C-6^{ArF}), 130.2 (C-1^{ArF}), 129.3 (o-C^{Ar}), 128.1 (m-C^{Ar}), 126.5 (p-C^{Ar}), 122.3 (C-3^{ArF}), 54.0 (α-CH^{Phe}), 50.4 (α-CH^{Leu}), 40.1 (β-CH_2^{Leu}), 37.5 (β-CH_2^{Phe}), 24.3 (γ-CH_2^{Leu}), 22.9 (δ-CH_3^{Leu}), 21.4 (δ-CH_3^{Leu}).

IR (ATR): ṽ (cm^{-1}) = 3257, 2960, 1719, 1647, 1584, 1545, 1344, 1244, 913, 833.

Final compounds, strategy B

13 Synthesis of (*S*)-2-((*S*)-2-(*N*-(5-fluoro-2,4-dinitrophenyl)amino)propanamido)-4-phenylbutanoic acid

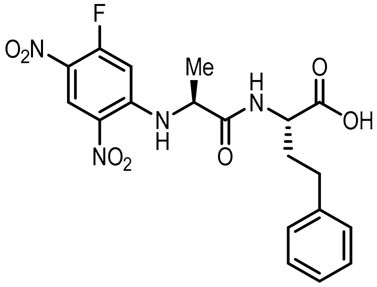

This compound was prepared starting from Fmoc-D-hPhe-OH (250 mg, 0.6 mmol) following GP5. The product was obtained after preparative HPLC (ACE-C18-PFP, isocratic 45:55 MeCN/H_2O, product peak after 12.80 min) as a yellow solid (24 mg, 0.06 mmol, 10 %).

Rf. = 0.32 (DCM/MeOH, 2:0.2).

MS (ESI): m/z (%) = 435.1 (100) $[M+H]^+$

HRMS (ESI): 433.1151 ($[M-H]^-$, calc. for $C_{19}H_{18}FN_4O_7^-$: 433.1165).

Mp. = 182.6 °C (decomp.)

$[\alpha]_D^{22}$ = +3.3° (c = 1.00 g/mL, CH_2Cl_2).

^{1}H-NMR, COSY (600 MHz, DMSO-d_6) δ/ppm = 12.79 (s, 1H, OH), 9.01 (d, J = 6.8 Hz, 1H, NH^{Ala}), 8.91 (d, J = 8.0 Hz, 1H, H-3^{Ar-F}), 8.67 (d, J = 7.9 Hz, 1H, NH^{hPhe}), 7.27 (dd, J = 8.6, 6.7 Hz, 2H, H^{Ar}), 7.20 – 7.10 (m, 3H, H^{Ar}), 6.96 (d, J = 14.1 Hz, 1H, H-6^{Ar-F}), 4.53 (t, J = 6.8 Hz, 1H, α-CH^{Ala}), 4.21 (ddd, J = 9.7, 7.8, 4.3 Hz, 1H, α-CH^{hPhe}), 2.65 (ddd, J = 14.5, 9.6, 5.3 Hz, 1H, γ-CH^{hPhe}), 2.59 (ddd, J = 13.7, 9.2, 7.0 Hz, 1H, γ-CH^{hPhe}), 2.12 – 1.99 (m, 1H, β-CH_2^{hPhe}), 1.99 – 1.87 (m, 1H, β-CH_2^{hPhe}), 1.50 (d, J = 6.8 Hz, 3H, CH_3).

^{19}F-NMR (282 MHz, DMSO-d_6) δ/ppm = -107.27 – -107.88 (m).

^{13}C-NMR, HSQC, HMBC (151 MHz, DMSO) δ/ppm = 173.0 (CO^{hPhe}), 170.7 CO^{Ala}), 159.9 (C-5^{Ar-F}), 158.1 (C-4^{Ar-F}), 147.6 (d, J = 14.2 Hz, C-2^{Ar-F}), 140.8 (C^{Ar}), 128.3 (C^{Ar}), 128.2 (C^{Ar}), 127.4 (C-3^{Ar-F}), 125.9 (C^{Ar}), 125.1 (d, J = 9.5 Hz, C-1^{Ar-F}), 102.1 (d, J = 27.1 Hz, C-6^{Ar-F}), 51.7 (α-CH^{Ala}), 51.5 (α-CH^{hPhe}), 32.4 (β-CH_2^{hPhe}), 31.4 (γ-CH^{hPhe}), 18.2 (CH_3).

IR (ATR): $\tilde{\nu}$ (cm^{-1}) = 3164, 2943, 1719, 1443, 1375, 1472, 1037, 918, 750, 669.

14 Synthesis of ((*R*)-2-((5-fluoro-2,4-dinitrophenyl)amino)-4-phenylbutanoyl)-D-alanine

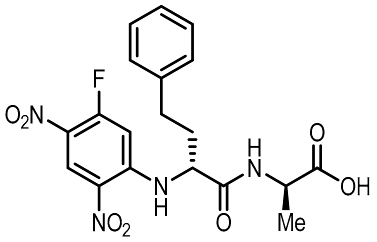

This compound was prepared starting from **35** (82 mg, 0.18 mmol, 1.0 Eq.) GP4. The compound was obtained after preparative HPLC (HTec-C18, isocratic 50:50 MeCN/H_2O, product peak after 11.58 min) as a yellow solid (46 mg, 0.11 mmol, 58 %).

Rf. = 0.22 (DCM).

MS (ESI): m/z (%) = 435.1 (100) $[M+H]^+$

HRMS (ESI): 435.1302 ($[M+H]^+$, calc. for $C_{19}H_{20}FN_4O_7^+$: 435.1311).

Mp. = 137.2–139.4 °C

$[\alpha]_D^{22}$ = +42.2° (c = 1.00 g/mL, $CHCl_3$).

^{1}H-NMR, COSY (600 MHz, DMSO-d_6) δ/ppm = 12.75 (s, 1H, OH), 9.04 (d, J = 7.3 Hz, 1H, NH^{Phe}), 8.88 [a](d, J = 7.9 Hz, 1H, H-3^{ArF}), 8.72 (d, J = 7.2 Hz, 1H, NH^{Ala}), 7.29 – 7.11 (m, 5H, H^{Ar}), 6.97 (d, J = 14.1 Hz, 1H, H-6^{ArF}), 4.51 (q, J = 6.0 Hz, 1H, α-CH^{hPhe}), 4.32 (p, J = 7.3 Hz, 1H, α-CH^{Ala}), 2.73 – 2.56 (m, 2H, γ-CH_2^{hPhe}), 2.18 (td, J = 8.1, 5.5 Hz, 2H, β-CH_2^{hPhe}), 1.33 (d, J = 7.3 Hz, 3H, β-CH_3^{Ala}).

^{19}F-NMR (282 MHz, Acetone-d_6) δ/ppm = -108.02 (dd, J = 14.0, 8.1 Hz).

^{13}C-NMR, HSQC, HMBC (151 MHz, DMSO) δ/ppm = 173.8 (CO^{Ala}), 169.3 (CO^{hPhe}), 159.1 (d, J = 267.0 Hz, C-5^{ArF}), 147.8 (C-4^{ArF}), 141.1 (C-1^{hPhe}), 128.3 (C^{Ar} and C-1^{ArF}), 127.5 (d, J = 15.6 Hz, C-2^{ArF}), 125.9 (p-C^{hPhe}), 125.2 (C-3^{ArF}), 102.30 (d, J = 26.5 Hz, C-6^{ArF}), 55.7 (α-CH^{hPhe}), 47.9 (α-CH^{Ala}), 33.5 (β-CH_2^{hPhe}), 30.1 (γ-CH_2^{hPhe}), 16.9 (β-CH_3^{Ala}).

IR (ATR): ṽ (cm^{-1}) = 3337, 1726, 1630, 1581, 1520, 1364, 1330, 1053, 741, 701.

15 Synthesis of (*R*)-2-((*R*)-2-(*N*-(5-fluoro-2,4-dinitrophenyl)amino)propanamido)-4-phenylbutanoic acid

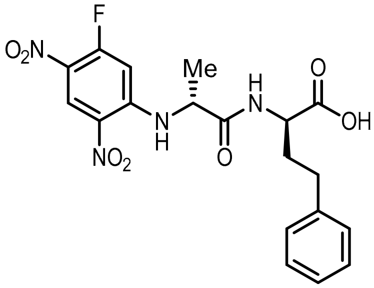

To a 50 mL round bottom flask **36** (450 mg, 1.02 mmol, 1.0 Eq.) was added and dissolved in 10 mL THF. Pd/C (10 mg) was added, and the resulting mixture was stirred for 2 h under a hydrogen atmosphere. The reaction mixture was filtered over a pad of celite, and the solvent removed under reduced pressure. The resulting oil was stirred in 5 mL 3 M HCl in dioxane for 1 h. Dry cyclohexane was added, and the resulting precipitate filtered and dried under reduced pressure. The resulting hydrochloride (292 mg, 1.01 mmol, 99 %) was used without further purification.
To a solution of the hydrochloride (100 mg, 0.35 mmol, 1.0 Eq.) in *tert*-butanol was first 1,5-difluoro-2,4-dinitrobenzene (71 mg, 0.35 mmol, 1.0 Eq.) and then triethylamine (0.15 mL, 1.05 mmol, 3.0 Eq.) added and the mixture turned yellow. The mixture was stirred over night at room temperature. Diluted HCl was added, and the resulting mixture was extracted three times with EtOAc (15 mL). The solvent was removed under reduced pressure and the crude product was purified using preparative HPLC (ACEC18-PFP, isocratic 45:55 MeCN/H_2O, eluted after 15 min). The product was obtained as a yellow solid (32 mg, 0.07 mmol, 21 %).

R_f= 0.28 (4:96, MeOH/DCM).

Smb. = 189.5 °C (decomp.)

MS (ESI): m/z (%) = 435.1 (45) $[M+H]^+$

HRMS (ESI): 433.1171 ($[M-H]^-$, calc. $C_{19}H_{18}FN_4O_7^-$: 433.1165).

$[\alpha]_D^{23}$ = -56.5° (*c* = 1.00 g/mL, MeOH).

^{1}H-NMR, COSY (400 MHz, DMSO) δ/ppm = 12.79 (s, 1H, COOH), 8.94 (dd, *J* = 6.6, 1.6 Hz, 1H, NH^{Ala}), 8.90 (d, *J* = 8.1 Hz, 1H, H-3^{ArF}), 8.75 (d, *J* = 8.0 Hz, 1H, NH^{hPhe}), 7.33 – 7.23 (m, 2H, H^{Ar}), 7.23 – 7.13 (m, 3H, H^{Ar}), 6.93 (d, *J* = 14.2 Hz, 1H, H-6^{ArF}), 4.49 (p, *J* = 6.7 Hz, 1H, α-CH^{Ala}), 4.18 (ddd, *J* = 10.0, 7.9, 4.3 Hz, 1H, α-CH^{hPhe}), 2.71 – 2.52 (m, 2H, γ-CH_2^{hPhe}), 2.13 – 2.01 (m, 1H, β-CH_2^{hPhe}), 1.99 – 1.85 (m, 1H, β-CH_2^{hPhe}), 1.53 (d, *J* = 6.7 Hz, 3H, β-CH_3^{Ala}).

^{19}F-NMR (282 MHz, DMSO) δ/ppm = -106.78 (ddd, *J* = 14.3, 8.2, 1.8 Hz).

^{13}C-NMR, HSQC, HMBC (101 MHz, DMSO) δ/ppm = 173.2 (CO^{hPhe}), 170.9 (CO^{Ala}), 159.0 (d, *J* = 267.3 Hz, C-5^{ArF}), 147.7 (d, *J* = 14.1 Hz, C-4^{ArF}), 140.7 (C-1^{Ar}), 128.4 (2C, C^{Ar}), 128.3 (2C, C^{Ar}), 127.5 (C-3^{ArF}), 127.4 (C-1^{ArF}), 126.0 (*p*-C^{Ar}), 125.3 (d, *J* = 9.5 Hz, C-2^{ArF}), 102.3 (d, *J* = 27.1 Hz, C-6^{ArF}), 52.1 (α-CH^{Ala}), 51.5 (α-CH^{hPhe}), 32.5 (β-CH_2^{hPhe}), 31.4 (, γ-CH_2^{hPhe}), 18.6 (β-CH_3^{Ala}).

IR (ATR): (cm^{-1}) = 3364, 2929, 1721, 1630, 1582, 1521, 1331, 1290, 1125, 700.

16 Synthesis of ((*S*)-2-((5-fluoro-2,4-dinitrophenyl)amino)-4-phenylbutanoyl)-L-alanine

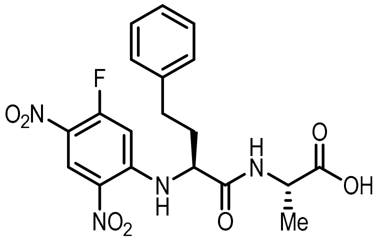

This compound was prepared starting from **37** (57 mg, 0.12 mmol, 1.0 Eq.) GP4. The product was obtained as a yellow solid (41 mg, 0.094 mmol, 81 %).

Rf. = 0.22 (DCM).

MS (ESI): m/z (%) = 435.1 (100) $[M+H]^+$

HRMS (ESI): 433.1158 ($[M-H]^-$, calc. for $C_{19}H_{18}FN_4O_7^{-}$: 433.1165).

Mp. = 137.2–139.4 °C

$[\alpha]_D^{22}$ = -44.5° (c = 1.00 g/mL, $CHCl_3$).

^{1}H-NMR, COSY (600 MHz, Acetone-d_6) δ/ppm = 8.98 (d, J = 8.0 Hz, 1H, H-3^{Ar-F}), 7.20 (d, J = 5.1 Hz, 4H, H^{Ar}), 7.12 (tt, J = 4.7, 3.5 Hz, 1H, p-H^{Ar}), 6.93 (d, J = 14.1 Hz, 1H, H-6 $^{Ar-F}$), 4.68 (t, J = 5.7 Hz, 1H, α-CH^{hPhe}), 4.48 (q, J = 7.3 Hz, 1H, α-CH^{Ala}), 2.79 (qdd, J = 13.7, 10.0, 6.1 Hz, 2H, γ-CH_2^{hPhe}), 2.36 (ddt, J = 14.0, 10.0, 6.0 Hz, 1H, β-CH_2^{hPhe}), 2.29 (ddt, J = 14.0, 10.0, 5.9 Hz, 1H, β-CH_2^{hPhe}), 1.41 (d, J = 7.3 Hz, 3H, β-CH_3^{Ala}).

^{19}F-NMR (282 MHz, Acetone-d_6) δ/ppm = -108.07 (ddd, J = 14.3, 8.0, 2.0 Hz).

^{13}C-NMR, HSQC, HMBC (151 MHz, Acetone-d_6) δ/ppm = 173.7 (CO^{Ala}), 168.2 (CO^{hPhe}), 158.7 (d, J = 267.5 Hz, C-4^{Ar-F}), 147.1 (d, J = 13.8 Hz, C-2^{Ar-F}), 140.1 (C_q^{Ar}), 127.4 (C^{Ar}), 127.3 (C^{Ar}), 126.9 (C-1^{Ar-F}), 126.4 (C-3^{Ar-F}), 124.9 (p-C^{Ar}), 124.6 (d, J = 9.7 Hz, C-5^{Ar-F}), 101.2 (d, J = 27.4 Hz, C-6^{Ar-F}), 55.3 (α-CH^{hPhe}), 47.9 (α-CH^{Ala}), 33.1 (β-CH_2^{hPhe}), 29.7 (γ-CH_2^{hPhe}), 16.3 (β-CH_3^{Ala}).

IR (ATR): ṽ (cm^{-1}) = 3368, 3334, 3115, 1632, 1577, 1288, 1193, 1193, 1164, 1052.

17 Synthesis of (*S*)-2-((*S*)-2-((5-fluoro-2,4-dinitrophenyl)amino)-3-phenylpropanamido)-4-phenylbutanoic acid

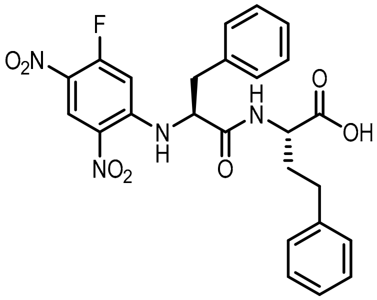

This compound was prepared starting from Fmoc-D-hPhe-OH (250 mg, 0.6 mmol) following GP5. The product was obtained after preparative HPLC (ACE-C18-PFP, isocratic 52:48 MeCN/H_2O, product peak after 7.23 min) as a yellow solid (27 mg, 0.05 mmol, 9 %).

Rf. = 0.39 (DCM/MeOH, 2:0.2).

MS (ESI): m/z (%) = 511.2 (100) $[M+H]^+$

HRMS (ESI): 509.1465 ($[M-H]^-$, calc. for $C_{25}H_{22}FN_4O_7^-$: 509.1478).

Mp. = 80.1 °C (decomp.)

$[\alpha]_D^{22}$ = +15.2° (c = 1.00 g/mL, CH_2Cl_2).

^{1}H-NMR, COSY (600 MHz, DMSO) δ/ppm = 12.84 (s, 1H, OH), 8.85 (d, J = 8.0 Hz, 1H, H-3^{Ar-F}), 8.81 (d, J = 7.6 Hz, 1H, NH^{Phe}), 8.73 (d, J = 7.9 Hz, 1H, NH^{hPhe}), 7.35 – 7.24 (m, 6H, H^{Ar}), 7.23 – 7.15 (m, 4H, H^{Ar}), 6.78 (d, J = 14.3 Hz, 1H, H-6^{Ar-F}), 4.80 (td, J = 7.7, 4.9 Hz, 1H, α-CH^{Phe}), 4.22 (ddd, J = 9.7, 7.9, 4.4 Hz, 1H, α-CH^{hPhe}), 3.32 (dd, J = 14.0, 4.9 Hz, 1H, β-CH_2^{Phe}), 3.17 (dd, J = 13.9, 7.8 Hz, 1H, β-CH_2^{Phe}), 2.69 – 2.55 (m, 2H, γ-CH^{hPhe}), 2.06 (dddd, J = 13.8, 9.6, 7.1, 4.4 Hz, 1H, β-CH_2^{hPhe}), 1.94 (dtd, J = 13.6, 9.5, 5.4 Hz, 1H, β-CH_2^{hPhe}).

^{19}F-NMR (282 MHz, DMSO) δ/ppm = -107.50 (m).

^{13}C-NMR, HSQC, HMBC (151 MHz, DMSO) δ/ppm = 173.0 (CO^{hPhe}), 169.2 (CO^{Phe}), 159.6 (C-5^{Ar-F}), 157.9 (C-4^{Ar-F}), 148.1 (C^{Ar}), 148.0 (d, J = 14.1 Hz, C-2^{Ar-F}), 136.0 (C^{Ar}), 129.5 (C^{Ar}), 128.4 (C^{Ar}), 128.3 (C^{Ar}), 128.3 (C^{Ar}), 128.2 (C-3^{Ar-F}), 127.2 (C^{Ar}), 126.9 (C-2^{Ar-F}), 125.9 (C^{Ar}), 125.2 (d, J = 9.8 Hz, C-1^{Ar-F}), 102.3 (d, J = 27.4 Hz, C-6^{Ar-F}), 57.4 (α-CH^{Phe}), 51.5 (α-CH^{hPhe}), 37.7 (β-CH_2^{Phe}), 32.5 (β-CH_2^{hPhe}), 31.3 (γ-CH^{hPhe}).

IR (ATR): ṽ (cm^{-1}) = 3334, 2929, 1725, 1630, 1583, 1519, 1330, 1119, 1051, 699.

18 Synthesis of ((*R*)-2-((5-fluoro-2,4-dinitrophenyl)amino)-4-phenylbutanoyl)-D-phenylalanine

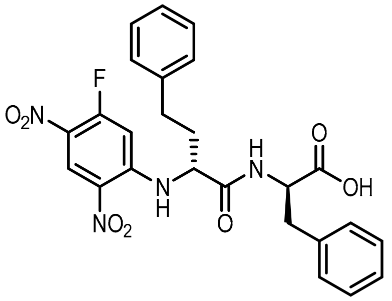

This compound was prepared starting from **38** (100 mg, 0.18 mmol, 1.0 Eq.) GP4. The compound was obtained after preparative HPLC (HTec-C18, isocratic 60:40 MeCN/H_2O, product peak after 9.46 min) as a yellow solid (77 mg, 0.15 mmol, 83 %).

Rf. = 0.15 (98:2, DCM/MeOH).

MS (ESI): m/z (%) = 511.2 (100) [M+H$^+$]

HRMS (ESI): 509.1489 ([M-H]$^-$, calc. for $C_{25}H_{22}FN_4O_7^-$: 509.1478).

Mp. = 87.2–90.9 °C

[α]$_D^{22}$ = +13.6° (c = 1.00 g/mL, MeOH).

^{1}H-NMR, COSY (600 MHz, DMSO-d$_6$) δ/ppm = 12.99 (s, 1H, OH), 8.86 (dd, J = 8.1, 6.0 Hz, 2H, H-6 and NH^{hPhe}), 8.72 (d, J = 8.1 Hz, 1H, NH^{Phe}), 7.28 – 7.10 (m, 10H), 6.89 (d, J = 14.2 Hz, 1H, H-3), 4.57 (ddd, J = 10.2, 8.1, 4.5 Hz, 1H, α-CH^{Phe}), 4.43 (dt, J = 7.7, 5.9 Hz, 1H, α-CH^{hPhe}), 3.15 (dd, J = 14.1, 4.5 Hz, 1H, β-CH$_2^{Phe}$), 2.93 (dd, J = 14.1, 10.1 Hz, 1H, β-CH$_2^{Phe}$), 2.57 (dddd, J = 33.4, 13.8, 9.0, 6.9 Hz, 2H, γ-CH$_2^{hPhe}$), 2.12 (dtd, J = 9.8, 6.3, 3.2 Hz, 2H, β-CH$_2^{hPhe}$).

^{19}F-NMR (282 MHz, DMSO-d$_6$) δ/ppm = -103.17 – -109.17 (m).

^{13}C-NMR, HSQC, HMBC (151 MHz, DMSO) δ/ppm = 172.7 (CO^{Phe}), 169.5 (CO^{hPhe}), 159.0 (d, J = 267.1 Hz, C-5^{ArF}), 147.8 (d, J = 13.8 Hz, C-4^{ArF}), 140.9 (C-1^{hPhe}), 137.5 (C-1^{Phe}), 129.0 (C^{Ar}), 128.3 (C^{Ar}), 128.1 (C^{Ar}), 127.4 (d, J = 23.9 Hz, C-3^{ArF}), 126.4 (C-1^{ArF}), 126.0 (C^{Ar}), 125.3 (d, J = 9.5 Hz, C-2^{ArF}), 102.3 (d, J = 27.3 Hz, C-6^{ArF}), 55.8 (α-CH^{hPhe}), 53.5 (α-CH^{Phe}), 36.3 (β-CH$_2^{Phe}$), 33.4 (β-CH$_2^{hPhe}$), 30.2 (γ-CH$_2^{hPhe}$).

IR (ATR): ṽ (cm^{-1}) = 3349, 1730, 1630, 1583, 1519, 1421, 1364, 1289, 1052, 699.

Precursors, strategy A

19 Synthesis of *tert*-butyl-*N*-(2-nitrophenyl)-L-phenylalanyl-L-leucinate

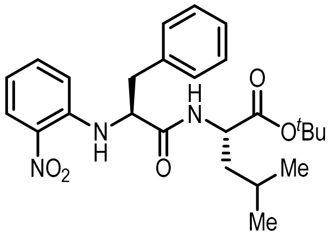

2-Fluoronitrobenzene (77 mg, 0.53 mmol, 1.0 Eq.) and **31** (213 mg, 0.64 mmol, 1.2 Eq.) were dissolved in 0.8 mL DMSO and K_2CO_3 (221 mg, 1.6 mmol, 3.0 Eq.) was added. The suspension was stirred for 12 h at 100 °C. 10 mL water were added and the precipitate was filtered. The product was obtained as a colorless oil following flash-chromatography (235 mg, 0.52 mmol, 98 %).

Rf. = 0.18 (^{c}Hex/EtOAc, 7:1).

MS (ESI): m/z (%) = 400.2 (100) [M+H (free acid)]$^+$, 478.2 (100) [M+Na]$^+$

HRMS (ESI): 478.2305 ([M+H]$^+$, calc. for $C_{26}H_{35}N_4O_6^+$: 478.2312).

$[\alpha]_D^{22}$ = -0.65° (c = 1.00 g/mL, $CHCl_3$).

^{1}H-NMR, COSY (400 MHz, $CDCl_3$) δ/ppm = 8.19 – 8.13 (m, 2H, NH^{Phe} and H-3), 7.43 (ddd, J = 8.6, 7.0, 1.6 Hz, 1H, H-5), 7.37 – 7.27 (m, 5H, H^{Ar}), 6.80 – 6.70 (m, 2H, H-4 and H-6), 6.55 (d, J = 8.5 Hz, 1H, NH^{Leu}), 4.49 (td, J = 8.7, 5.2 Hz, 1H, α-CH^{Leu}), 4.19 (dt, J = 8.1, 4.1 Hz, 1H, α-CH^{Phe}), 3.35 (dd, J = 14.0, 4.4 Hz, 1H, β-CH_2^{Phe}), 3.19 (dd, J = 14.0, 8.0 Hz, 1H, β-CH_2^{Phe}), 1.55 – 1.35 (m, 3H, , β-CH_2^{Leu} and γ-CH^{Leu}), 1.31 (s, 9H, CH_3), 0.92 (dd, J = 6.2, 3.5 Hz, 6H, δ-CH_3^{Leu}).

^{13}C-NMR, HSQC, HMBC (101 MHz, $CDCl_3$) δ/ppm = 171.0 (CO^{Leu}), 170.9 (CO^{Phe}), 143.9 (C_q^{Ar}), 136.4 (C-5), 135.7 (C-1), 133.3 (C-2), 129.3 (C^{Ar}), 129.0 (C^{Ar}), 127.5 (C^{Ar}), 126.7 (C-3), 117.5 (C-4), 115.1 (C-6), 81.8 (C_q^{Boc}), 60.4 (α-CH^{Phe}), 51.4 (α-CH^{Leu}), 41.6 (β-CH_2^{Leu}), 39.0 (β-CH_2^{Phe}), 27.8 (3C, CH_3), 24.8 (γ-CH^{Leu}), 22.7 (δ-CH_3^{Leu}), 22.0 (δ-CH_3^{Leu}).

IR (ATR): $\tilde{\nu}$ (cm^{-1}) = 3165, 2253, 1442, 1375, 1038, 918, 874, 541, 520, 453.

20 Synthesis of *tert*-butyl-*N*-(3,4-dinitrophenyl)-L-phenylalanyl-L-leucinate

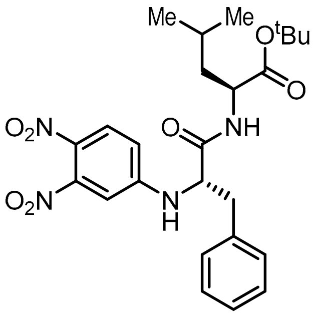

This compound was prepared starting from **31** (662 mg, 1.98 mmol) and 3,4-dinitrofluorobenzene (368 mg, 1.98 mmol) following GP3. The product was obtained as an intensively yellow solid (241 mg, 0.48 mmol, 24 %).

Rf. = 0.17 (^{c}Hex/EtOAc, 8:1).

MS (ESI): m/z (%) = 445.2 (100) $[M+H \text{ (free acid)}]^+$, 523.2 (23.1) $[M+Na]^+$

HRMS (ESI): 523.2159 ($[M+H]^+$, calc. for $C_{25}H_{32}N_4NaO_7^+$: 523.2163).

Mp. = 77.4–78.6 °C

$[\alpha]_D^{22}$ = -7.2° (c = 1.00 g/mL, $CHCl_3$).

¹H-NMR, COSY (600 MHz, $CDCl_3$) δ/ppm = 7.87 (d, J = 9.0 Hz, 1H, H-5), 7.31 – 7.22 (m, 3H, H^{Ar}), 7.20 – 7.15 (m, 2H, H^{Ar}), 6.64 – 6.57 (m, 3H, H-2, H-6 and NH^{Leuz}), 5.86 (d, J = 6.7 Hz, 1H, NH^{Phe}), 4.49 (td, J = 8.7, 5.0 Hz, 1H, α-CH^{Leu}), 4.24 (td, J = 7.3, 5.6 Hz, 1H, α-CH^{Phe}), 3.22 (td, J = 15.4, 5.1 Hz, 1H, β-CH_2^{Phe}), 3.09 (dd, J = 14.1, 7.6 Hz, 1H, β-CH_2^{Phe}), 1.63 – 1.53 (m, 2H, β-CH_2^{Leu} and γ-CH^{Leu}), 1.48 (dd, J = 9.1, 7.8 Hz, 1H, β-CH_2^{Leu}), 1.45 (s, 9H, CH_3^{tBu}), 0.97 – 0.84 (m, 6H, δ-CH_3^{Leu}).

¹³C-NMR, HSQC, HMBC (151 MHz, $CDCl_3$) δ/ppm = 171.6 (CO^{Leu}), 170.3 (CO^{Phe}), 151.7 (C-3), 146.7 (C-4), 135.3 (C^{Ar}), 130.2 (C^{Ar}), 129.6 (C^{Ar}), 129.2 (C^{Ar}), 129.0 (C^{Ar}), 128.7 (C^{Ar}), 128.0 (C^{Ar}), 127.7 (C-5), 113.5 (C-2), 107.6 (C-6), 82.5 (C^{tBu}), 58.7 (α-CH^{Phe}), 51.7 (α-CH^{Leu}), 41.6 (β-CH_2^{Leu}), 39.0 (β-CH_2^{Phe}), 28.0 (CH_3^{tBu}), 25.0 (γ-CH^{Leu}), 22.7 (δ-CH_3^{Leu}), 21.9 (δ-CH_3^{Leu}).

IR (ATR): $\tilde{\nu}$ (cm^{-1}) = 3275, 2959, 1731, 1657, 1606,1368, 1321, 1144, 944, 843.

21 Synthesis of *tert*-butyl-*N*-(5-fluoro-2,4-dinitrophenyl)-L-phenylalanyl-L-leucinate

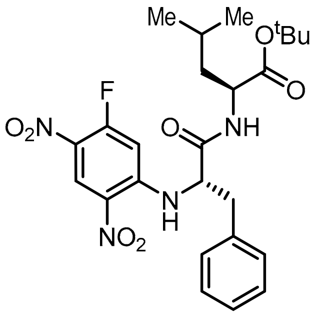

This compound was prepared starting from **31** (250 mg, 0.75 mmol) and 1,3-difluoro-4,6-dinitrobenzene (153 mg, 0.75 mmol) following GP3. DCM was used as a solvent. The product was obtained as an intensively yellow solid (310 mg, 0.6 mmol, 80 %).

Rf. = 0.33 (^{c}Hex/EtOAc, 5:1).

MS (ESI): m/z (%) = 463.1 (100) [M+H (free acid)]$^+$, 541.2 (14.2) [M+Na]$^+$

HRMS (ESI): 541.2077 ([M+H]$^+$, calc. for $C_{25}H_{31}FN_4NaO_7^+$: 541.2069).

Mp. = 162.2–172.2 °C

$[\alpha]_D^{22}$ = +4.5° (c = 1.00 g/mL, $CHCl_3$).

^{1}H-NMR, COSY (400 MHz, $CDCl_3$) δ/ppm = 9.10 (d, J = 7.8 Hz, 1H, H-3$^{Ar\text{-}F}$), 8.85 – 8.77 (m, 1H, NH^{Phe}), 7.40 – 7.24 (m, 5H, H^{Ar}), 6.46 (d, J = 12.9 Hz, 1H, H-6$^{Ar\text{-}F}$), 6.19 (d, J = 8.5 Hz, 1H, NH^{Leu}), 4.50 (td, J = 8.7, 5.0 Hz, 1H, α-CH^{Leu}), 4.24 (dt, J = 7.6, 5.4 Hz, 1H, α-CH^{Phe}), 3.35 (dd, J = 14.1, 5.1 Hz, 1H, β-CH_2^{Phe}), 3.23 (dd, J = 14.1, 7.6 Hz, 1H, β-CH_2^{Phe}), 1.59 – 1.51 (m, 2H, β-CH_2^{Leu} and γ-CH^{Leu}), 1.47 – 1.42 (m, 1H, β-CH_2^{Leu}), 1.41 (s, 9H, CH_3^{tBu}), 0.93 (dd, J = 6.1, 4.4 Hz, 6H, δ-CH_3^{Leu}).

^{19}F-NMR (377 MHz, $CDCl_3$) δ/ppm = -103.9 (dd, J = 12.9, 8.0 Hz).

^{13}C-NMR, HSQC, HMBC (101 MHz, $CDCl_3$) δ/ppm = 171.5 (CO^{Leu}), 168.9 (CO^{Phe}), 161.2 (C-5$^{Ar\text{-}F}$), 158.5 (C-4$^{Ar\text{-}F}$), 148.3 (d, J = 13.4 Hz, C-2$^{Ar\text{-}F}$), 134.7 (C^{Ar}), 129.3 (C^{Ar}), 128.1 (C-1$^{Ar\text{-}F}$), 128.0 (C^{Ar}), 127.6 (C-3$^{Ar\text{-}F}$), 102.70 (d, J = 27.1 Hz, C-6$^{Ar\text{-}Ff}$), 82.5 (C^{tBu}), 60.1 (α-CH^{Phe}), 51.5 (α-CH^{Leu}), 41.5 (β-CH_2^{Leu}), 38.8 (β-CH_2^{Phe}), 27.9 (CH_3^{tBu}), 24.9 (γ-CH^{Leu}), 22.7 (δ-CH_3^{Leu}), 21.9 (δ-CH_3^{Leu}).

IR (ATR): ṽ (cm^{-1}) = 3344, 3278, 2945, 1704, 1630, 1456, 1232, 1150, 967, 921.

22 Synthesis of *tert*-butyl-*N*-(4-nitrophenyl)-L-phenylalanyl-L-leucinate

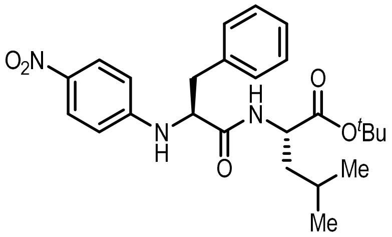

1-Fluoro-4-nitrobenzene (75 mg, 0.53 mmol, 1.0 Eq.) and **31** (213 mg, 0.64 mmol, 1.2 Eq.) were dissolved in 0.8 mL DMSO and K_2CO_3 (221 mg, 1.6 mmol, 3.0 Eq.) was added. The suspension was stirred for 12 h at 100 °C. 10 mL water were added and the precipitate was filtered. The product was obtained as a yellow solid following flash-chromatography (95 mg, 0.31 mmol, 59 %).

Rf. = 0.19 (^{c}Hex/EtOAc, 5:3).

MS (ESI): m/z (%) = 400.2 (100) [M+H (free acid)]$^+$, 478.2 (100) [M+Na]$^+$

HRMS (ESI): 478.2315 ([M+H]$^+$, calc. for $C_{26}H_{35}N_4O_6^+$: 478.2312).

Mp. = 58.3–59.9 °C

$[\alpha]_D^{22}$ = +2.1° (c = 1.00 g/mL, $CHCl_3$).

^{1}H-NMR, COSY (400 MHz, $CDCl_3$) δ/ppm = 8.06 – 8.02 (m, 2H, H-3,5), 7.33 – 7.17 (m, 5H, H^{Ar}), 6.61 – 6.49 (m, 3H, H-2,6 and NH^{Leu}), 4.55 – 4.37 (m, 1H, α-CH^{Leu}), 4.18 (dd, J = 7.5, 5.4 Hz, 1H, α-CH^{Phe}), 3.27 (dd, J = 14.1, 5.4 Hz, 1H, β-CH_2^{Phe}), 3.13 (dd, J = 14.1, 7.4 Hz, 1H, β-CH_2^{Phe}), 1.62 – 1.41 (m, 3H, β-CH_2^{Leu} and γ-CH^{Leu}), 1.38 (s, 9H, CH_3), 0.91 (dd, J = 6.1, 3.6 Hz, 6H, δ-CH_3^{Leu}).

^{13}C-NMR, HSQC, HMBC (101 MHz, $CDCl_3$) δ/ppm = 171.3 (CO^{Leu}), 170.7 (CO^{Phe}), 151.7 (C-1), 139.4 (C-4), 135.7 (C_q^{Ar}), 129.1 (C^{Ar}), 129.0 (C^{Ar}), 127.5 (C^{Ar}), 126.2 (C-3,5), 112.6 (C-2,6), 82.2 (C_q^{Boc}), 59.1 (α-CH^{Phe}), 51.5 (α-CH^{Leu}), 41.7 (β-CH_2^{Leu}), 38.6 (β-CH_2^{Phe}), 27.9 (3C, CH_3), 24.9 (γ-CH^{Leu}), 22.7 (δ-CH_3^{Leu}), 22.0 (δ-CH_3^{Leu}).

IR (ATR): $\tilde{\nu}$ (cm^{-1}) = 3357, 2959, 1730, 1652, 1598, 1308, 1146, 1110, 909, 698.

23 Synthesis of *tert*-butyl-*N*-(5-fluoro-2-nitrophenyl)-L-phenylalanyl-L-leucinate

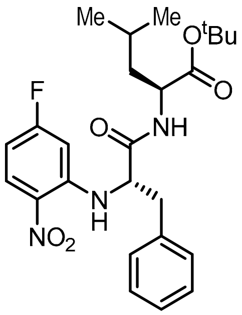

This compound was prepared starting from **31** (250 mg, 0.75 mmol) and 2,4-difluoro-1-nitrobenzene (0.08 mL, 0.75 mmol) following GP3. The product was obtained as an intensively yellow solid (224 mg, 0.47 mmol, 63 %).

Rf. = 0.16 (^{c}Hex/EtOAc, 20:1).

MS (ESI): m/z (%) = 418.2 (100) [M+H (free acid)]$^{+}$, 496.2 (15.4) [M+Na]$^{+}$

HRMS (ESI): 496.2223 ([M+H]$^{+}$, calc. for $C_{25}H_{32}FN_3NaO_5^{+}$: 496.2218).

Mp. = 148.4–149.5 °C

[α]$_D^{22}$ = +15.9° (c = 1.00 g/mL, $CHCl_3$).

^{1}**H-NMR**, COSY (400 MHz, $CDCl_3$) δ/ppm = 8.39 – 8.32 (m, 1H, NH^{Phe}), 8.21 (dd, J = 9.4, 5.9 Hz, 1H, H-3^{Ar-F}), 7.37 – 7.27 (m, 5H, H^{Ar}), 6.51 – 6.41 (m, 2H, NH^{Leu} and H-6^{Ar-F}), 6.38 (dd, J = 10.9, 2.5 Hz, 1H, H-4^{Ar-F}), 4.51 (td, J = 8.7, 5.1 Hz, 1H, α-CH^{Leu}), 4.13 (dt, J = 7.7, 4.8 Hz, 1H, α-CH^{Phe}), 3.34 (dd, J = 14.0, 4.6 Hz, 1H, β-CH_2^{Phe}), 3.20 (dd, J = 14.0, 7.8 Hz, 1H, β-CH_2^{Phe}), 1.59 – 1.48 (m, 2H, β-CH_2^{Leu} and γ-CH^{Leu}), 1.47 – 1.36 (m, 1H, β-CH_2^{Leu}), 1.35 (s, 9H, CH_3^{tBu}), 0.92 (dd, J = 6.1, 4.6 Hz, 6H, δ-CH_3^{Leu}).

^{19}**F-NMR** (377 MHz, $CDCl_3$) δ/ppm = -98.18 (dt, J = 12.1, 6.7 Hz).

^{13}**C-NMR**, HSQC, HMBC (101 MHz, $CDCl_3$) δ/ppm = 171.2 (CO^{Leu}), 170.3 (CO^{Phe}), 167.4 (d, J = 257.9 Hz, C-5^{Ar-F}) 146.1 (d, J = 13.2 Hz, C-1^{Ar-F}), 135.4 (C-2^{Ar-F}), 129.9 (C^{Ar}), 129.8 (C-3^{Ar-F}), 129.3 (C^{Ar}), 129.0 (C^{Ar}), 127.6 (C^{Ar}), 105.8 (d, J = 24.4 Hz, C-4^{Ar-F}), 101.1 (d, J = 27.5 Hz, C-6^{Ar-F}), 82.0 (C^{tBu}), 60.4 (α-CH^{Phe}), 51.4 (α-CH^{Leu}), 41.6 (β-CH_2^{Leu}), 38.8 (β-CH_2^{Phe}), 27.8 (CH_3^{tBu}), 24.8 (γ-CH^{Leu}), 22.7 (δ-CH_3^{Leu}), 22.0 (δ-CH_3^{Leu}).

IR (ATR): ṽ (cm^{-1}) = 3270, 3110, 1733, 1457, 1394, 1230, 1145, 1079, 753, 626.

24 Synthesis of *tert*-butyl-*N*-(6-chloropyrimidin-4-yl)-L-phenylalanyl-L-leucinate

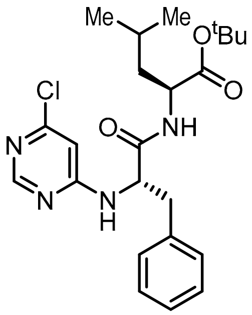

This compound was prepared starting from **31** (250 mg, 0.75 mmol) and 4,6-dichloropyrimidine (74 mg, 0.5 mmol) following GP3. The product was obtained as a colorless solid (204 mg, 0.3 mmol, 60 %).

Rf. = 0.40 (^{c}Hex/EtOAc, 3:1).

MS (ESI): m/z (%) = 447.2(100) $[M+H]^+$

HRMS (ESI): 447.2156 ($[M+H]^+$, calc. for $C_{23}H_{32}ClN_4O_3^+$: 447.2157).

Mp. = 62.8–64.2 °C

$[\alpha]_D^{22}$ = -14.5° (c = 1.00 g/mL, $CHCl_3$).

^{1}H-NMR, COSY (400 MHz, $CDCl_3$) δ/ppm = 8.39 – 8.34 (m, 1H, H-2^{pyr}), 7.34 – 7.19 (m, 5H, H^{Ar}), 6.37 (d, J = 0.9 Hz, 2H, H-5^{pyr} and NH^{Leu}), 6.10 (d, J = 7.2 Hz, 1H, NH^{Phe}), 4.75 (s, 1H, α-CH^{Phe}), 4.46 (td, J = 8.4, 5.0 Hz, 1H, α-CH^{Leu}), 3.17 (d, J = 6.7 Hz, 2H, β-CH_2^{Phe}), 1.64 – 1.49 (m, 2H, β-CH_2^{Leu}), 1.45 (s, 10H, CH_3^{tBu} and γ-CH^{Leu}), 0.90 (d, J = 6.2 Hz, 6H, δ-CH_3^{Leu}).

^{13}C-NMR, HSQC, HMBC (101 MHz, $CDCl_3$) δ/ppm = 171.5 (CO^{Leu}), 170.6 (CO^{Phe}), 162.1 (C-6^{pyr}), 158.1 (C-2^{pyr}), 136.0 (C-4^{pyr}), 129.3 (C^{Ar}), 128.8 (C^{Ar}), 127.3 (C^{Ar}), 104.4 (C-5^{pyr}), 82.2 (C^{tBu}), 56.3 (α-CH^{Phe}), 51.6 (α-CH^{Leu}), 41.9 (β-CH_2^{Leu}), 38.3 (β-CH_2^{Phe}), 28.0 (CH_3^{tBu}), 24.8 (γ-CH^{Leu}), 22.7 (δ-CH_3^{Leu}), 22.1 (δ-CH_3^{Leu}).

IR (ATR): ṽ (cm^{-1}) = 3281, 2959, 1732, 1580, 1496, 1323, 1146, 1091, 981, 698.

25 Synthesis of *tert*-butyl (*N*-(4-fluoro-3-nitrophenyl)carbamoyl)-L-phenylalanyl-L-leucinate

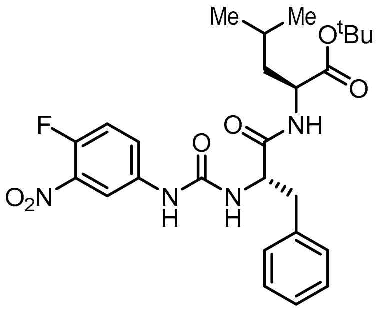

This compound was prepared starting from **31** (250 mg, 0.75 mmol) and 4-fluoro-3-nitrophenylisocyanate (136 mg, 0.75 mmol) following GP3. DCM was used as a solvent. The product was obtained as a colorless solid (297 mg, 0.57 mmol, 77 %).

Rf. = 0.30 (^{c}Hex/EtOAc, 5:2).

MS (ESI): m/z (%) = 461.2(100) [M+H (free acid)]$^+$, 539.3 (4.86) [M+Na]$^+$

HRMS (ESI): 539.2280 ([M+Na]$^+$, calc. for $C_{26}H_{33}FN_4NaO_6^+$: 539.2276).

Mp. = 77.1–82.6 °C

$[\alpha]_D^{22}$ = -7.7° (c = 1.00 g/mL, $CHCl_3$).

^{1}H-NMR, COSY (400 MHz, $CDCl_3$) δ/ppm = 8.03 (s, 1H, NH), 7.94 (dd, J = 6.5, 2.7 Hz, 1H, H-2^{Ar-F}), 7.42 (dq, J = 9.1, 3.1 Hz, 1H, H-5^{Ar-F}), 7.30 – 7.15 (m, 5H, H^{Ar}), 6.99 (dd, J = 10.3, 9.0 Hz, 1H, H-6^{Ar-F}), 6.97 – 6.76 (m, 2H, NH^{Phe} and NH^{Leu}), 4.74 (d, J = 7.6 Hz, 1H, α-CH^{Phe}), 4.33 (td, J = 7.9, 5.4 Hz, 1H, α-CH^{Leu}), 3.08 (dd, J = 13.9, 6.2 Hz, 1H, β-CH_2^{Phe}), 2.90 (dd, J = 13.9, 7.7 Hz, 1H, β-CH_2^{Phe}), 1.62 (dt, J = 13.0, 6.0 Hz, 2H, β-CH_2^{Leu} and γ-CH^{Leu}), 1.54 (td, J = 9.1, 6.9 Hz, 1H, β-CH_2^{Leu}), 1.45 (s, 9H, CH_3^{tBu}), 0.85 (dd, J = 12.9, 5.7 Hz, 6H, δ-CH_3^{Leu}).

^{19}F-NMR (377 MHz, $CDCl_3$) δ/ppm = -125.9.

^{13}C-NMR, HSQC, HMBC (101 MHz, $CDCl_3$) δ/ppm = 173.6 (CO^{Phe}), 171.2 (CO^{Leu}), 155.1 (CO), 150.5 (d, J = 258.9 Hz, C-4^{Ar-F}), 136.78 (d, J = 8.1 Hz, C-3^{Ar-F}), 136.1 (C-1^{Ar}), 135.6 (C-1^{Ar-F}), 129.3 (C-3,5^{Ar-F}), 128.7 (C-2,6^{Ar}), 127.2 (C-4^{Ar}), 125.6 (d, J = 7.6 Hz, C-5^{Ar-F}), 118.1 (d, J = 21.7 Hz, C-6^{Ar-F}), 115.6 (C-2^{Ar-F}), 82.5 (C^{tBu}), 55.2 (α-CH^{Phe}), 52.5 (α-CH^{Leu}), 41.0 (β-CH_2^{Leu}), 39.0 (β-CH_2^{Phe}), 28.0 (CH_3^{tBu}), 24.8 (γ-CH^{Leu}), 22.6 (δ-CH_3^{Leu}), 22.0 (δ-CH_3^{Leu}).

IR (ATR): ṽ (cm^{-1}) = 3307, 2959, 1733, 1639, 1050, 952, 609, 560, 544, 499.

26 Synthesis of *tert*-butyl-*N*-(2,6-dichloronicotinoyl)-L-phenylalanyl-L-leucinate

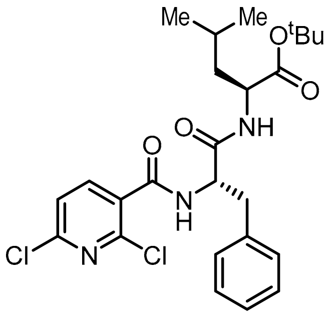

In a 10 mL round bottom flask 2,6-dichloropyridin-3-carbonylchloride (165 mg, 0.78 mmol, 1.0 Eq.) and **31** 262 mg, 0.78 mmol, 1.0 Eq.) were dissolved in 3 mL abs. DCM and cooled to 0 °C. Triethylamine (0.25 mL, 1.79 mmol, 2.3 Eq.) in 3 mL dry DCM was added dropwise and the reaction mixture was stirred for 1 h. The organic phase was washed with 10 mL H_2O and then dried over Na_2SO_4. The solvent was removed under reduced pressure and the crude product was purified using flash-chromatography. The product was isolated as a colorless solid (329 mg, 0.65 mmol, 83 %).

Rf. = 0.53 (^{c}Hex/EtOAc, 5:3).

MS (ESI): m/z (%) = 452.1 (100) [M+H (free acid)]$^+$, 530.2 (19.0) [M+Na]$^+$

HRMS (ESI): 530.1582 ([M+H]$^+$, calc. for $C_{25}H_{31}Cl_2N_3NaO_4^+$: 530.1584).

Mp. = 145.7–150.7 °C

$[\alpha]_D^{22}$ = +2.7° (c = 1.00 g/mL, $CHCl_3$).

^{1}H-NMR, COSY (400 MHz, $CDCl_3$) δ/ppm = 8.87 (dd, J = 6.4, 2.9 Hz, 1H, a4), 8.34 (ddd, J = 9.0, 4.2, 2.9 Hz, 1H, H-5), 7.43 (dd, J = 10.1, 7.4 Hz, 1H, NH^{Phe}), 7.34 – 7.16 (m, 5H, H^{Ar}), 6.36 (d, J = 8.0 Hz, 1H, NH^{Leu}), 4.94 (qd, J = 6.6, 1.7 Hz, 1H, α-CH^{Phe}), 4.44 (td, J = 8.1, 5.2 Hz, 1H, α-CH^{Leu}), 3.21 (d, J = 6.5 Hz, 2H, β-CH_2^{Phe}), 1.63 – 1.55 (m, 2H, β-CH_2^{Leu}), 1.47 (s, 9H, CH_3^{tBu}), 0.90 (ddd, J = 9.1, 6.2, 3.9 Hz, 7H, δ-CH_3^{Leu} and γ-CH^{Leu}).

^{13}C-NMR, HSQC, HMBC (101 MHz, $CDCl_3$) δ/ppm = 171.5 (CO^{Leu}), 169.7 (CO^{Ph}), 164.7 (CO), 160.7 (C-6^{Pyr}), 144.6 (C-2^{Pyr}), 135.9 (C_q^{Ar}), 129.4 (C^{Ar}), 128.7 (C^{Ar}), 128.6 (C^{Ar}), 128.5 (C-$5z^{Pyr}$), 128.0 (C-4^{Pyr}), 127.2 (p-C^{Ar}), 122.2 (C-3^{Pyr}), 117.8, 82.1 (C^{tBu}), 55.2 (α-CH^{Phe}), 51.7 (α-CH^{Leu}), 41.7 (β-CH_2^{Leu}), 38.4 (β-CH_2^{Phe}), 28.2 (CH_3^{tBu}), 28.0 (CH_3^{tBu}), 27.9 (CH_3^{tBu}), 24.9 (γ-CH^{Leu}), 22.6 (δ-CH_3^{Leu}), 22.1 (δ-CH_3^{Leu}).

IR (ATR): ṽ (cm^{-1}) = 3280, 2958, 1736, 1638, 1575, 1542, 1454, 1142, 879, 730.

27 Synthesis of *tert*-butyl-*N*-(5-fluoro-2,4-dinitrobenzoyl)-L-phenylalanyl-L-leucinate

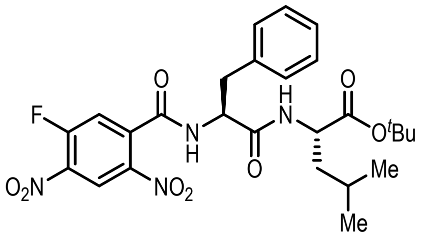

33 (78 mg, 0.31 mmol, 1.0 Eq.) was dissolved in 3 mL toluene and thionylchloride (0.1 mL, 1.4 mmol, 4.6 Eq.) and heated for 3 h at 60 °C. The solvent was removed under reduced pressure and the freshly prepared acid chloride was dissolved in 2 mL DCM and quickly added to a solution of **31** (100 mg, 0.3 mmol, 1.0 Eq.) and triethylamine (0.083 µL,0.6 mmol, 2.0 Eq.) in 3 mL dry DCM at 0 °C. The reaction mixture was stirred for an additional hour and the solvent was removed under reduced pressure. The crude product was purified via flash-column chromatography. The product was obtained as a yellow solid (109 mg, 0.2 mmol, 66 %).

Rf. = 0.74 (2:98, MeOH/DCM).

MS (ESI): m/z (%) = 491.2 (100) $[M+H$ (free acid)$]^+$, 569.3 (30) $[M+Na]^+$

HRMS (ESI): 545.2059 ([M-H], calc. for $C_{26}H_{30}FN_4O_8^-$: 545.2053).

Mp. = 171.5–173.9 °C

$[\alpha]_D^{22}$ = +11.5° (c = 1.00 g/mL, $CHCl_3$).

^{1}H-NMR, COSY (300 MHz, $CDCl_3$) δ/ppm = 8.73 (d, J = 6.3 Hz, 1H, H-3^{ArF}), 7.80 (d, J = 8.3 Hz, 1H, NH^{Phe}), 7.29 – 7.10 (m, 6H, H^{Ar} and H-6^{ArF}), 6.65 (d, J = 7.8 Hz, 1H, NH^{Leu}), 4.96 (q, J = 7.4 Hz, 1H, α-CH^{Phe}), 4.23 (td, J = 7.9, 5.4 Hz, 1H, α-CH^{Leu}), 3.14 (h, J = 7.2 Hz, 2H, β-CH_2^{Phe}), 1.65 – 1.51 (m, 2H, β-CH_2^{Leu} and γ-CH^{Leu}), 1.45 (s, 10H, CH_3^{tBu} and β-CH_2^{Leu}), 0.87 (dd, J = 11.7, 5.9 Hz, 6H, δ-CH_3^{Leu}).

^{19}F-NMR (282 MHz, $CDCl_3$) δ/ppm = -106.02 (dd, J = 9.4, 6.4 Hz).

^{13}C-NMR, HSQC, HMBC (75 MHz, $CDCl_3$) δ/ppm = 171.3 (CO^{Leu}), 170.1 (CO^{Phe}), 163.1 (CO^{ArF}), 157.5 (d, J = 277.4 Hz, C-5^{ArF}), 141.7 (C-2^{ArF}), 139.0 (C-4^{ArF}), 137.0 (C-1^{ArF}), 136.0 (C-1^{Ar}), 129.5 (2C, C^{Ar}), 128.8 (2C, C^{Ar}), 127.4 (C-4^{Ar}), 123.6 (C-3^{ArF}), 119.8 (d, J = 23.9 Hz, C-6^{ArF}), 82.3 (C_q^{tBu}), 54.9 (α-CH^{Phe}), 52.0 (α-CH^{Leu}), 41.3 (β-CH_2^{Leu}), 38.4 (β-CH_2^{Phe}), 28.0 (3C, CH_3^{tBu}), 24.9 (γ-CH^{Leu}), 22.7 (δ-CH_3^{Leu}), 22.1 (δ-CH_3^{Leu}).

IR (ATR): $\tilde{\nu}$ (cm^{-1}) = 3271, 3065, 2961, 1643, 1605, 1542, 1542, 1343, 1149, 834.

28 Synthesis of *tert*-butyl-*N*-(2-fluoro-3-nitrobenzoyl)-L-phenylalanyl-L-leucinate

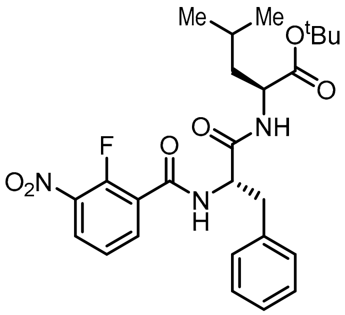

To a 10 mL round bottom flask 2-fluoro-3-nitrobenzoic acid (138 mg, 0.74 mmol, 1.05 Eq.), 4 mL dry toluene and thionylchloride (2.5 mL, 3.4 mmol, 4.6 Eq.) were added. The solution was refluxed for 12 h and afterwards the solvent was evaporated under reduced pressure.

31 (238 mg, 0.71 mmol, 1.0 Eq.) was dissolved in 2 mL DCM and triethylamine (0.13 mL, 0.92 mmol, 1.3 Eq.) was added. The crude acid chloride was dissolved in 2 mL DCM and added dropwise to the previously prepared solution. After the addition was finished, the solution was stirred for 48 h and the solvent was removed under reduced pressure. The crude product was purified using flash-chromatography to yield the product as a slightly yellow solid (309 mg, 0.59 mmol, 83 %).

Rf. = 0.3 (^{c}Hex/EtOAc, 5:2).

MS (ESI): m/z (%) = 446.2 (100) [M+H (free acid)]$^+$, 524.2 (15.4) [M+Na]$^+$

HRMS (ESI): 524.2166 ([M+Na]$^+$, calc. for $C_{26}H_{32}FN_3NaO_6^+$: 524.2167).

Mp. = 78.6–79.2 °C

$[\alpha]_D^{22}$ = -15.0° (c = 1.00 g/mL, $CHCl_3$).

^{1}H-NMR, COSY (400 MHz, $CDCl_3$) δ/ppm = 8.24 (ddd, J = 8.0, 6.3, 1.9 Hz, 1H, H-4^{Ar-F}), 8.14 (ddd, J = 8.1, 7.1, 1.9 Hz, 1H, H-3^{Ar-F}), 7.39 (td, J = 8.0, 1.0 Hz, 1H, H-5^{Ar-F}), 7.33 – 7.24 (m, 6H, H^{Ar} and NH^{Phe}), 6.14 (d, J = 8.0 Hz, 1H, NH^{Leu}), 4.93 – 4.83 (m, 1H, α-CH^{Phe}), 4.43 (td, J = 8.2, 5.4 Hz, 1H, α-CH^{Leu}), 3.27 – 3.13 (m, 2H, β-CH_2^{Phe}), 1.57 (dtd, J = 10.0, 7.5, 5.5 Hz, 2H, β-$CH_2^{Leu\ and}$ γ-CH^{Leu}), 1.46 (s, 10H, β-$CH_2^{Leu\ and}$ CH_3^{tBu}), 0.94 – 0.86 (m, 6H, δ-CH_3^{Leu}).

^{19}F-NMR (377 MHz, $CDCl_3$) δ/ppm = -120.98 (q, J = 7.4 Hz).

^{13}C-NMR (101 MHz, $CDCl_3$) δ/ppm = 171.5 (CO^{Leu}), 169.5 (CO^{Phe}), 161.1 (CO), 153.3 (d, J = 266.1 Hz, C-2^{Ar-F}), 152.0 (C^{Ar}), 137.0 (d, J = 2.9 Hz, C-3^{Ar-F}), 135.9 (C-4^{Ar-F}), 129.4 (C^{Ar}), 129.1 (C-6), 128.8 (C^{Ar}), 127.3 (C^{Ar}), 124.7 (d, J = 4.9 Hz, C-5^{Ar-F}), 124.0 (d, J = 11.7 Hz, C-1^{Ar-F}) 82.1 (C^{tBu}), 55.3 (α-CH^{Phe}), 51.6 (α-CH^{Leu}), 41.8 (β-CH_2^{Leu}), 38.4 (β-CH_2^{Phe}), 28.0 (CH_3^{tBu}), 24.9 (γ-CH^{Leu}), 22.6 (δ-CH_3^{Leu}), 22.2 (δ-CH_3^{Leu}).

IR (ATR): ṽ (cm^{-1}) = 3292, 2960, 1733, 1650, 1537, 1352, 1250, 1150, 742, 547.

29 Synthesis of *tert*-butyl-*N*-(2-fluoro-5-nitrobenzoyl)-L-phenylalanyl-L-leucinate

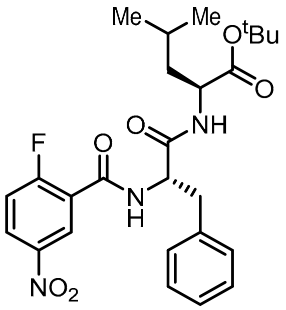

To a 10 mL round bottom flask 2-fluoro-5-nitrobenzoic acid (138 mg, 0.74 mmol, 1.05 Eq.), 4 mL dry toluene and thionylchloride (2.5 mL, 3.4 mmol, 4.6 Eq.) were added. The solution was refluxed for 12 h and afterwards the solvent was evaporated under reduced pressure.
31 (238 mg, 0.71 mmol, 1.0 Eq.) was dissolved in 2 mL DCM and triethylamine (0.13 mL, 0.92 mmol, 1.3 Eq.) was added. The crude acid chloride was dissolved in 2 mL DCM and added dropwise to the previously prepared solution. After the addition was finished the solution was stirred for 48 h and the solvent was removed under reduced pressure. The crude product was purified using flash-chromatography to yield the product as a slightly yellow solid (137 mg, 0.27 mmol, 36 %).

Rf. = 0.6 (^{c}Hex/EtOAc, 5:2).

MS (ESI): m/z (%) = 446.2 (100) [M+H (free acid)]$^+$, 524.2 (19.0) [M+Na]$^+$

HRMS (ESI): 446.1717 ([M+H-tBu]$^+$, calc. for $C_{22}H_{25}FN_3O_6^+$: 446.1721).

Mp. = 68.9–72.3 °C

$[\alpha]_D^{22}$ = -9.5° (c = 1.00 g/mL, $CHCl_3$).

^{1}H-NMR, COSY (400 MHz, $CDCl_3$) δ/ppm = 8.90 (dd, J = 6.4, 2.9 Hz, 1H, H-6$^{Ar\text{-}F}$), 8.35 (ddd, J = 9.0, 4.2, 2.9 Hz, 1H, H-4$^{Ar\text{-}F}$), 7.39 (dd, J = 10.4, 7.3 Hz, 1H, H-3$^{Ar\text{-}F}$), 7.33 – 7.21 (m, 7H, H^{Ar}, NH^{Phe} and H^{Ar}), 6.18 (d, J = 8.0 Hz, 1H,NH^{Leu}), 4.95 – 4.85 (m, 1H, α-CH^{Phe}), 4.43 (td, J = 8.1, 5.4 Hz, 1H, α-CH^{Leu}), 3.28 – 3.14 (m, 2H, β-CH_2^{Phe}), 1.62 – 1.50 (m, 2H, β-$CH_2^{Leu\ and}$ γCH^{Leu}), 1.47 (s, 10H, β-$CH_2^{Leu\ and}$ CH_3^{tBu}), 0.89 (dd, J = 6.2, 3.3 Hz, 6H, δ-CH_3^{Leu}).

^{19}F-NMR (377 MHz, $CDCl_3$) δ/ppm = -103.28 (tt, J = 10.7, 5.1 Hz).

^{13}C-NMR (101 MHz, $CDCl_3$) δ/ppm = 171.4 (CO^{Leu}), 169.6 (CO^{Phe}), 164.7 (CO), 161.36 (d, J = 156.4 Hz, C-2$^{Ar\text{-}F}$), 144.6 (C-5$^{Ar\text{-}F}$), 135.9 (C^{Ar}), 129.4 (C^{Ar}), 128.6 (d, J = 11.1 Hz, C-4$^{Ar\text{-}F}$), 128.5 (C^{Ar}), 128.1 (d, J = 4.3 Hz, C-6$^{Ar\text{-}F}$), 127.3 (C^{Ar}), 122.2 (d, J = 14.2 Hz, C-1$^{Ar\text{-}F}$), 117.7 (d, J = 27.3 Hz, C-3$^{Ar\text{-}F}$), 82.2 (C^{tBu}), 55.2 (α-CH^{Phe}), 51.7 (α-CH^{Leu}), 41.8 (β-CH_2^{Leu}), 38.4 (β-CH_2^{Phe}), 28.0 (CH_3^{tBu}), 24.9 (γ-CH^{Leu}), 22.6 (δ-CH_3^{Leu}), 22.2 (δ-CH_3^{Leu}).

IR (ATR): ṽ (cm^{-1}) = 2969, 2901, 1718, 1653, 1530, 1384, 1242, 1231, 1050, 1026.

30 Synthesis of *tert*-butyl-*N*-(5-chloro-2,4-dinitrobenzoyl)-L-phenylalanyl-L-leucinate

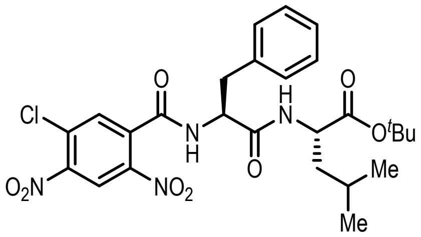

2,4-Dinitro-5-chloro-benzoic acid (78 mg, 0.31 mmol, 1.0 Eq.) was dissolved in 3 mL toluene and thionylchloride (0.1 mL, 1.4 mmol, 4.6 Eq.) and heated for 3 h at 60 °C. The solvent was removed under reduced pressure and the freshly prepared acid chloride was dissolved in 2 mL DCM and quickly added to a solution of **31** (100mg, 0.3 mmol, 1.0 Eq.) and triethylamine (0.083 µL,0.6 mmol, 2.0 Eq.) in 3 mL dry. DCM at 0 °C. The reaction mixture was stirred for an additional hour and the solvent was removed under reduced pressure. The crude product was purified via flash-column chromatography. The product was obtained as a yellow solid (103 mg, 0.18 mmol, 61 %).

Rf. = 0.42 (DCM).

MS (ESI): m/z (%) = 507.1 (100) $[M+H\,(\text{free acid})]^+$, 589.1 (27.0) $[M+Na]^+$

HRMS (APCI): 561.1769 ($[M-H]^-$, calc. $C_{26}H_{30}ClN_4O_8^-$: 561,1757).

$[\alpha]_D^{22}$ = +6.9° (c = 1.00 g/mL, $CHCl_3$).

Mp. = 179.5–181.7 °C

^{1}H-NMR, COSY (300 MHz, $CDCl_3$) δ/ppm = 8.58 (s, 1H, H-3^{ArCl}), 7.43 (s, 1H, H-6^{ArCl}), 7.36 – 7.20 (m, 5H, H^{Ar}), 7.09 (d, J = 7.9 Hz, 1H, NH^{Phe}), 6.26 (d, J = 7.9 Hz, 1H, NH^{Leu}), 4.89 (q, J = 7.2 Hz, 1H, α-CH^{Phe}), 4.34 (td, J = 8.0, 5.2 Hz, 1H, α-CH^{Leu}), 3.29 – 3.14 (m, 2H, β-CH_2^{Phe}), 1.69 – 1.51 (m, 2H, β-CH_2^{Leu} and γ-CH^{Leu}), 1.46 (s, 10H, CH_3^{tBu} and β-CH_2^{Leu}), 0.90 (t, J = 5.7 Hz, 6H, δ-CH_3^{Leu}).

^{13}C-NMR, HSQC, HMBC (75 MHz, $CDCl_3$) δ/ppm = 171.4 (CO^{Leu}), 169.6 (CO^{Phe}), 162.9 (C-4^{ArCl}), 147.6 (C-2^{ArCl}), 144.4 (C-1^{ArCl}), 136.1 (C-1), 133.3 (C-5^{ArCl}), 132.7 (C-6^{ArCl}) , 129.6 (2C, C^{Ar}), 128.9 (2C, C^{Ar}), 127.5 (C-4), 122.4 (C-3^{ArCl}), 82.4 (C_q^{tBu}), 55.0 (α-CH^{Phe}), 51.9 (α-CH^{Leu}), 41.6 (β-CH_2^{Leu}), 38.3 (β-CH_2^{Phe}), 28.1 (3C, CH_3^{tBu}), 24.9 (γ-CH^{Leu}), 22.8 (δ-CH_3^{Leu}), 22.2 (δ-CH_3^{Leu}).

IR (ATR): ṽ (cm^{-1}) = 3271, 2960, 1736, 1642, 1584, 1542, 1343, 1149, 965, 832.

31 Synthesis of *tert*-butyl L-phenylalanyl- L-leucinate

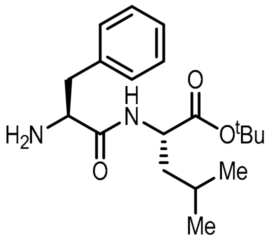

This compound was prepared starting from **32** (3.0 g, 6.4 mmol) following GP2. The product was obtained as a colorless solid (2.19 g, 6.4 mmol, quant.).

Rf. = 0.63 (DCM/MeOH/NEt_3, 9:0.8:0.2).

MS (ESI): m/z (%) = 279.1(100) [M+H (free acid)]$^+$

Mp. = 29.0–31.2 °C

^{1}H-NMR, COSY (300 MHz, $CDCl_3$) δ/ppm = 7.65 (d, J = 8.6 Hz, 1H, NH^{Leu}), 7.37 – 7.17 (m, 5H, H^{Ar}), 4.49 (td, J = 8.6, 5.1 Hz, 1H, α-CH^{Leu}), 3.66 (dd, J = 9.2, 4.0 Hz, 1H, α-CH^{Phe}), 3.24 (dd, J = 13.7, 4.0 Hz, 1H, β-CH_2^{Phe}), 2.73 (dd, J = 13.7, 9.2 Hz, 1H, β-CH_2^{Phe}), 1.79 – 1.49 (m, 3H, γ-CH^{Leu}, β-CH_2^{Leu}), 1.46 (s, 9H, CH_3^{tBu}), 0.99 – 0.86 (m, 6H, δ-CH_3^{Leu}).

^{13}C-NMR, HSQC, HMBC (101 MHz, $CDCl_3$) δ/ppm = 173.9 (CO^{Leu}), 172.4 (CO^{Phe}), 137.8 (C^{Ar}), 129.5 (C^{Ar}), 128.8 (C^{Ar}), 127.0 (C^{Ar}), 81.8 (C^{tBu}), 56.5 (α-CH^{Phe}), 51.1 (α-CH^{Leu}), 41.9 (β-CH_2^{Leu}), 40.9 (β-CH_2^{Phe}), 28.1 (CH_3^{tBu}), 25.1 (γ-CH^{Leu}), 23.0 (δ-CH_3^{Leu}), 22.2 (δ-CH_3^{Leu}).

IR (ATR): $\tilde{\nu}$ (cm^{-1}) = 3323, 2957, 2871, 1732, 1660, 1508, 1367, 1247, 1146, 845, 749.

The spectroscopic data are in accordance to literature [23].

32 Synthesis of *tert*-butyl-*N*-((benzyloxy)carbonyl)-L-phenylalanyl-L-leucinate

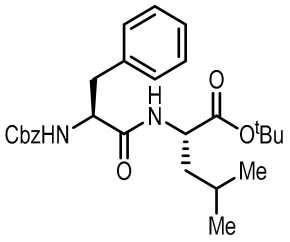

This compound was prepared starting from Z-L-phenylalanine (6.42 g, 21.3 mmol, 1.01 Eq.) and L-leucine-*tert*-butyl ester hydrochloride (4.70 g, 21.0 mmol, 1.0 Eq.) following GP1. The product was obtained as a colorless solid (7.31 g, 15.5 mmol, 74 %).

Rf. = 0.22 (^{c}Hex/EtOAc, 5:1).

MS (ESI): m/z (%) = 413.4 (74) [M+H (free acid)]$^+$, 491.3 (100) [M+Na]$^+$.

Mp. = 88.2–90.4 °C (Lit [24]: 89–91 °C)

^{1}H-NMR, COSY (400 MHz, $CDCl_3$) δ/ppm = 7.43 – 7.16 (m, 10H, H^{Ar}), 6.26 (d, J = 8.2 Hz, 1H, NH^{Leu}), 5.35 (d, J = 8.3 Hz, 1H, NH^{Phe}), 5.10 (s, 2H, CH_2^{Bn}), 4.51 – 4.37 (m, 2H, α-$CH^{Phe,Leu}$), 3.10 (t, J = 5.9 Hz, 2H, β-CH_2^{Phe}), 1.62 – 1.50 (m, 2H, γ-CH^{Leu}), 1.46 (s, 9H CH_3^{tBu}), 0.91 (dd, J = 6.2, 3.9 Hz, 6H, δ-CH_3^{Leu}).

^{13}C-NMR, HMBC, HSQC (101 MHz, CDCl3) δ/ppm =171.6 (CO^{Leu}), 170.3 (CO^{Phe}), 155.9 (COO), 136.3 (C^{Ar}), 136.3 (C^{Ar}), 129.5 (C^{Ar}), 128.7 (C^{Ar}), 128.6 (C^{Ar}), 128.3 (C^{Ar}), 128.1 (C^{Ar}), 127.1 (C^{Ar}), 82.0 (C^{tBu}), 67.1 (CH_2^{Bn}), 56.1 (α-CH^{Phe}), 51.5 (α- CH^{Leu}), 41.9 (β-CH_2^{Leu}), 38.5 (β-CH_2^{Phe}), 28.1 (CH_3^{tBu}), 24.9 (γ-CH^{Leu}), 22.8 (δ-CH_3^{Leu}), 22.2 (δ-CH_3^{Leu}).

IR (ATR): ṽ (cm^{-1}) = 3307, 3065, 2958, 1734, 1696, 1656, 1543, 1257, 1148, 1027.

The spectroscopic data are in accordance to literature [24].

33 Synthesis of 5-fluoro-2,4-dinitrobenzoic acid

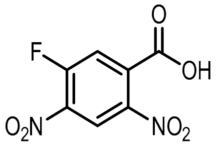

Following a modified procedure of Matsumoto et al. [25].

In a 25 mL round bottom flask **34** (502 mg, 2.5 mmol, 1.0 Eq.) was dissolved in 5 mL concentrated H_2SO_4. The vigorously stirred solution was cooled to 0 °C and slowly CrO_3 (600 mg, 6.0 mmol, 2.4 Eq.) in 1 mL H_2O was added dropwise, so that the temperature would not rise above 5 °C. After the addition was completed, the solution was stirred for 4 h without cooling.
The reaction mixture was poured onto 200 mL of ice water and extracted twice with 30 mL of EtOAc. The organic phase was then extracted with 30 mL of saturated sodium bicarbonate. After addition of 2 N HCl to acidify the solution, it was again extracted twice with 30 mL EtOAc. The solvent was removed under reduced pressure and the product was recrystallized from *n*-hexane/Et_2O (1:1). The product was obtained as a colorless solid (410 mg, 1.78 mmol, 71 %).

Rf. = 0.57 (8:92, MeOH/DCM).

MS (ESI): m/z (%) = 458.9 (100) $[2M-H]^-$

Mp. = 164.3–165.6 °C Lit: 164-166 °C [25]

^{1}H-NMR, COSY (400 MHz, DMSO-d_6) δ/ppm = 14.44 (s, 1H, OH), 8.84 (d, J = 6.5 Hz, 1H, H-3), 8.14 (d, J = 10.6 Hz, 1H, H-6).

^{19}F-NMR (282 MHz, DMSO-d_6) δ/ppm = -108.44 (dd, J = 10.6, 6.5 Hz).

^{13}C-NMR (101 MHz, DMSO-d_6) δ/ppm = 163.9 (CO), 156.6 (d, J = 271.4 Hz, C-5), 142.5 (d, J = 3.9 Hz, C-2), 137.4 (d, J = 8.7 Hz, C-4), 134.9 (d, J = 9.5 Hz, C-1), 123.2 (C-3), 120.1 (d, J = 25.0 Hz, C-6).

IR (ATR): $\tilde{\nu}$ (cm^{-1}) = 3072, 1723, 1630, 1535, 1487, 1342, 1278, 1246, 1230, 834.

The spectroscopic data are in accordance to literature [25].

34 Synthesis of 5-fluoro-2,4-dinitrotoluene

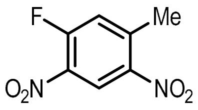

Following a modified procedure of Matsumoto et al. [25].

To a 50 mL flask equipped with concentrated HNO_3 (7.5 mL, 0.169 mol, 6.2 Eq.) concentrated H_2SO_4 (10.8 mL, 0.204 mol, 7.5 Eq.) was slowly added over 5 min, while the temperature was maintained around 2 °C. To this freshly prepared solution 3-fluorotoluene (3.03 mL, 0.0272 mol, 1.0 Eq.) was slowly added in that manner, that the temperature could not rise above 10 °C. After the addition was completed, the suspension was stirred for 30 min at rt, and ice water was added. The mixture was stirred for 20 min and the resulting solid was filtered of and washed with ice water. Recrystallization from EtOH yielded the product as a colorless solid (3.1 g, 15.5 mmol, 57 %).

Rf. = 0.85 (DCM).

GC-MS: m/z (%) = 199.9 $[M]^+$

Mp. = 78.2–78.8 °C Lit: 79-80 °C [25]

^{1}H-NMR, COSY (300 MHz, DMSO-d_6) δ/ppm = 8.76 (d, J = 7.1 Hz, 1H, H-3), 7.97 – 7.68 (m, 1H, H-6), 2.63 (d, J = 0.7 Hz, 3H, CH_3).

^{19}F-NMR (282 MHz, DMSO-d_6) δ/ppm = -111.79 (dd, J = 12.0, 7.1 Hz).

^{13}C-NMR, HSQC, HMBC (75 MHz, DMSO-d_6) δ/ppm = 157.7 (C-4), 154.1 (C-2), 144.2 (C-1), 143.1 (d, J = 11.1 Hz, C-5), 123.4 (C-3), 122.4 (d, J = 23.0 Hz, C-6), 19.8 (CH_3).

IR (ATR): ṽ (cm^{-1}) = 3135, 3051, 1620, 1599, 1530, 1445, 1338, 1311, 1273, 883.

The spectroscopic data are in accordance to literature [25].

Precursors, strategy B

35 Synthesis of *tert*-butyl-((*R*)-2-((5-fluoro-2,4-dinitrophenyl)amino)-4-phenylbutanoyl)-D-alaninate

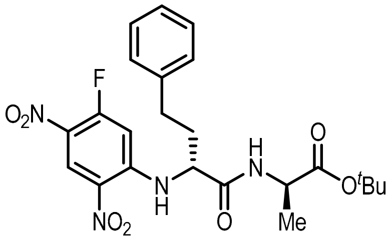

This compound was prepared starting from **39** (125 mg, 0.41 mmol, 1.0 Eq.) and 1,5-difluoro-2,4-dinitrobenzene (83 mg, 0.41 mmol, 1.0 Eq.) GP3. The product was obtained as a yellow solid (186 mg, 0.38 mmol, 93 %).

Rf. = 0.47 (1:1, DCM/^{c}Hex).

MS (ESI): m/z (%) = 435.1 (100) $[M+H \text{ (free acid)}]^+$, 513.2 (30) $[M+Na]^+$

HRMS (ESI): 489.1783 ($[M-H]^-$, calc. for $C_{23}H_{27}FN_4O_7^-$: 489.1791).

Mp. = 136.3–138.5 °C

^{1}H-NMR, COSY (300 MHz, $CDCl_3$) δ/ppm = 9.12 (d, J = 7.9 Hz, 1H, H-3^{ArF}), 8.92 – 8.76 (m, 1H, NH^{hPhe}), 7.34 – 7.15 (m, 5H, H^{Ar}), 6.50 (d, J = 7.4 Hz, 1H, NH^{Ala}), 6.40 (d, J = 13.1 Hz, 1H, -6^{ArF}), 4.47 (p, J = 7.2 Hz, 1H, α-CH^{Ala}), 3.98 (q, J = 6.4 Hz, 1H, α-CH^{hPhe}), 2.82 (t, J = 7.5 Hz, 2H, γ-CH_2^{hPhe}), 2.49 – 2.31 (m, 1H, β-CH_2^{hPhe}), 2.31 – 2.12 (m, 1H, β-CH_2^{hPhe}), 1.45 (s, 9H, CH_3), 1.39 (d, J = 7.2 Hz, 3H, β-CH_3^{Ala}).

^{19}F-NMR (282 MHz, $CDCl_3$) δ/ppm = -103.87 (ddd, J = 13.0, 8.0, 1.9 Hz).

^{13}C-NMR, HSQC, HMBC (75 MHz, $CDCl_3$) δ/ppm = 171.7 (CO^{Ala}), 169.1 (CO^{hPhe}), 160.0 (d, J = 271.9 Hz, C-5^{ArF}), 148.2 (d, J = 13.2 Hz, C-4^{ArF}), 139.5 (C-1^{hPhe}), 129.0 (2C, o-C^{hPhe}), 128.5 (2C, m-C^{hPhe}), 128.2 (C-2^{ArF}), 127.9 (C-3^{ArF}), 126.9 (p-C^{hPhe}), 102.2 (d, J = 26.9 Hz, C-6^{ArF}), 82.8 (C^{tBu}), 57.6 (α-CH^{hPhe}), 49.0 (α-CH^{Ala}), 34.4 (β-CH_2^{hPhe}), 31.7 (γ-CH_2^{hPhe}), 28.0 (CH_3), 18.5 (β-CH_3^{Ala}).

IR (ATR): $\tilde{\nu}$ (cm^{-1}) = 3327, 298, 2935, 1632, 1582, 1421, 1331, 1290, 733.

36 Synthesis of benzyl-(*R*)-2-((*R*)-2-((*tert*-butoxycarbonyl)amino)propanamido)-4-phenylbutanoate

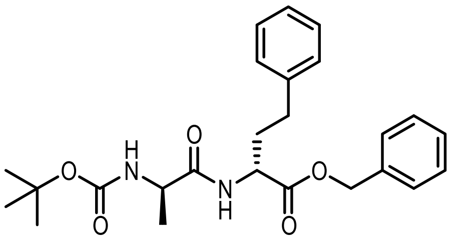

This compound was prepared starting from D-homophenylalanine benzyl ester (465 mg, 1.73 mmol, 1.01 Eq.) and Boc-*R*-alanine (330 mg, 1.74 mmol, 1.0 Eq.) following GP1. The product was obtained as a colorless solid (812 mg, 1.57 mmol, 78 %).

R_f= 0.33 (2:8, EtOAc/^{c}Hex + 2 % NEt_3).

Mp. = 82.5–83.7 °C

MS (ESI): m/z (%) = 463.2 (45) $[M+Na]^+$

HRMS (ESI): 441.2375 ($[M+H]^+$, calc. $C_{25}H_{33}N_2O_5^+$: 441.2384).

$[\alpha]_D^{22}$ = +9.7° (*c* = 1.00 g/mL, MeOH).

^{1}H-NMR, COSY (300 MHz, $CDCl_3$) δ/ppm = 7.38 – 7.33 (m, $5H^{Bn}$), 7.25 (tt, *J* = 6.5, 1.2 Hz, 2H, H-2,6^{hPhe}), 7.21 – 7.14 (m, 1H, H-4^{hPhe}), 7.12 – 7.06 (m, 2H, H-3,5^{hPhe}), 6.78 (d, *J* = 7.9 Hz, 1H, NH^{hPhe}), 5.24 – 5.08 (m, 2H, CH_2^{Bn}), 5.02 (d, *J* = 7.7 Hz, 1H, NH^{Ala}), 4.67 (td, *J* = 7.6, 4.9 Hz, 1H, α-CH^{hPhe}), 4.16 (t, *J* = 7.7 Hz, 1H, α-CH^{Ala}), 2.70 – 2.48 (m, 2H, γ-CH_2^{hPhe}), 2.19 (tdd, *J* = 12.2, 5.5, 3.7 Hz, 1H, β-CH_2^{hPhe}), 2.02 (tdd, *J* = 9.3, 7.2, 4.2 Hz, 1H, β-CH_2^{hPhe}), 1.44 (s, 9H, CH_3^{Boc}), 1.32 (d, *J* = 7.1 Hz, 3H, β-CH_3^{Ala}).

^{13}C-NMR, HSQC, HMBC (75 MHz, $CDCl_3$) δ/ppm = 172.5 (CO^{Ala}), 171.9 (CO^{hPhe}), 155.6 (CO^{Boc}), 140.8 (C-1^{hPhe}), 135.3 (C-1^{Bn}), 128.7 (C^{Ar}), 128.6 (C^{Ar}), 128.6 (C^{Ar}), 128.5 (C^{Ar}), 128.5 (C^{Ar}), 126.2 (C-4^{hPhe}), 80.2 (C_q), 67.3 (CH_2^{Bn}), 52.2 (α-CH^{hPhe}), 50.1 (α-CH^{Ala}), 33.9 (β-CH_2^{hPhe}), 31.5 (γ-CH_2^{hPhe}), 28.4 (3C, CH_3^{Boc}), 18.1 (β-CH_3^{Ala}).

IR (ATR): (cm^{-1}) = 3293, 3029, 2978, 1738, 1661, 1513, 1380, 1248, 1163, 697.

37 Synthesis of *tert*-butyl-((*S*)-2-((5-fluoro-2,4-dinitrophenyl)amino)-4-phenylbutanoyl)-L-alaninate

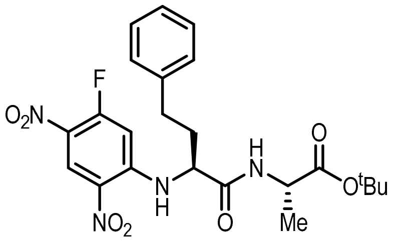

This compound was prepared starting from **39** (100 mg, 0.49 mmol, 1.0 Eq.) and 1,5-difluoro-2,4-dinitrobenzene (150 mg, 0.49 mmol, 1.0 Eq.) GP3. The product was isolated as a yellow solid (187 mg, 0.38 mmol, 78 %).

Rf. = 0.25 (^{c}Hex/EtOAc, 5:1).

MS (ESI): m/z (%) = 435.1 (100) [M+H (free acid)]$^+$, 513.2 (19.0) [M+Na]$^+$

HRMS (ESI): 490.1798 ([M]$^-$, calc. for $C_{23}H_{26}FN_4O_7^-$: 490.1822).

Mp. = 141.3–143.7 °C

$[\alpha]_D^{22}$ = -20.4° (c = 1.00 g/mL, $CHCl_3$).

^{1}H-NMR, COSY (300 MHz, $CDCl_3$) δ/ppm = 9.11 (d, J = 7.9 Hz, 1H, H-3^{Ar-F}), 8.97 – 8.79 (m, 1H, NH^{Ala}), 7.38 – 7.07 (m, 5H, H^{Ar}), 6.51 (d, J = 7.4 Hz, 1H, NH^{hPhe}), 6.41 (d, J = 13.1 Hz, 1H, H-6^{Ar-F}), 4.47 (p, J = 7.2 Hz, 1H, α-CH^{Ala}), 3.99 (q, J = 6.4 Hz, 1H, α-CH^{hPhe}), 2.82 (t, J = 7.5 Hz, 2H, γ-CH_2^{hPhe}), 2.41 (ddd, J = 14.8, 7.4, 1.6 Hz, 1H, β-CH_2^{hPhe}), 2.22 (dq, J = 14.5, 7.4 Hz, 1H, β-CH_2^{hPhe}), 1.45 (s, 9H, CH_3^{tBu}), 1.39 (d, J = 7.2 Hz, 3H, β-CH_3^{Ala}).

^{19}F-NMR (282 MHz, $CDCl_3$) δ/ppm = -103.89 (ddd, J = 13.2, 8.0, 1.9 Hz).

^{13}C-NMR, HSQC, HMBC (75 MHz, $CDCl_3$) δ/ppm = 171.6 (CO^{Ala}), 169.0 (CO^{hPhe}), 161.7 (C-5^{Ar-F}), 158.1 (C-4^{Ar-F}), 148.1 (d, J = 12.9 Hz, C-2^{Ar-F}), 139.4 (C_q^{Ar}), 128.9 (C^{Ar}), 128.3 (C^{Ar}), 127.8 (C-3^{Ar-F}), 127.3 (d, J = 111.8 Hz, C-1^{Ar-F}), 126.7 (C^{Ar}), 102.1 (d, J = 27.0 Hz, C-6^{Ar-F}), 82.7 (C^{tBu}), 57.4 (α-CH^{hPhe}), 48.9 (α-CH^{Ala}) 34.3 (β-CH_2^{hPhe}), 31.6 (γ-CH_2^{hPhe}), 27.9 (CH_3^{tBu}), 18.4 (β-CH_3^{Ala}).

IR (ATR): ṽ (cm^{-1}) = 3334, 2977, 1734, 1633, 1583, 1522, 1421, 1367, 1291, 1150.

38 Synthesis of *tert*-butyl-((*R*)-2-((5-fluoro-2,4-dinitrophenyl)amino)-4-phenylbutanoyl)-D-phenylalaninate

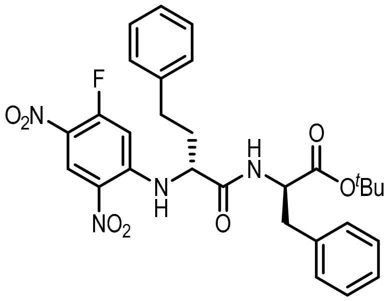

This compound was prepared starting from **40** (190 mg, 0.48 mmol, 1.0 Eq.) and 1,5-difluoro-2,4-dinitrobenzene (99 mg, 0.48 mmol, 1.0 Eq.) GP3. The product was obtained as a yellow solid (158 mg, 0.28 mmol, 58 %).

Rf. = 0.42 (8:2, ^{c}Hex/EtOAc).

MS (ESI): m/z (%) = 511.2 (100) [M+H$^+$ (free acid)], 589.3 (17) [M+Na$^+$],

HRMS (APCI): 565.2115 ([M-H]$^-$, calc. for $C_{29}H_{30}FN_4O_7^-$: 565.2104).

Mp. = 124.9–127.8 °C

[α]$_D^{22}$ = -5.0° (c = 1.00 g/mL, $CHCl_3$).

^{1}H-NMR, COSY (400 MHz, $CDCl_3$) δ/ppm = 9.10 (d, J = 7.9 Hz, 1H, H-6), 8.72 (dd, J = 6.6, 1.7 Hz, 1H, NH^{hPhe}), 7.33 – 7.18 (m, 6H, H^{Ar}), 7.12 – 7.06 (m, 4H, H^{Ar}), 6.33 (d, J = 12.9 Hz, 1H, H-3), 6.26 (d, J = 7.9 Hz, 1H, NH^{Phe}), 4.76 (dt, J = 7.9, 6.2 Hz, 1H, α-CH^{Phe}), 3.97 – 3.80 (m, 1H, α-CH^{hPhe}), 3.11 (qd, J = 14.1, 6.2 Hz, 2H, γ-CH$_2^{hPhe}$), 2.74 (td, J = 7.5, 1.6 Hz, 2H, β-CH$_2^{hPhe}$), 2.38 – 2.25 (m, 1H, β-CH$_2^{hPhe}$), 2.12 (dq, J = 14.6, 7.4 Hz, 1H, β-CH$_2^{hPhe}$), 1.43 (s, 9H, CH_3).

^{19}F-NMR (282 MHz, $CDCl_3$) δ/ppm = -103.59 (ddd, J = 13.1, 8.0, 1.8 Hz).

^{13}C-NMR, HSQC, HMBC (101 MHz, $CDCl_3$) δ/ppm = 170.0 (CO^{hPhe}), 169.1 (CO^{Phe}), 159.8 (d, J = 272.3 Hz, C-5^{ArF}), 148.0 (d, J = 13.2 Hz, C-4^{ArF}), 139.3 (C-1^{hPhe}), 135.5 (C-1^{Phe}), 129.2, 128.8, 128.6, 128.3, 128.0, 127.7 (C-3^{ArF}), 127.3 (C-1^{ArF}), 126.7 (C-2^{ArF}), 102.1 (d, J = 27.1 Hz, C-6^{ArF}), 83.0 (C^{tBu}), 57.4 (α-CH^{hPhe}), 53.4 (α-CH^{Phe}), 37.6 (β-CH$_2^{Phe}$), 34.1 (β-CH$_2^{hPhe}$), 31.6 (γ-CH$_2^{hPhe}$), 27.9 (3C, CH$_3^{tBu}$).

IR (ATR): $\tilde{\nu}$ (cm^{-1}) = 3331, 2929, 1723, 1630, 1583, 1534, 1326, 1119, 1047, 699.

39 Synthesis of *tert*-butyl-((*S*)-2-amino-4-phenylbutanoyl)-L-alaninate

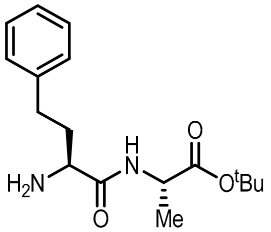

This compound was prepared starting from **42** (260 mg, 0.59 mmol, 1.0 Eq.) in EtOH following GP2. The product was obtained as a colorless oil (190 mg, 0.59 mmol, quant.).

Rf. = 0.72 (DCM/MeOH/NEt_3, 9:0.8:0.2).

MS (ESI): m/z (%) = 307.2 (100) $[M+H]^+$

HRMS (ESI): 307.1984 ($[M+H]^+$, calc. for $C_{17}H_{27}N_2O_3^+$: 307.1992).

$[\alpha]_D^{22}$ = +3.0° (c = 1.00 g/mL, $CHCl_3$).

^{1}H-NMR, COSY (400 MHz, $CDCl_3$) δ/ppm = 7.74 (d, J = 7.5 Hz, 1H, NH^{Ala}), 7.39 – 7.03 (m, 5H, H^{Ar}), 4.42 (p, J = 7.2 Hz, 1H, α-CH^{Ala}), 3.53 (s, 1H, α-CH^{hPhe}), 3.42 – 2.84 (m, 2H, NH_2^{hPhe}), 2.83 – 2.69 (m, 2H, γ-CH_2^{hPhe}), 2.26 – 2.09 (m, 1H, β-CH_2^{hPhe}), 1.94 – 1.84 (m, 1H, β-CH_2^{hPhe}), 1.45 (s, 9H, CH_3^{tBu}), 1.36 (d, J = 7.1 Hz, 3H, β-CH_3^{Ala}).

^{13}C-NMR, HSQC, HMBC (101 MHz, $CDCl_3$) δ/ppm = 173.5 (CO^{hPhe}), 172.2 (CO^{Ala}), 141.0 (C^{Ar}), 128.5 (C^{Ar}), 128.4 (C^{Ar}), 126.1 (C^{Ar}), 81.9 (C^{tBu}), 54.5 (α-CH^{hPhe}), 48.4 (α-CH^{Ala}), 36.2 (β-CH_2^{hPhe}), 31.9 (γ-CH_2^{hPhe}), 28.0 (CH_3^{tBu}), 27.9 (CH_3^{tBu}), 18.5 (β-CH_3^{Ala}).

IR (ATR): ṽ (cm^{-1}) = 3292, 2026, 2979, 1734, 1652, 1498, 1454, 1393, 1151, 700.

40 Synthesis of *tert*-butyl-((*R*)-2-amino-4-phenylbutanoyl)-D-phenylalaninate

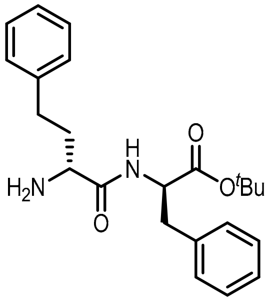

This compound was prepared starting from **43** (250 mg, 0.484 mmol) following GP2. The product was obtained as a colorless, viscous oil (189 mg, 0.483 mmol, quant.).

Rf. = 0.5 (DCM/MeOH, 98:2).

MS (ESI): m/z (%) = 383.2 (100) [M+H$^+$

HRMS (ESI): 383.2323 ([M+H]$^+$, calc. for $C_{23}H_{31}N_2O_3^+$: 383.2329).

$[\alpha]_D^{22}$ = +8.4° (c = 1.00 g/mL, $CHCl_3$).

^{1}H-NMR, COSY (300 MHz, $CDCl_3$) δ/ppm = 7.67 (d, J = 8.4 Hz, 1H, NH^{Phe}), 7.31 – 7.11 (m, 10H, H^{Ar}), 4.77 (dt, J = 8.2, 6.4 Hz, 1H, α-CH^{Phe}), 3.35 (dd, J = 8.1, 4.6 Hz, 1H, α-CH^{hPhe}), 3.09 (qd, J = 13.8, 6.4 Hz, 2H, β-CH_2^{Phe}), 2.69 – 2.56 (m, 2H, γ-CH_2^{hPhe}), 2.14 – 1.98 (m, 1H, β-CH_2^{hPhe}), 1.82 – 1.63 (m, 1H, β-CH_2^{hPhe}), 1.42 (s, 9H, CH_3).

^{13}C-NMR, HSQC, HMBC (75 MHz, $CDCl_3$) δ/ppm = 174.5 (CO^{hPhe}), 170.9 (CO^{Phe}), 141.3 (C-1^{hPhe}), 136.5 (C-1^{Phe}), 129.5 (C^{Ar}), 128.6 (C^{Ar}), 128.5 (C^{Ar}), 128.5 (C^{Ar}), 128.4 (C^{Ar}), 127.0 (C^{Aer}), 126.1 (C^{Ar}), 82.2 (C^{tBu}), 54.8 (α-CH^{hPhe}), 53.1 (α-CH^{Phe}), 38.4 (β-CH_2^{Phe}), 36.7 (β-CH_2^{hPhe}), 32.0 (γ-CH_2^{hPhe}), 28.0 (3C, CH_3^{tBu}).

IR (ATR): ṽ (cm^{-1}) = 3323, 3062, 2930, 1731, 1655, 1497, 1368, 1251, 1153, 700.

42 Synthesis of *tert*-butyl-((*R*)-2-amino-4-phenylbutanoyl)-D-alaninate

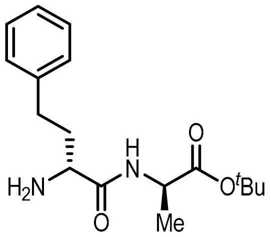

This compound was prepared starting from **44** (220 mg, 0.50 mmol, 1.0 Eq.) in EtOH following GP2. The product was obtained as a colorless oil (140 mg, 0.46 mmol, 92 %).

Rf. = 0.62 (DCM (2 % NEt_3)).

MS (ESI): m/z (%) = 307.2 (100) $[M+H\text{ (free acid)}]^+$

HRMS (ESI): 307.2015 ($[M+Na]^+$, calc. for $C_{25}H_{32}N_2O_5Na^+$: 307.2016).

$[\alpha]_D^{22}$ = +49.2° (c = 1.00 g/mL, $CHCl_3$).

^{1}H-NMR, COSY (300 MHz, $CDCl_3$) δ/ppm = 7.69 (d, J = 7.8 Hz, 1H, NH^{Ala}), 7.32 – 7.23 (m, 2H, H^{Ar}), 7.23 – 7.13 (m, 3H, H^{Ar}), 4.53 – 4.36 (m, 1H, α-CH^{Ala}), 3.38 (dd, J = 8.2, 4.5 Hz, 1H, α-CH^{hPhe}), 2.72 (ddd, J = 9.4, 6.6, 3.3 Hz, 2H, γ-CH_2^{hPhe}), 2.17 (dddd, J = 13.8, 9.4, 7.0, 4.5 Hz, 1H, β-CH_2^{hPhe}), 1.81 (dddd, J = 13.8, 9.1, 8.1, 6.5 Hz, 1H, β-CH_2^{hPhe}), 1.46 (s, 9H, CH_3), 1.37 (d, J = 7.1 Hz, 3H, β-CH_3^{Ala}).

^{13}C-NMR, HSQC, HMBC (75 MHz, $CDCl_3$) δ/ppm = 174.9 (CO^{hPhe}), 172.8 (CO^{Ala}), 141.7 (C-1^{hPhe}), 128.9 (2C, C^{Ar}), 128.9 (2C, C^{Ar}), 126.5 (p-C), 82.3 (C^{tBu}), 55.2 (α-CH^{hPhe}), 48.7 (α-CH^{Ala}), 37.2 (β-CH_2^{hPhe}), 32.5 (γ-CH_2^{hPhe}), 28.4 (3C, CH_3^{tBu}), 19.2 (β-CH_3^{Ala}).

IR (ATR): ṽ (cm^{-1}) = 3306, 3062, 2933, 1733, 1651, 1510, 1454, 1227, 1155, 700.

42 Synthesis of *tert*-butyl ((*S*)-2-(((benzyloxy)carbonyl)amino)-4-phenylbutanoyl)-L-alaninate

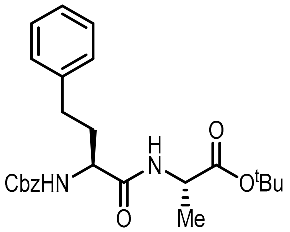

This compound was prepared starting from Z-L-homophenylalanine (500 mg, 1.59 mmol, 1.01 Eq.) and L-alanine-*tert*-butylester hydrochloride (287 mg, 1.58 mmol, 1.0 Eq.) following GP1. The product was obtained as a colorless solid (670 mg, 1.52 mmol, 96 %).

Rf. = 0.23 (^{c}Hex/EtOAc, 7:3).

MS (ESI): m/z (%) = 385.2 (100) [M+H (free acid)]$^+$, 463.2 (19.0) [M+Na]$^+$

HRMS (ESI): 463.2210 ([M+Na]$^+$, calc. for $C_{25}H_{32}N_2O_5Na^+$: 463.2203).

Mp. = 105.2–107.1 °C

$[\alpha]_D^{22}$ = +3.9° (c = 1.00 g/mL, $CHCl_3$).

^{1}H-NMR, COSY (400 MHz, $CDCl_3$) δ/ppm = 7.35 (q, J = 4.0, 3.2 Hz, 5H, $H^{Ar, Cbz}$), 7.27 – 7.12 (m, 5H, $H^{Ar, hPhe}$), 6.39 (d, J = 7.4 Hz, 1H, NH^{Ala}), 5.39 (d, J = 8.2 Hz, 1H, NH^{hPhe}), 5.12 (s, 2H, CH_2^{Cbz}), 4.41 (p, J = 7.1 Hz, 1H, α-CH^{Ala}), 4.19 (d, J = 7.6 Hz, 1H, α-CH^{hPhe}), 2.68 (t, J = 7.9 Hz, 2H, γ-CH_2^{hPhe}), 2.32 – 1.81 (m, 2H, β-CH_2^{hPhe}), 1.46 (s, 9H, CH_3^{tBu}), 1.35 (d, J = 7.1 Hz, 3H, β-CH_3^{Ala}).

^{13}C-NMR, HSQC, HMBC (101 MHz, $CDCl_3$) δ/ppm = 171.8 (CO^{Ala}), 170.8 (CO^{hPhe}), 156.1 (CO^{Cbz}), 140.7 (C^{Ar}), 136.2 (C-1^{Cbz}), 128.6 (C^{Ar}), 128.5 (C^{Ar}), 128.5 (C^{Ar}), 128.1 (C^{Ar}), 126.2 (C^{Ar}), 82.2 (C^{tBu}), 67.1 (CH_2^{Cbz}), 48.8 (α-CH^{hPhe}), 45.2 (α-CH^{Ala}), 34.5 (β-CH_2^{hPhe}), 31.6 (γ-CH_2^{hPhe}), 28.0 (CH_3^{tBu}), 18.5 (β-CH_3^{Ala}).

IR (ATR): ṽ (cm^{-1}) = 3327, 2979, 1721, 1658, 1604, 1535, 1368, 1241, 1149, 698.

43 Synthesis of *tert*-butyl-((*R*)-2-(((benzyloxy)carbonyl)amino)-4-phenylbutanoyl)-D-phenylalaninate

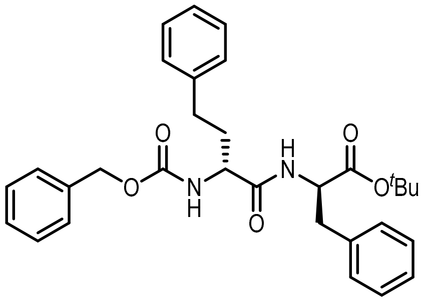

This compound was prepared starting from **45** (634 mg, 2.02 mmol, 1.01 Eq.) and D-phenylalanine-*tert*-butylester hydrochloride (515 mg, 2.0 mmol, 1.0 Eq.) following GP1. The product was obtained as a colorless, viscous oil (812 mg, 1.57 mmol, 78 %).

Rf. = 0.6 (^{c}Hex/EtOAc, 1:1).

MS (ESI): m/z (%) = 461.2 (100) [M+H (free acid)]$^+$, 539.2 (27.0) [M+Na]$^+$

HRMS (ESI): 539.2512 ([M+Na]$^+$, calc. for $C_{31}H_{36}N_2O_5Na^+$: 539.2516).

$[\alpha]_D^{22}$ = -24.9° (c = 1.00 g/mL, $CHCl_3$).

^{1}H-NMR, COSY (300 MHz, $CDCl_3$) δ/ppm = 7.41 (d, J = 4.1 Hz, 5H, H^{Ar}), 7.37 – 7.28 (m, 5H, H^{Ar}), 7.22 – 7.13 (m, 5H, H^{Ar}), 6.52 (d, J = 7.8 Hz, 1H, NH^{Phe}), 5.46 (d, J = 8.2 Hz, 1H, NH^{hPhe}), 5.25 – 5.08 (m, 2H, CH_2^{Cbz}), 4.79 (dt, J = 7.8, 6.1 Hz, 1H α-CH^{Phe}), 4.26 (q, J = 7.4 Hz, 1H, α-CH^{hPhe}), 3.13 (d, J = 6.1 Hz, 2H, β-CH_2^{Phe}), 2.70 (t, J = 7.9 Hz, 2H, γ-CH_2^{hPhe}), 2.30 – 2.12 (m, 1H, β-CH_2^{hPhe}), 1.96 (dq, J = 15.1, 7.8 Hz, 1H, β-CH_2^{hPhe}), 1.46 (s, 9H, CH_3).

^{13}C-NMR, HSQC, HMBC (75 MHz, $CDCl_3$) δ/ppm = 171.0 (CO^{hPhe}), 170.3 (CO^{Phe}), 156.1 (CO^{Cbz}), 140.9 (C-1^{hPhe}), 136.3 (C-1^{Cbz}), 136.1 (C-1^{Phe}), 129.6 (C^{Ar}), 128.7 (C^{Ar}), 128.6 (C^{Ar}), 128.5 (C^{Ar}), 128.3 (C^{Ar}), 128.2 (C^{Ar}), 127.1 (C^{Ar}), 126.3 (C^{Ar}), 82.6 (C^{tBu}), 67.2 (CH_2^{Cbz}), 54.6 (α-CH^{hPhe}), 53.7 (α-CH^{Phe}), 38.1 (β-CH_2^{Phe}), 34.4 (β-CH_2^{hPhe}), 31.7 (γ-CH_2^{hPhe}), 28.1 (3C, CH_3^{tBu}).

IR (ATR): ṽ (cm^{-1}) = 3306, 3063, 2932, 1728, 1656, 1538, 1455, 1227, 1154, 698.

44 Synthesis of *tert*-butyl-((*R*)-2-(((benzyloxy)carbonyl)amino)-4-phenylbutanoyl)-D-alaninate

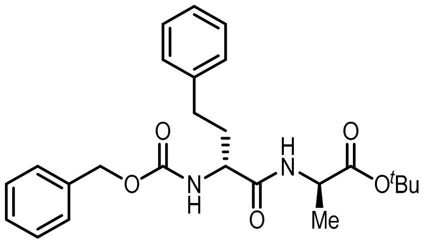

This compound was prepared starting from **45** (634 mg, 2.02 mmol, 1.01 Eq.) and D-alanine-*tert*-butylester hydrochloride (368 mg, 2.0 mmol, 1.0 Eq.) following GP1. The product was obtained as a colorless solid (610 mg, 1.38 mmol, 69 %).

Rf. = 0.23 (^{c}Hex/EtOAc, 7:3).

MS (ESI): m/z (%) = 385.2 (100) [M+H (free acid)]$^+$, 463.2 (19.0) [M+Na]$^+$

HRMS (ESI): 463.2210 ([M+Na]$^+$, calc. for $C_{25}H_{32}N_2O_5Na^+$: 463.2203).

Mp. = 116.5–116.6 °C

$[\alpha]_D^{22}$ = +3.9° (c = 1.00 g/mL, $CHCl_3$).

^{1}H-NMR, COSY (400 MHz, $CDCl_3$) δ/ppm = 7.38 – 7.30 (m, 5H, H^{Ar}), 7.29 – 7.24 (m, 3H, $H^{Ar, Cbz}$), 7.24 – 7.09 (m, 2H, $H^{Ar, Cbz}$), 6.38 (d, J = 7.3 Hz, 1H), 5.37 (d, J = 8.3 Hz, 1H, NH^{hPhe}), 5.12 (s, 2H, CH_2^{Cbz}), 4.41 (p, J = 7.2 Hz, 1H, α-CH^{Ala}), 4.19 (d, J = 7.1 Hz, 1H, α-CH^{hPhe}), 2.69 (t, J = 7.9 Hz, 2H, γ-CH_2^{hPhe}), 2.17 (dtd, J = 13.7, 7.9, 5.7 Hz, 1H, β-CH_2^{hPhe}), 2.01 – 1.88 (m, 1H, β-CH_2^{hPhe}), 1.46 (s, 9H, CH_3), 1.35 (d, J = 7.2 Hz, 3H, β-CH_3^{Ala}).

^{13}C-NMR, HSQC, HMBC (101 MHz, $CDCl_3$) δ/ppm = 171.9 (CO^{Ala}), 170.9 (CO^{hPhe}), 156.2 (CO^{Cbz}), 140.9 (C-1^{hPhe}), 136.4 (C-1^{Cbz}), 128.7 (C^{Ar}), 128.7 (C^{Ar}), 128.6 (C^{Ar}), 128.3 (C^{Ar}), 128.2 (C^{Ar}), 126.3 (C^{Ar}), 82.3 (C^{tBu}), 67.2 (CH_2^{Cbz}), 54.6 (α-CH^{hPhe}), 48.9 (α-CH^{Ala}), 34.6 (β-CH_2^{hPhe}), 31.7 (γ-CH_2^{hPhe}), 28.1 (CH_3^{tBu}), 18.6 (β-CH_3^{Ala}).

IR (ATR): ṽ (cm^{-1}) = 3295, 3062, 2935, 1734, 1657, 1538, 1454, 1368 1150, 699.

45 Synthesis of Z-*D*-homophenylalanine

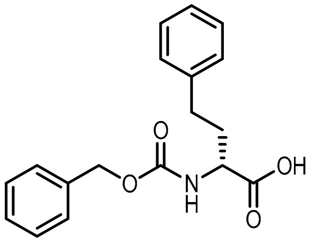

Following a procedure from Vater et al. [26].

To a well stirred solution in a 250 mL flask of 60 mL THF and 20 mL H_2O were D-homophenylalanine (3.22 g, 18.0 mmol, 1.0 Eq.) and Z-Osu (4.49 g, 18.0 mmol, 1.0 Eq.) added. To this suspension was triethylamine (10 mL, 72.0 mmol, 4.0 Eq.) added and the reaction mixture was stirred for 18 h. After that 200 mL of EtOAc were added and the phases separated. The organic phase was washed three times with 100 mL 1 N HCl and once with brine. The organic phase was dried over Na_2SO_4 and the solvent was removed under reduced pressure. The product was obtained as a colorless solid (5.5 g, 17.5 mmol, 98 %) and was used without further purification.

Rf. = 0.12 (DCM/MeOH, 98:2).

MS (ESI): m/z (%) = 336.1 (35) $[M+Na]^+$

Mp. = 103.1–103.9 °C

$[\alpha]_D^{22}$ = +14.8° (c = 1.00 g/mL, MeOH).

^{1}H-NMR, COSY (300 MHz, $CDCl_3$) δ/ppm = 10.12 (s, 1H, OH), 7.46 – 6.68 (m, 10H, H^{Ar}), 5.44 (d, J = 8.3 Hz, 1H, NH), 5.16 (t, J = 5.2 Hz, 2H, CH_2^{Bn}), 4.55 – 4.24 (m, 1H, α-CH^{hPhe}), 2.72 (t, J = 8.0 Hz, 2H, - γ-CH_2^{hPhe}), 2.24 (tdd, J = 14.7, 9.1, 5.1 Hz, 1H, β-CH_2^{hPhe}), 2.03 (dq, J = 15.3, 7.7 Hz, 1H, β-CH_2^{hPhe}).

^{13}C-NMR, HSQC, HMBC (75 MHz, $CDCl_3$) δ/ppm = 177.2 (CO^{hPhe}), 156.2 (CO), 140.5 (C_q^{CbZ}), 136.1 (C_q^{hPhe}), 128.7 (C^{Ar}), 128.6 (C^{Ar}), 128.5 (C^{Ar}), 128.4 (C^{Ar}), 128.2 (C^{Ar}), 126.3 (C^{Ar}), 67.3 (CH_2^{Bn}), 53.6 (α-CH^{hPhe}), 34.0 (γ-CH_2^{hPhe}), 31.6 (β-CH_2^{hPhe}).

IR (ATR): ṽ (cm^{-1}) = 3029, 1716, 1528, 1439, 1429, 1336, 1233, 1212, 1051, 775.

The spectroscopic data are in accordance to literature [26].

Synthesis of Candesartan

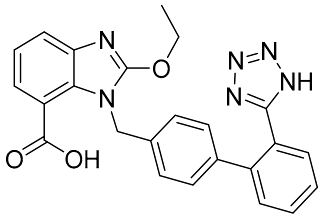

Commercially available candesartan cilexetil (40 mg,0.06 mmol) was dissolved in MeOH/1 M NaOH (1:1, 10 mL) and stirred for 12 h at rt. The solution was evaporated under reduced pressure and the crude product was purified via RP-HPLC (InfinityLab Pursuit XRs C18, 30 x 250 mm, 5 µm, preparative LC column, gradient method: 10 % ACN (0.1 % formic acid) to 100 % ACN (0.1 % formic acid) over 25 min) to yield candesartan as a colorless solid (13 mg, 0.03 mmol, 50%).

MS (ESI): m/z (%) = 441.2 (100) ($[M+H]^+$, calc. for $C_{24}H_{21}N_6O_3^+$: 441.4).

Mp. = 188 – 190 °C

^{1}H-NMR, (400 MHz, DMSO-*d*$_6$) δ/ppm = 7.69 – 7.60 (m, 3H), 7.56 – 7.46 (m, 3H), 7.17 (t, *J* = 8.0 Hz, 1H), 7.00 (d, *J* = 8.0 Hz, 2H), 6.92 (d, *J* = 8.0 Hz, 2H), 5.62 (s, 2H), 4.57 (q, *J* = 7.0 Hz, 2H), 1.37 (t, *J* = 7.0 Hz, 3H).

^{13}C-NMR, (101 MHz, DMSO-*d*$_6$): δ/ppm = 167.5, 158.2, 141.6, 140.9, 138.1, 136.7, 131.2, 130.9, 130.6, 129.0, 127.7, 126.4, 123.4, 121.4, 120.6, 116.5, 66.4, 46.2, 14.3.

FT-IR: $\tilde{\nu}$ (cm^{-1}) = 744, 1032, 1135, 1241, 1279, 1432, 1479, 1554, 1601, 1701.

The spectroscopic data are in accordance to literature.[27]

References

1. Ludewig, S.; Kossner, M.; Schiller, M.; Baumann, K.; Schirmeister, T. Enzyme Kinetics and Hit Validation in Fluorimetric Protease Assays. *Curr Top Med Chem* **2010**, *10*, 368–382, doi:10.2174/156802610790725498.
2. Weitner, T.; Friganović, T.; Šakić, D. Inner Filter Effect Correction for Fluorescence Measurements in Microplates Using Variable Vertical Axis Focus. *Anal Chem* **2022**, *94*, 7107–7114, doi:10.1021/acs.analchem.2c01031.
3. Klein, P.; Johe, P.; Wagner, A.; Jung, S.; Kühlborn, J.; Barthels, F.; Tenzer, S.; Distler, U.; Waigel, W.; Engels, B.; et al. New Cysteine Protease Inhibitors: Electrophilic (Het)Arenes and Unexpected Prodrug Identification for the Trypanosoma Protease Rhodesain. *Molecules* **2020**, *25*, 1451, doi:10.3390/molecules25061451.
4. Schirmeister, T.; Kesselring, J.; Jung, S.; Schneider, T.H.; Weickert, A.; Becker, J.; Lee, W.; Bamberger, D.; Wich, P.R.; Distler, U.; et al. Quantum Chemical-Based Protocol for the Rational Design of Covalent Inhibitors. *J Am Chem Soc* **2016**, *138*, 8332–8335, doi:10.1021/jacs.6b03052.
5. Caffrey, C.R.; Hansell, E.; Lucas, K.D.; Brinen, L.S.; Alvarez Hernandez, A.; Cheng, J.; Gwaltney, S.L.; Roush, W.R.; Stierhof, Y.D.; Bogyo, M.; et al. Active Site Mapping, Biochemical Properties and Subcellular Localization of Rhodesain, the Major Cysteine Protease of Trypanosoma Brucei Rhodesiense. *Mol Biochem Parasitol* **2001**, *118*, 61–73, doi:10.1016/S0166-6851(01)00368-1.
6. Schirmeister, T.; Kesselring, J.; Jung, S.; Schneider, T.H.; Weickert, A.; Becker, J.; Lee, W.; Bamberger, D.; Wich, P.R.; Distler, U.; et al. Quantum Chemical-Based Protocol for the Rational Design of Covalent Inhibitors. *J Am Chem Soc* **2016**, *138*, 8332–8335, doi:10.1021/jacs.6b03052.
7. Klein, P.; Johe, P.; Wagner, A.; Jung, S.; Kühlborn, J.; Barthels, F.; Tenzer, S.; Distler, U.; Waigel, W.; Engels, B.; et al. New Cysteine Protease Inhibitors : Electrophilic (Het)Arenes and Unexpected Prodrug Identification for the Trypanosoma Protease Rhodesain. **2020**.
8. Cheng, Y.-C.; Prusoff, W.H. Relationship between the Inhibition Constant (KI) and the Concentration of Inhibitor Which Causes 50 per Cent Inhibition (I50) of an Enzymatic Reaction. *Biochem Pharmacol* **1973**, *22*, 3099–3108, doi:10.1016/0006-2952(73)90196-2.
9. Jung, S.; Fuchs, N.; Johe, P.; Wagner, A.; Diehl, E.; Yuliani, T.; Zimmer, C.; Barthels, F.; Zimmermann, R.A.; Klein, P.; et al. Fluorovinylsulfones and -Sulfonates as Potent Covalent Reversible Inhibitors of the Trypanosomal Cysteine Protease Rhodesain: Structure-Activity Relationship, Inhibition Mechanism, Metabolism, and in Vivo Studies. *J Med Chem* **2021**, *64*, 12322–12358, doi:10.1021/acs.jmedchem.1c01002.
10. Strohalm, M.; Hassman, M.; Košata, B.; Kodíček, M. MMass Data Miner: An Open Source Alternative for Mass Spectrometric Data Analysis. *Rapid Communications in Mass Spectrometry* **2008**, *22*, 905–908, doi:10.1002/rcm.3444.
11. Kansy, M.; Senner, F.; Gubernator, K. Physicochemical High Throughput Screening: Parallel Artificial Membrane Permeation Assay in the Description of Passive Absorption Processes - SI. *J Med Chem* **1998**, *41*, 1007–1010, doi:10.1021/jm970530e.
12. Kerr, I.D.; Lee, J.H.; Farady, C.J.; Marion, R.; Rickert, M.; Sajid, M.; Pandey, K.C.; Caffrey, C.R.; Legac, J.; Hansell, E.; et al. Vinyl Sulfones as Antiparasitic Agents and a Structural Basis for Drug Design. *Journal of Biological Chemistry* **2009**, *284*, 25697–25703, doi:10.1074/jbc.M109.014340.
13. Halgren, T. a Merck Molecular Force Field. *J. Comput. Chem.* **1996**, *17*, 490–519, doi:10.1002/(SICI)1096-987X(199604)17:5/6<520::AID-JCC2>3.0.CO;2-W.
14. Neese, F. The ORCA Program System-Version 5.0. *WIREs Computational Molecular Science* **2022**, *12*, doi:10.1002/wcms.1606.
15. Chai, J.-D.; Head-Gordon, M. Long-Range Corrected Hybrid Density Functionals with Damped Atom–Atom Dispersion Corrections. *Physical Chemistry Chemical Physics* **2008**, *10*, 6615, doi:10.1039/b810189b.

16. Grimme, S.; Antony, J.; Ehrlich, S.; Krieg, H. A Consistent and Accurate Ab Initio Parametrization of Density Functional Dispersion Correction (DFT-D) for the 94 Elements H-Pu. *J Chem Phys* **2010**, *132*, doi:10.1063/1.3382344.
17. Weigend, F.; Ahlrichs, R. Balanced Basis Sets of Split Valence, Triple Zeta Valence and Quadruple Zeta Valence Quality for H to Rn: Design and Assessment of Accuracy. *Physical Chemistry Chemical Physics* **2005**, *7*, 3297, doi:10.1039/b508541a.
18. Zheng, J.; Xu, X.; Truhlar, D.G. Minimally Augmented Karlsruhe Basis Sets. *Theor Chem Acc* **2011**, *128*, 295–305, doi:10.1007/s00214-010-0846-z.
19. Stoychev, G.L.; Auer, A.A.; Neese, F. Automatic Generation of Auxiliary Basis Sets. *J Chem Theory Comput* **2017**, *13*, 554–562, doi:10.1021/acs.jctc.6b01041.
20. Barone, V.; Cossi, M. Quantum Calculation of Molecular Energies and Energy Gradients in Solution by a Conductor Solvent Model. *J Phys Chem A* **1998**, *102*, 1995–2001, doi:10.1021/jp9716997.
21. Ben-Naim, A. Standard Thermodynamics of Transfer. Uses and Misuses. *J Phys Chem* **1978**, *82*, 792–803, doi:10.1021/j100496a008.
22. Pliego Jr, J.R.; Riveros, J.M. Gibbs Energy of Solvation of Organic Ions in Aqueous and Dimethyl Sulfoxide Solutions. *Physical Chemistry Chemical Physics* **2002**, *4*, 1622–1627, doi:10.1039/b109595a.
23. Muramatsu, W.; Yamamoto, H. Peptide Bond Formation of Amino Acids by Transient Masking with Silylating Reagents. *J Am Chem Soc* **2021**, *143*, 6792–6797, doi:10.1021/jacs.1c02600.
24. Klein, P.; Barthels, F.; Johe, P.; Wagner, A.; Tenzer, S.; Distler, U.; Le, T.A.; Schmid, P.; Engel, V.; Engels, B.; et al. Naphthoquinones as Covalent Reversible Inhibitors of Cysteine Proteases—Studies on Inhibition Mechanism and Kinetics. *Molecules* **2020**, *25*, doi:10.3390/molecules25092064.
25. Egawa, H.; Kataoka, M.; Shibamori, K.-I.; Miyamoto, T.; Nakano, J.; Matsumoto, J.-I. A New Synthetic Route to 7-Halo-1-Cyclopropyl-6-Fluoro-1,4-Dihydro-4-Oxoquinoline-3-Carboxylic Acid, an Intermediate for the Synthesis of Quinolone Antibacterial Agents. *J Heterocycl Chem* **1987**, *24*, 181–185, doi:https://doi.org/10.1002/jhet.5570240134.
26. Draper, R.W.; Hou, D.; Iyer, R.; Lee, G.M.; Liang, J.T.; Mas, J.L.; Vater, E.J. Novel Stereoselective Syntheses of the Fused Benzazepine Dopamine D1 Antagonist (6aS,13bR)-11-Chloro-6,6a,7,8,9,13b-Hexahydro-7-Methyl- 5H-Benzo[d]Naphth[2,1-b]Azepin-12-Ol (Sch 39166): 2. l-Homophenylalanine-Based Syntheses. *Org Process Res Dev* **1998**, *2*, 186–193, doi:10.1021/op970122k.
27. Fotakis, C.; Christodouleas, D.; Zoumpoulakis, P.; Kritsi, E.; Benetis, N.-P.; Mavromoustakos, T.; Reis, H.; Gili, A.; Papadopoulos, M.G.; Zervou, M. Comparative Biophysical Studies of Sartan Class Drug Molecules Losartan and Candesartan (CV-11974) with Membrane Bilayers. *J Phys Chem B* **2011**, *115*, 6180–6192, doi:10.1021/jp110371k.

<u>Spectra</u>

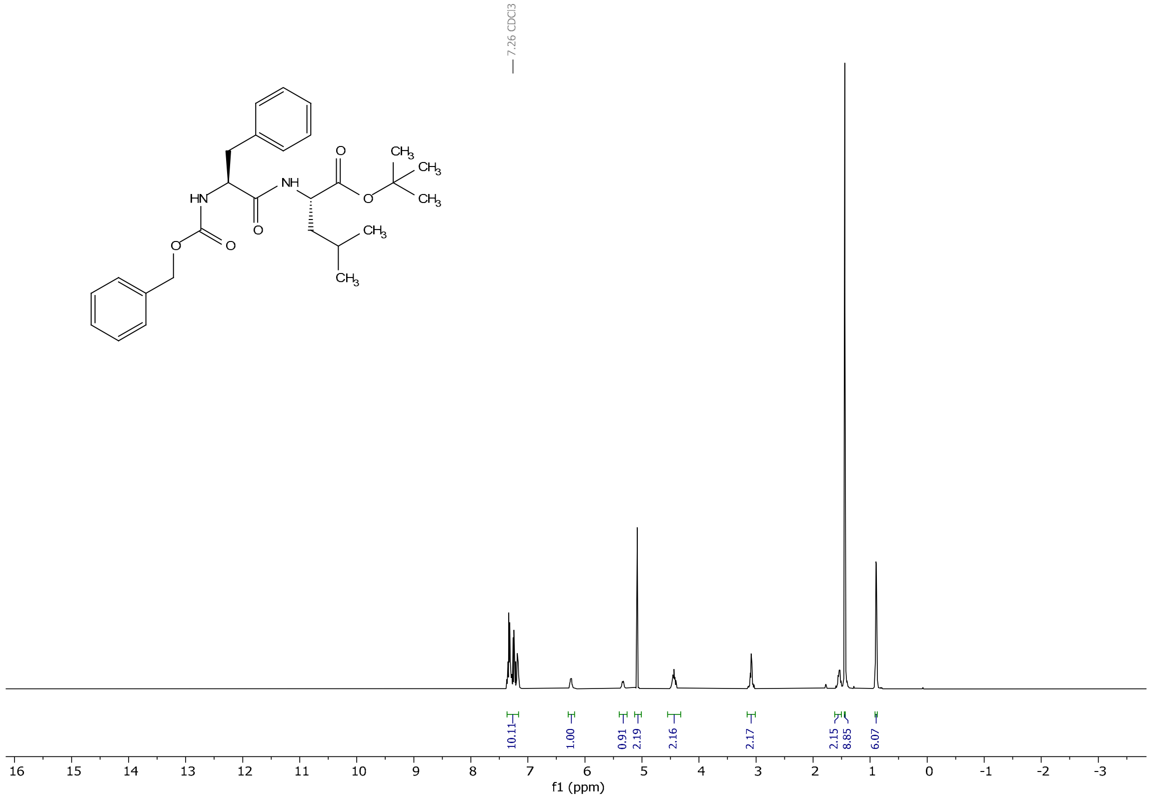

Spectrum 1: ^{1}H-NMR of **32**

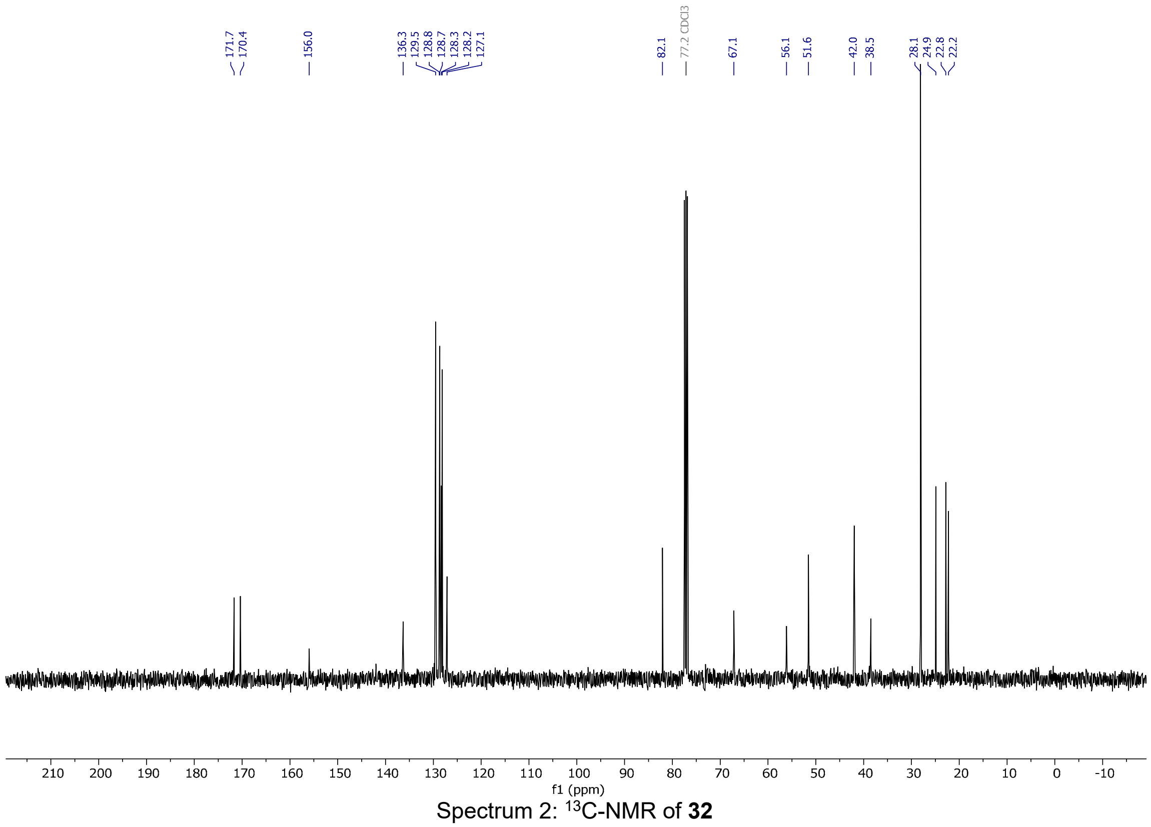

Spectrum 2: ^{13}C-NMR of **32**

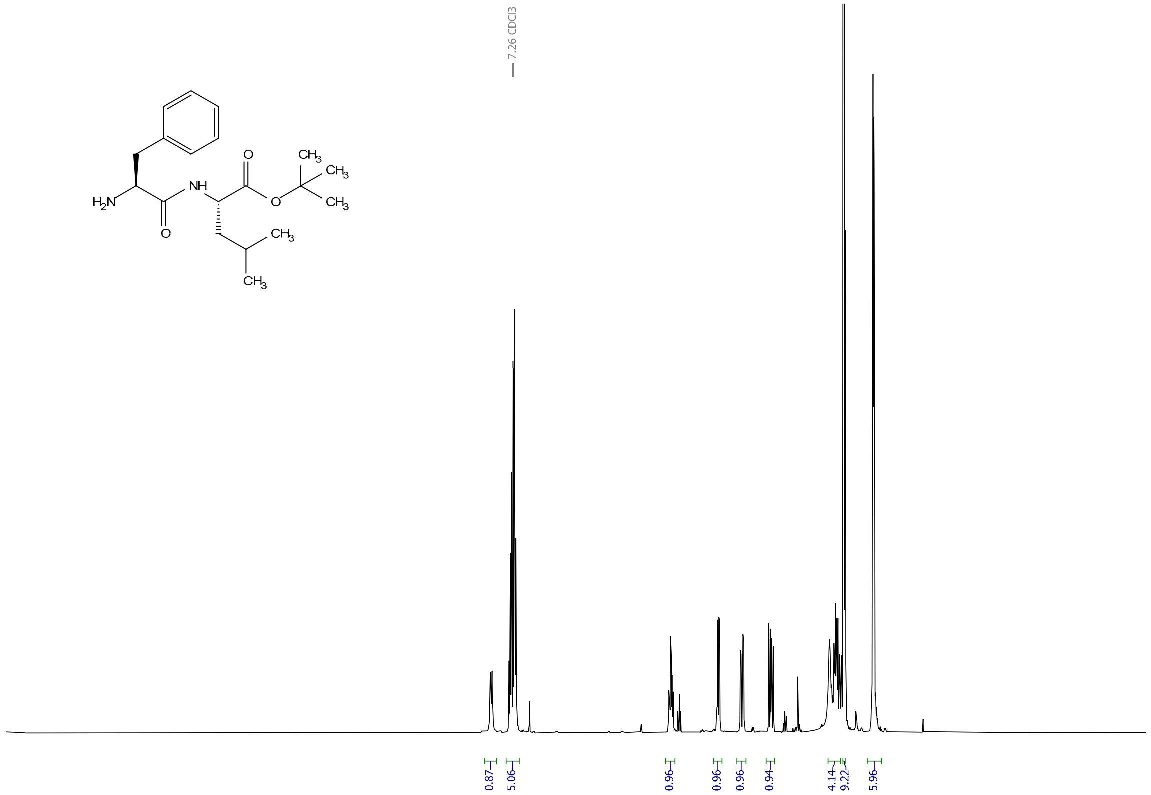

Spectrum 3: ^{1}H-NMR of **31**

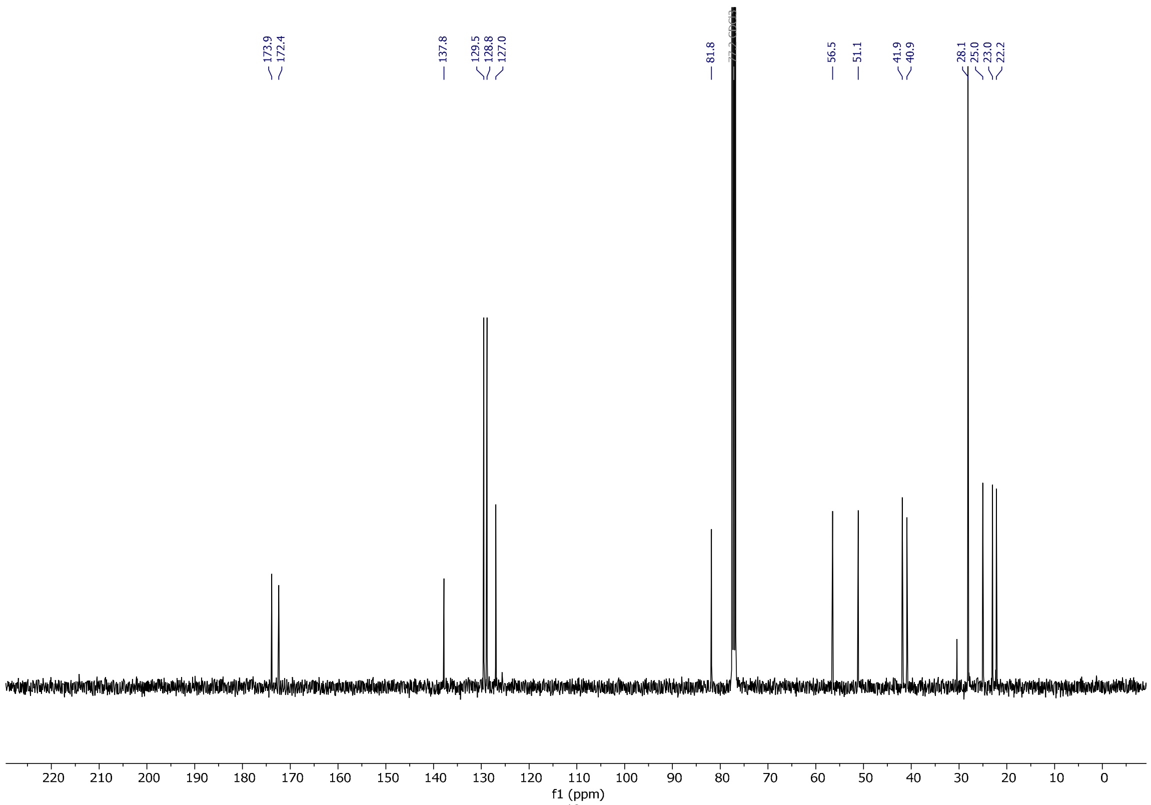

Spectrum 4: ^{13}C-NMR of **31**

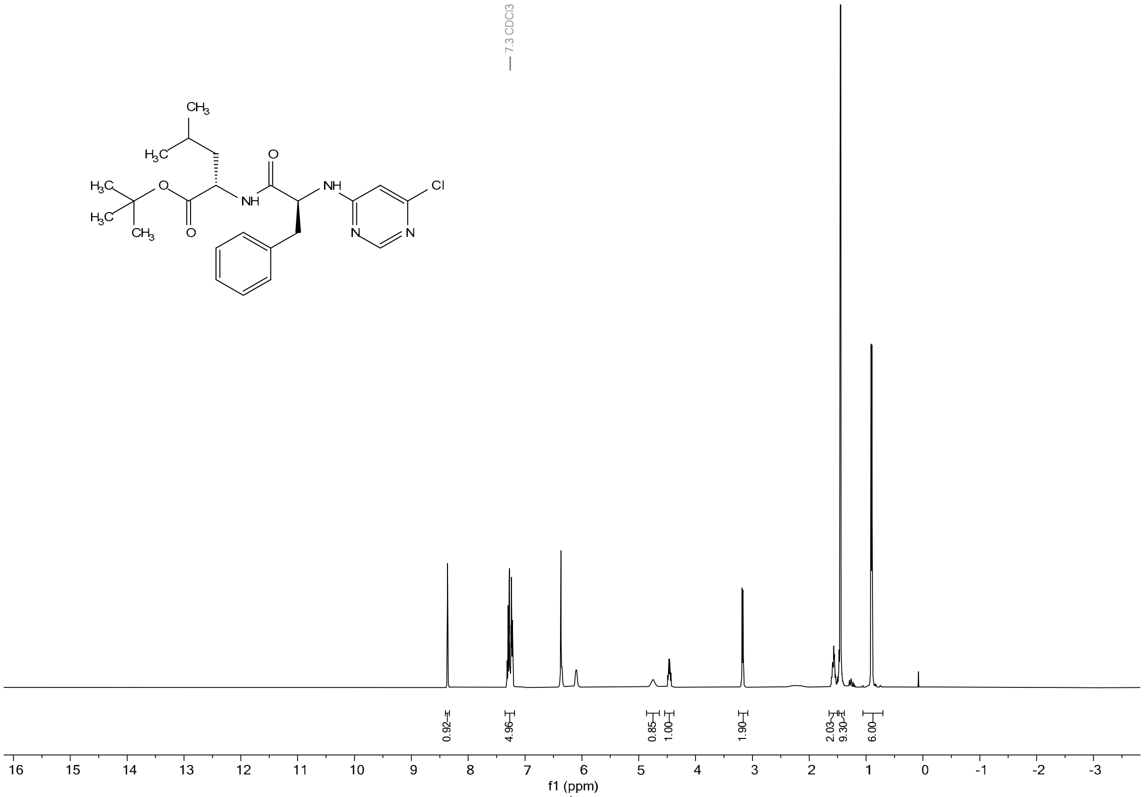

Spectrum 5: ^{1}H-NMR of **24**

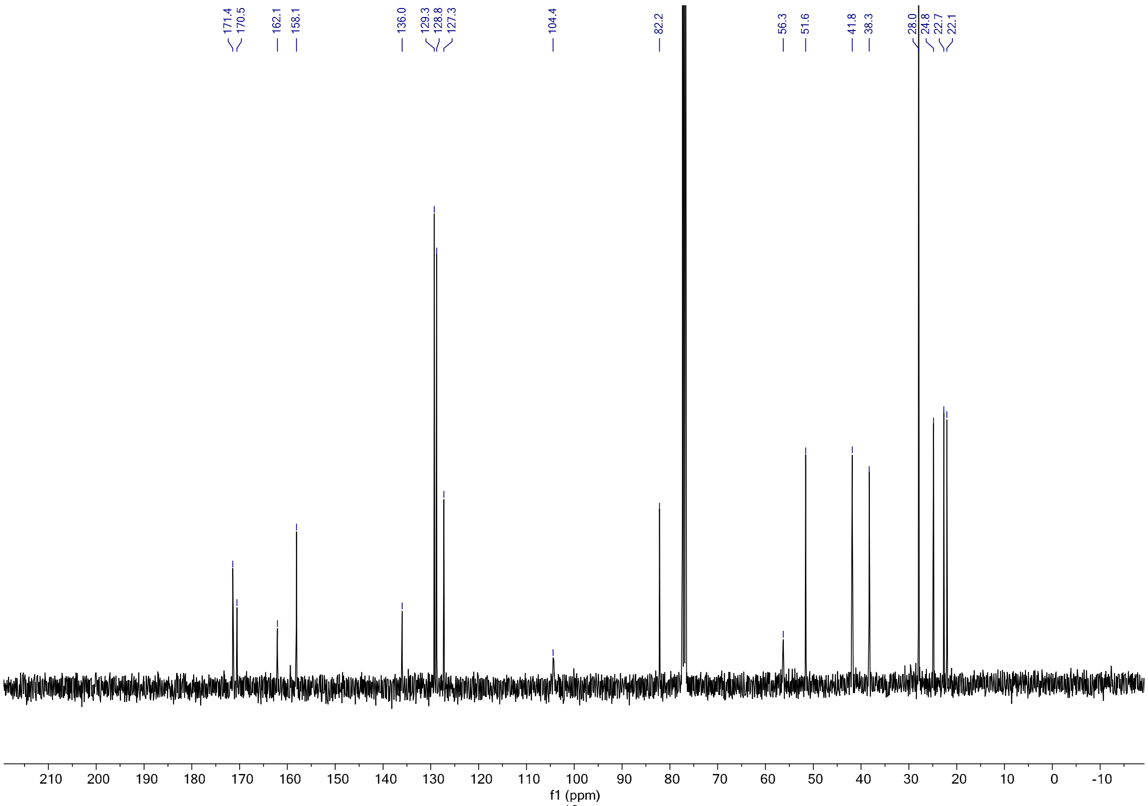

Spectrum 6: ^{13}C-NMR of **24**

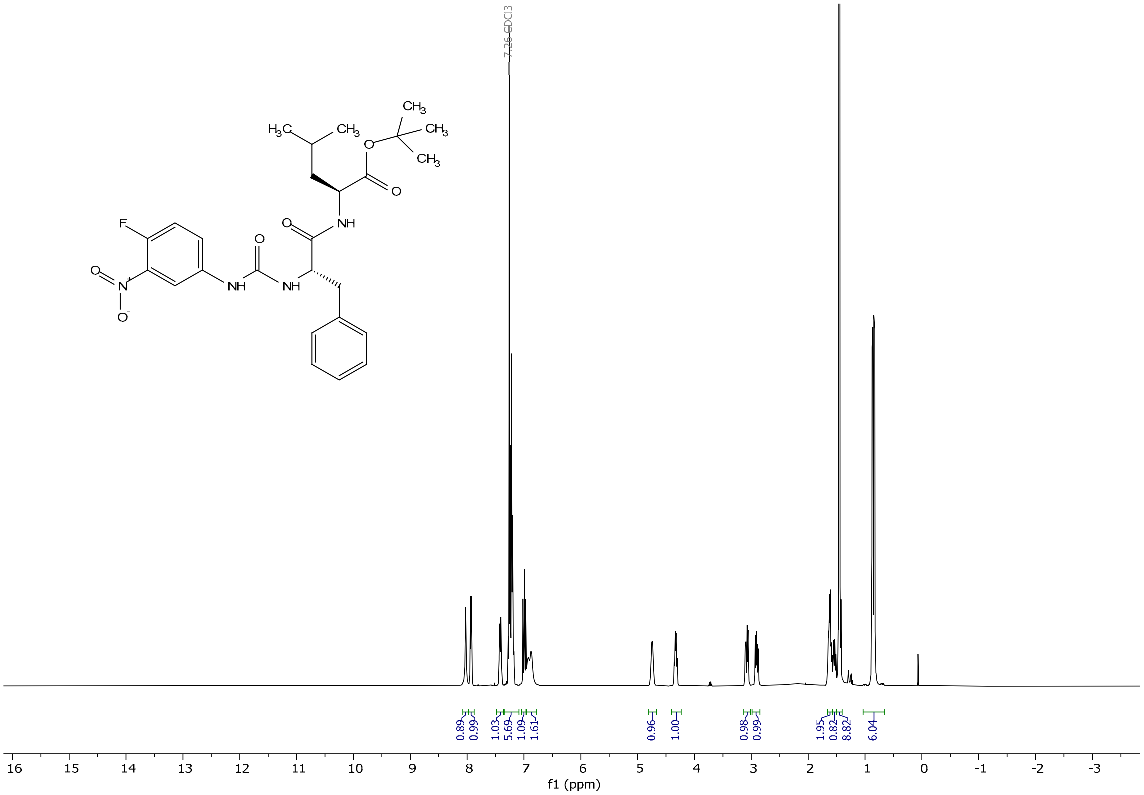

Spectrum 7: ^{1}H-NMR of **25**

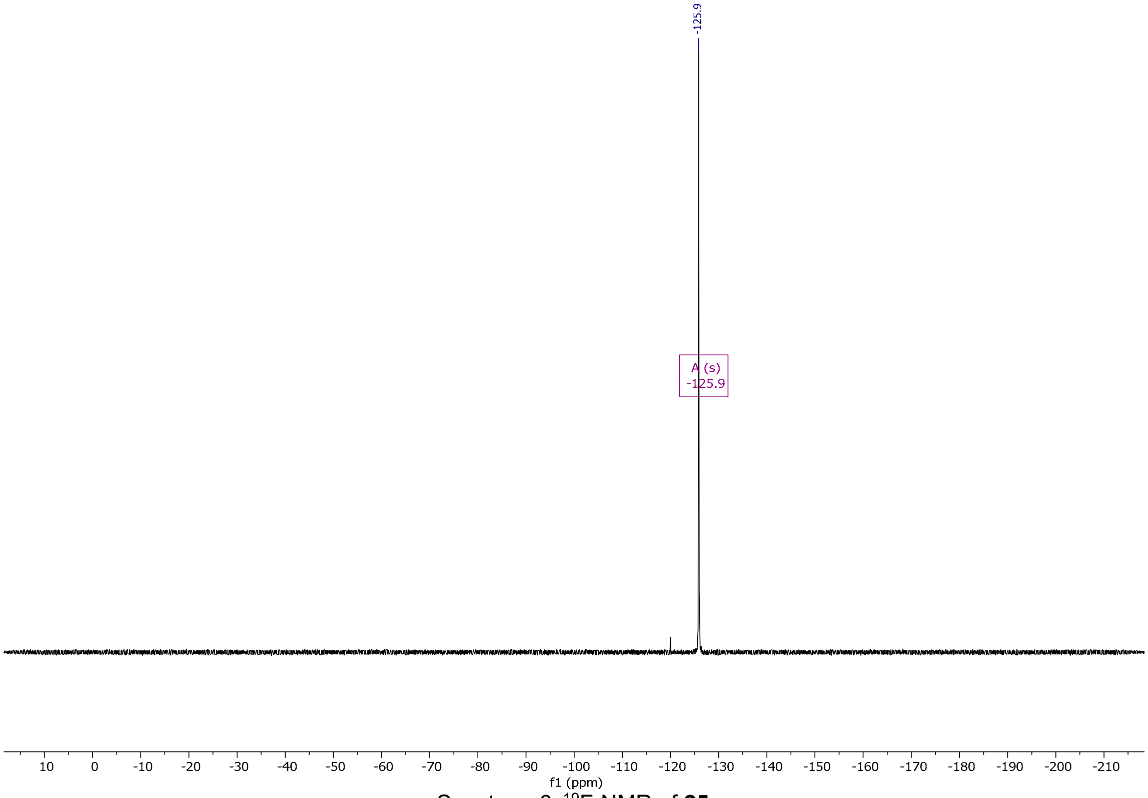

Spectrum 8: ^{19}F-NMR of **25**

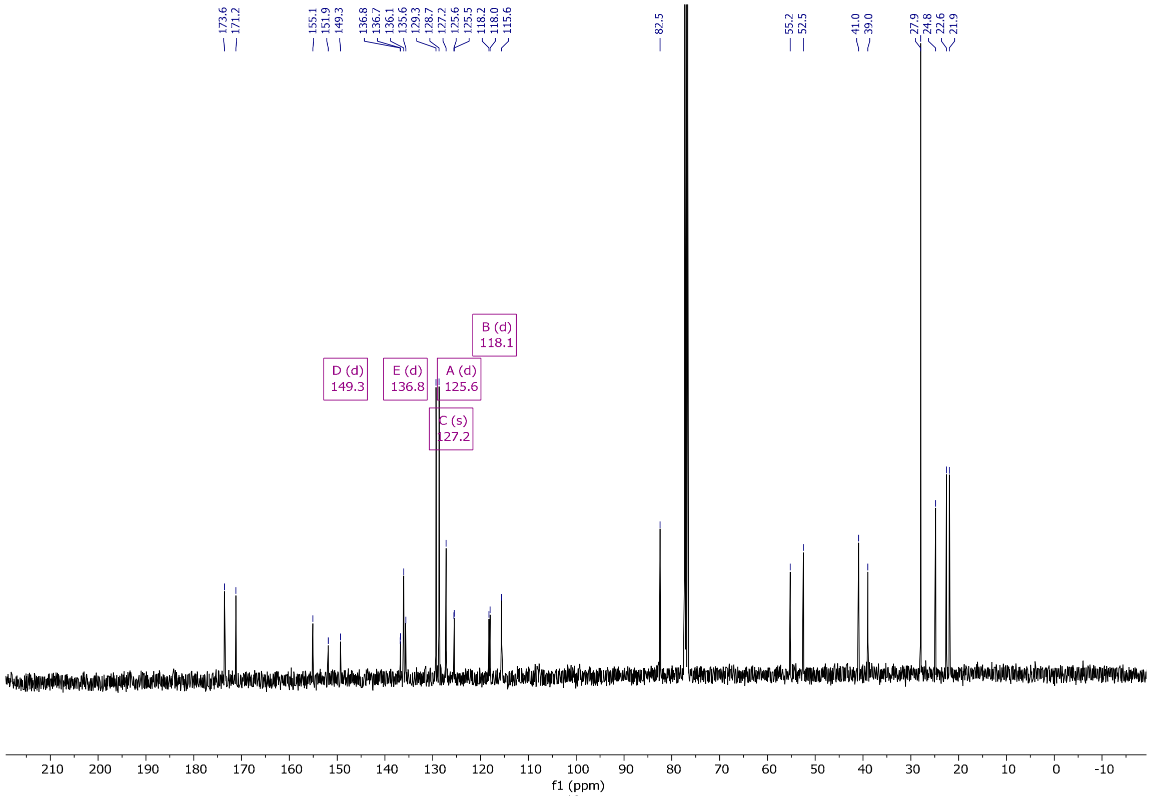

Spectrum 9: ^{13}C-NMR of **25**

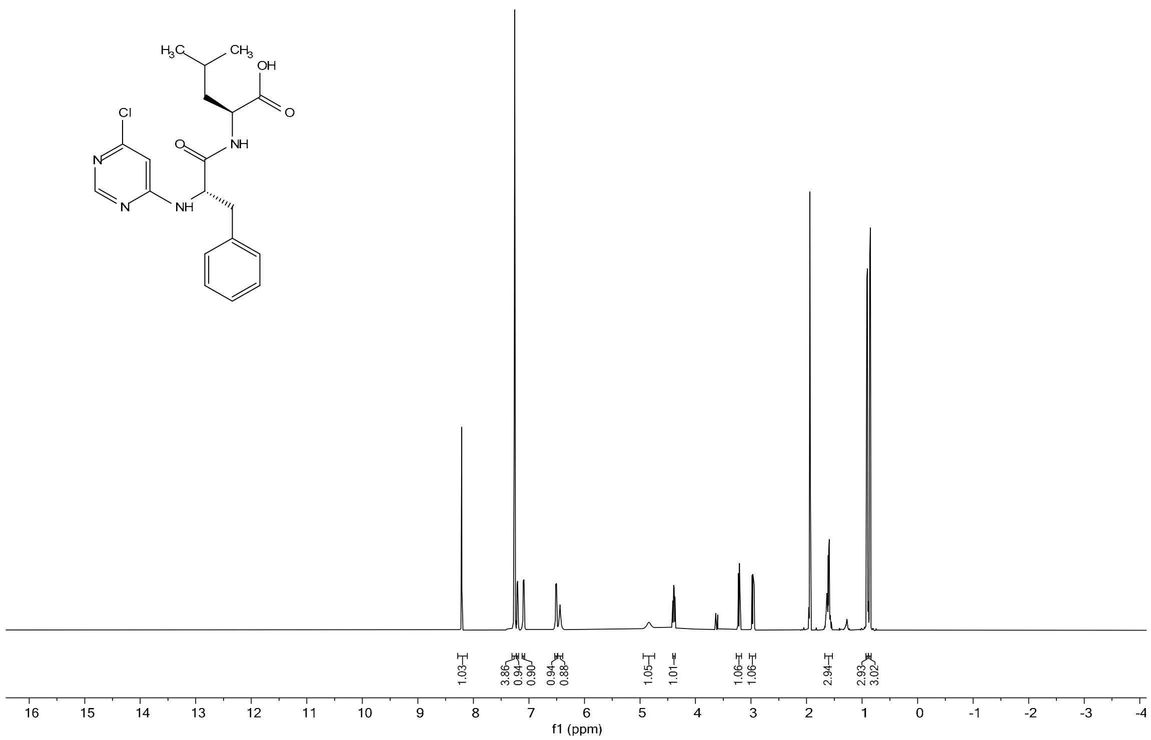

Spectrum 10: ^{1}H-NMR of **6**

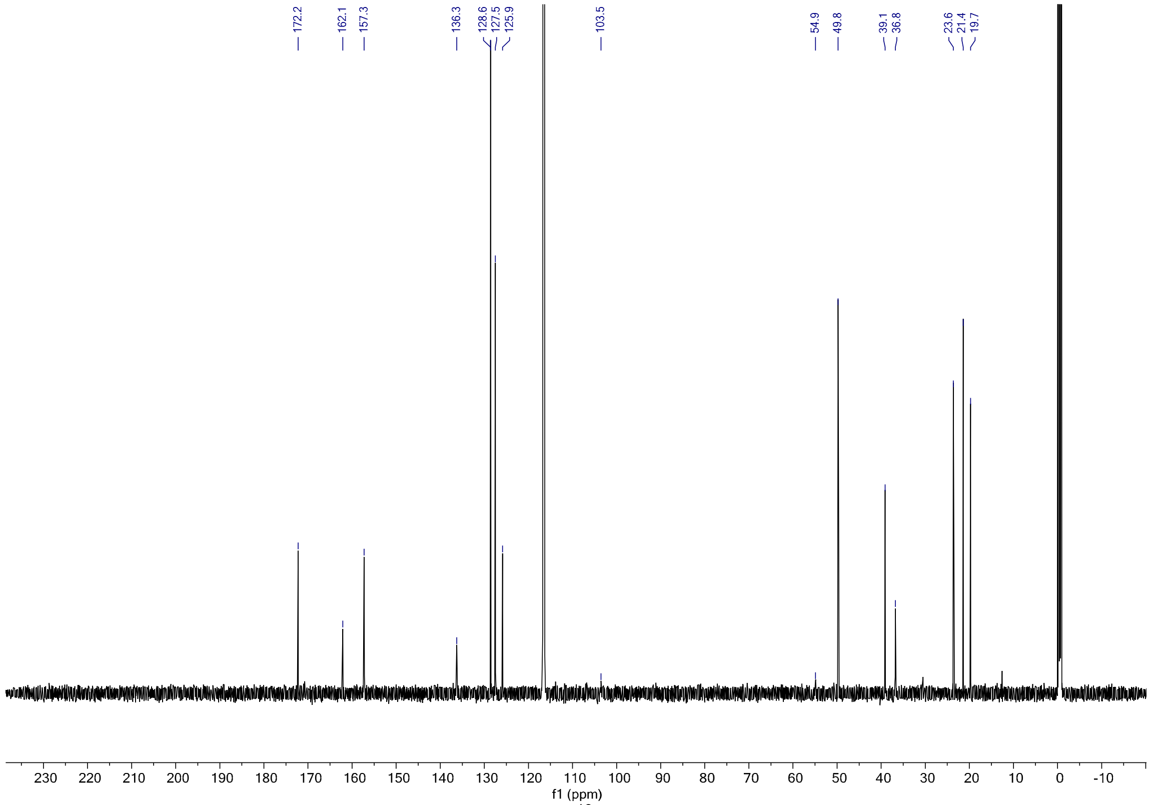

Spectrum 11: ^{13}C-NMR of **6**

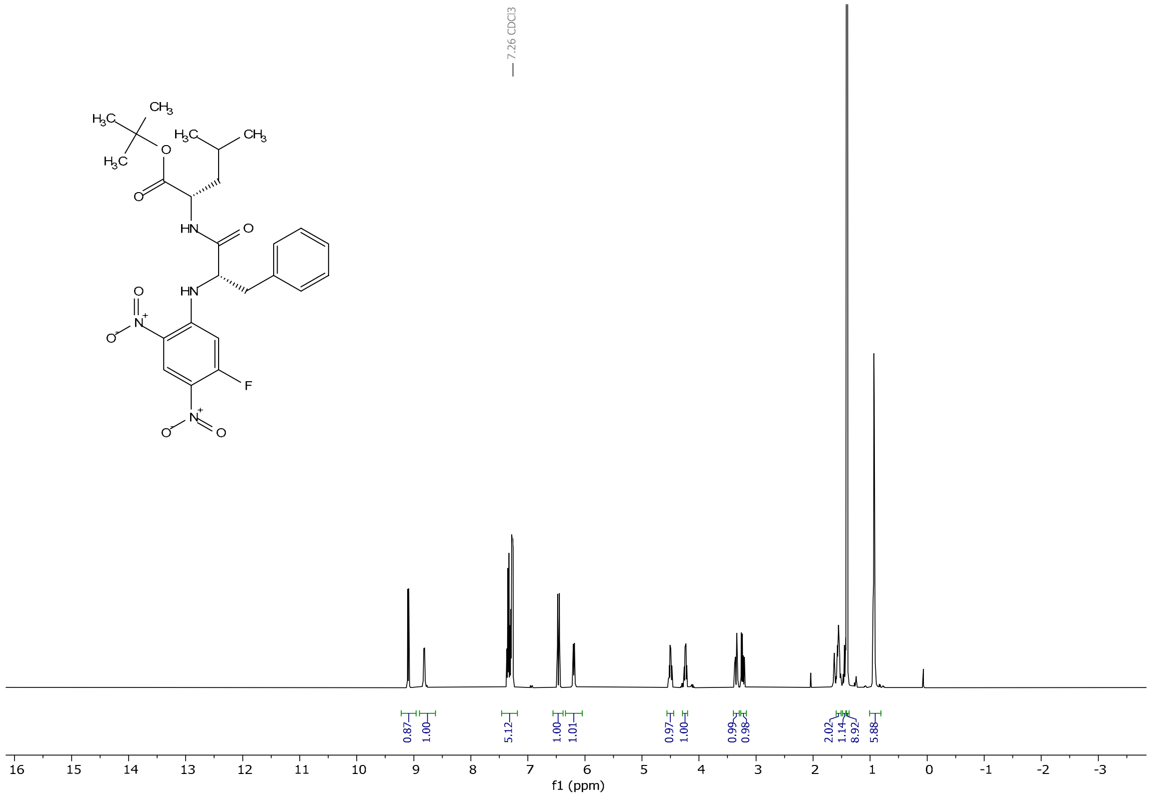

Spectrum 12: ^{1}H-NMR of **21**

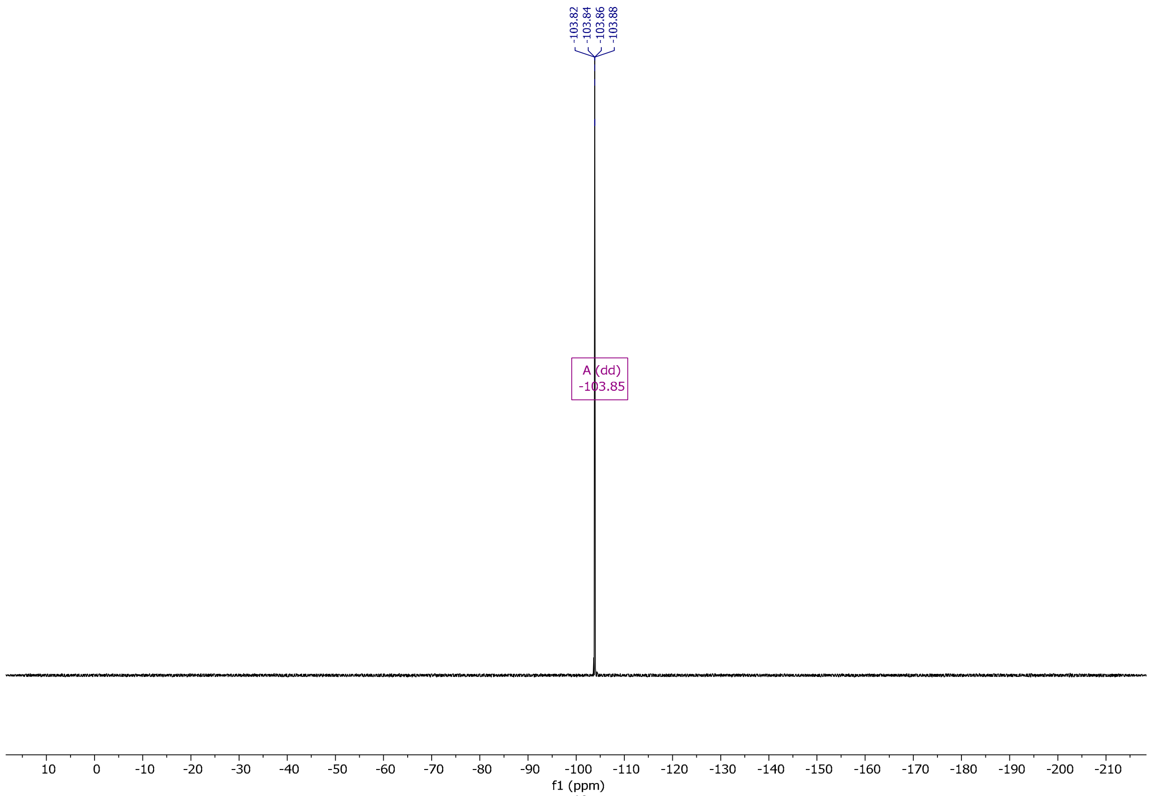

Spectrum 13: ^{19}F-NMR of **21**

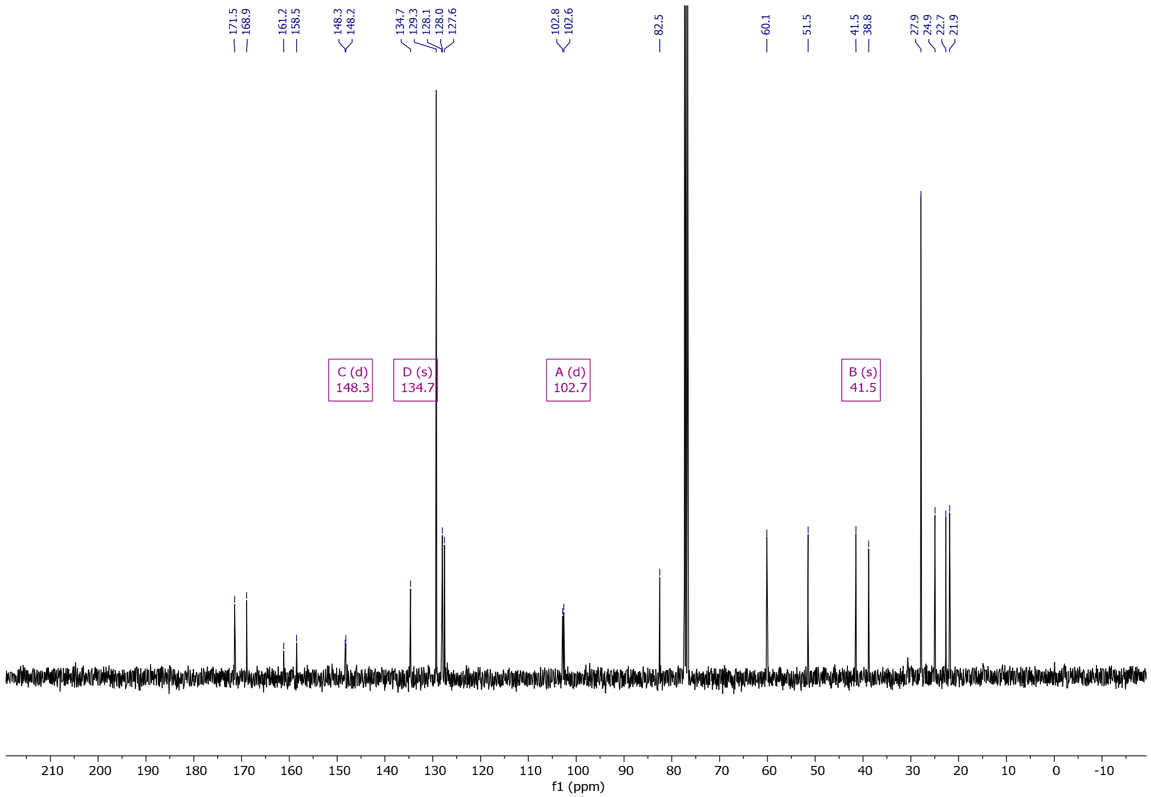

Spectrum 14: ^{13}C-NMR of **21**

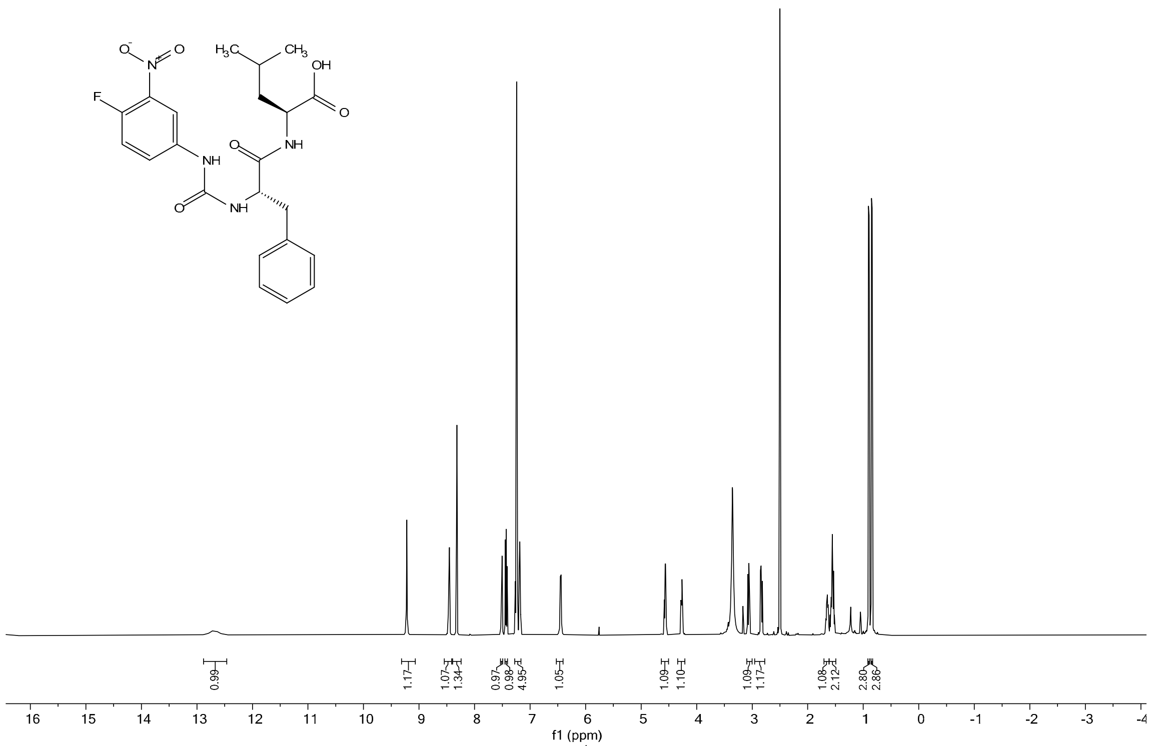

Spectrum 15: ^{1}H-NMR of **7**

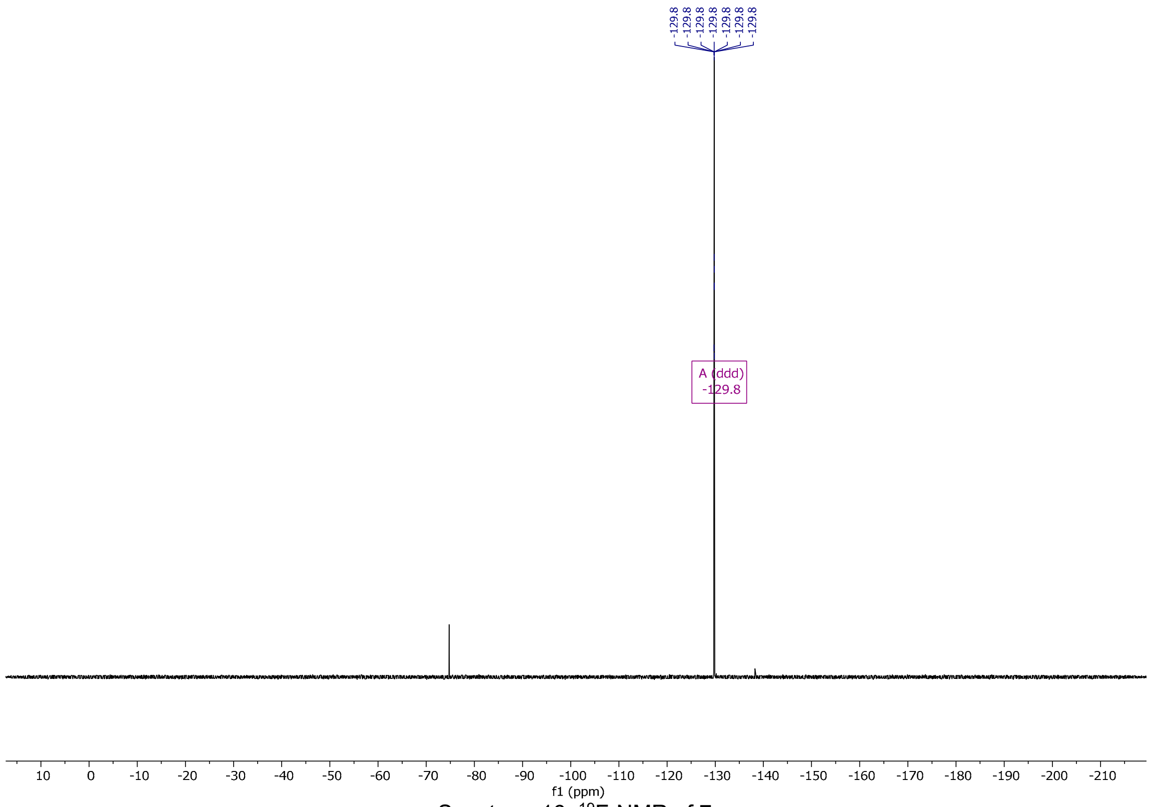

Spectrum 16: ^{19}F-NMR of **7**

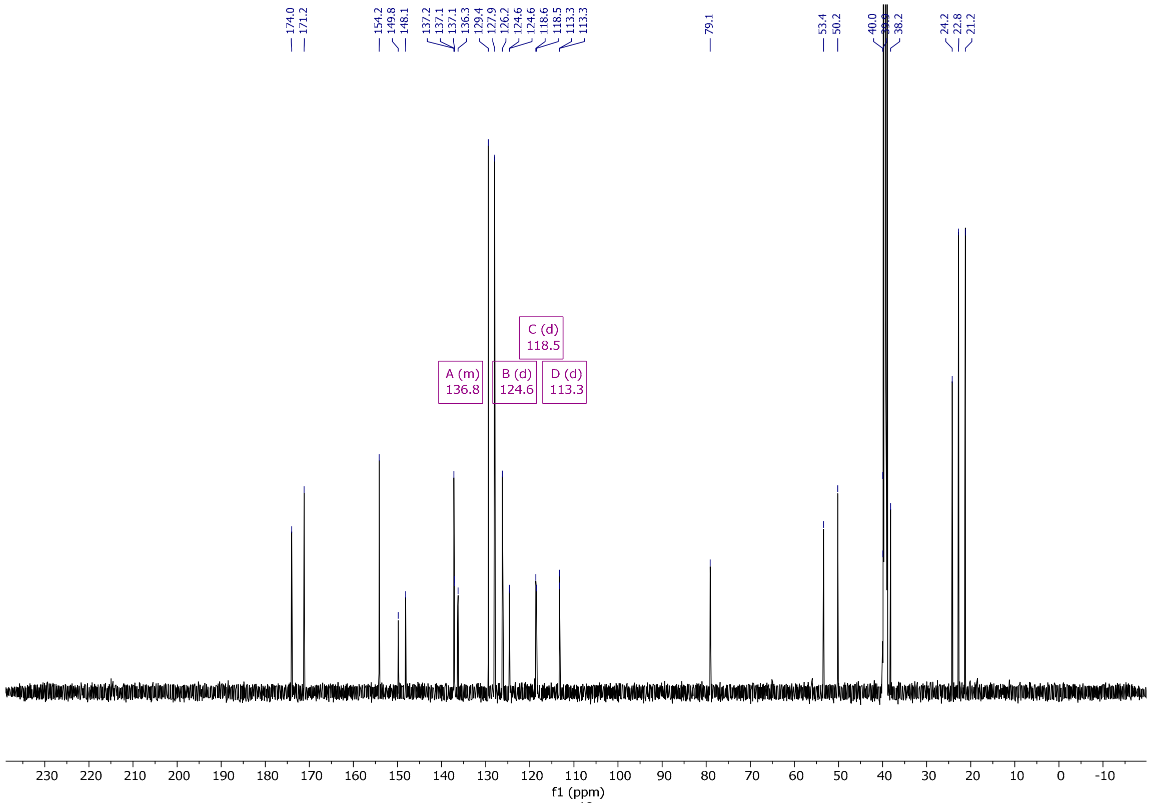

Spectrum 17: ^{13}C-NMR of **7**

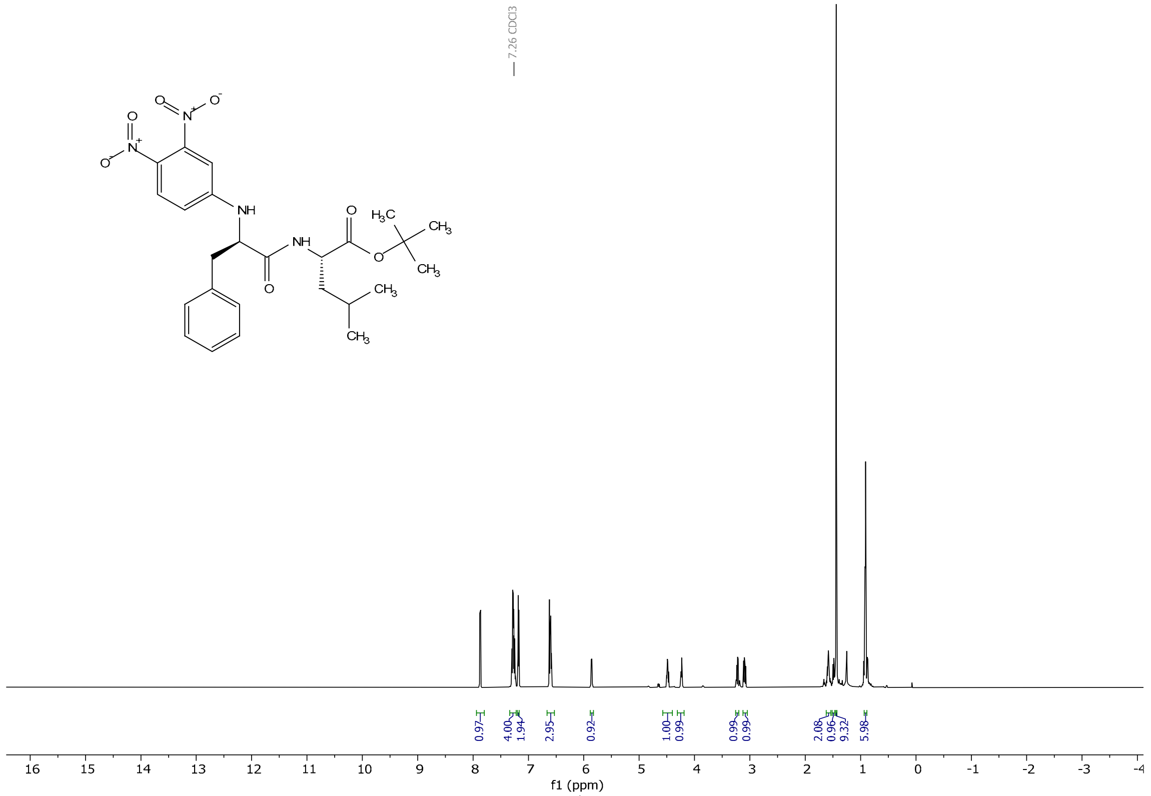

Spectrum 18: ^{1}H-NMR of **20**

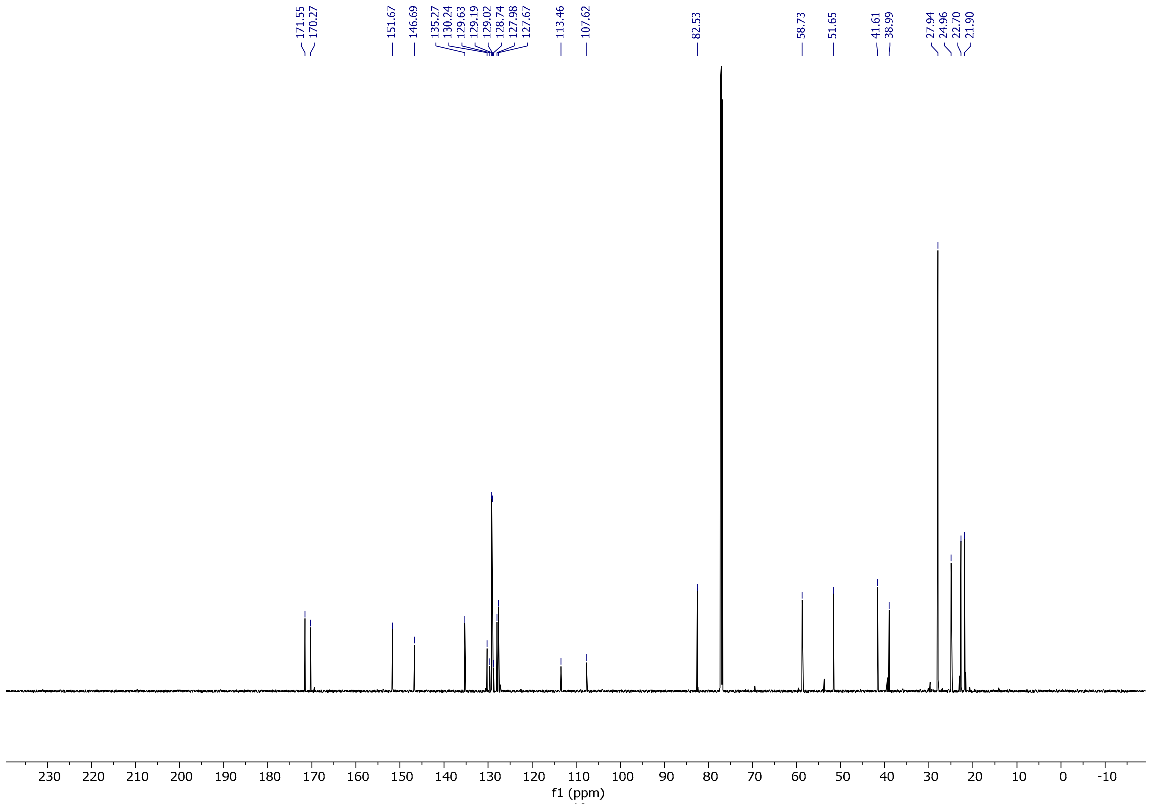

Spectrum 19: ^{13}C-NMR of **20**

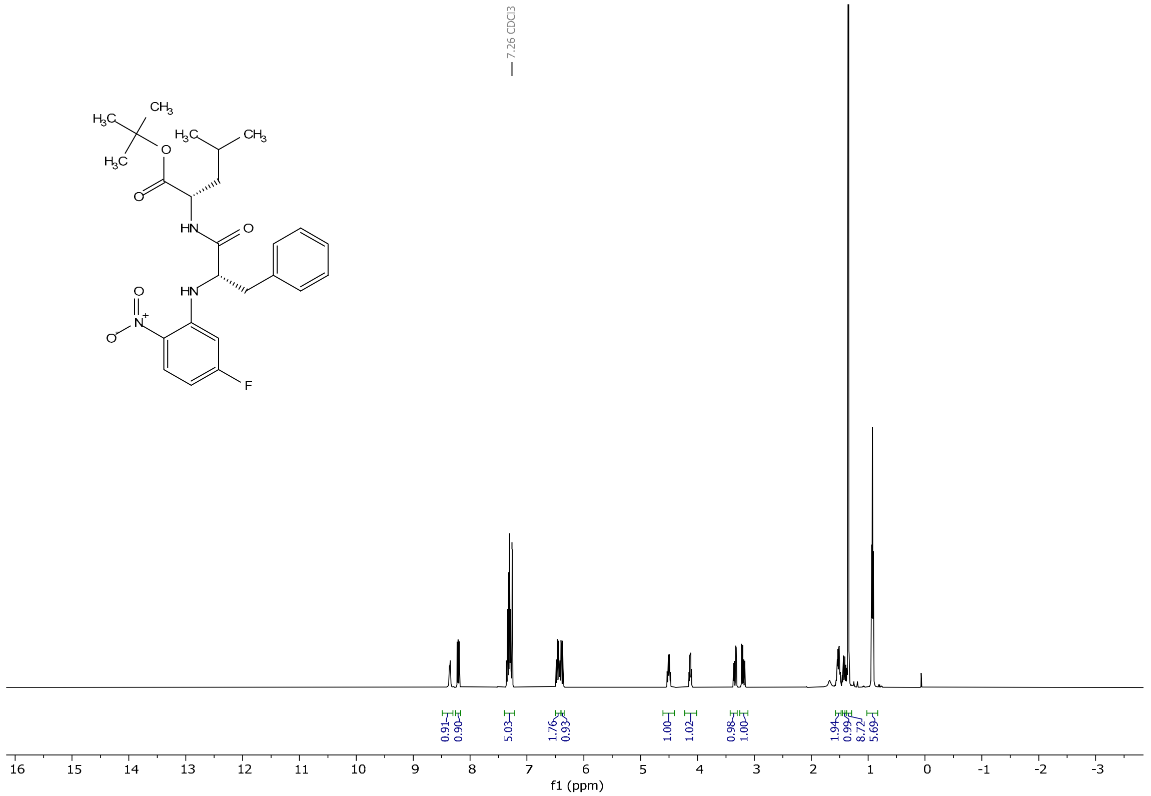

Spectrum 20: ^{1}H-NMR of **23**

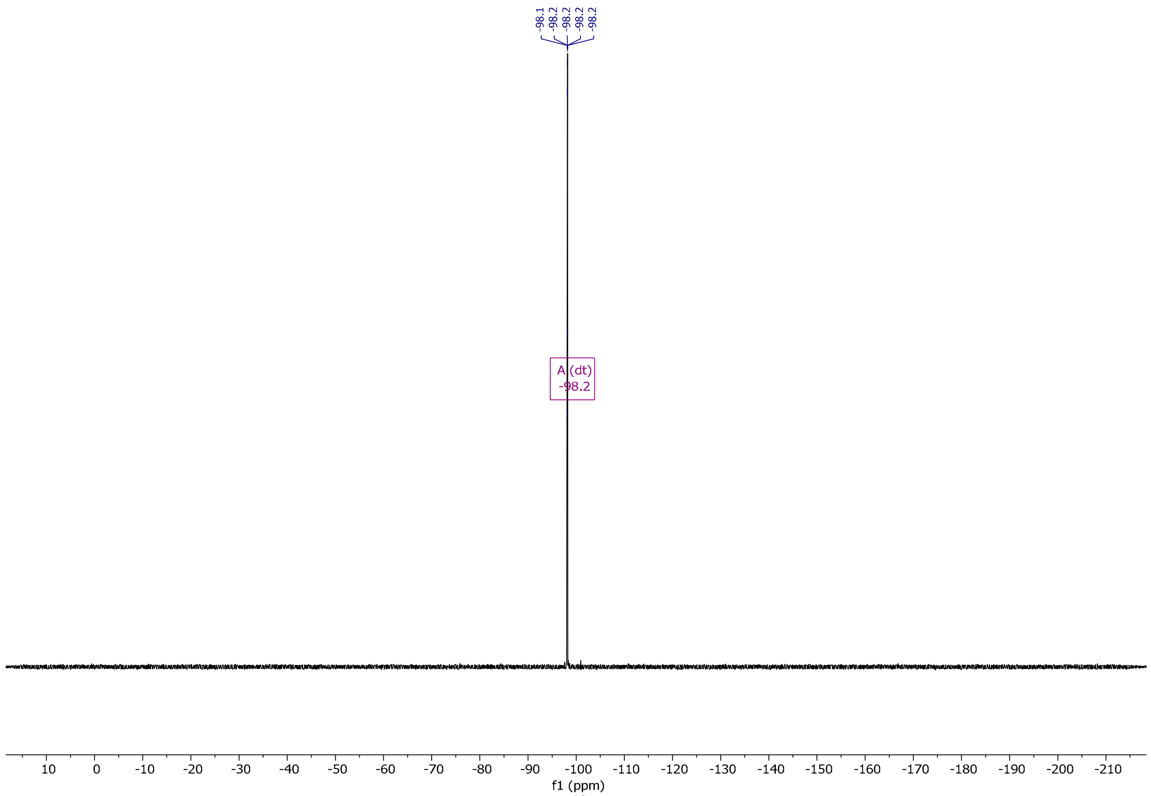

Spectrum 21: ^{19}F-NMR of **23**

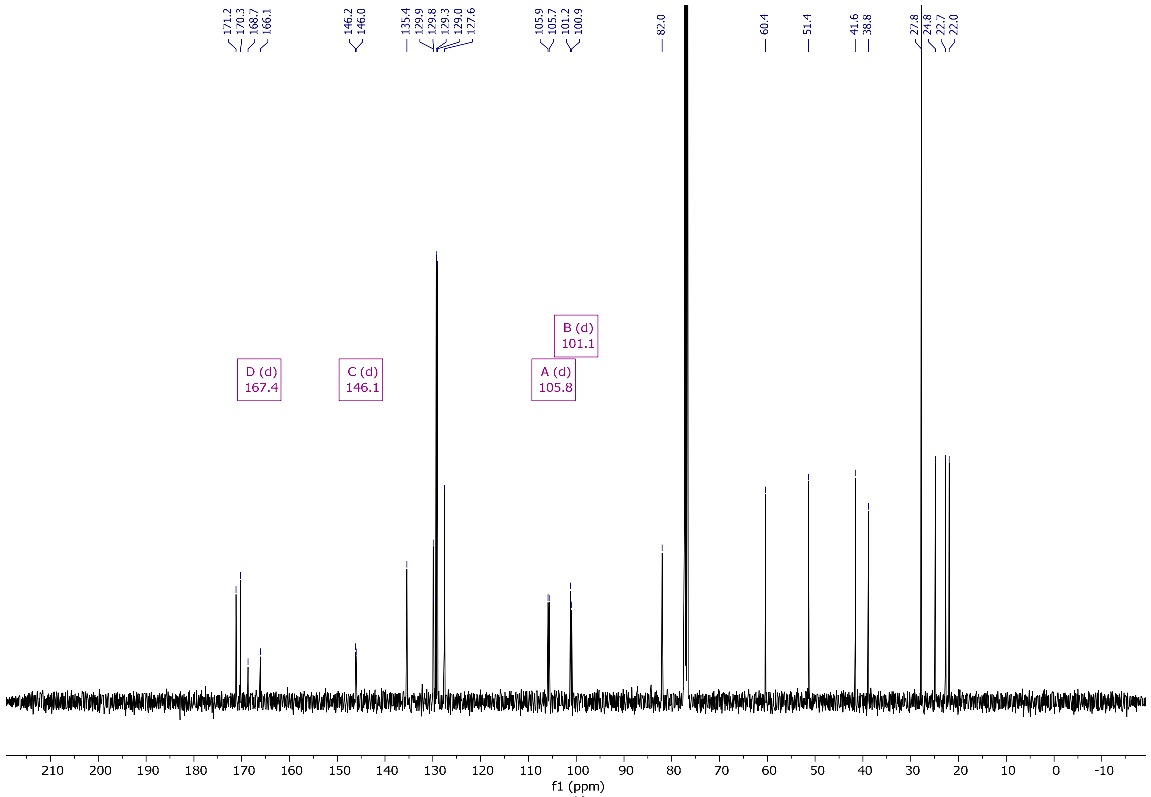

Spectrum 22: ^{13}C-NMR of **23**

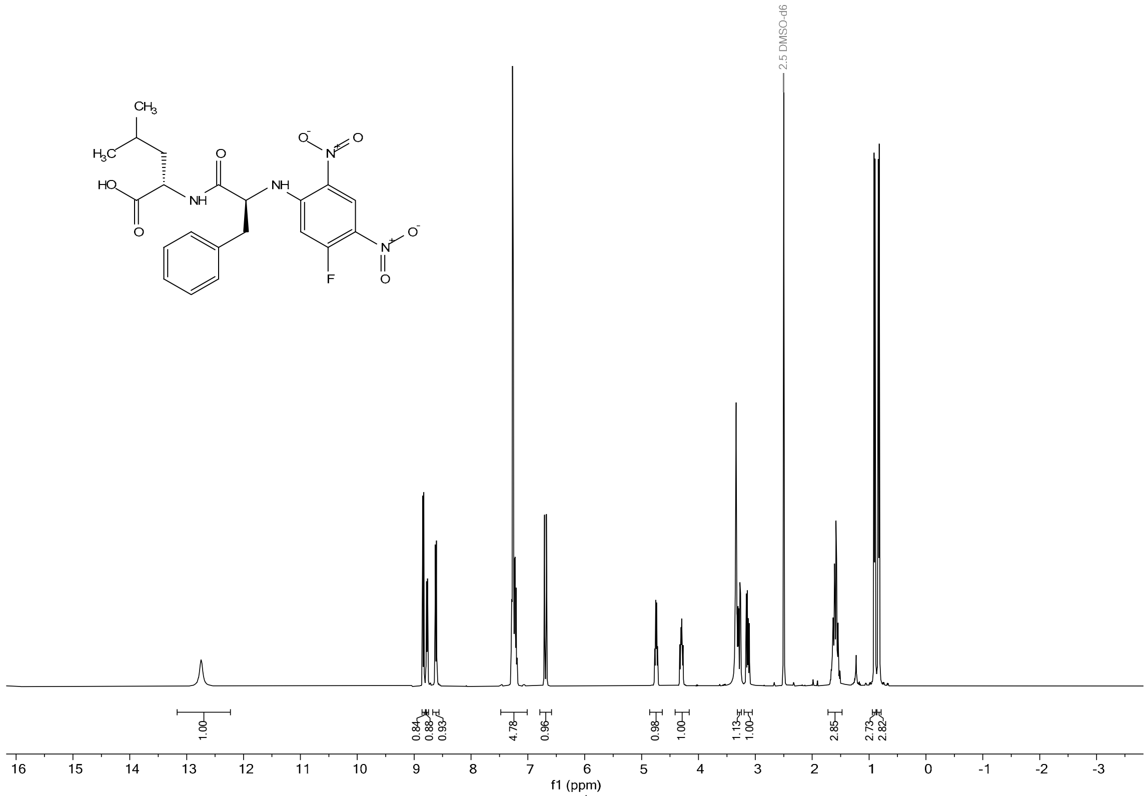

Spectrum 23: ^{1}H-NMR of **3**

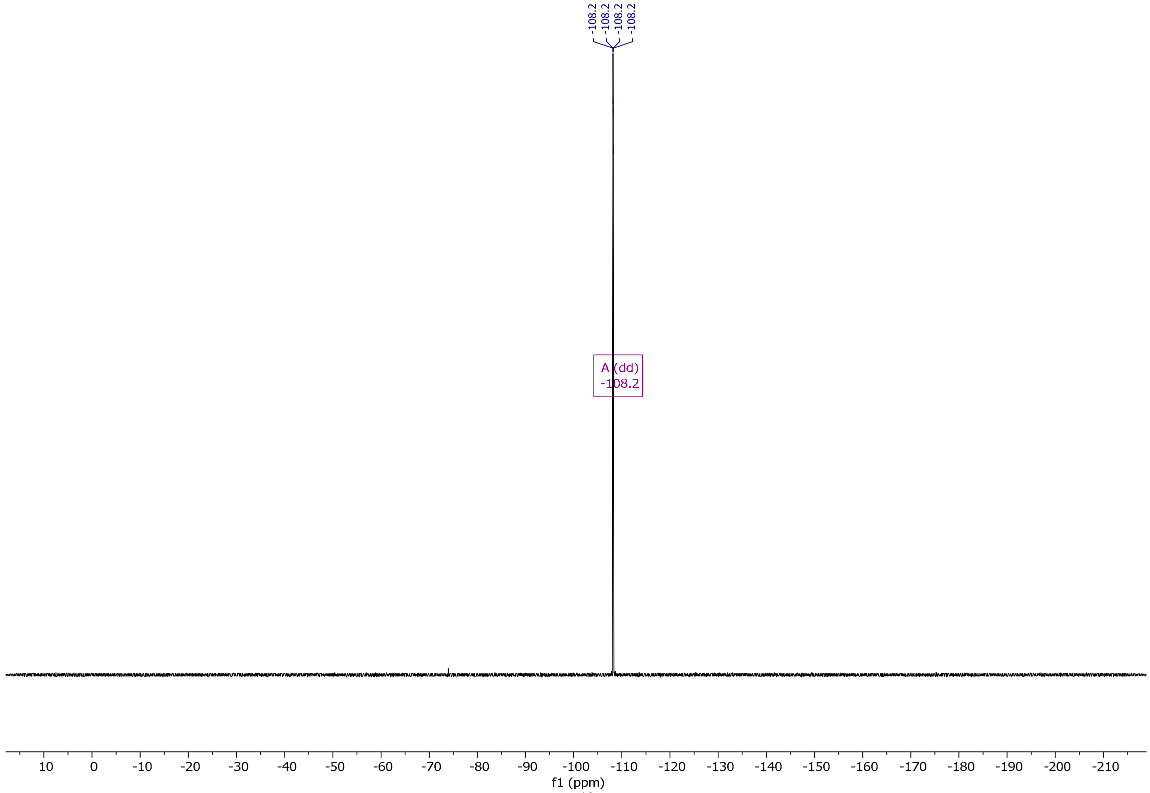

Spectrum 24: ^{19}F-NMR of **3**

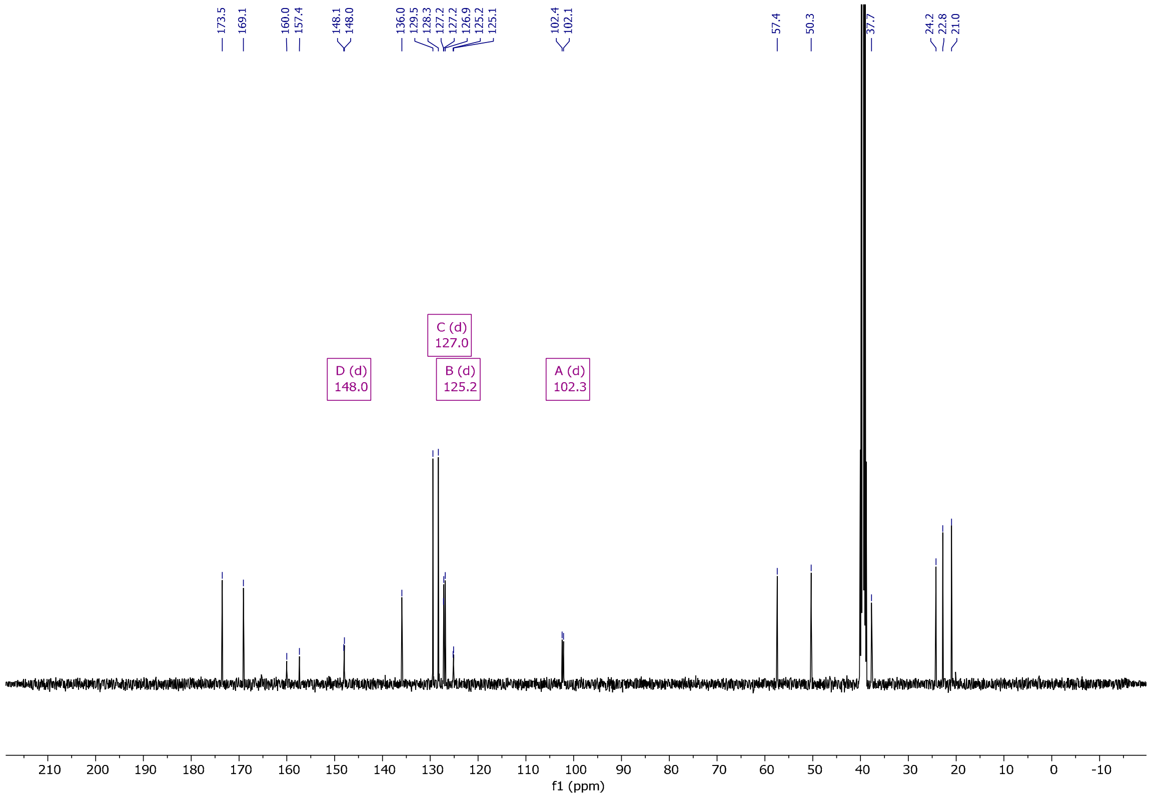

Spectrum 25: ^{13}C-NMR of **3**

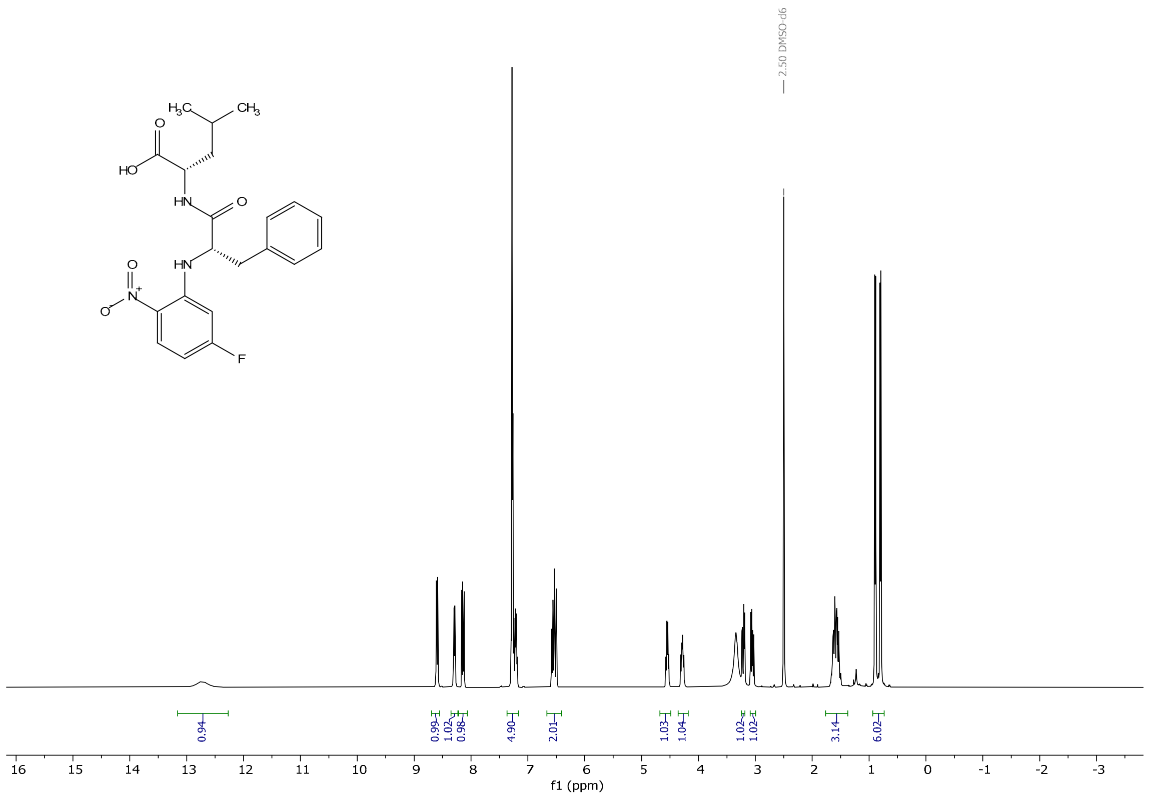

Spectrum 26: ^{1}H-NMR of **5**

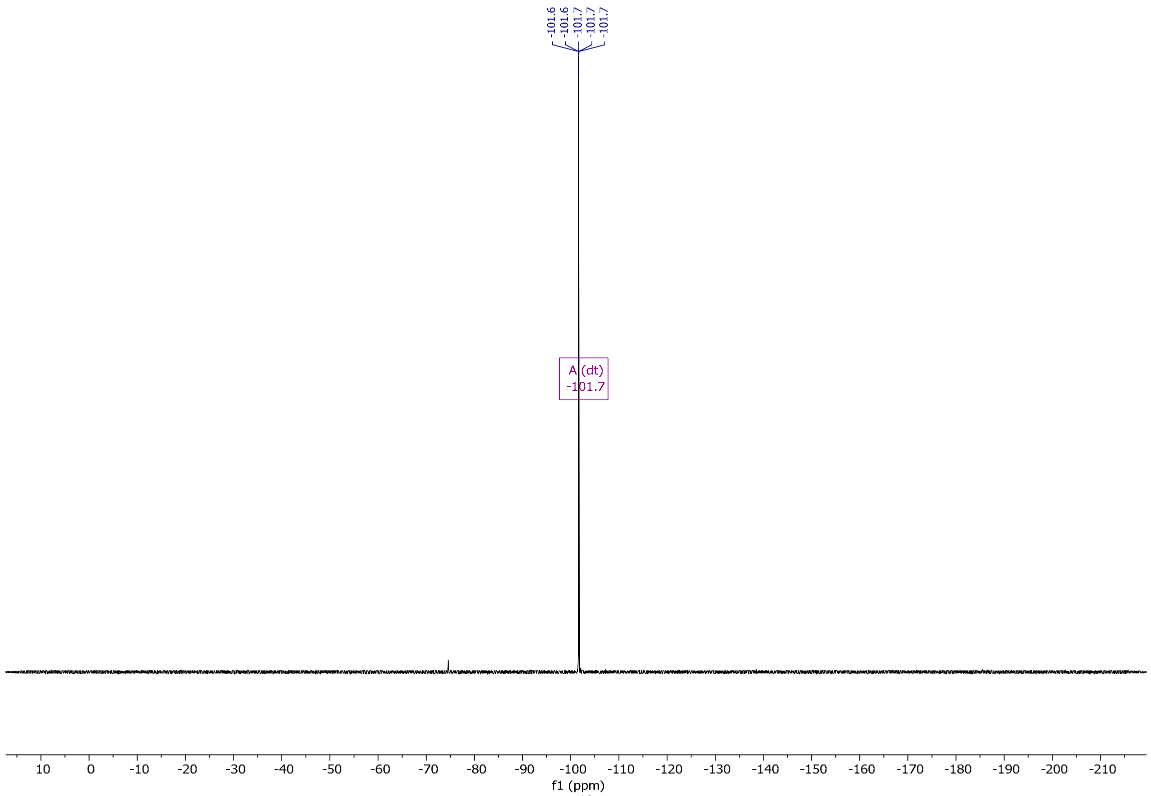

Spectrum 27: ^{19}F-NMR of **5**

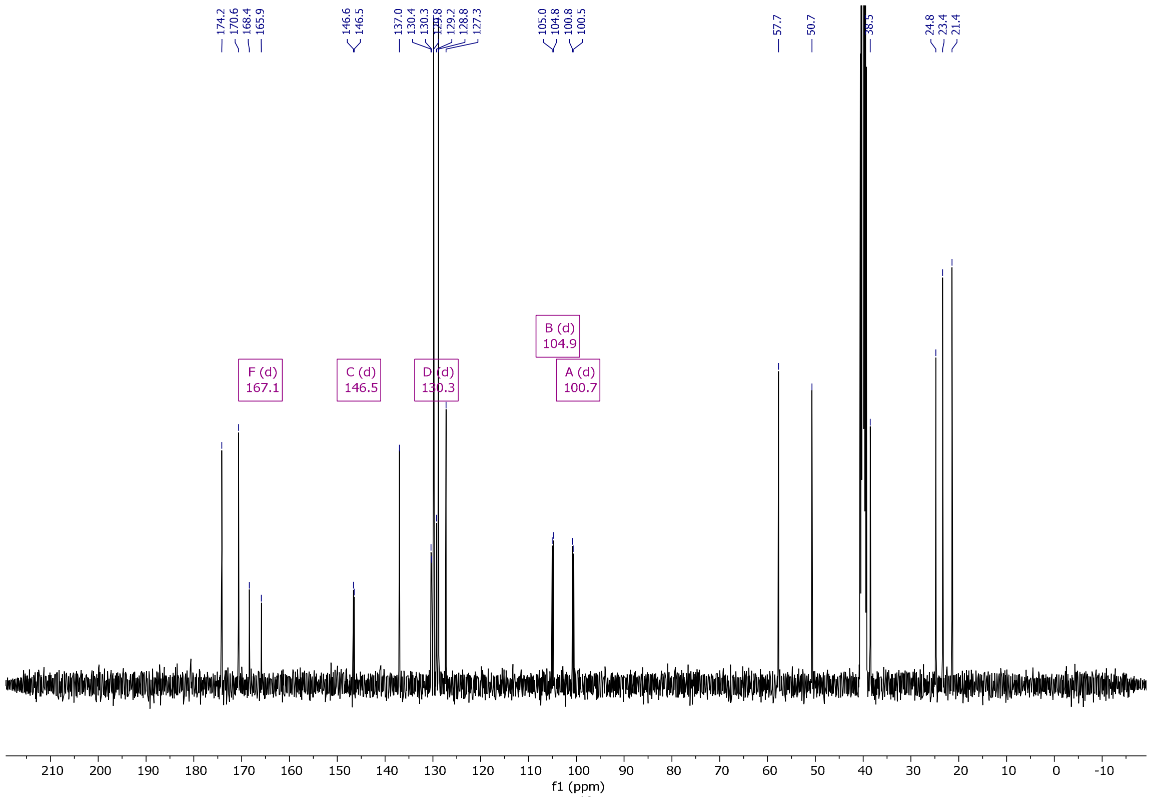

Spectrum 28: ^{13}C-NMR of **5**

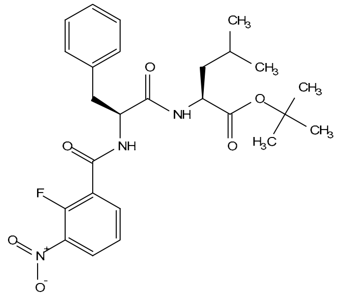

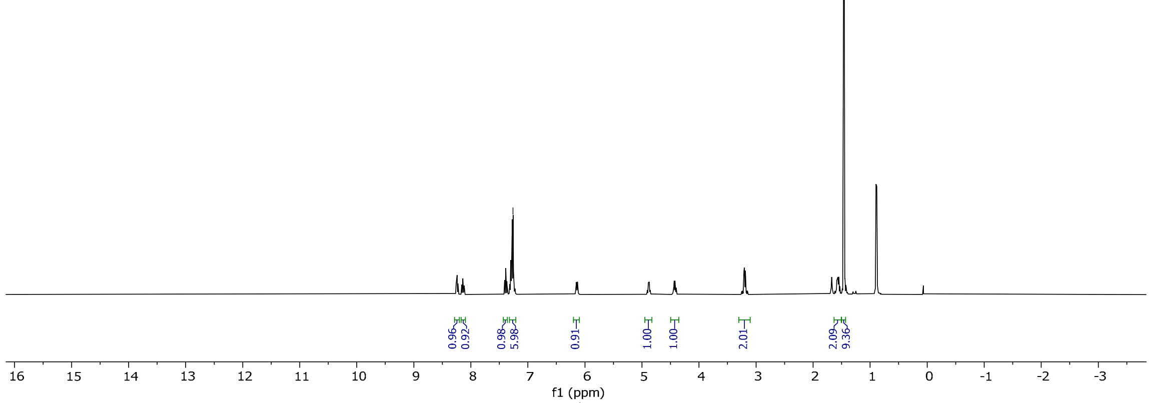

Spectrum 29: ^{1}H-NMR of **28**

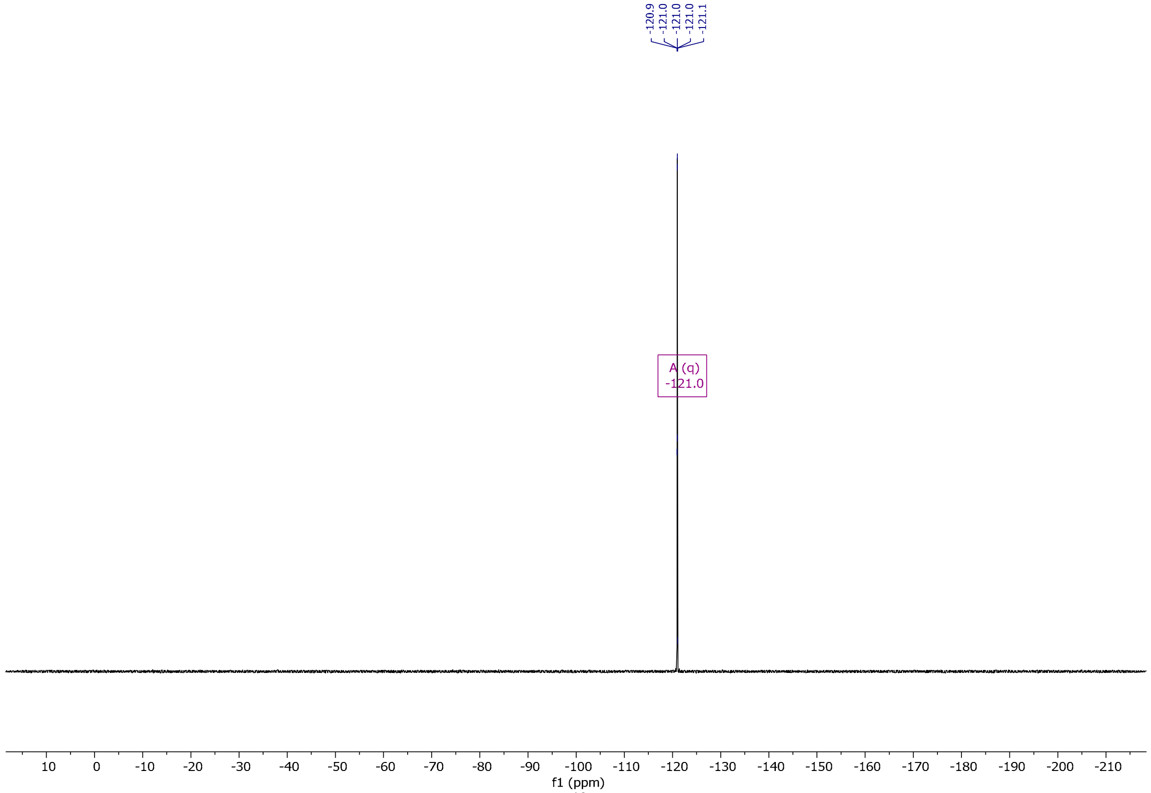

Spectrum 30: ^{19}F-NMR of **28**

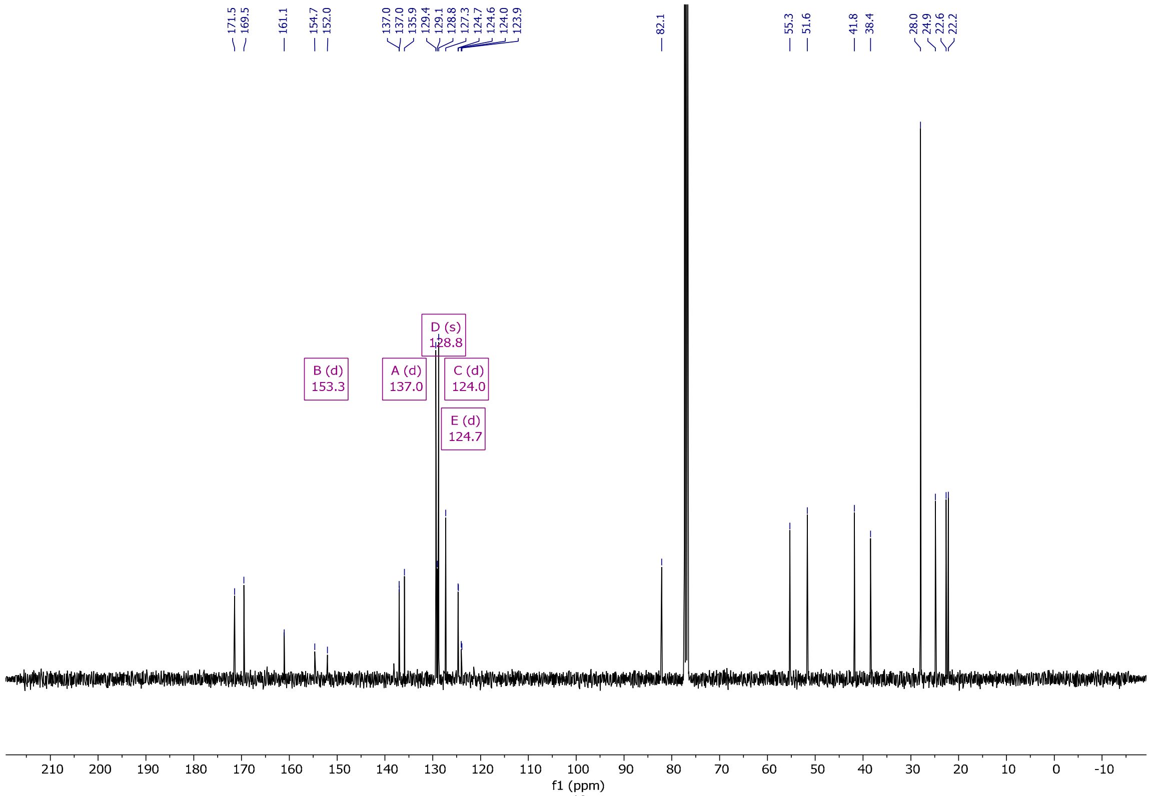

Spectrum 31: ^{13}C-NMR of **28**

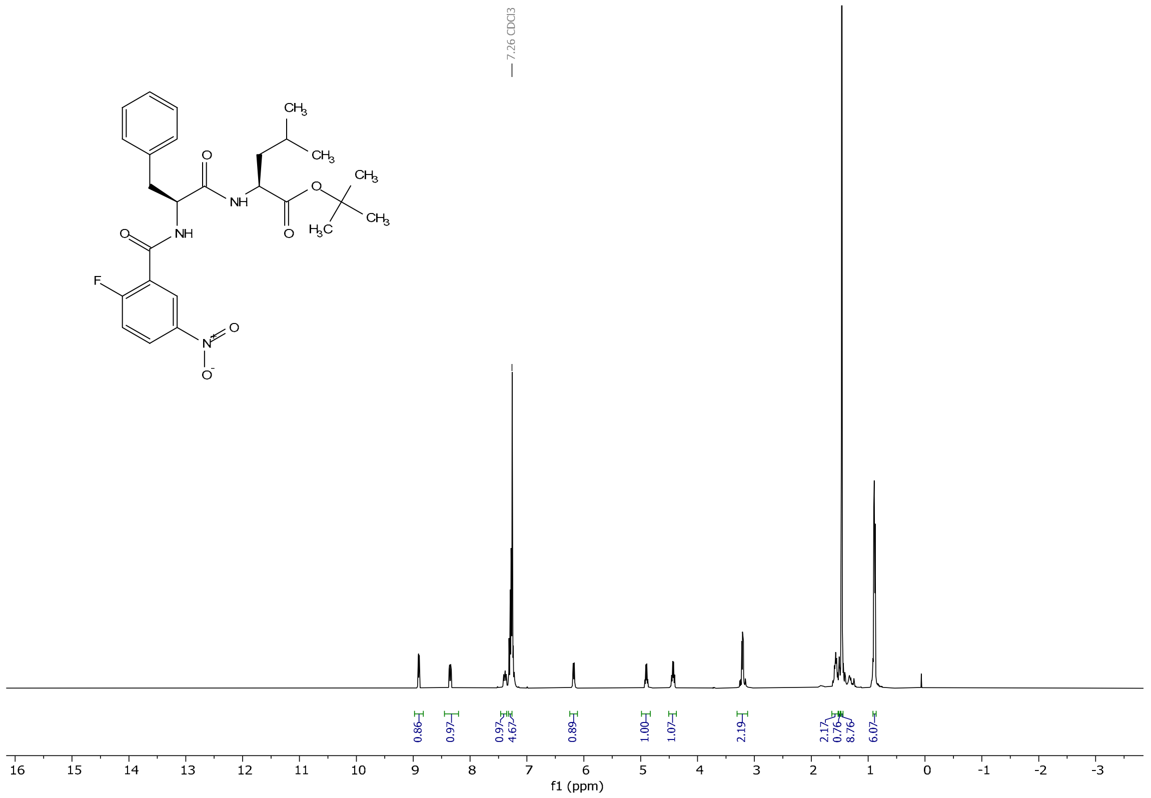

Spectrum 32: ^{1}H-NMR of **29**

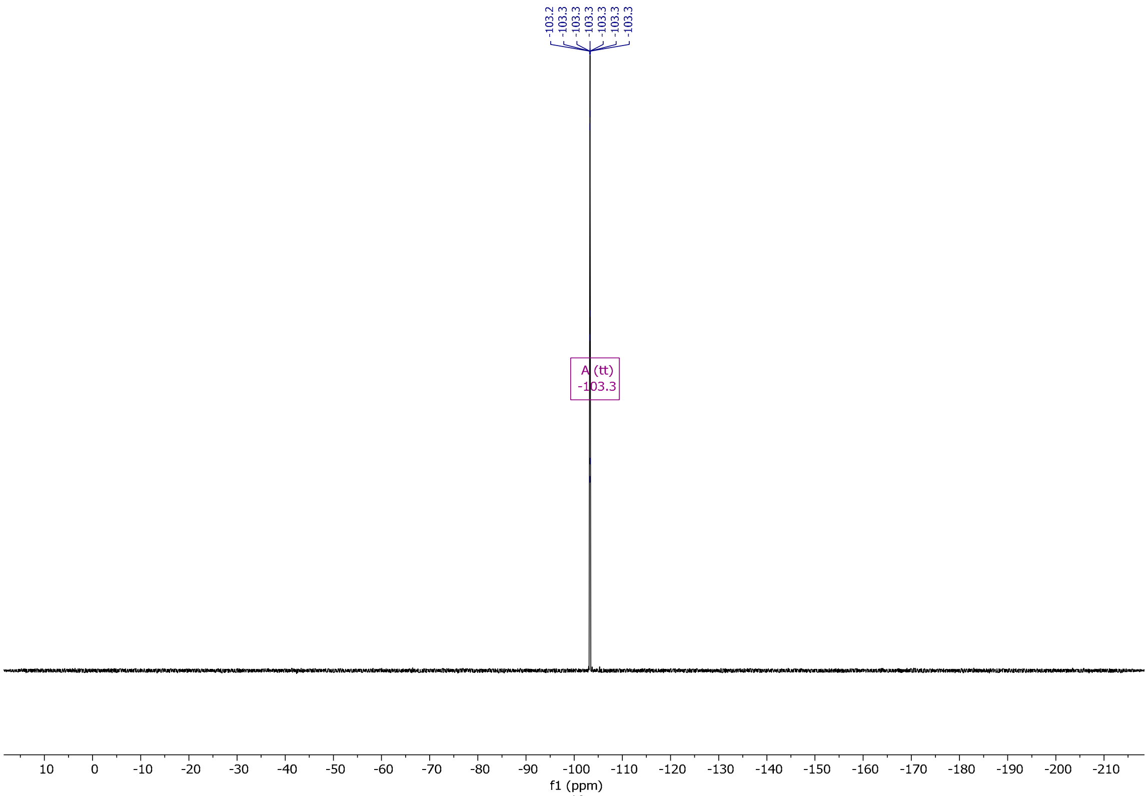

Spectrum 33: ^{19}F-NMR of **29**

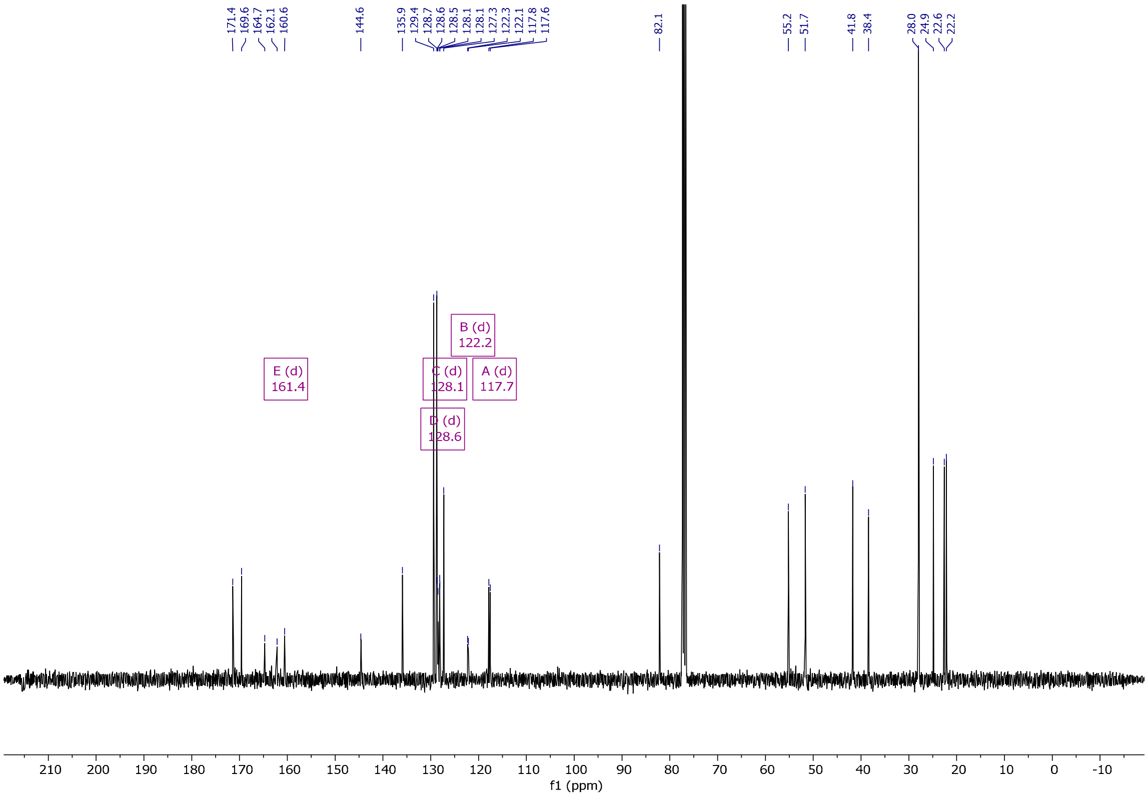

Spectrum 34: ^{13}C-NMR of **29**

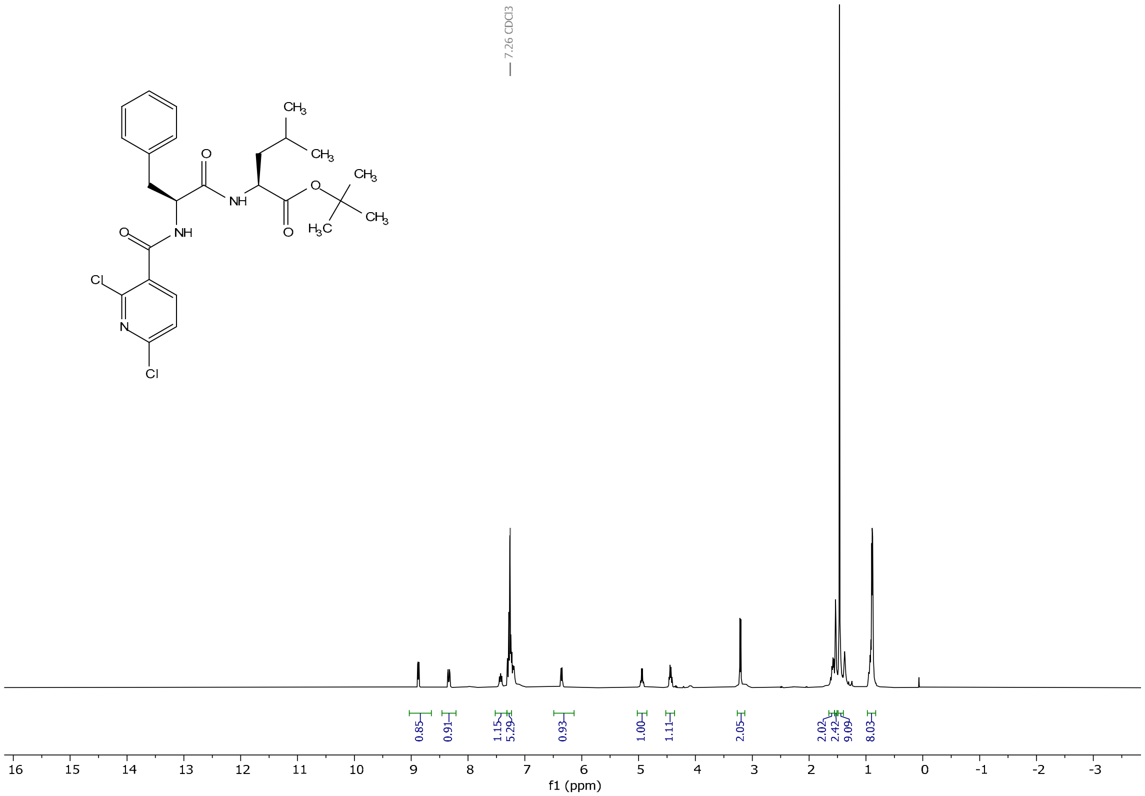

Spectrum 35: ^{1}H-NMR of **26**

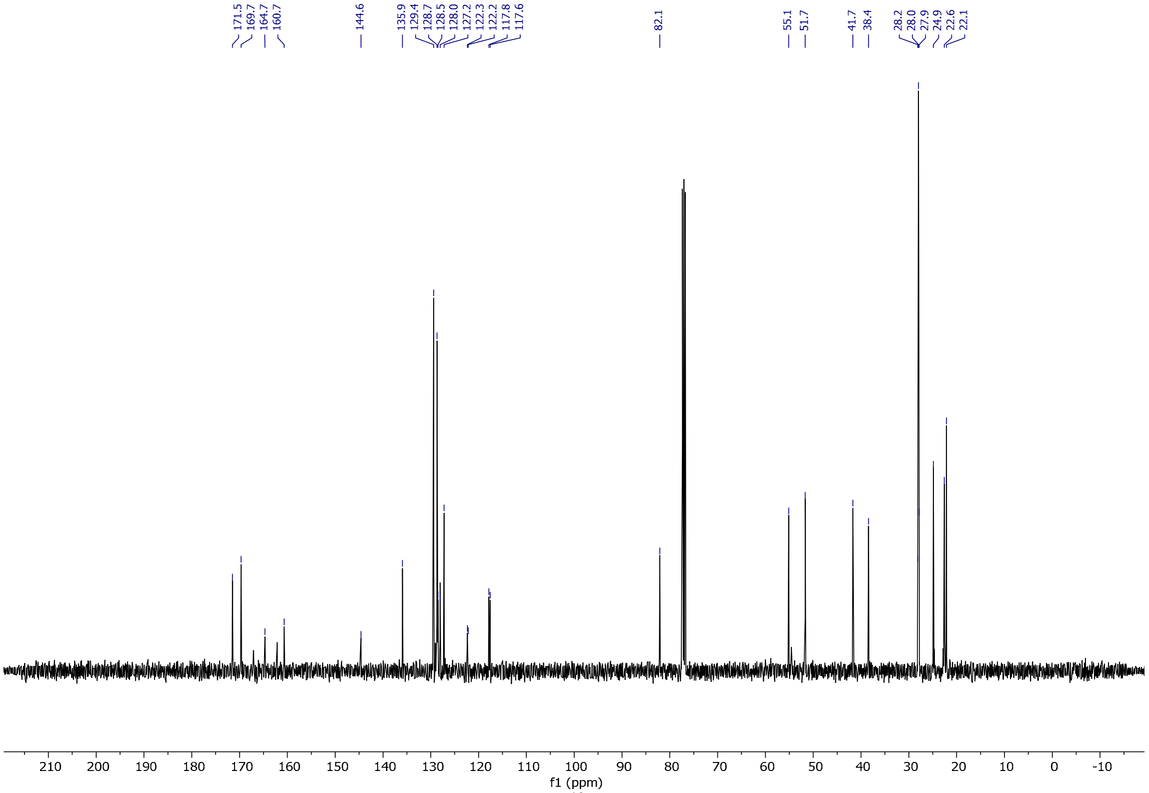

Spectrum 36: ^{13}C-NMR of **26**

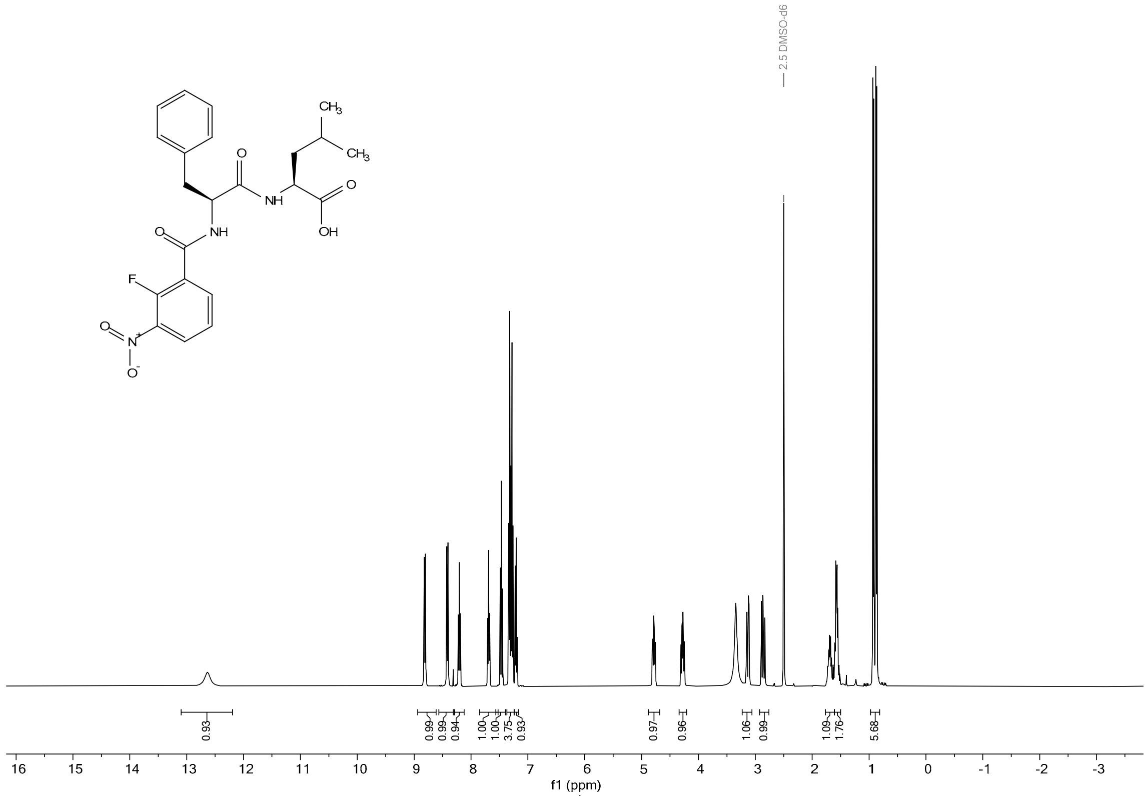

Spectrum 37: ^{1}H-NMR of **10**

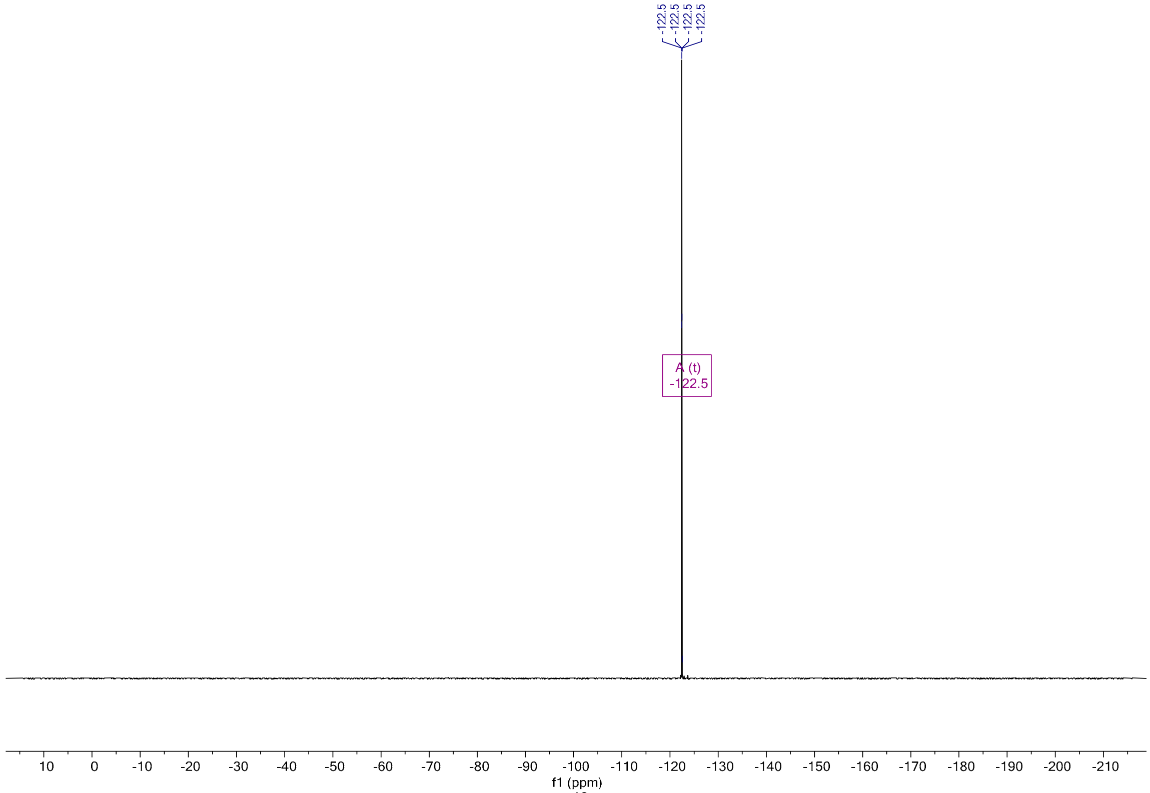

Spectrum 38: ^{19}F-NMR of **10**

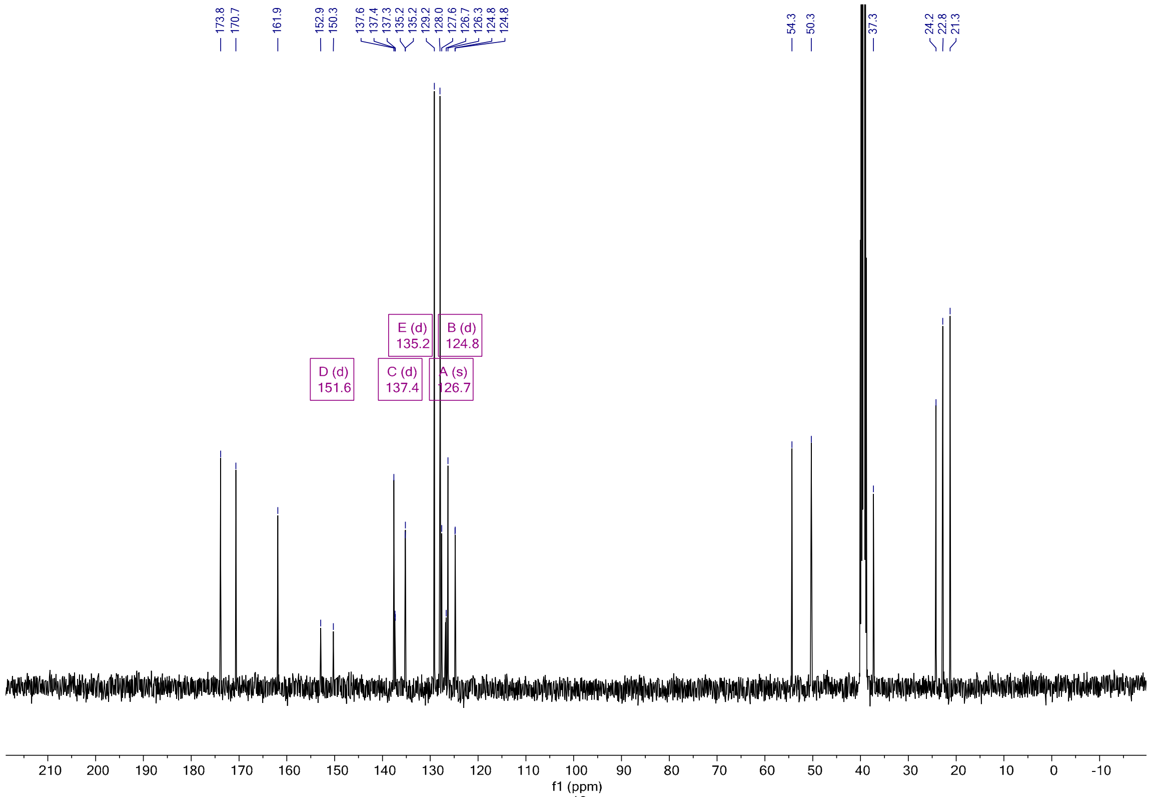

Spectrum 39: ^{13}C-NMR of **10**

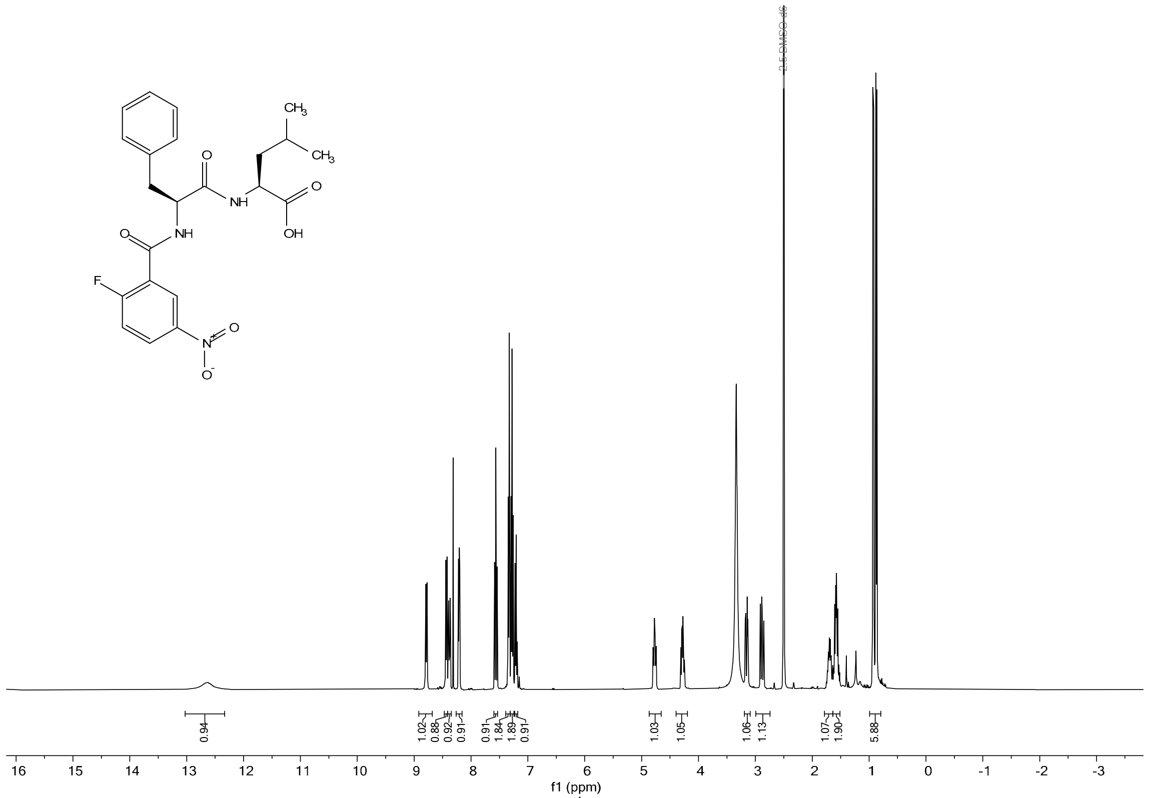

Spectrum 40: ^{1}H-NMR of **11**

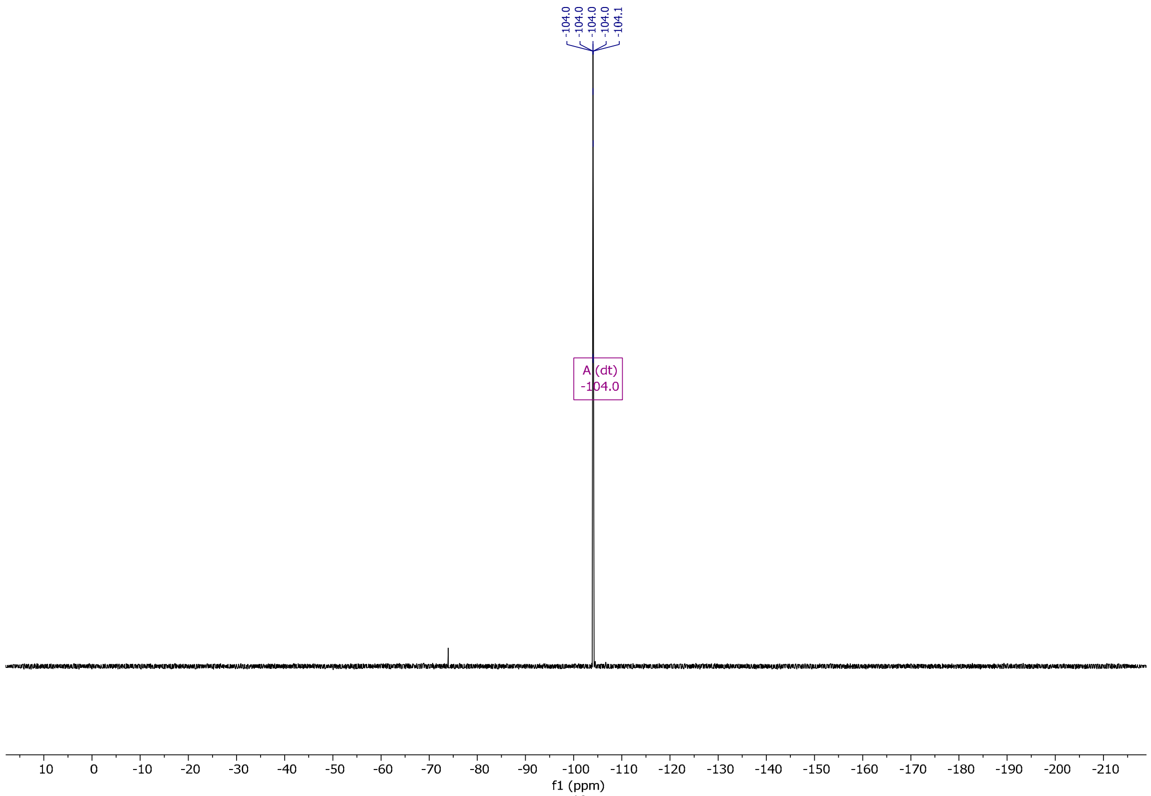

Spectrum 41: ^{19}F-NMR of **11**

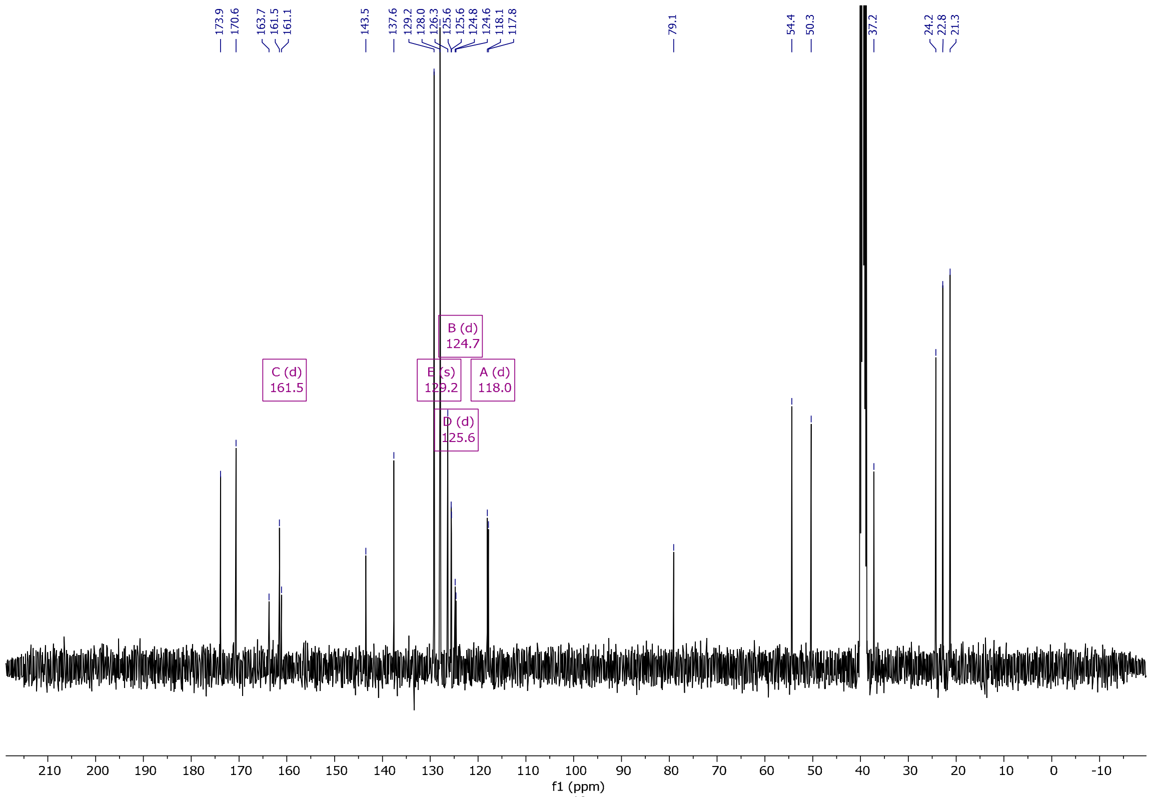

Spectrum 42: ^{13}C-NMR of **11**

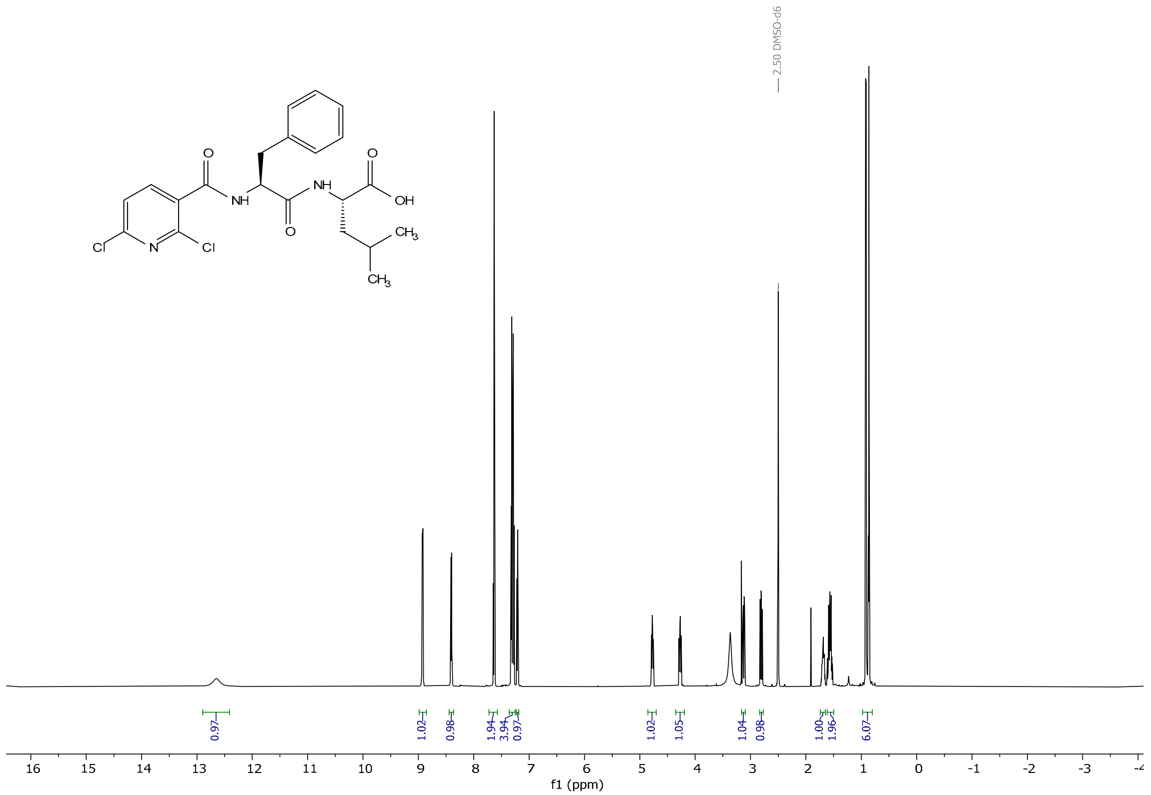

Spectrum 43: ^{1}H-NMR of **8**

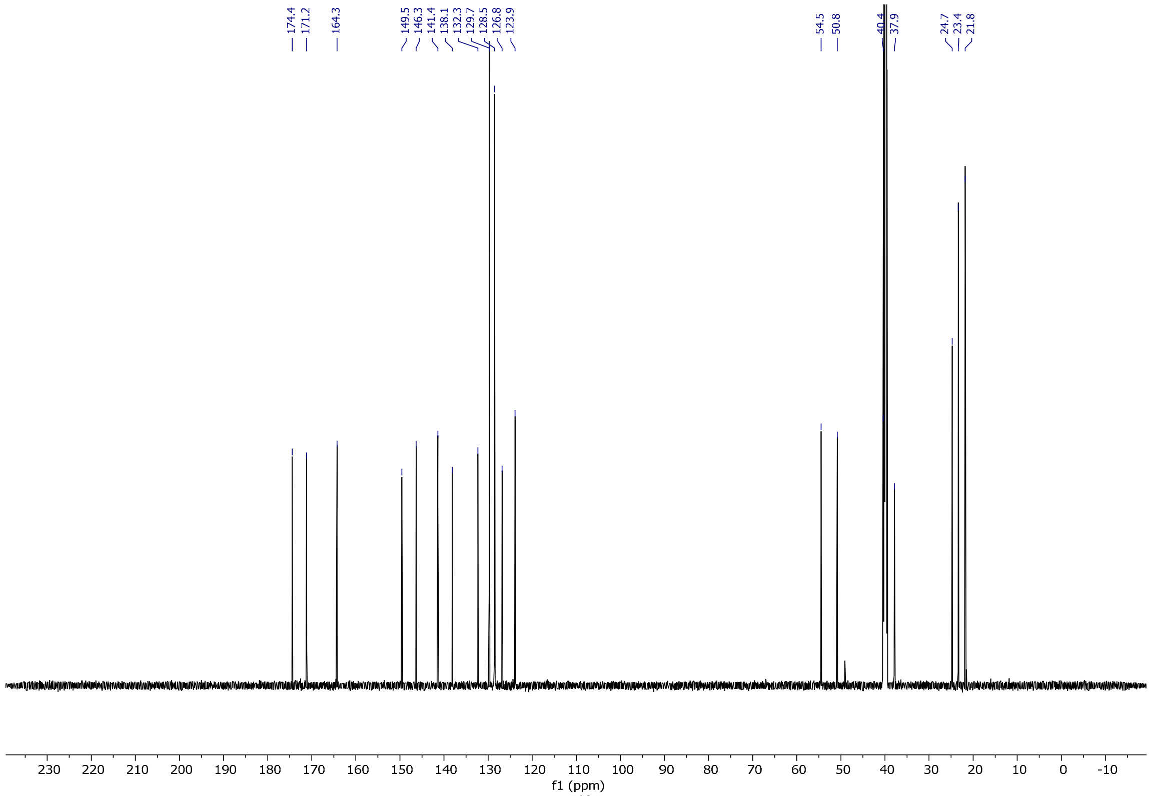

Spectrum 44: ^{13}C-NMR of **8**

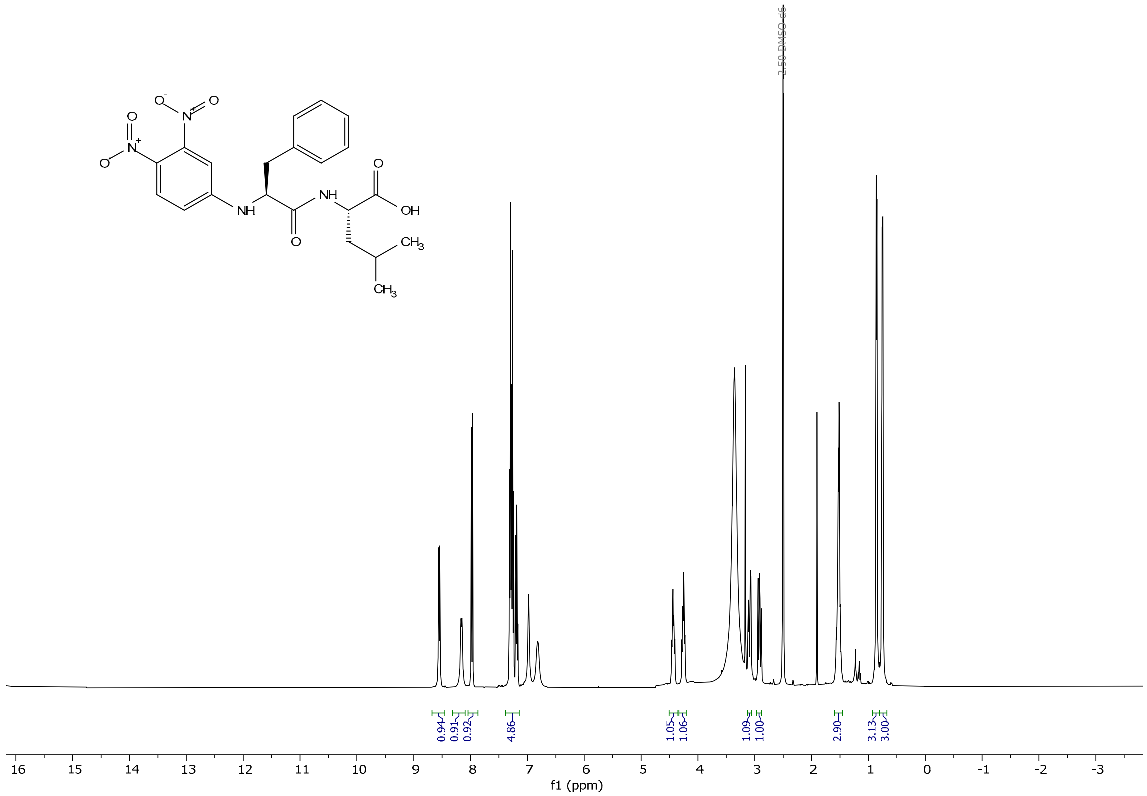

Spectrum 45: ^{1}H-NMR of **2**

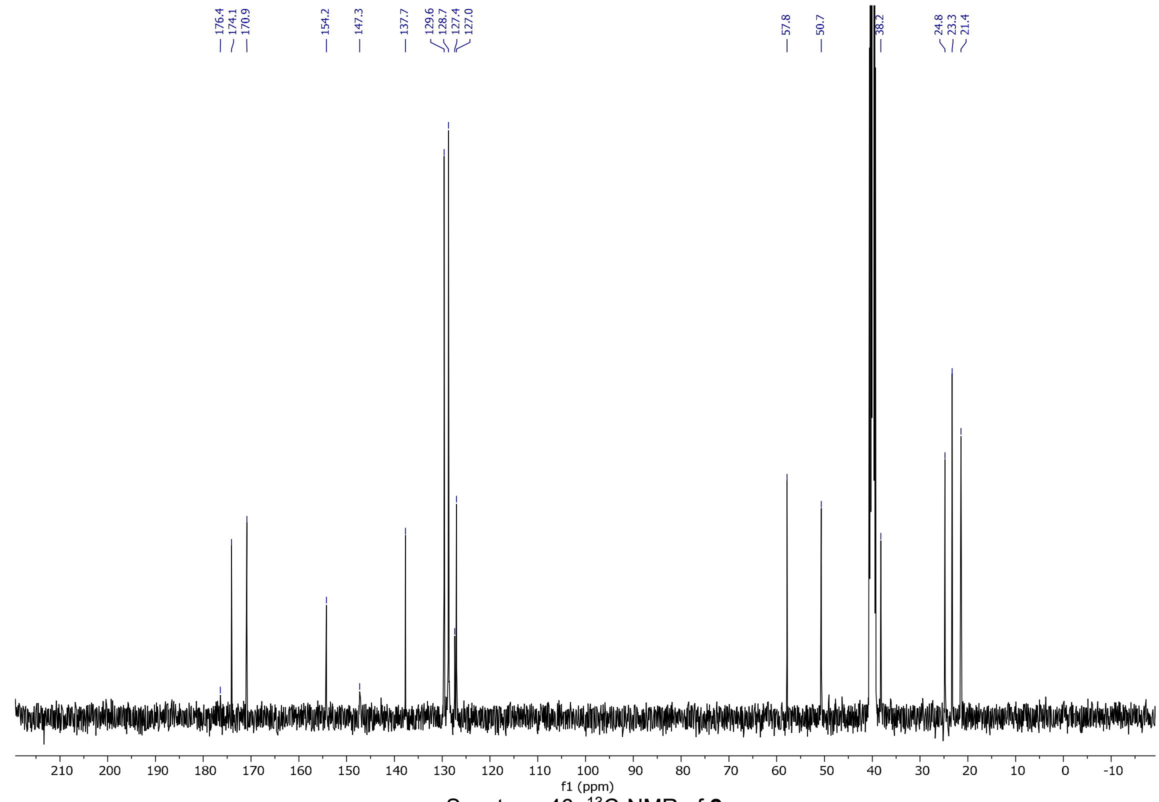

Spectrum 46: ^{13}C-NMR of **2**

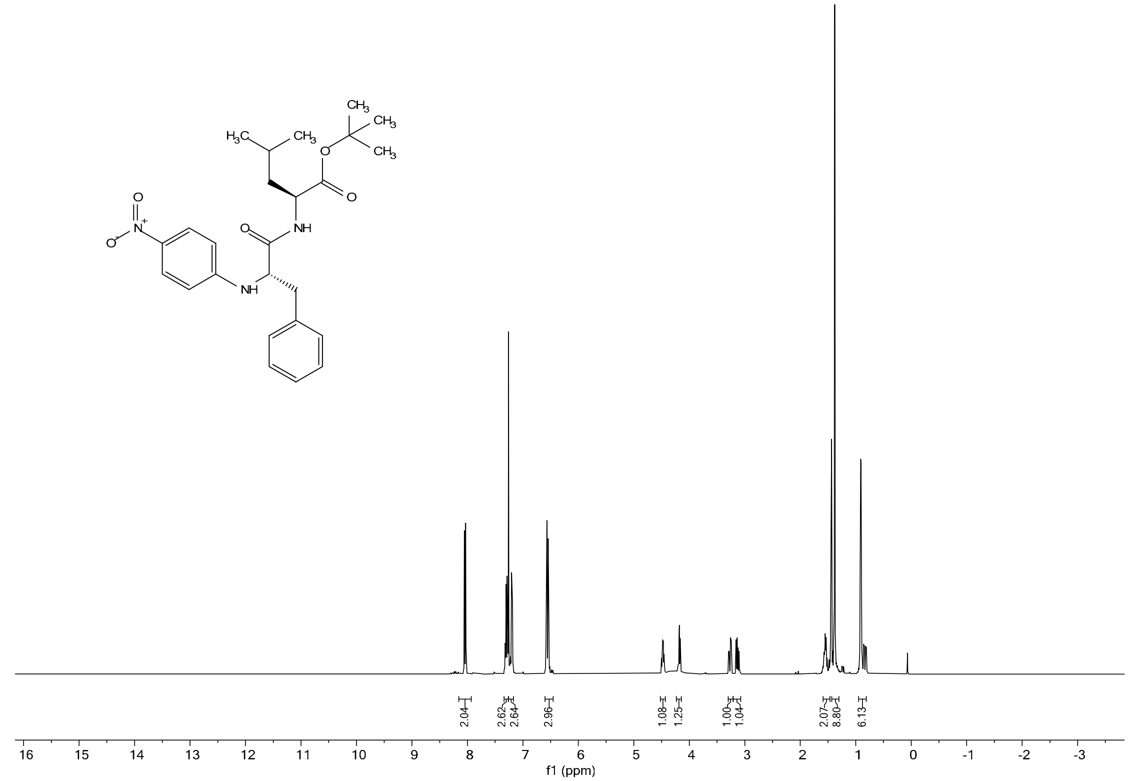

Spectrum 47: ^{1}H-NMR of **22**

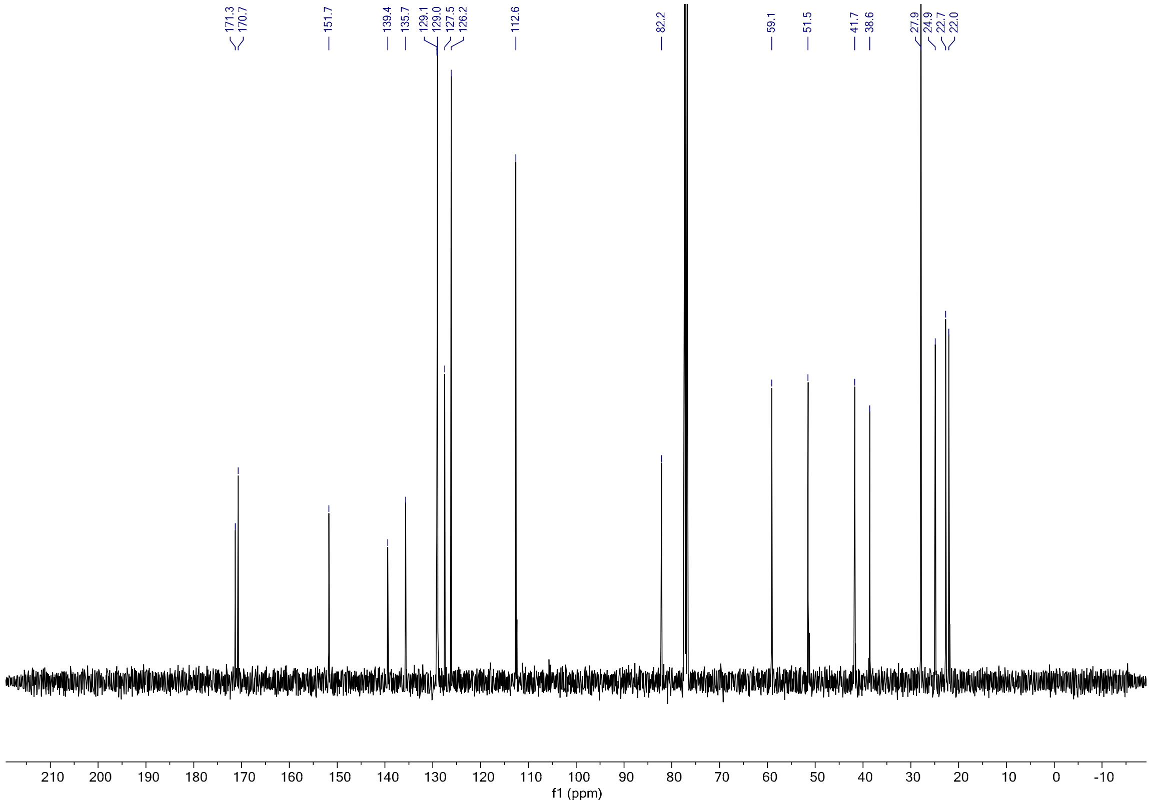

Spectrum 48: ^{13}C-NMR of **22**

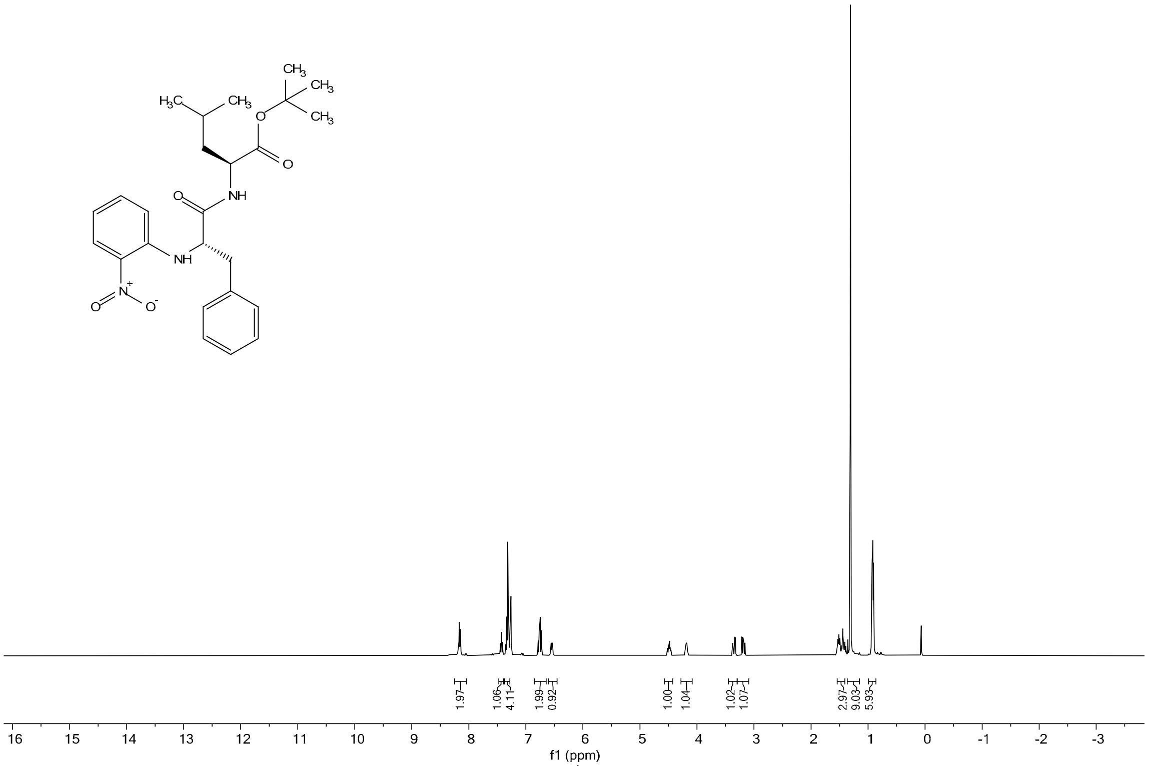

Spectrum 49: ^{1}H-NMR of **19**

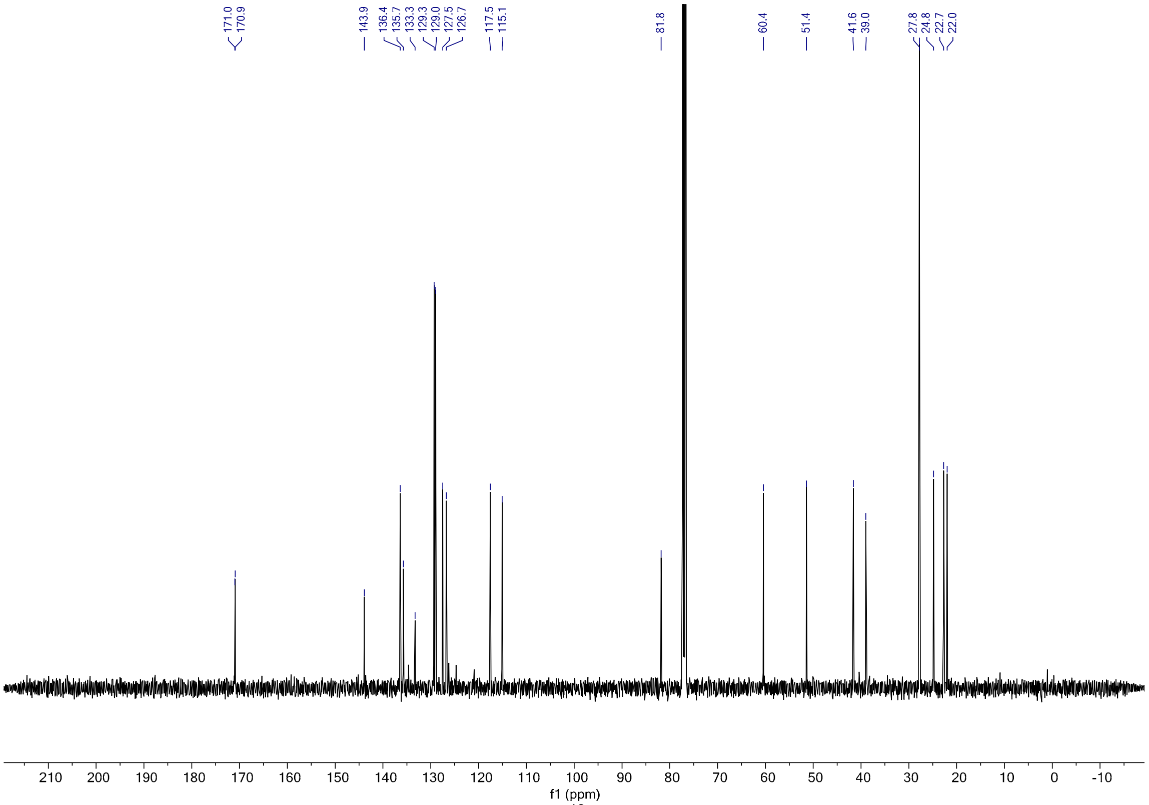

Spectrum 50: ^{13}C-NMR of **19**

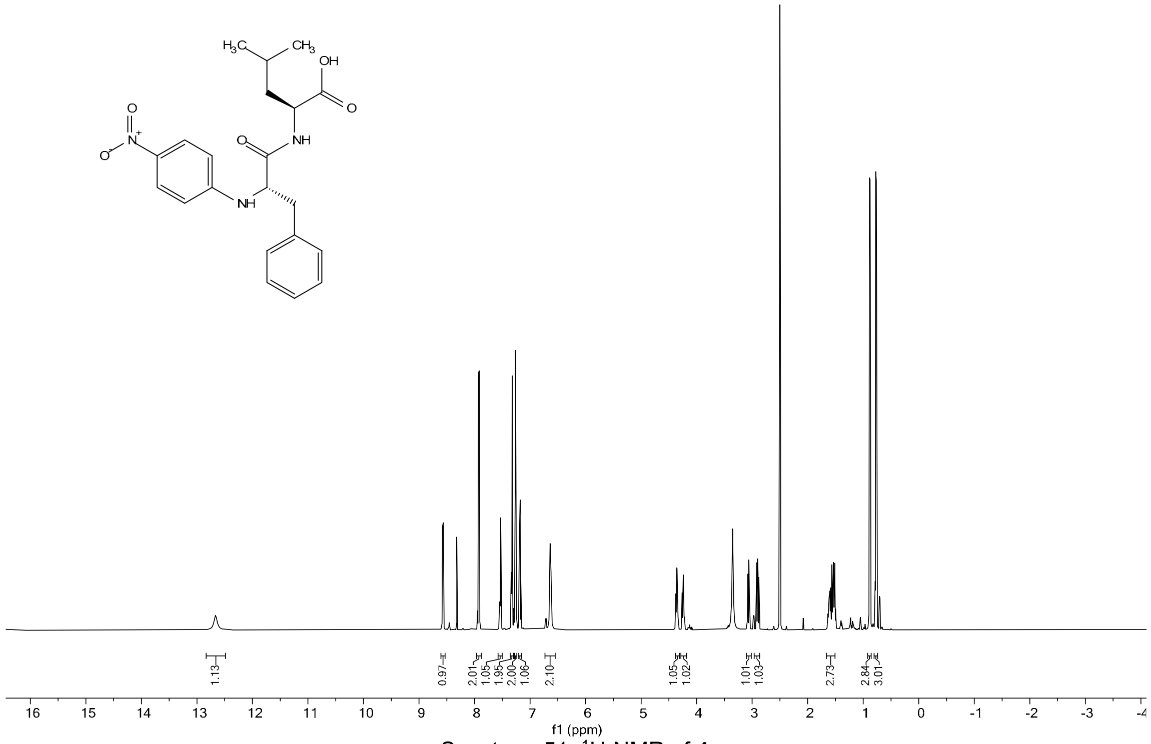

Spectrum 51: ^{1}H-NMR of **4**

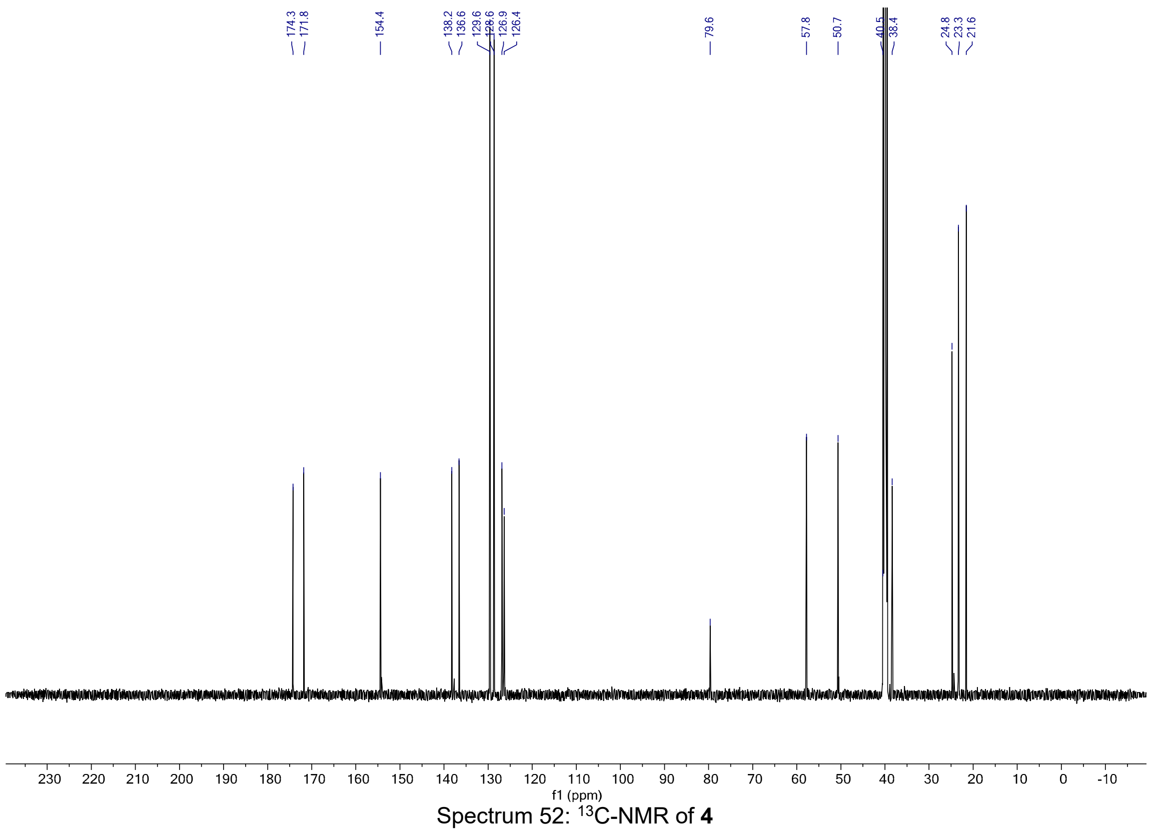

Spectrum 52: ^{13}C-NMR of **4**

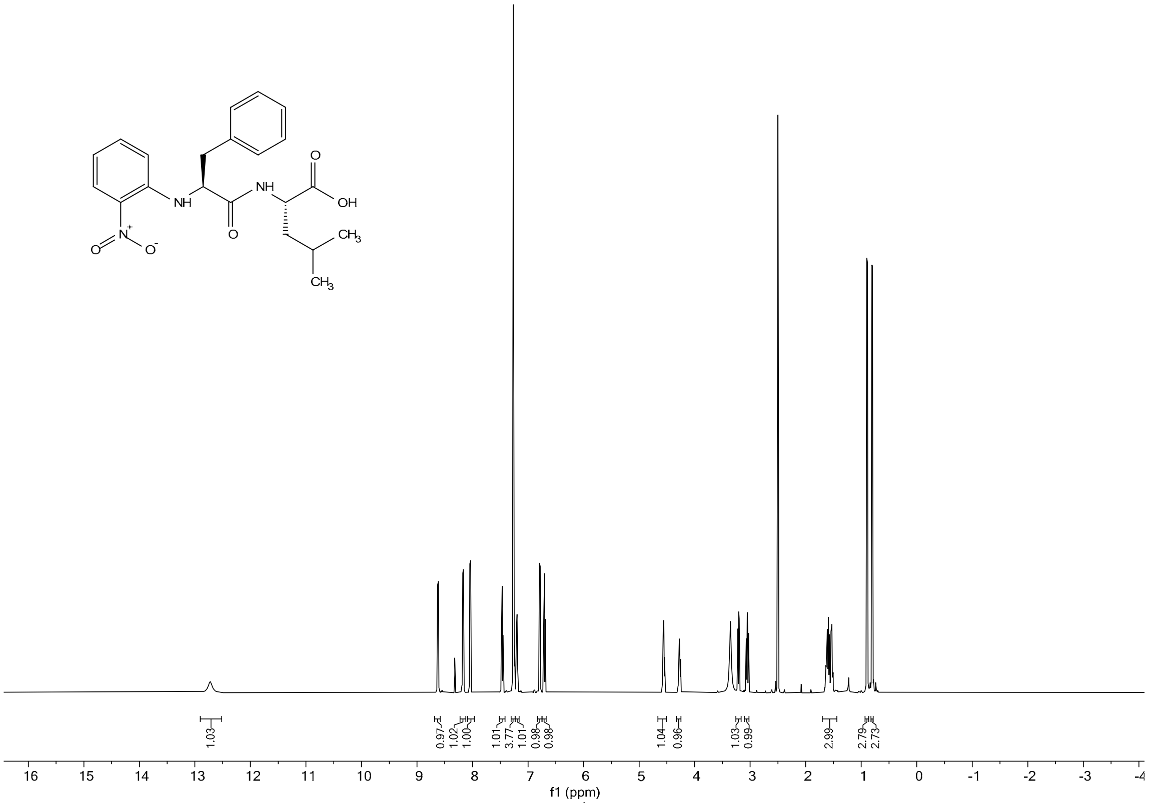

Spectrum 53: ^{1}H-NMR of **1**

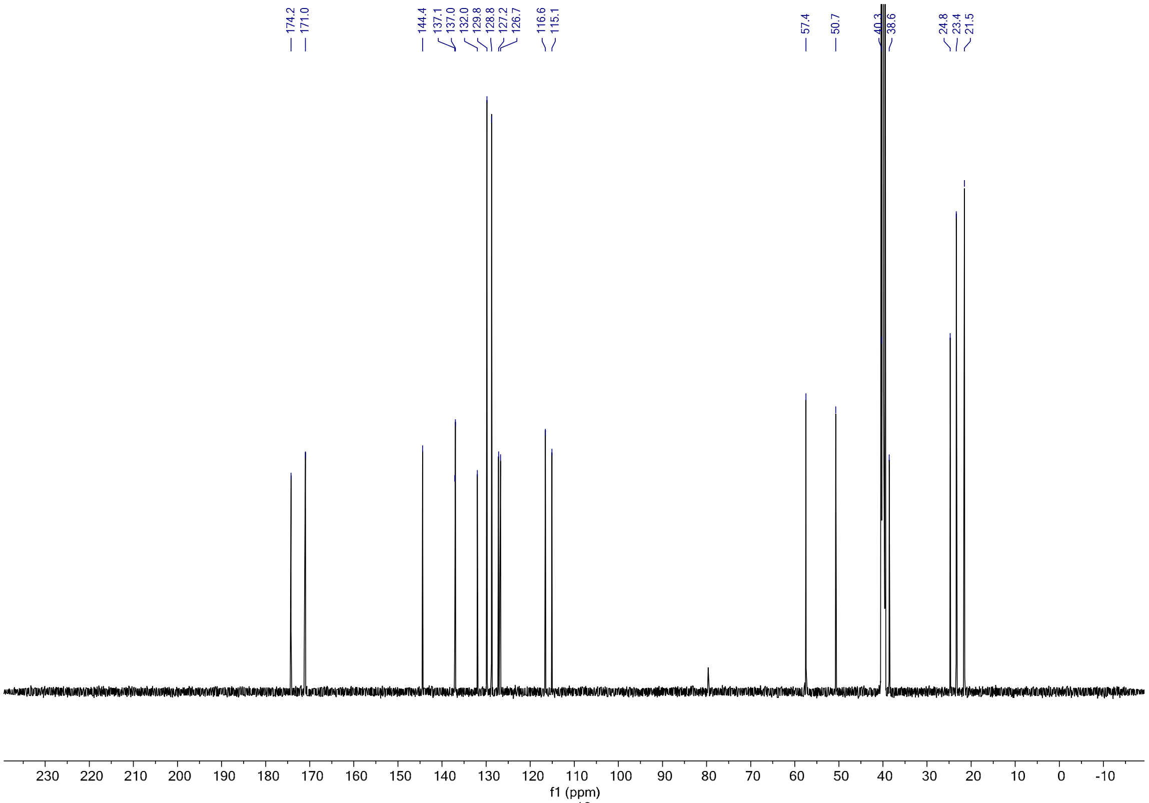

Spectrum 54: ^{13}C-NMR of **1**

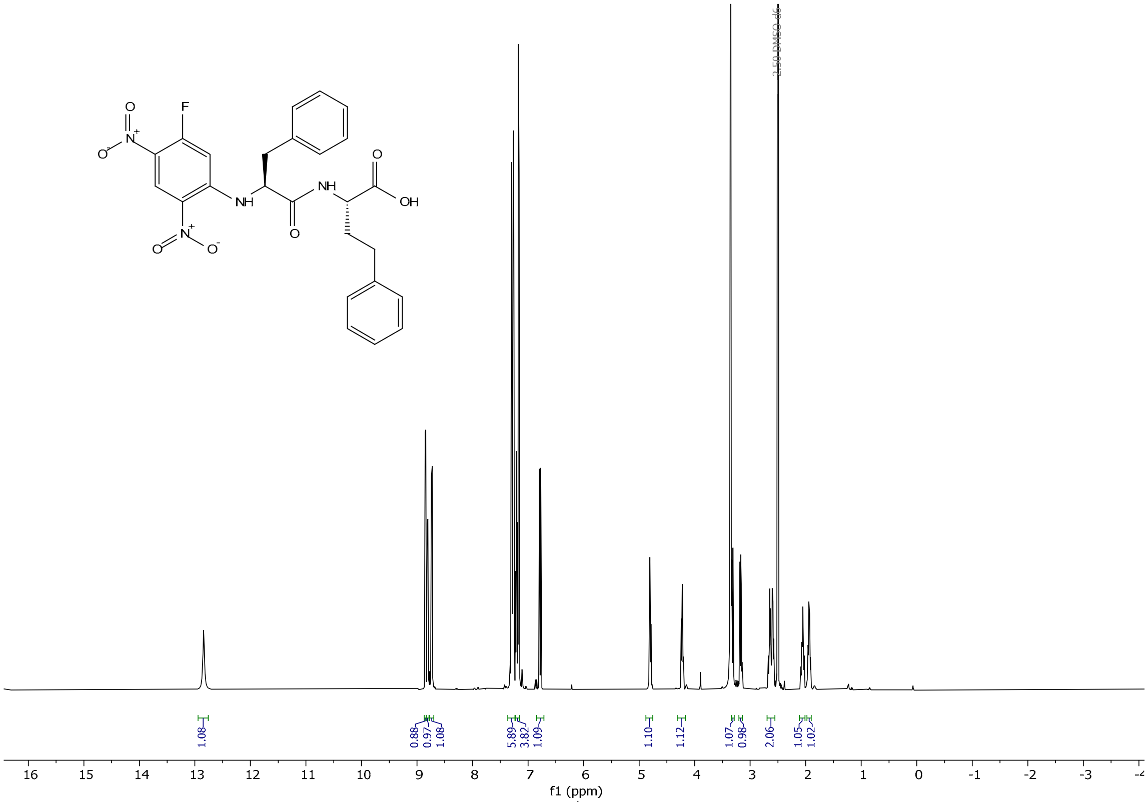

Spectrum 55: ^{1}H-NMR of **17**

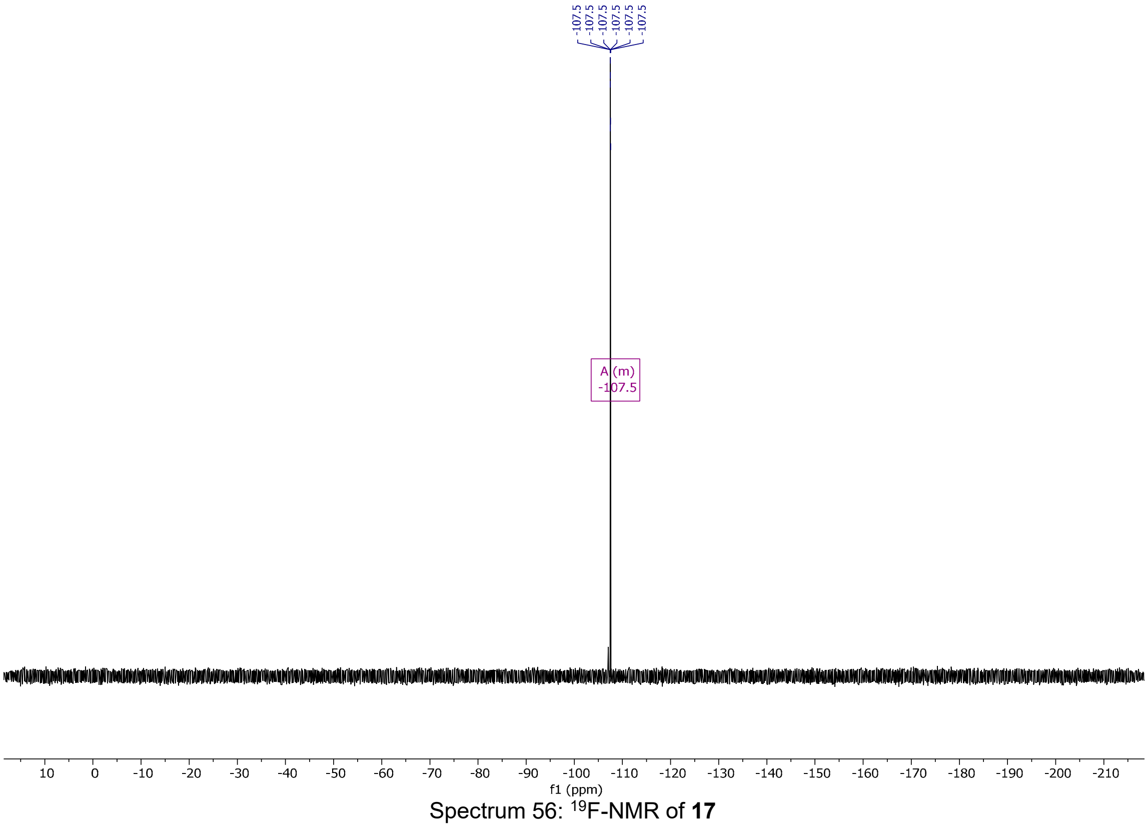

Spectrum 56: ^{19}F-NMR of **17**

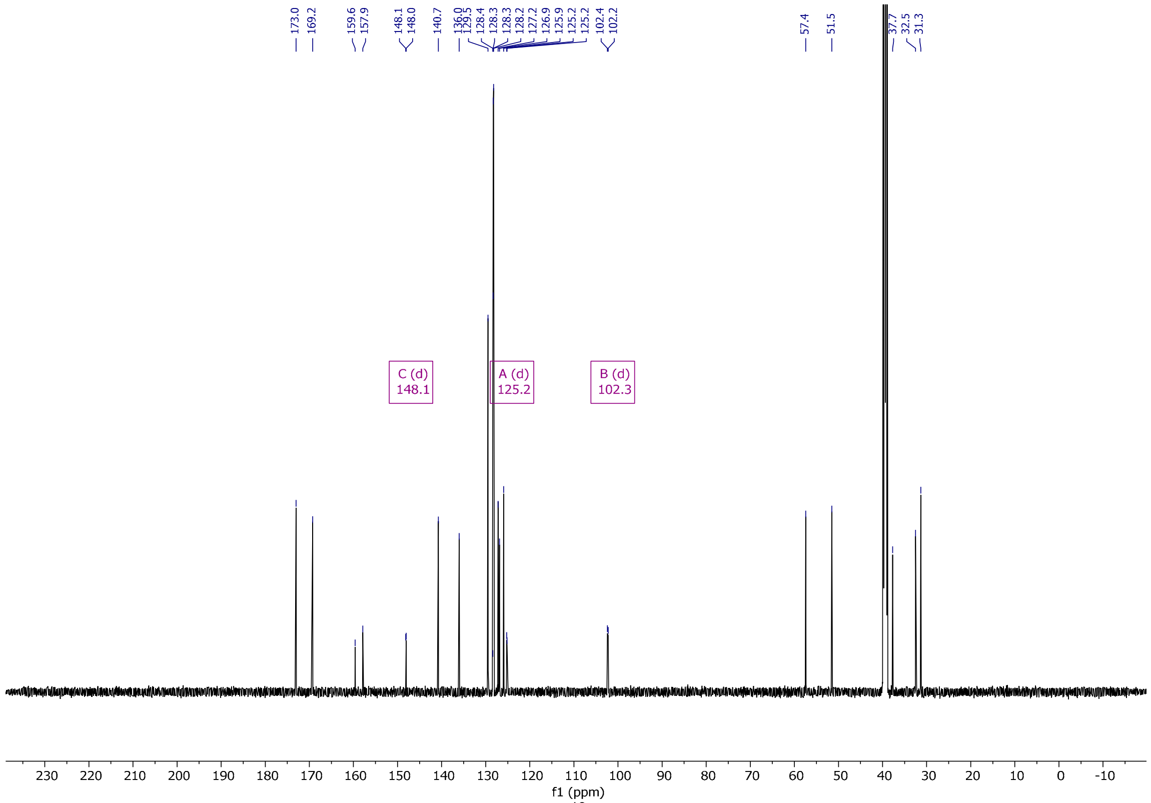

Spectrum 57: ^{13}C-NMR of **17**

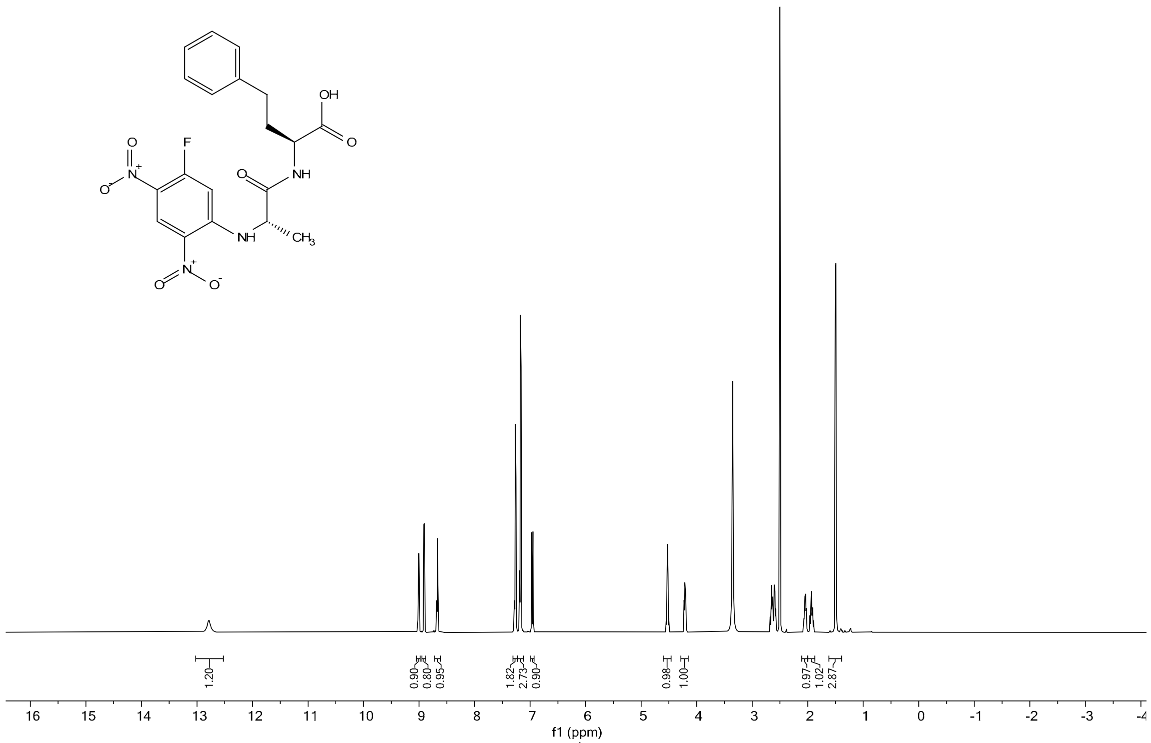

Spectrum 58: ^{1}H-NMR of **13**

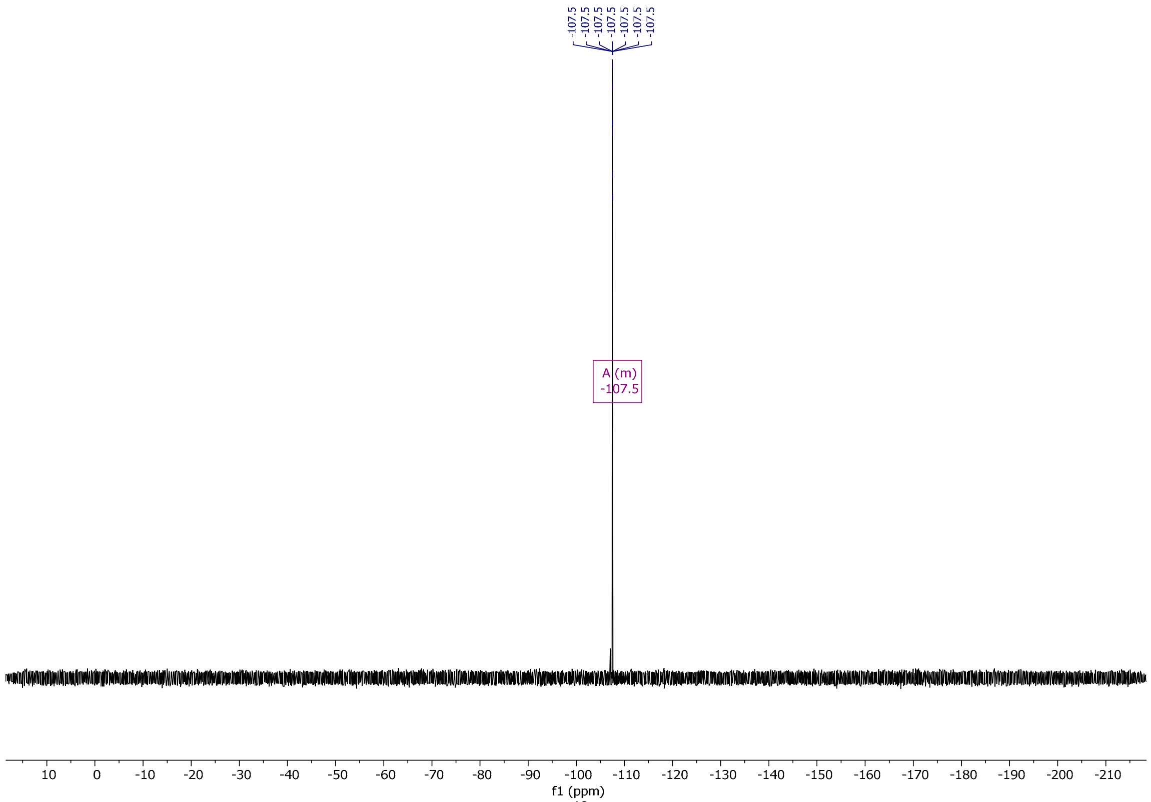

Spectrum 59: ^{19}F-NMR of **13**

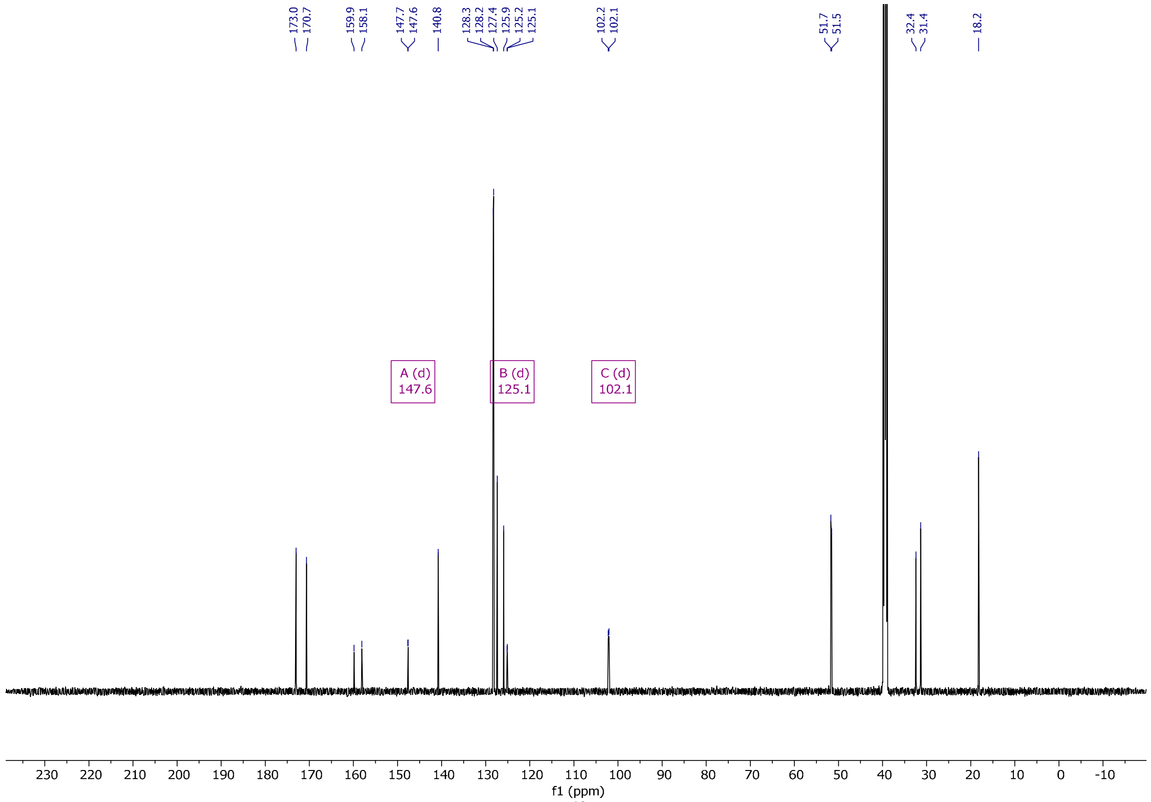

Spectrum 60: ^{13}C-NMR of **13**

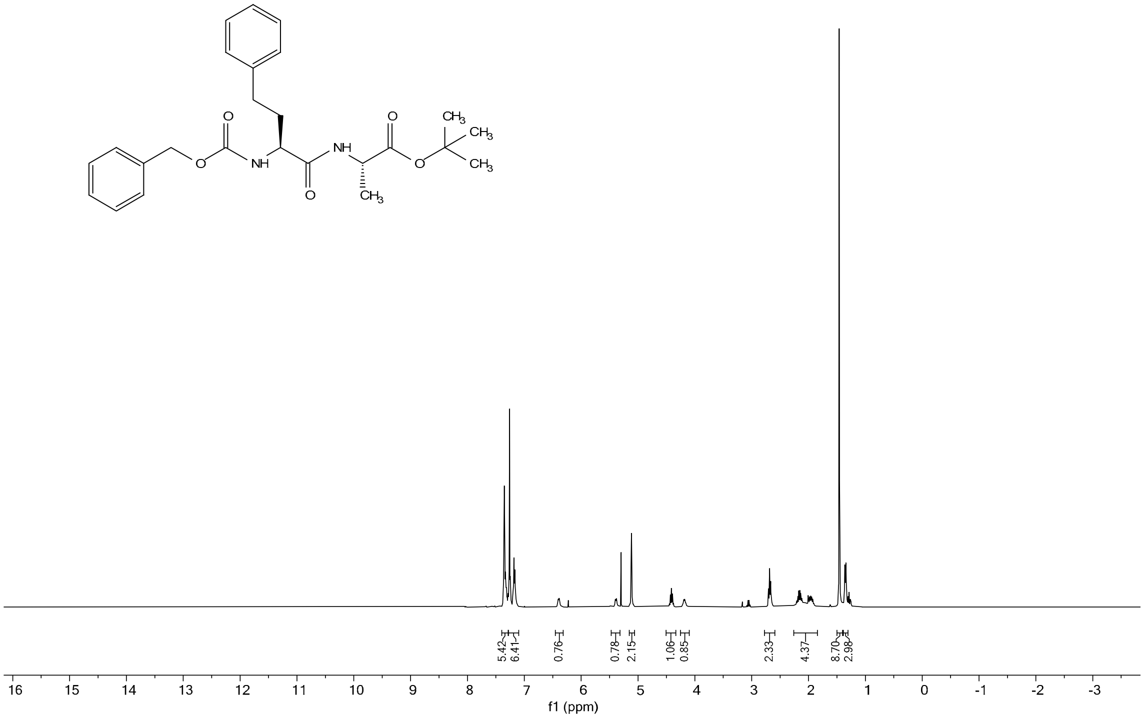

Spectrum 61: ^{1}H-NMR of **42**

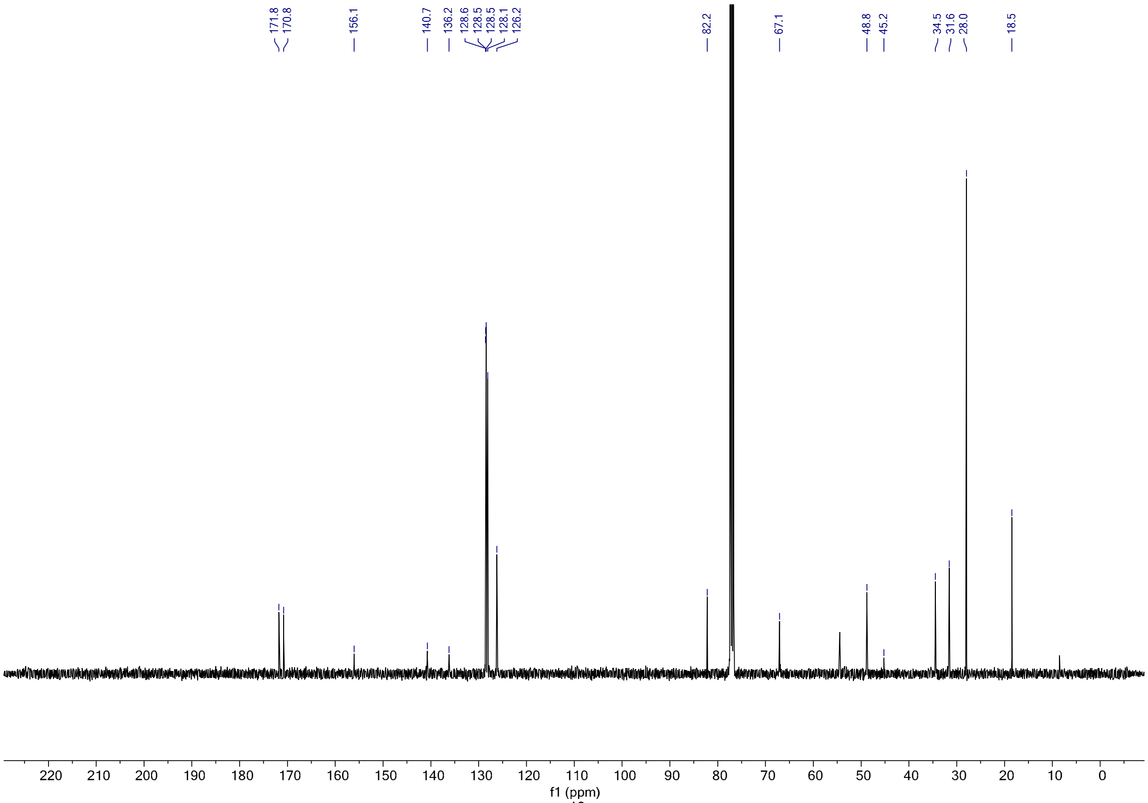

Spectrum 62: ^{13}C-NMR of **42**

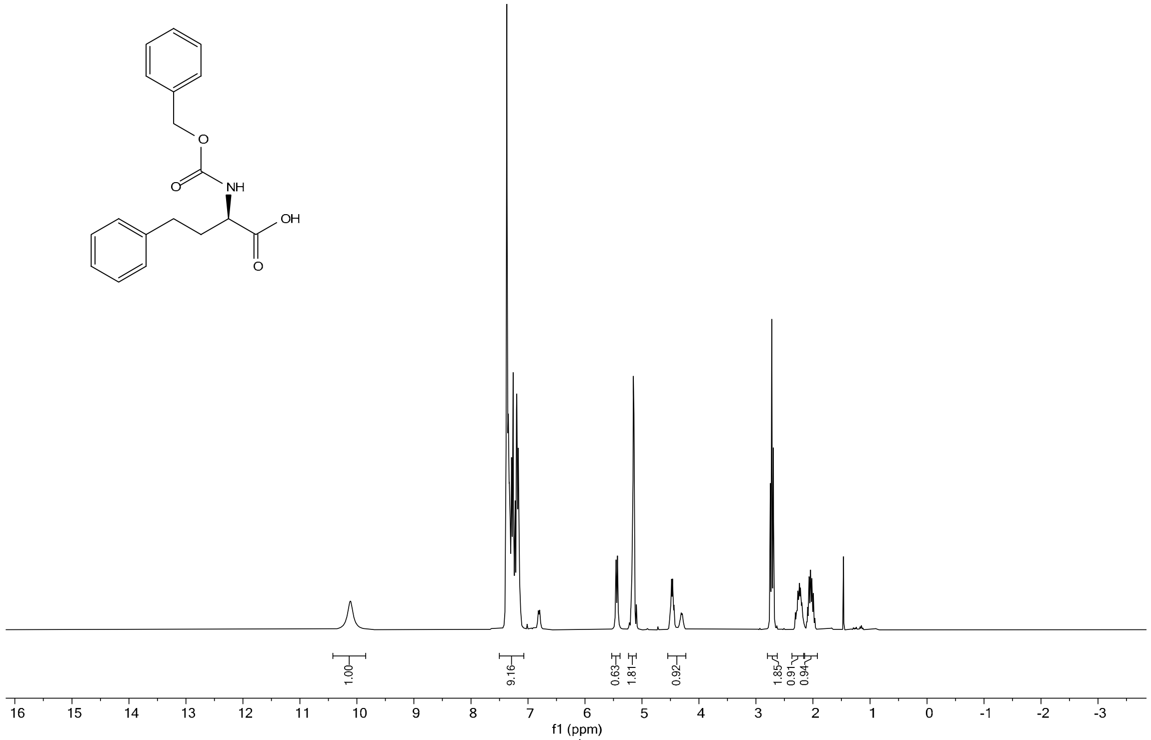

Spectrum 63: ^{1}H-NMR of **45**

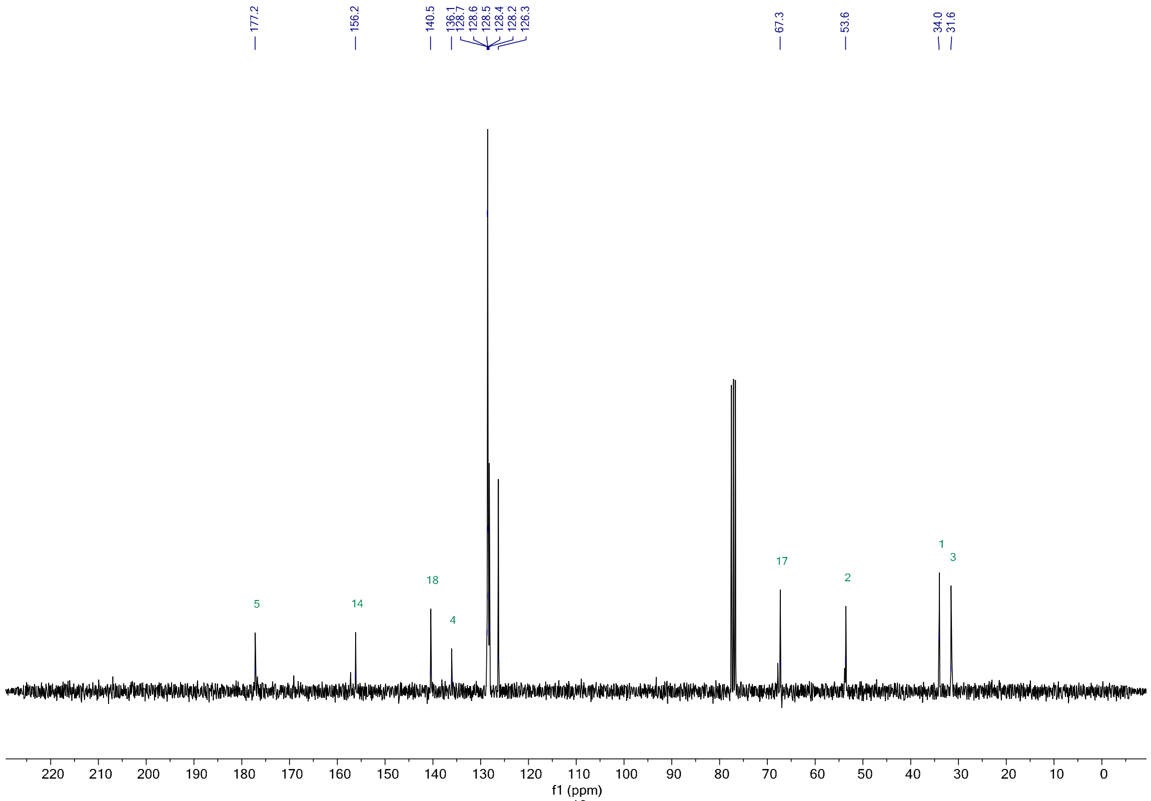

Spectrum 64: ^{13}C-NMR of **45**

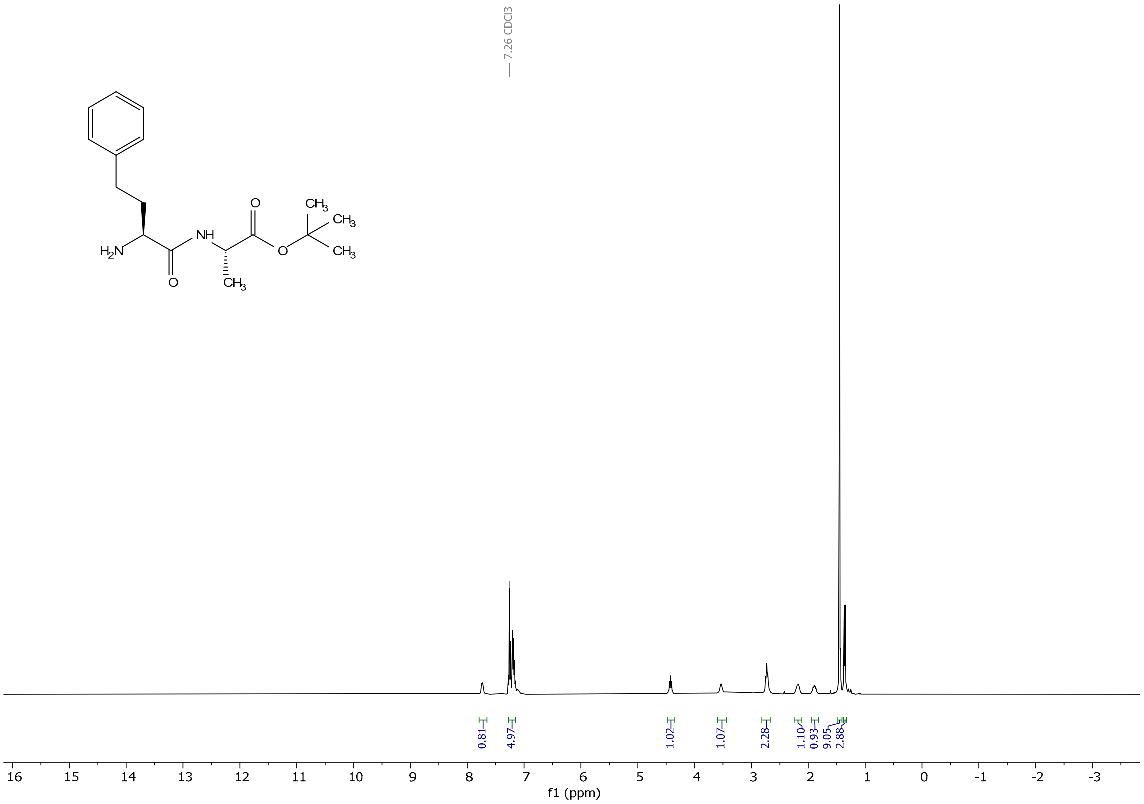

Spectrum 65: ^{1}H-NMR of **39**

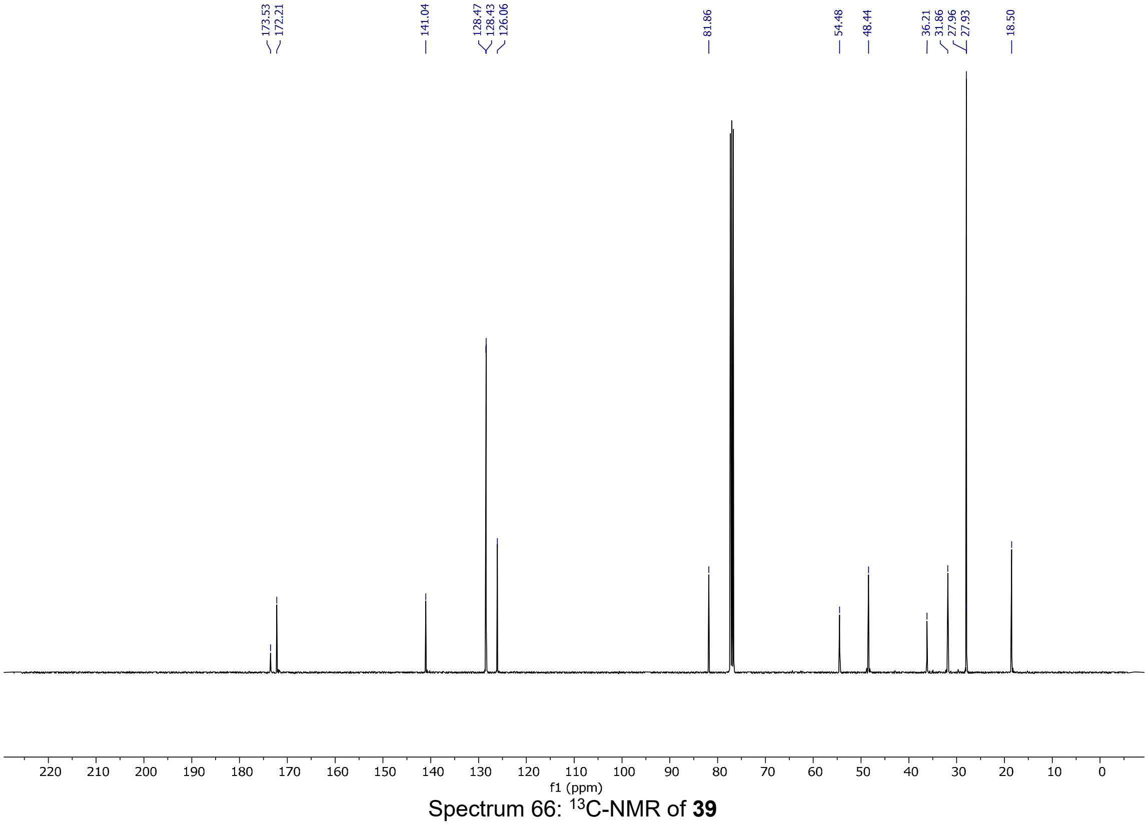

Spectrum 66: ^{13}C-NMR of **39**

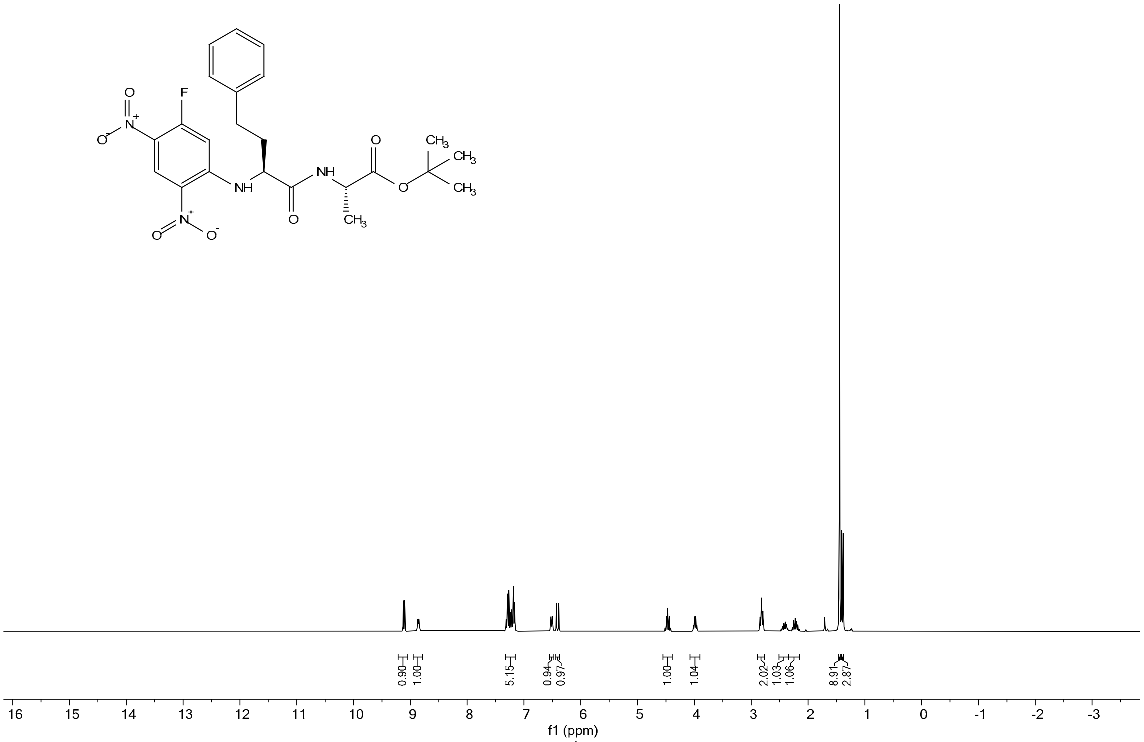

Spectrum 67: ^{1}H-NMR of **37**

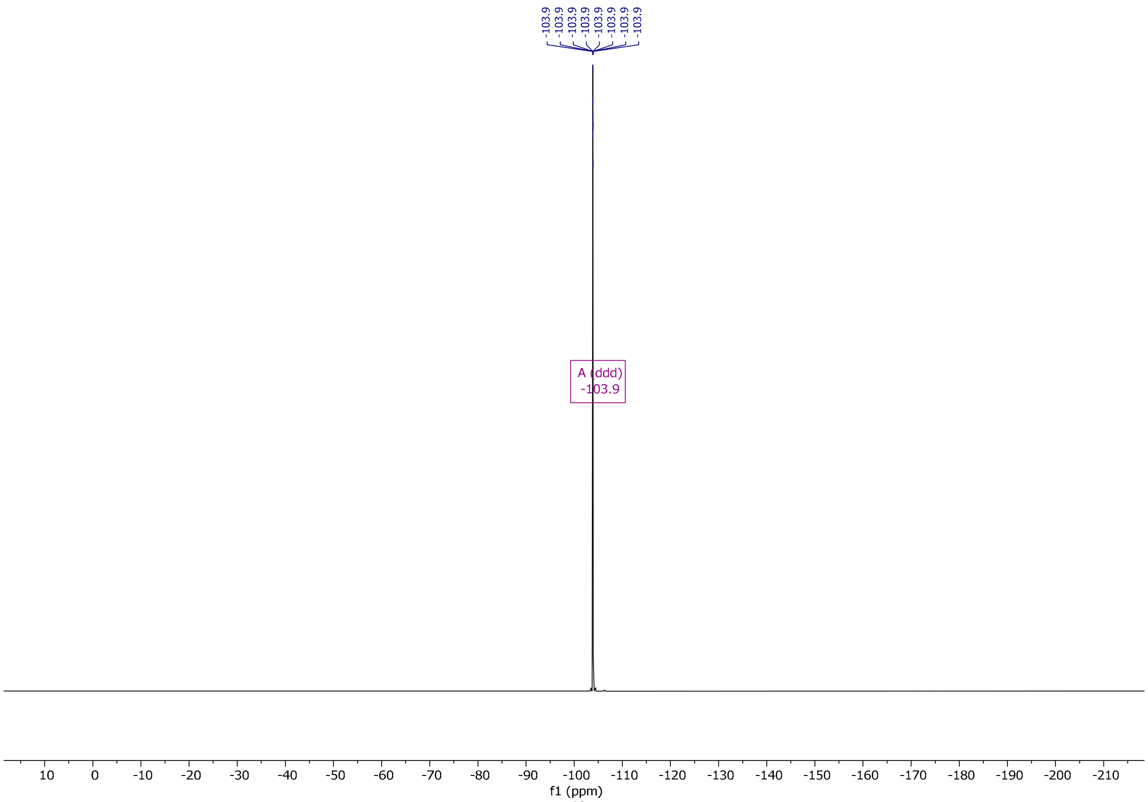

Spectrum 68: ^{19}F-NMR of **37**

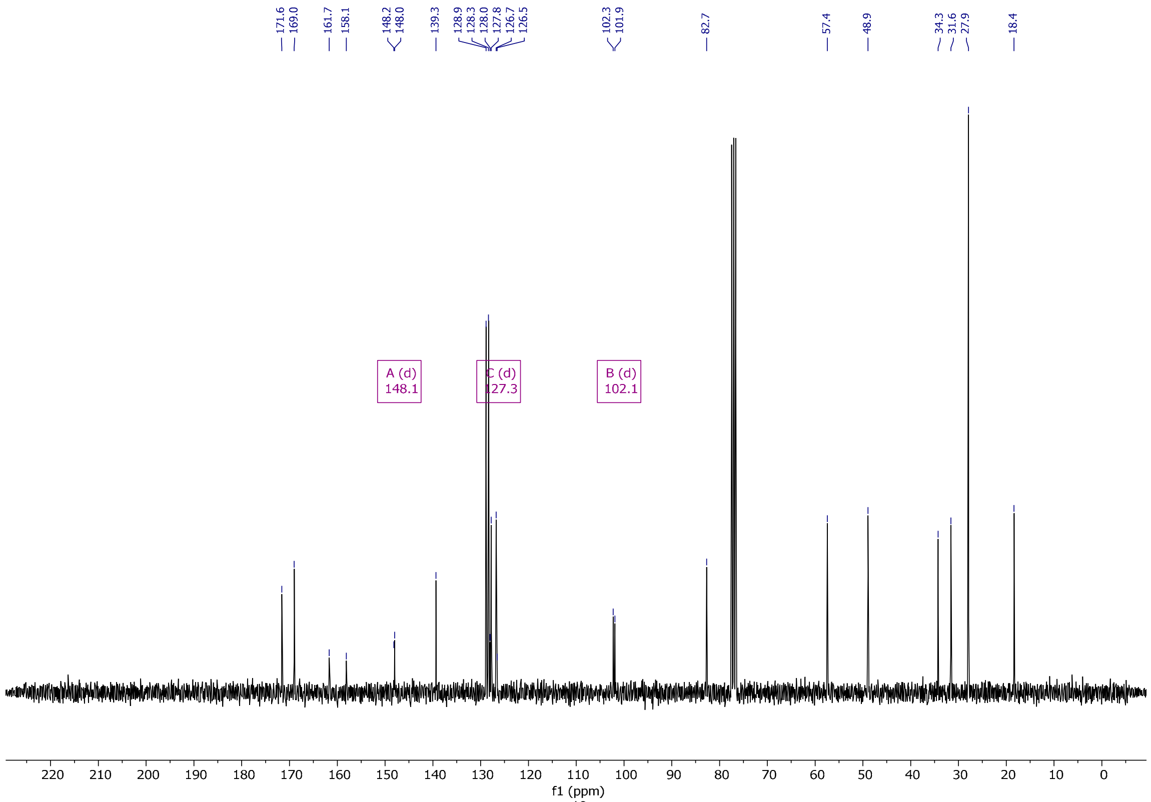

Spectrum 69: ^{13}C-NMR of **37**

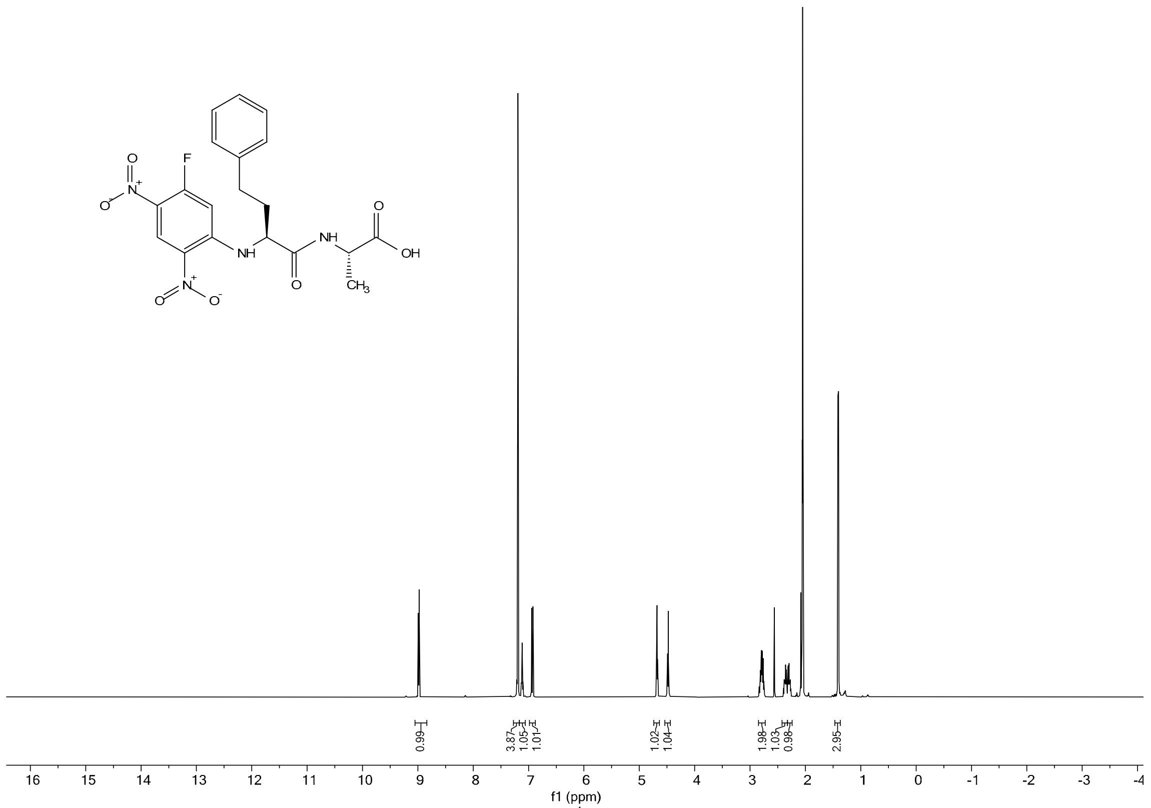

Spectrum 70: ^{1}H-NMR of **16**

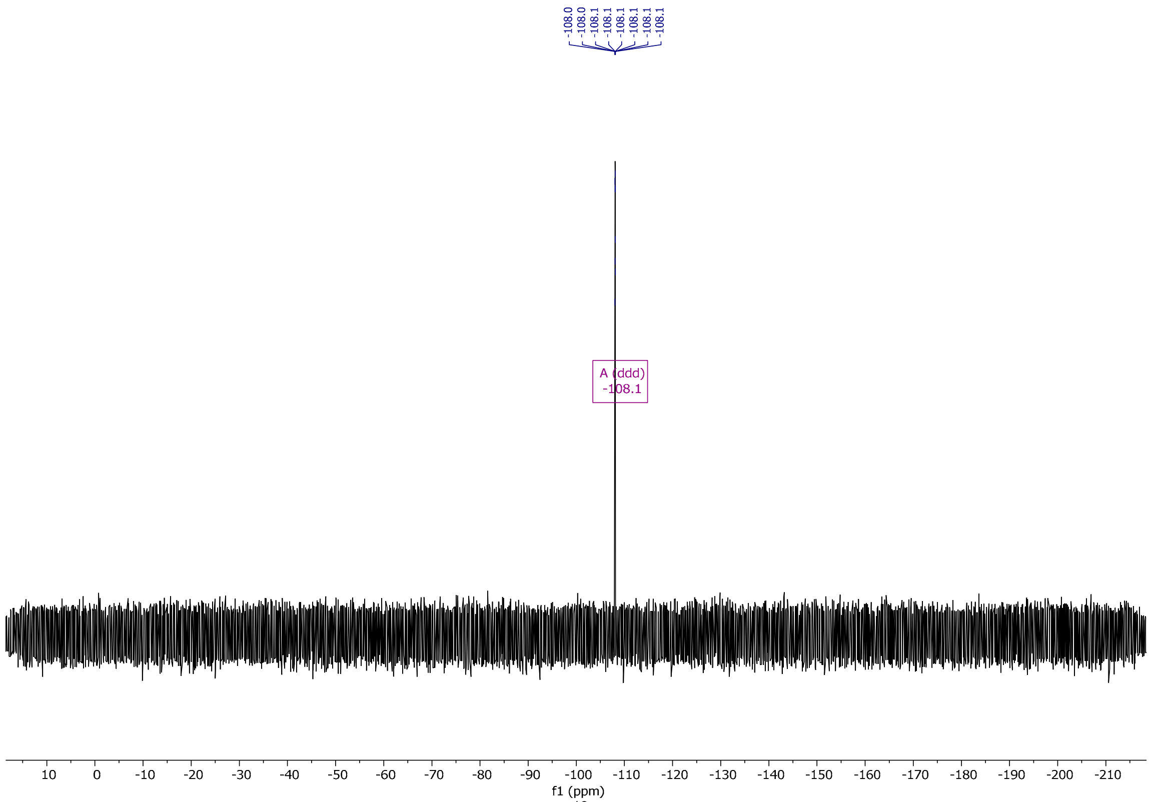

Spectrum 71: ^{19}F-NMR of **16**

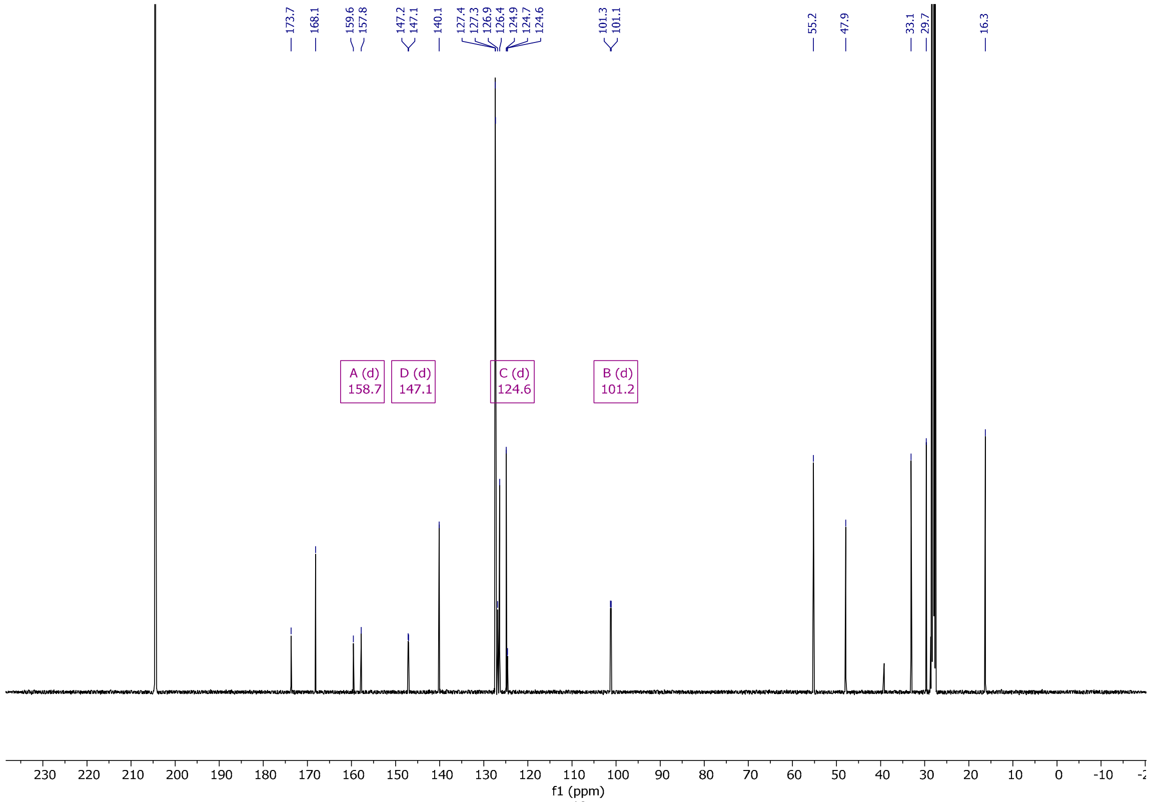

Spectrum 72: ^{13}C-NMR of **16**

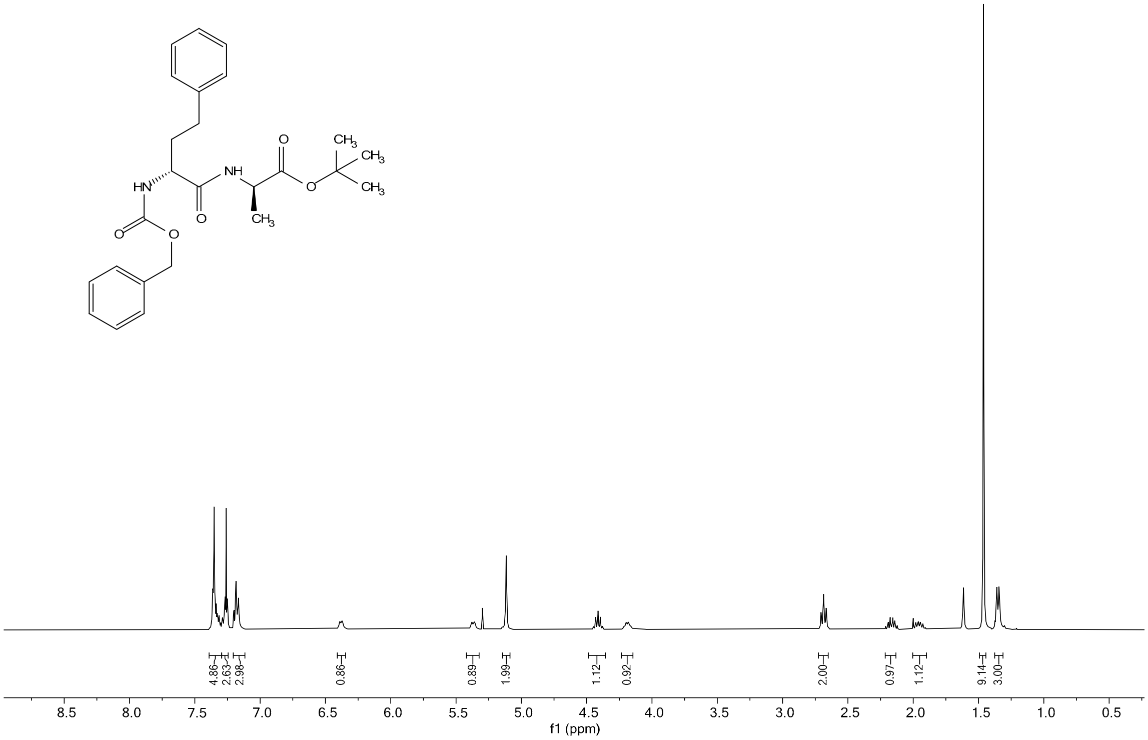

Spectrum 73: ^{1}H-NMR of **44**

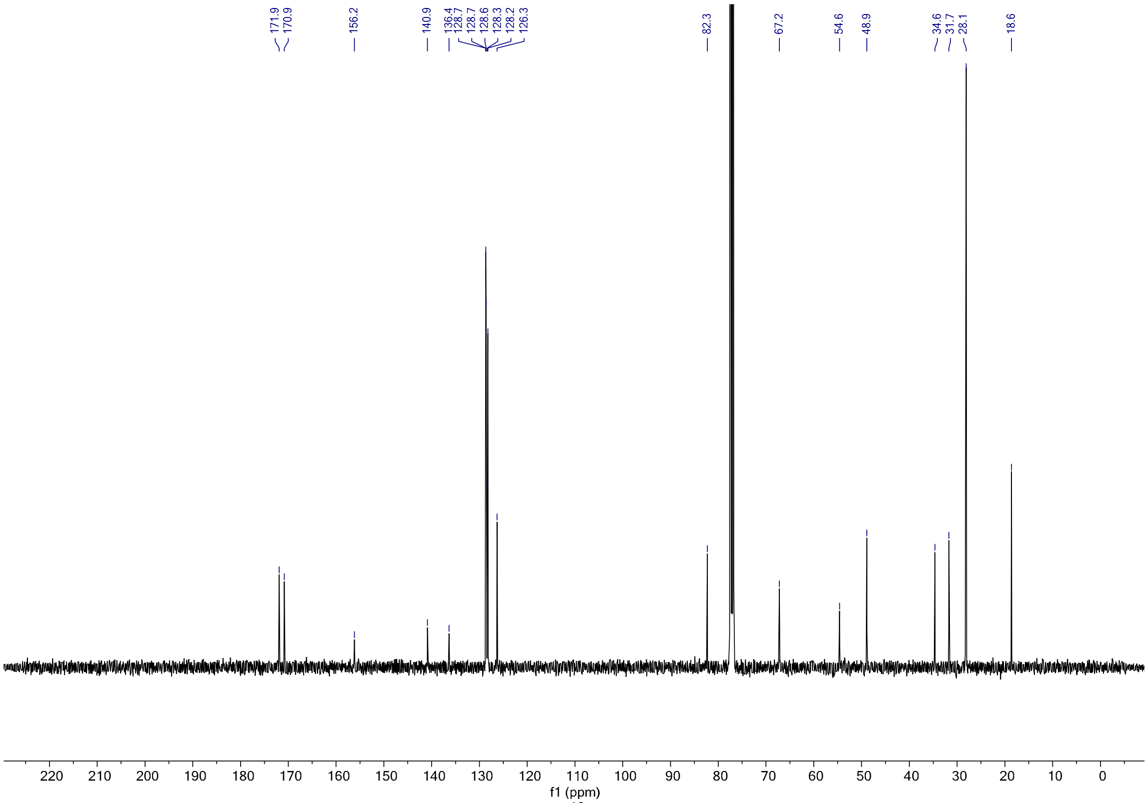

Spectrum 74: ^{13}C-NMR of **44**

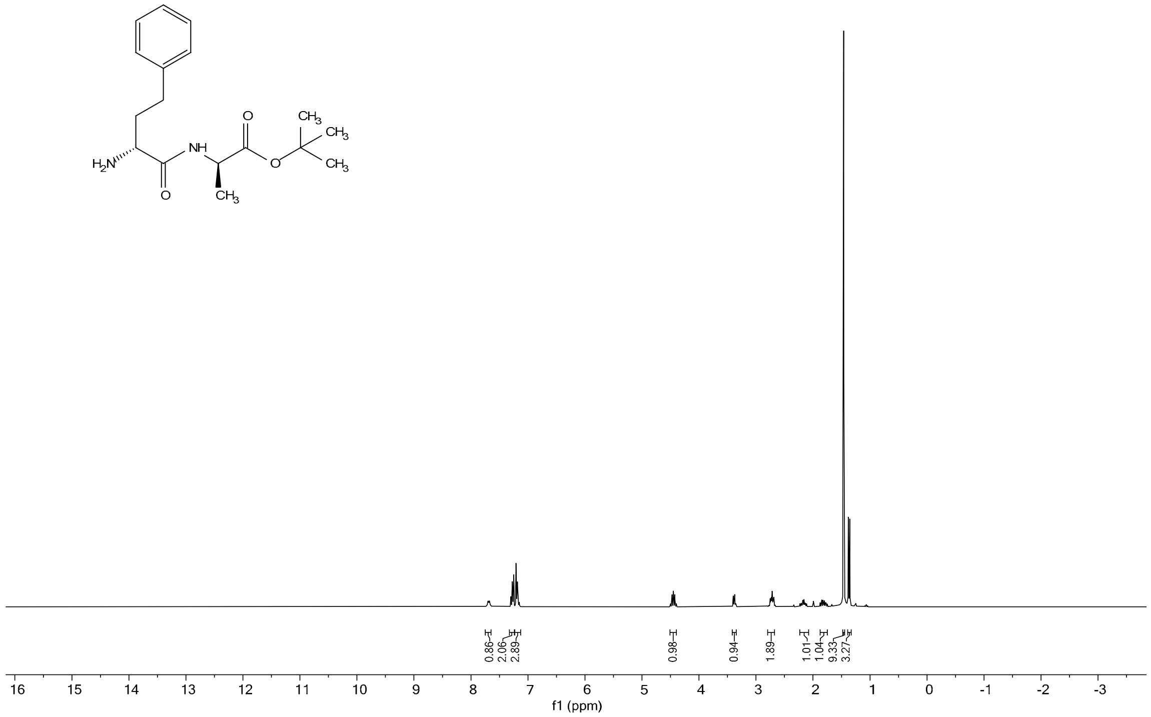

Spectrum 75: ^{1}H-NMR of **41**

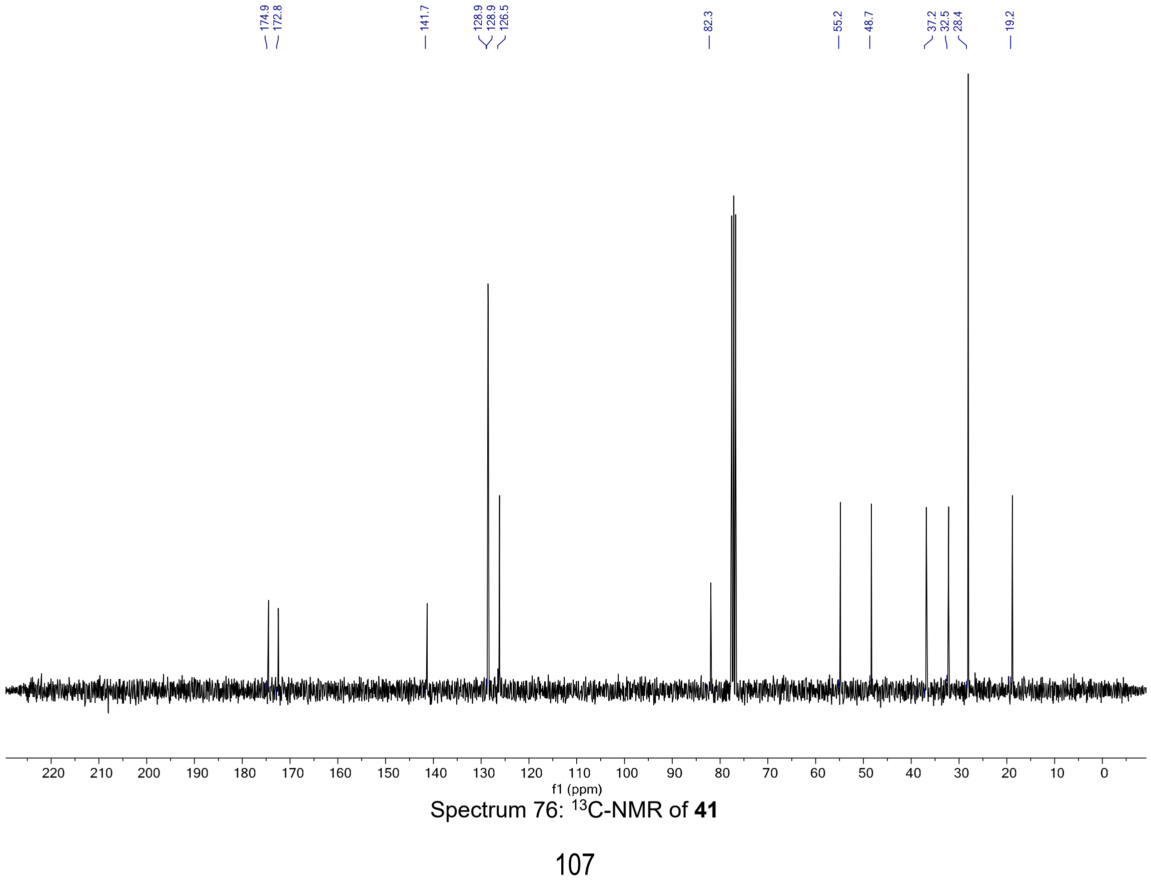

Spectrum 76: ^{13}C-NMR of **41**

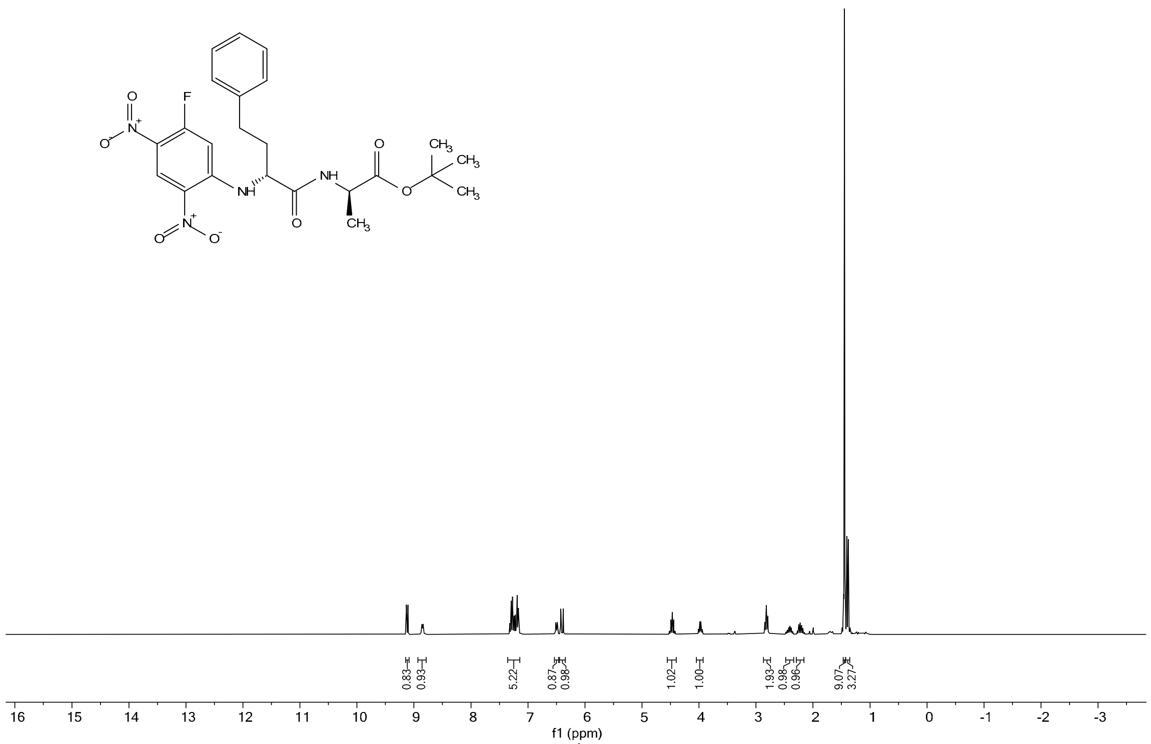

Spectrum 77: ^{1}H-NMR of **35**

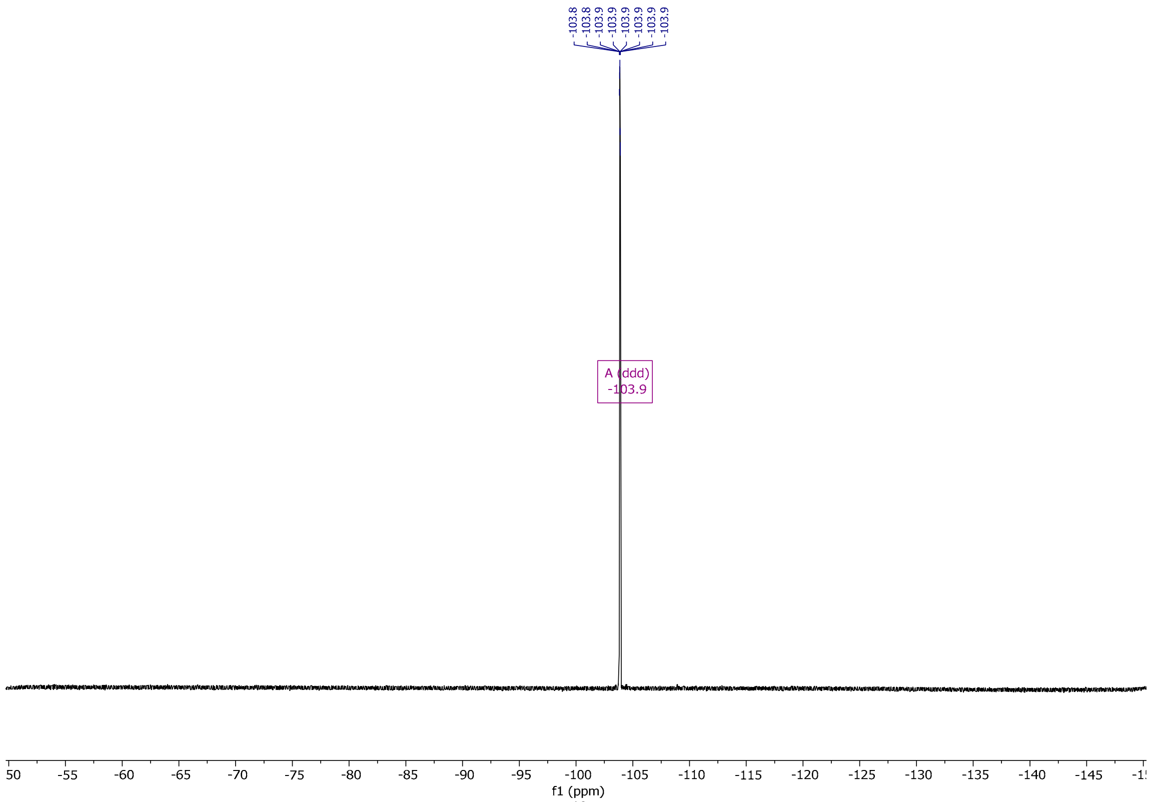

Spectrum 78: ^{19}F-NMR of **35**

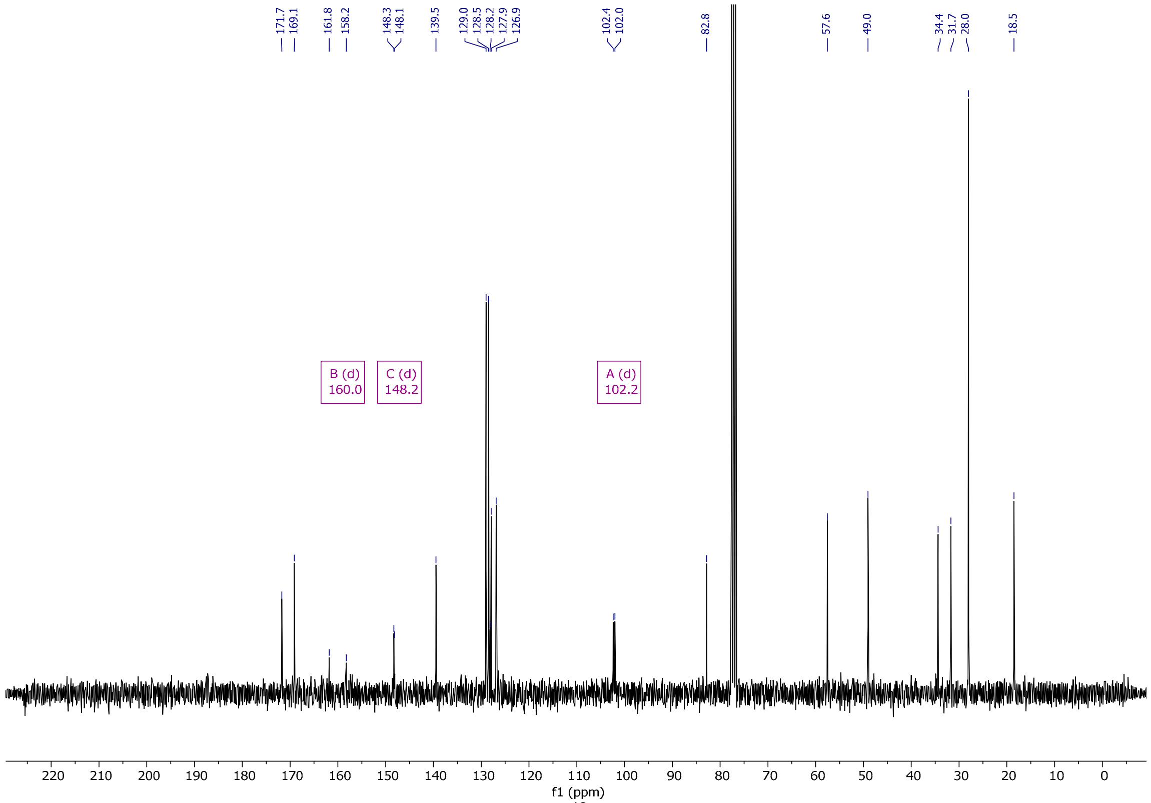

Spectrum 79: ^{13}C-NMR of **35**

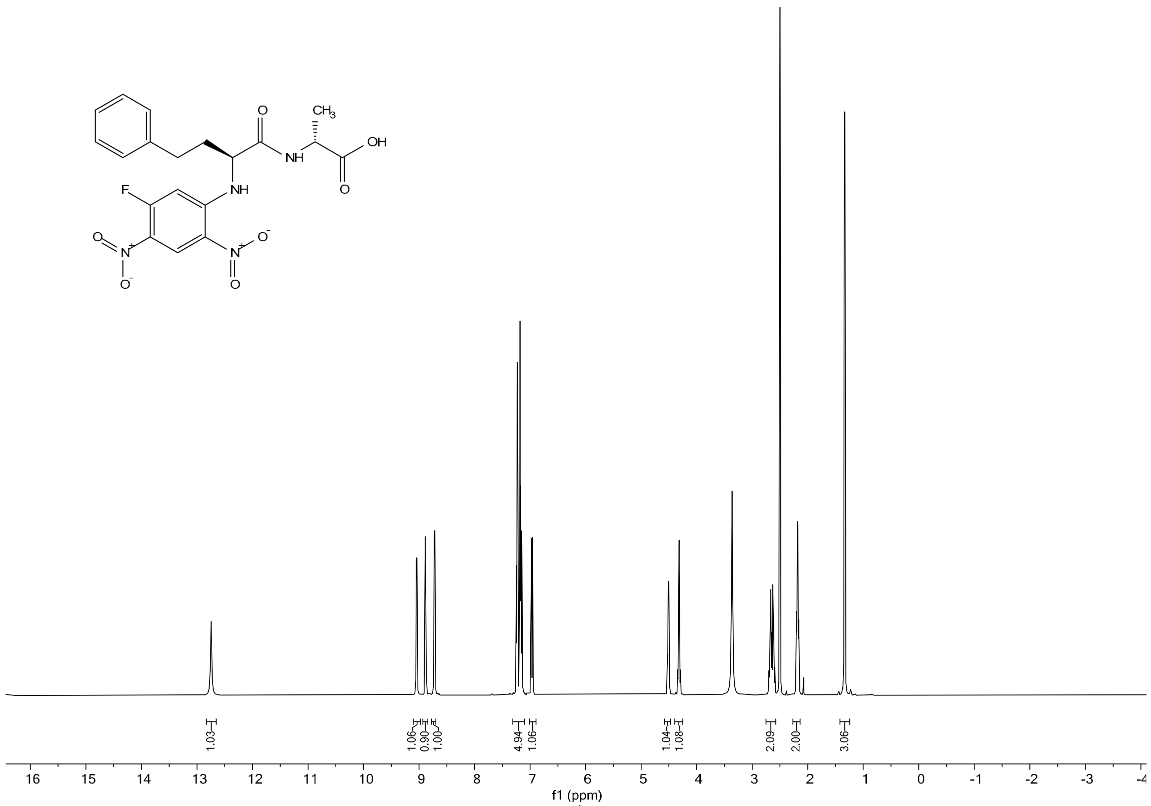

Spectrum 80: ^{1}H-NMR of **14**

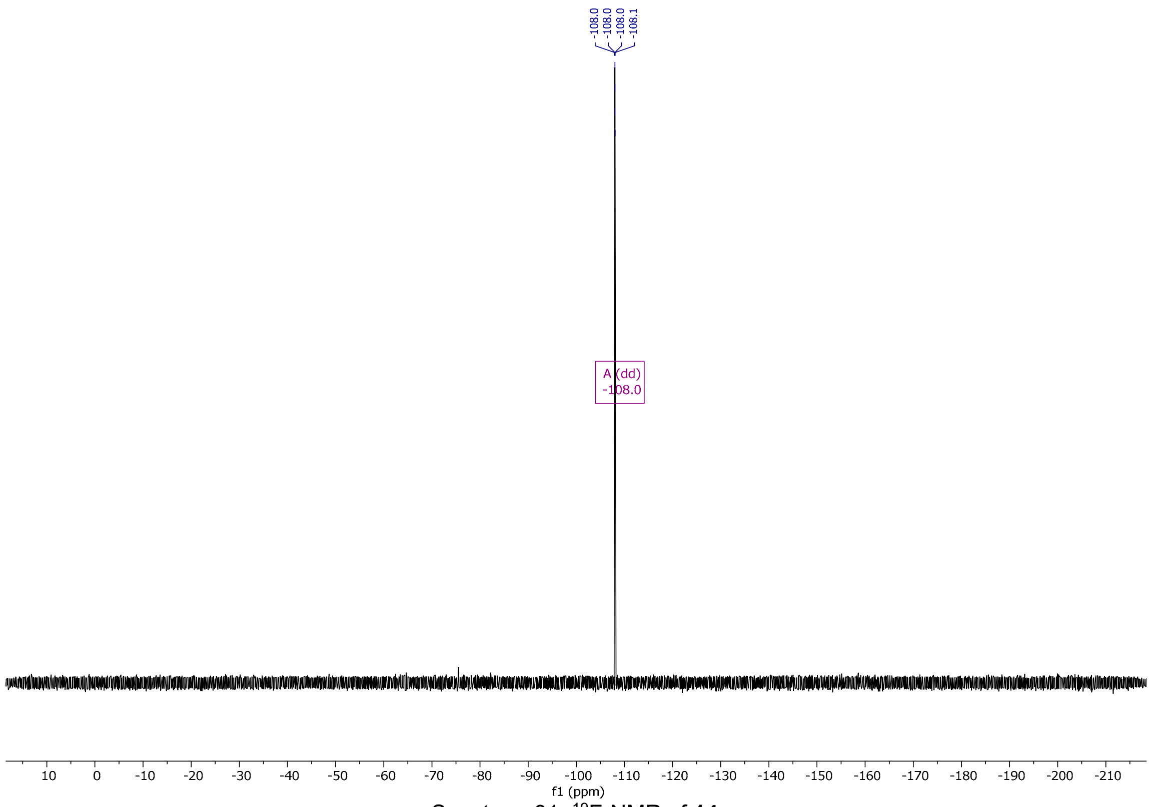

Spectrum 81: ^{19}F-NMR of **14**

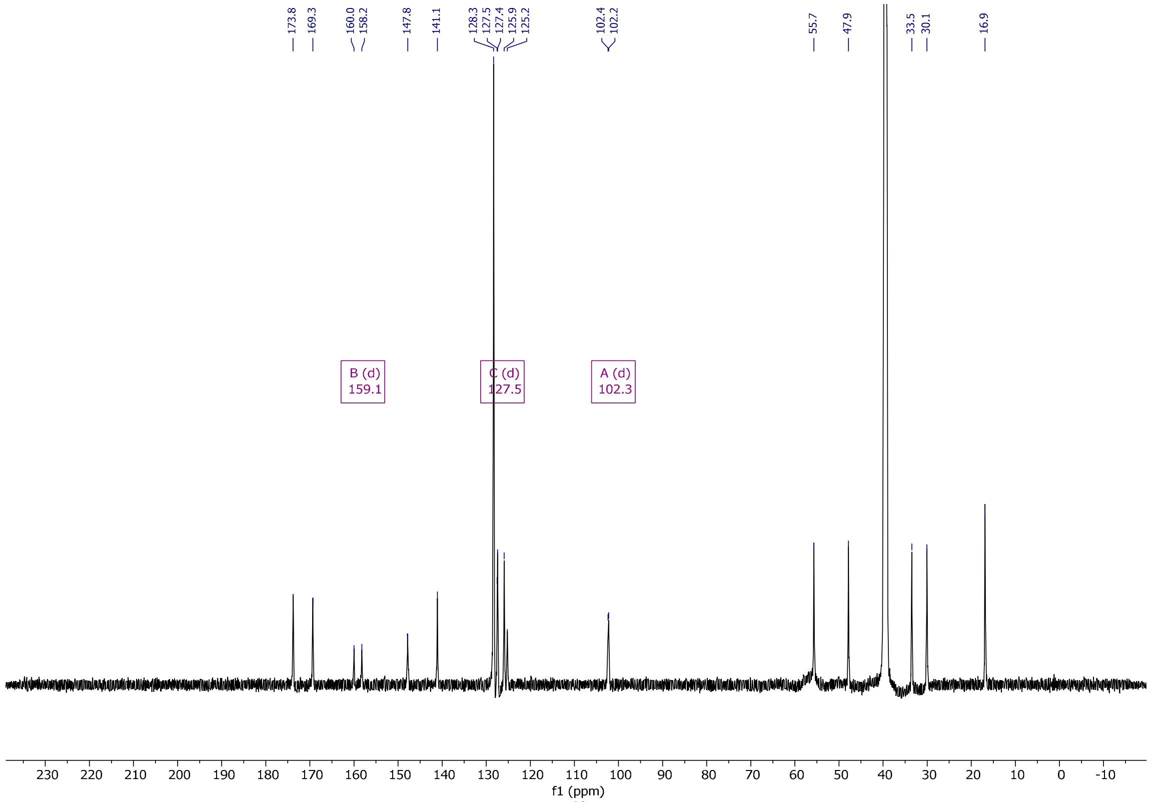

Spectrum 82: ^{13}C-NMR of **14**

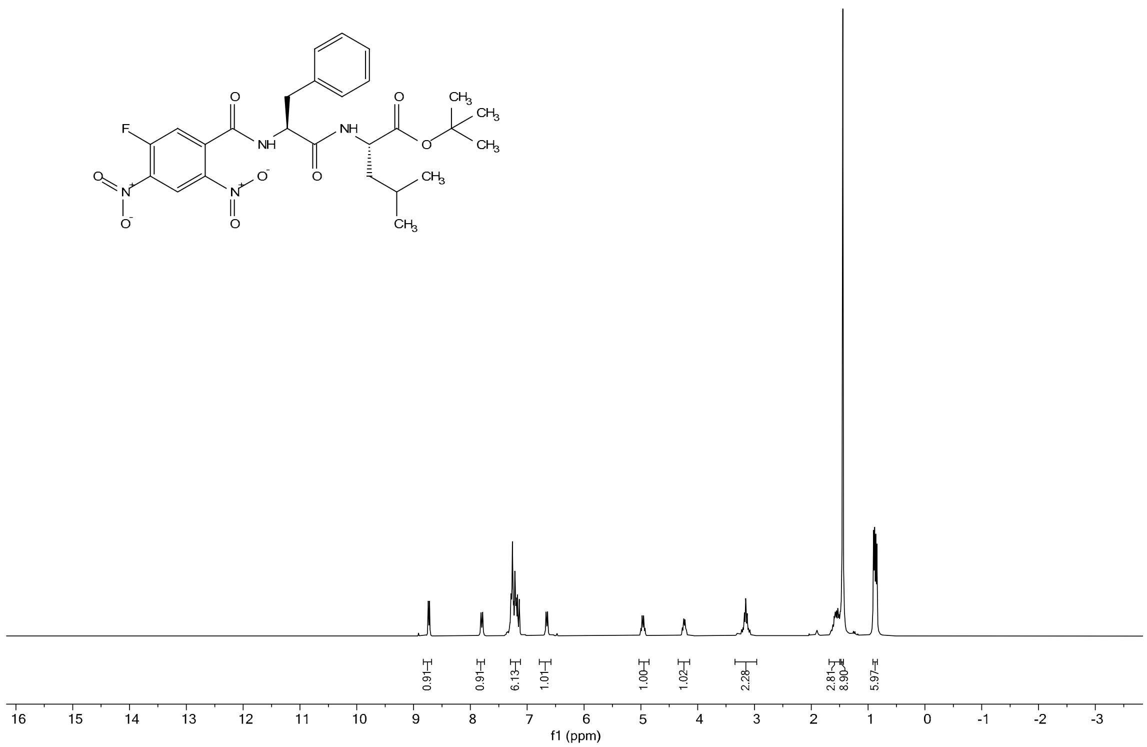

Spectrum 83: ^{1}H-NMR of **27**

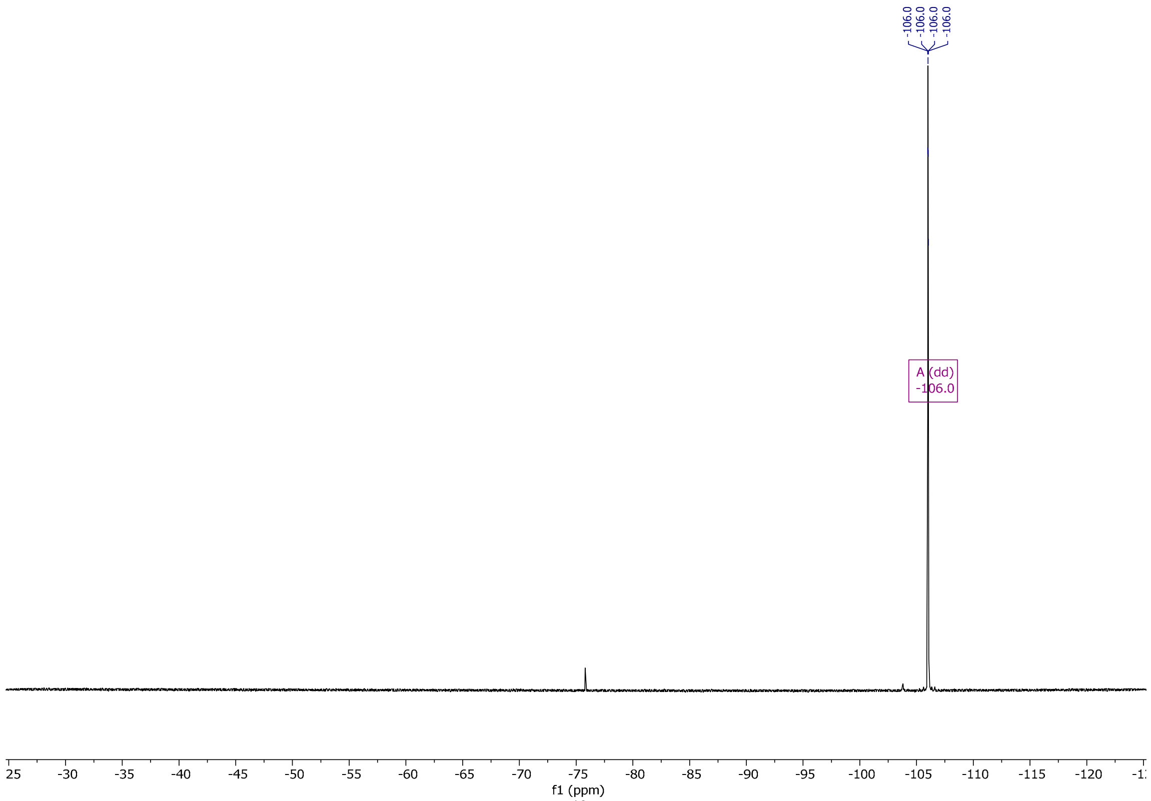

Spectrum 84: ^{19}F-NMR of **27**

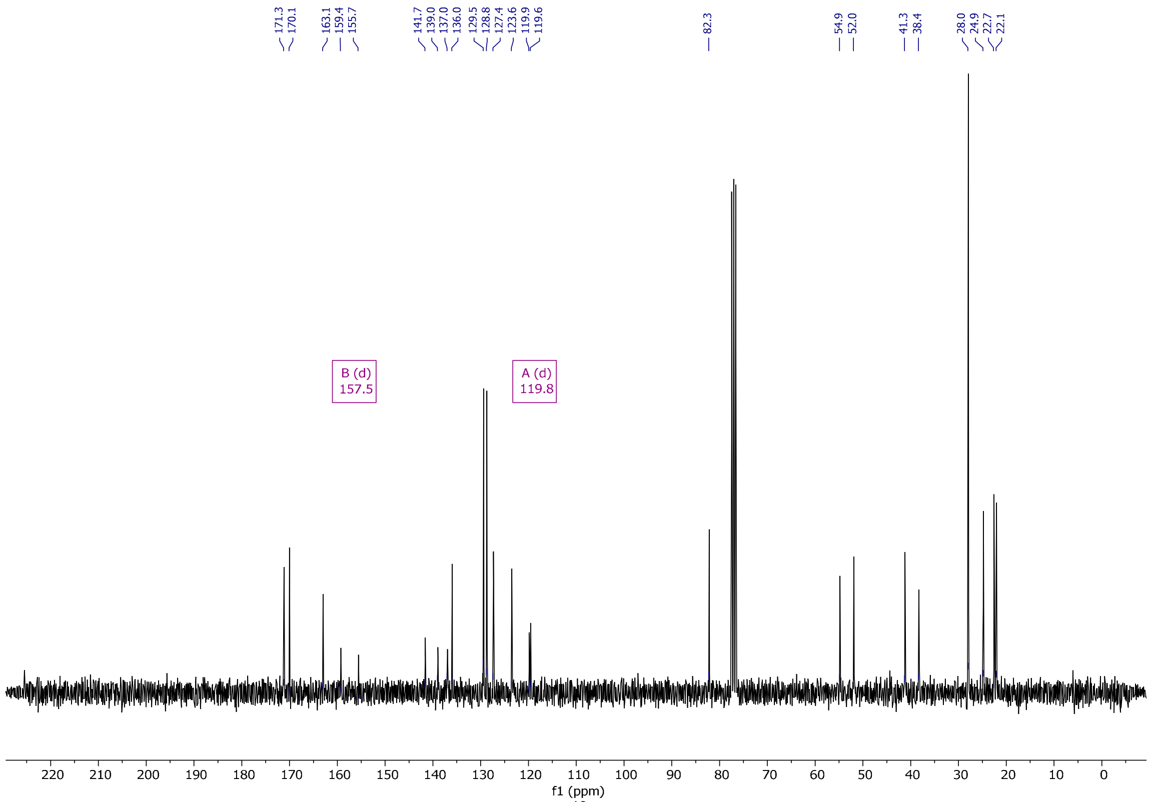

Spectrum 85: ^{13}C-NMR of **27**

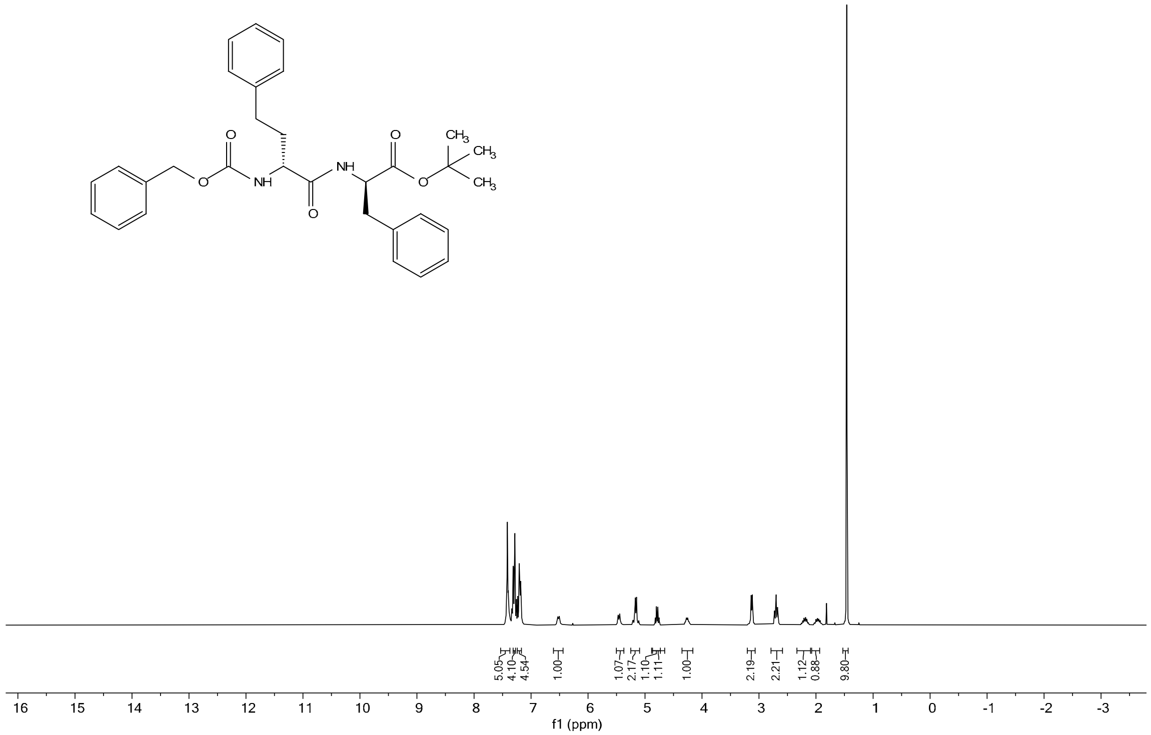

Spectrum 86: ^{1}H-NMR of **43**

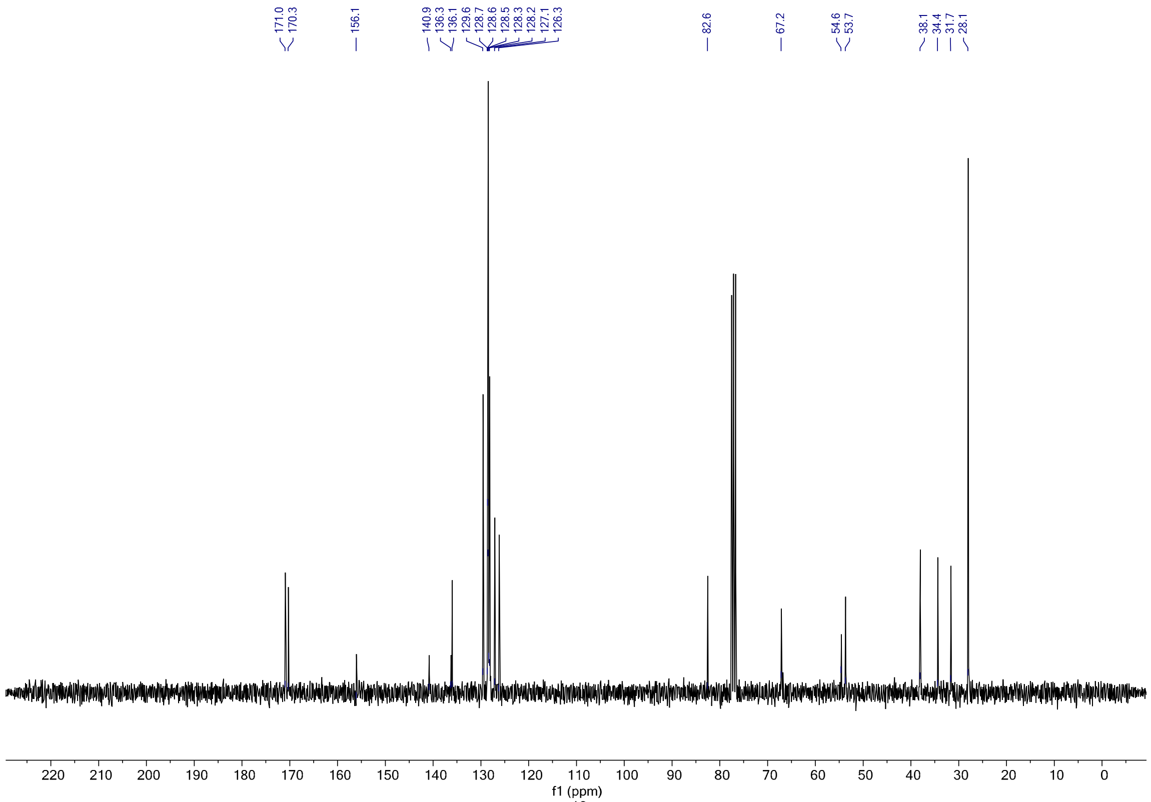

Spectrum 87: ^{13}C-NMR of **43**

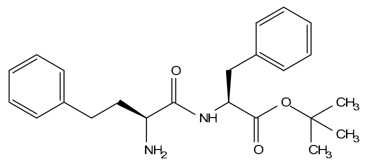

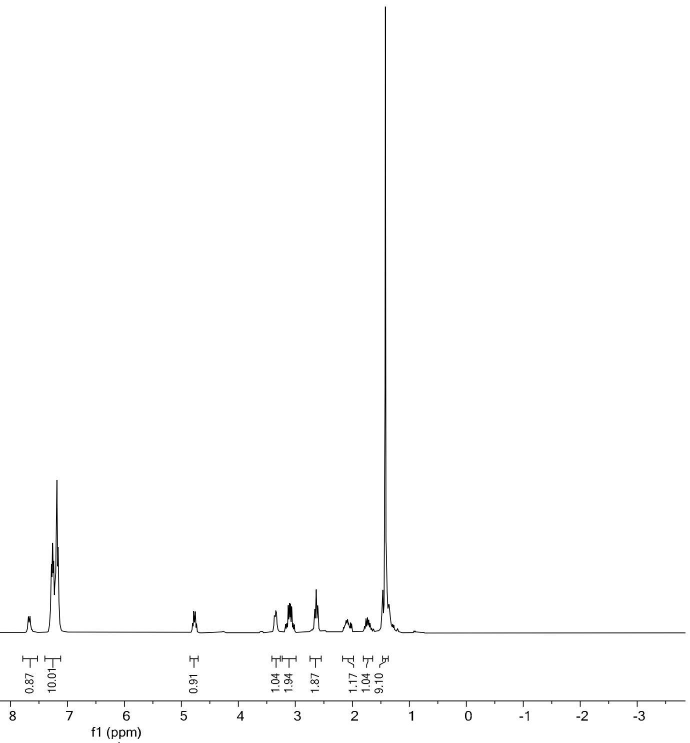

Spectrum 88: ^{1}H-NMR of **40**

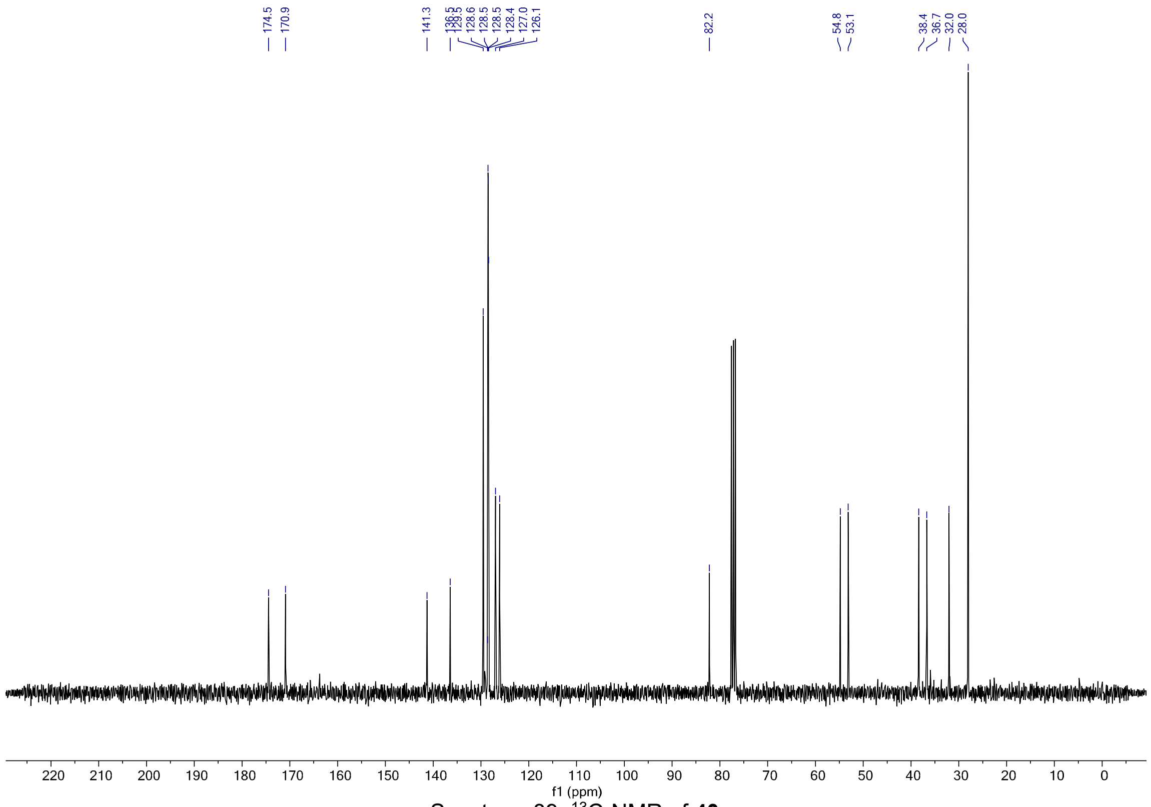

Spectrum 89: ^{13}C-NMR of **40**

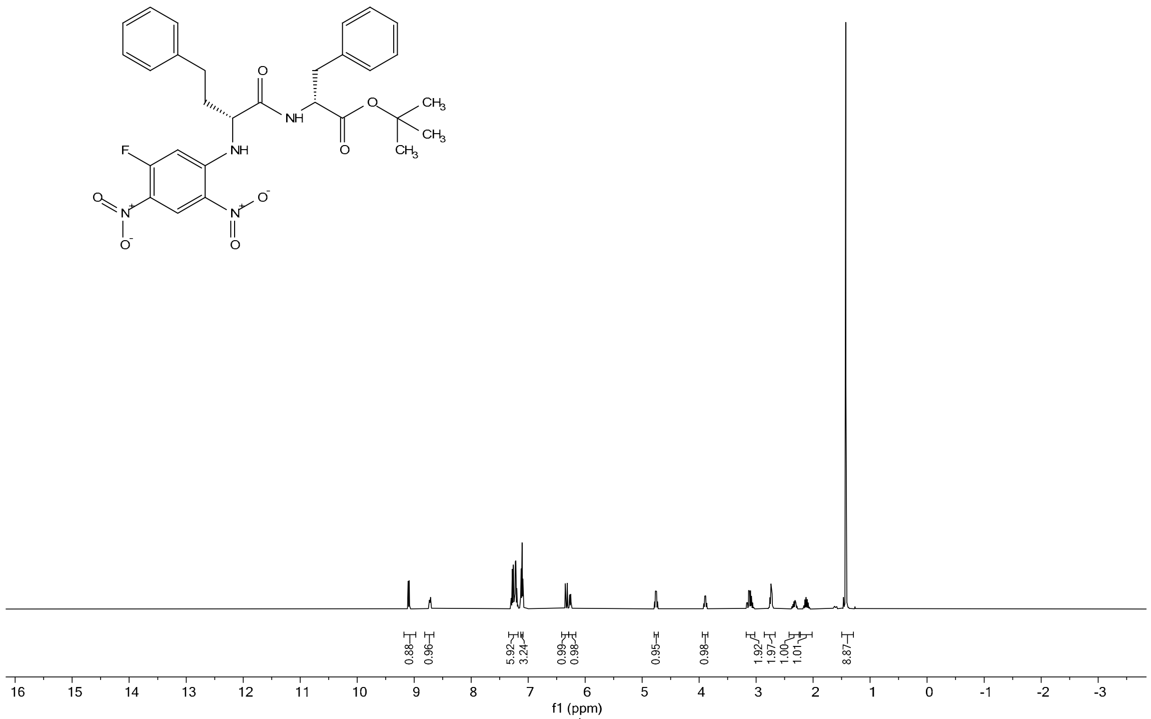

Spectrum 90: ^{1}H-NMR of **43**

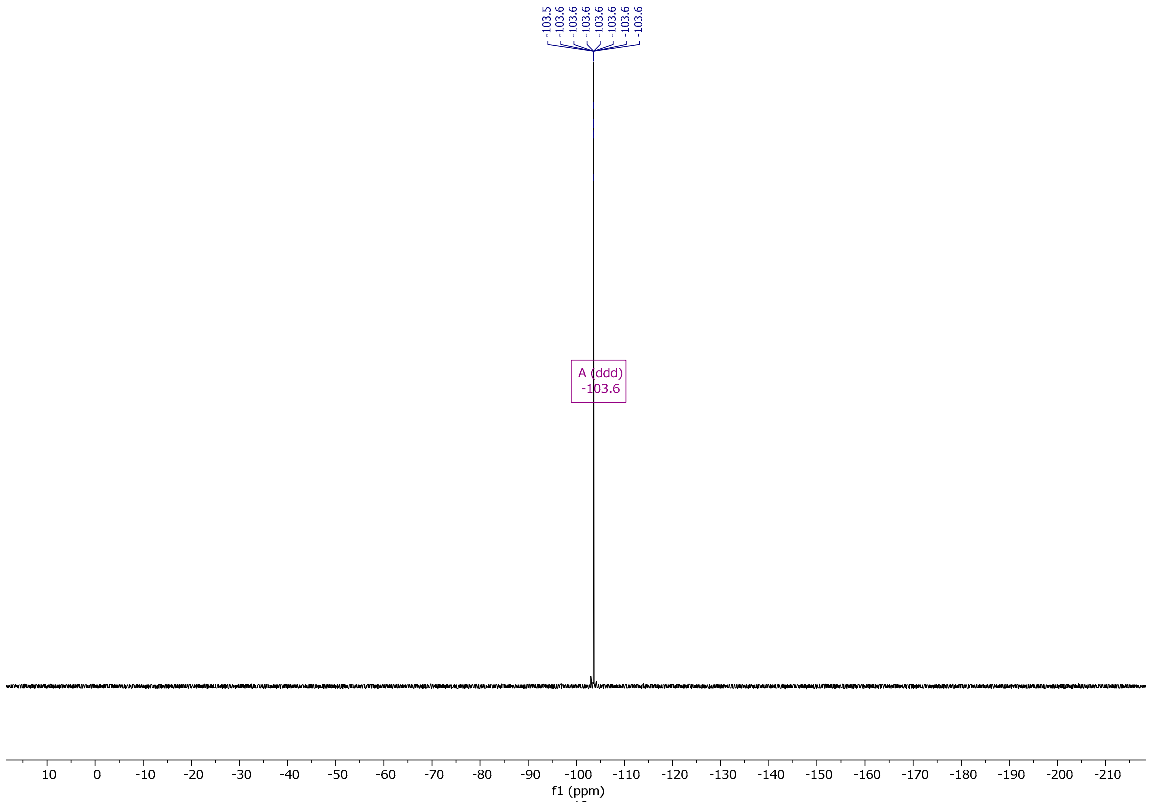

Spectrum 91: ^{19}F-NMR of **43**

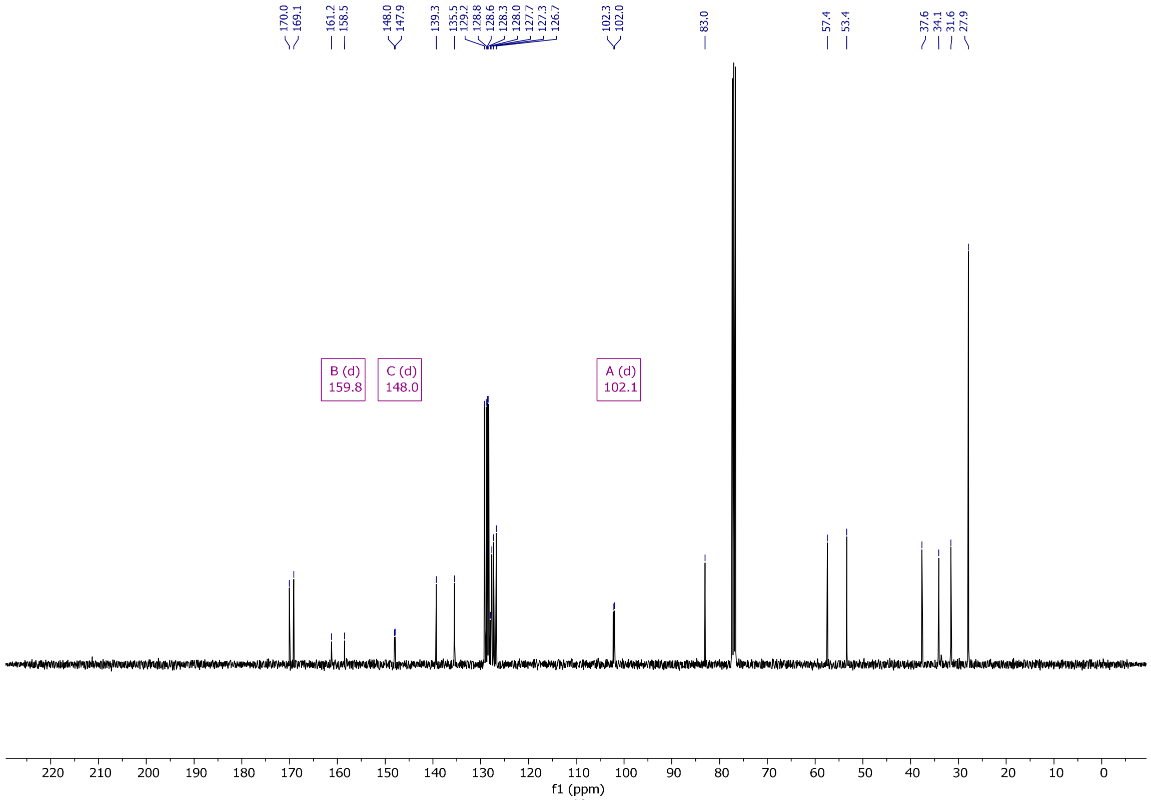

Spectrum 92: ^{13}C-NMR of **43**

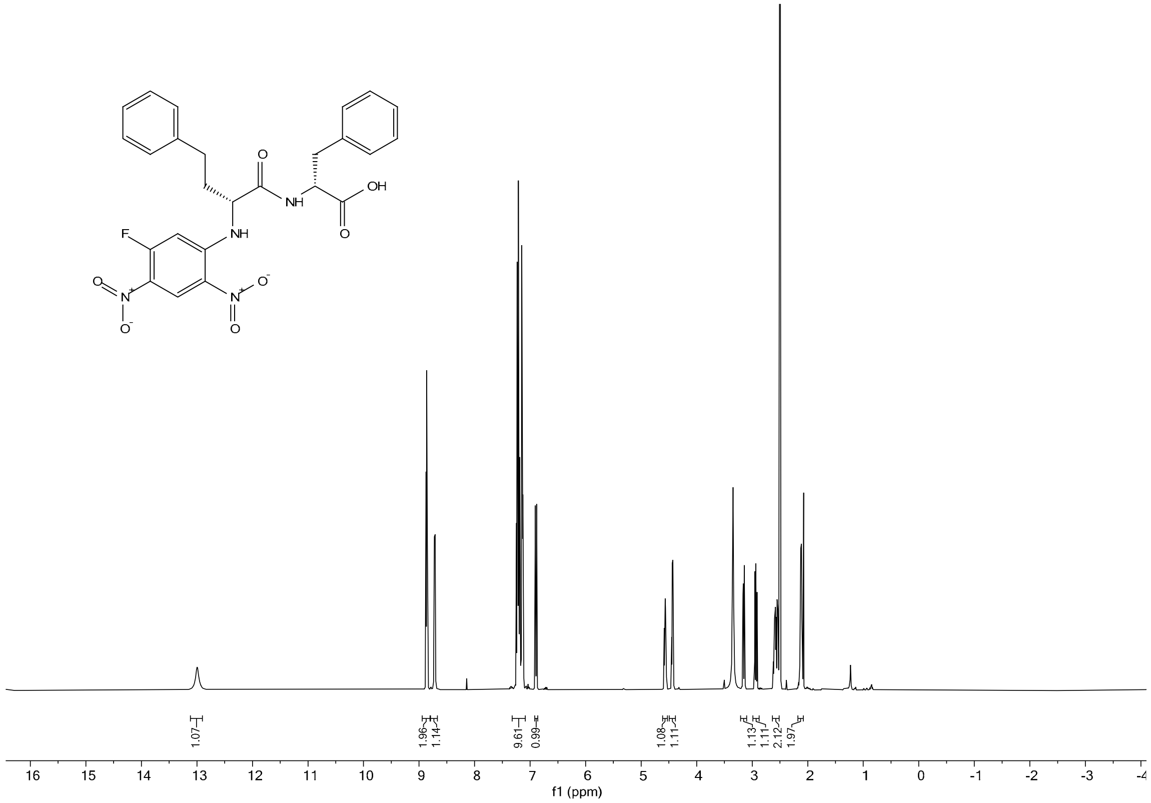

Spectrum 93: ^{1}H-NMR of **18**

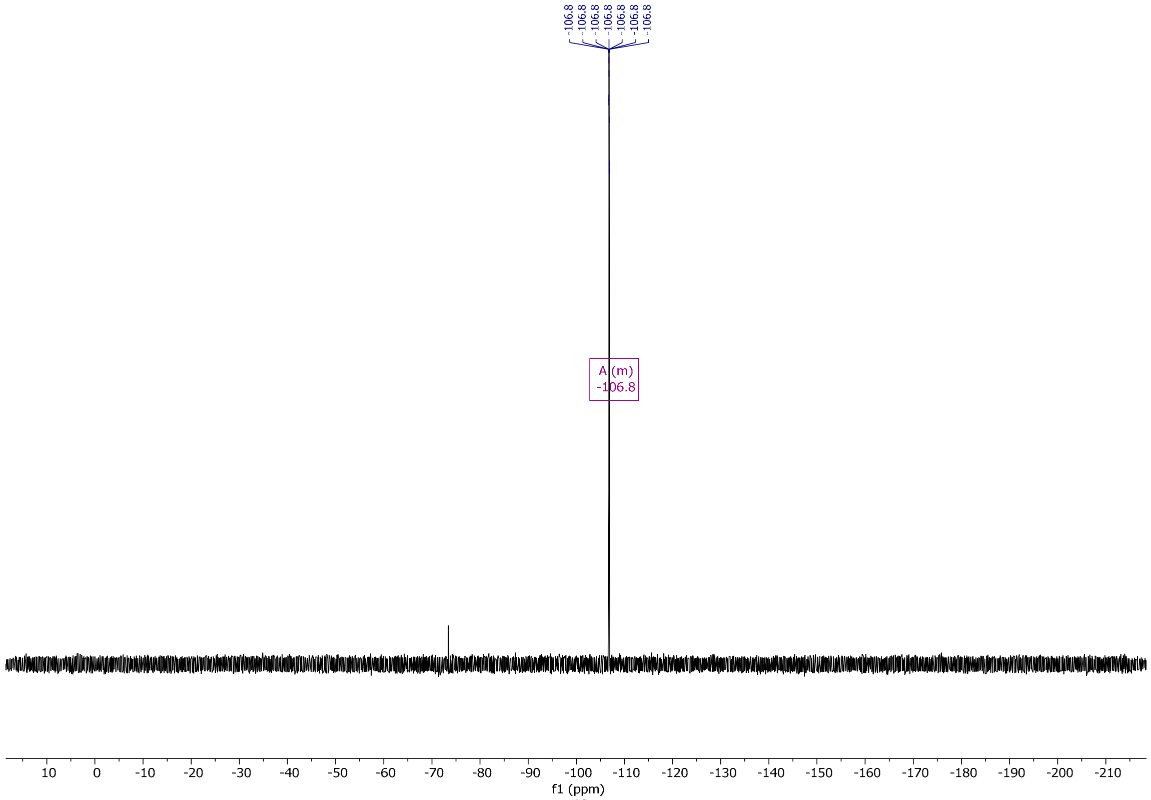

Spectrum 94: ^{19}F-NMR of **18**

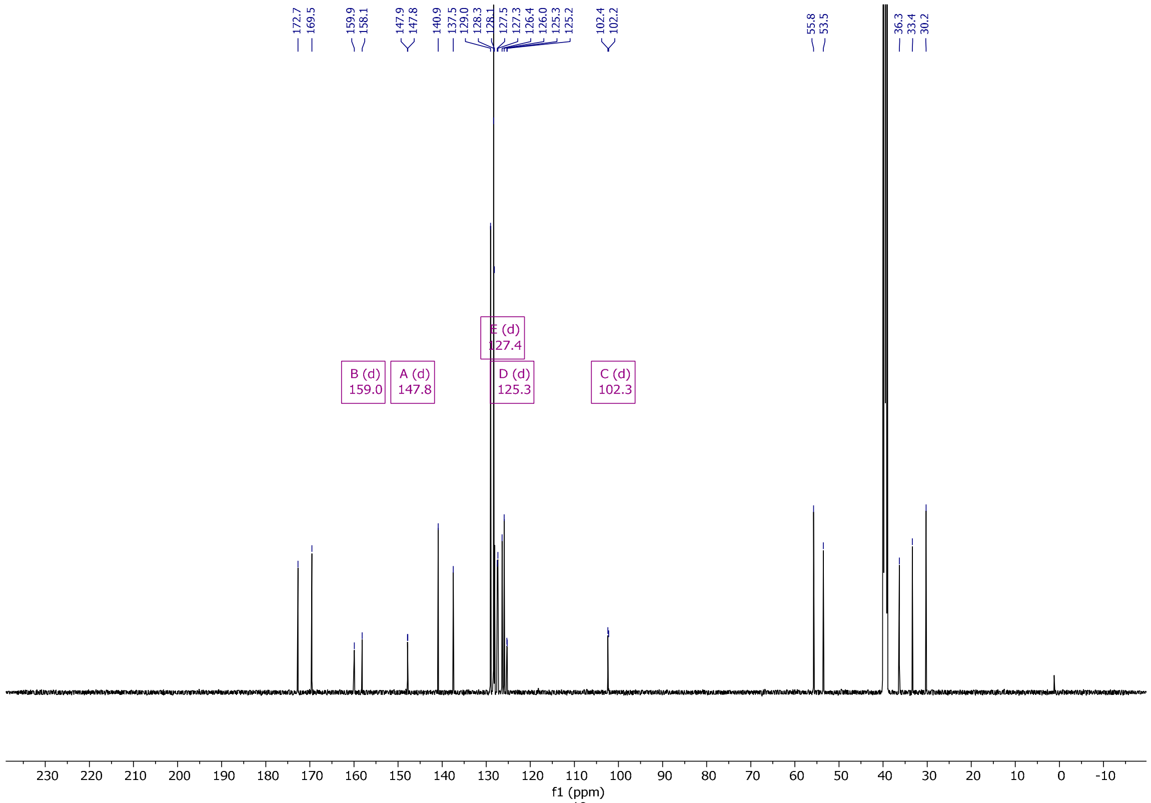

Spectrum 95: ^{13}C-NMR of **18**

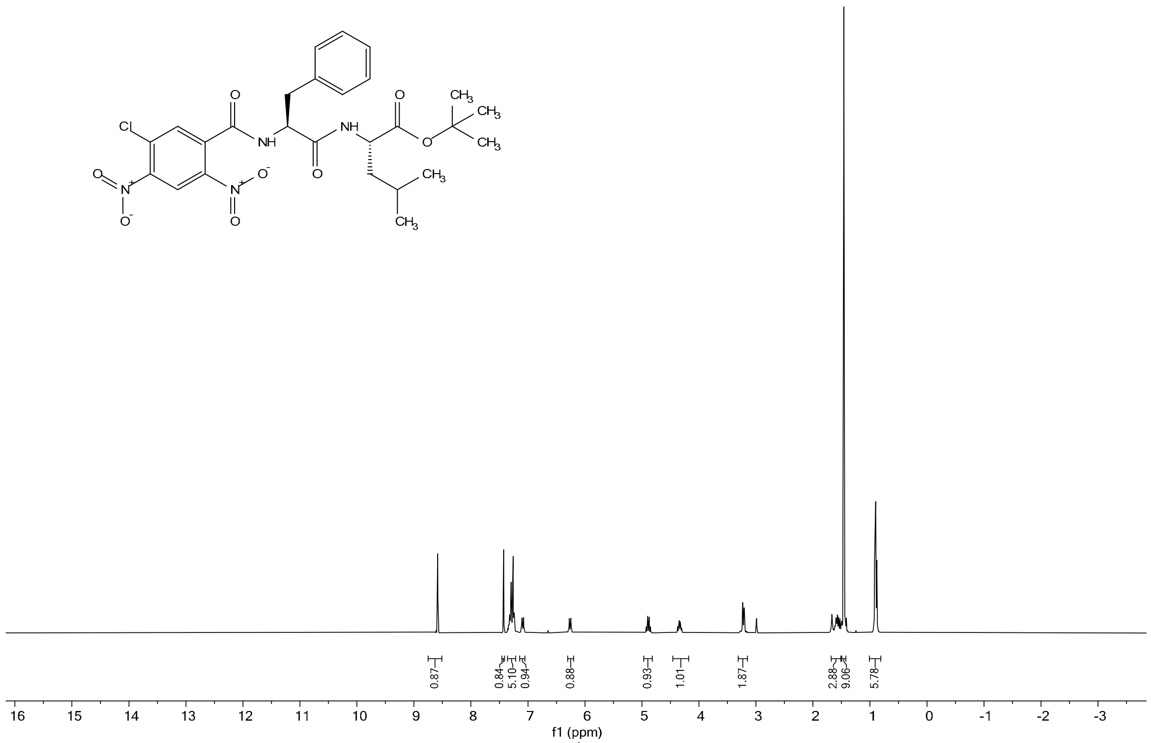

Spectrum 96: ^{1}H-NMR of **30**

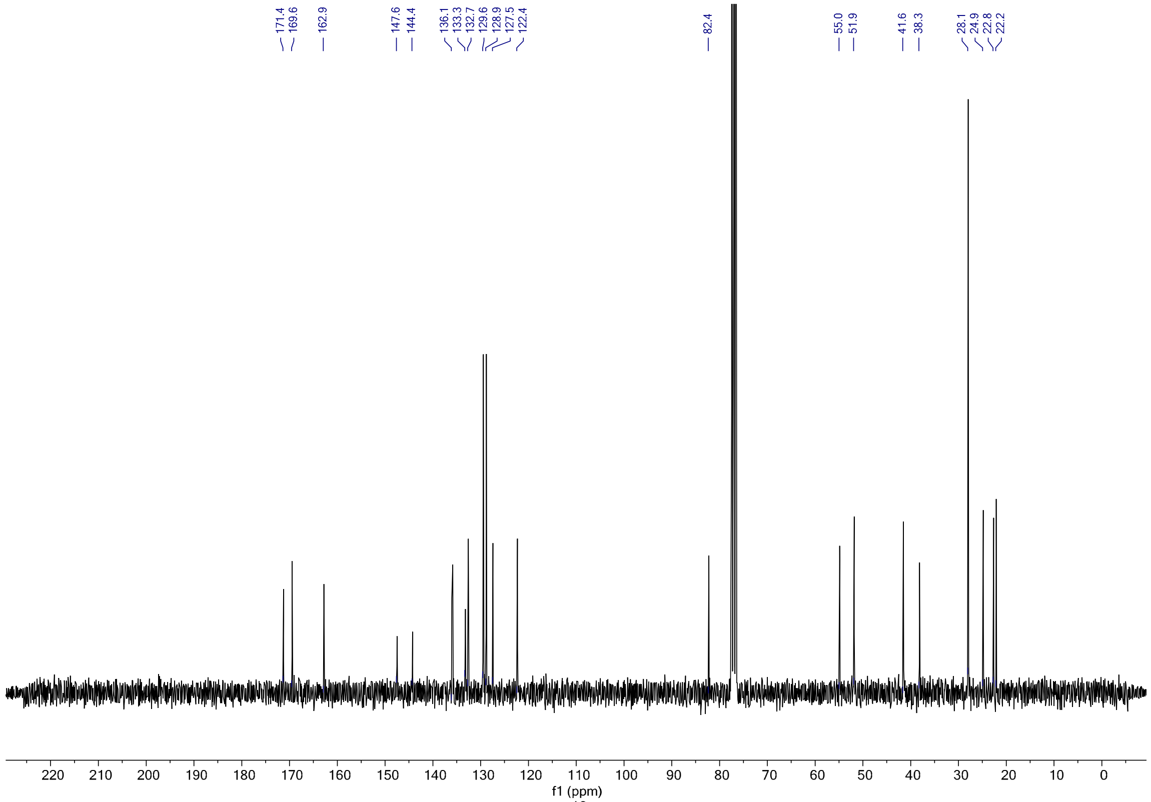

Spectrum 97: ^{13}C-NMR of **30**

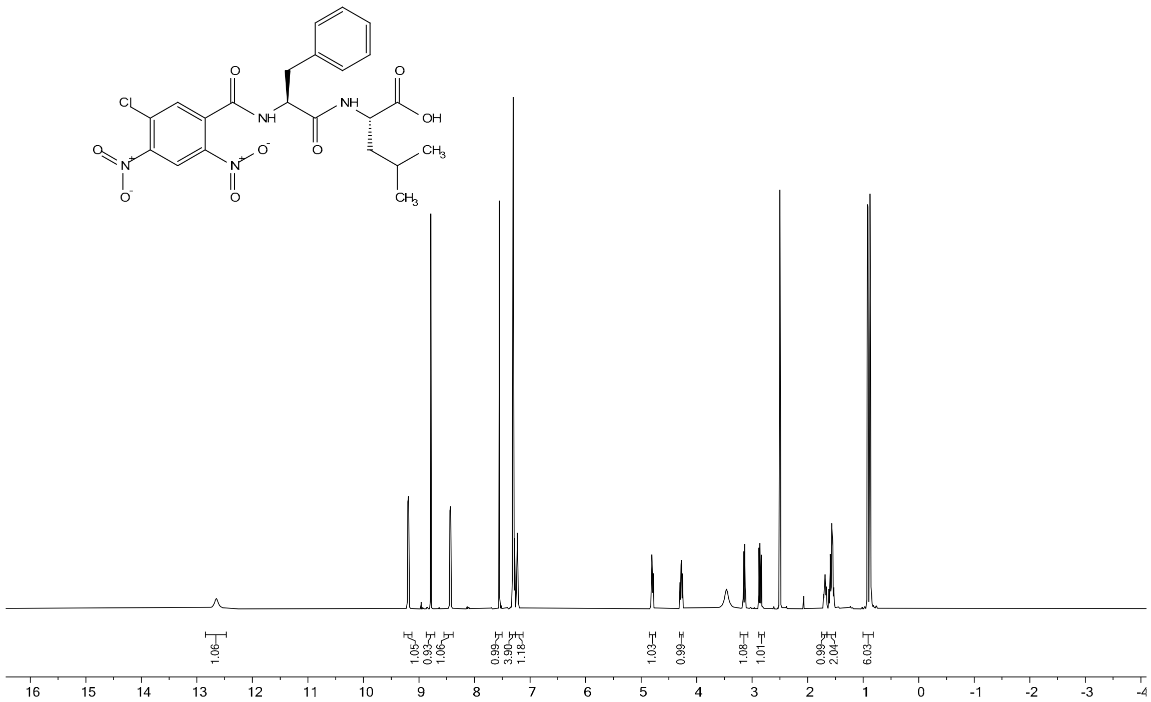

Spectrum 98: ^{1}H-NMR of **12**

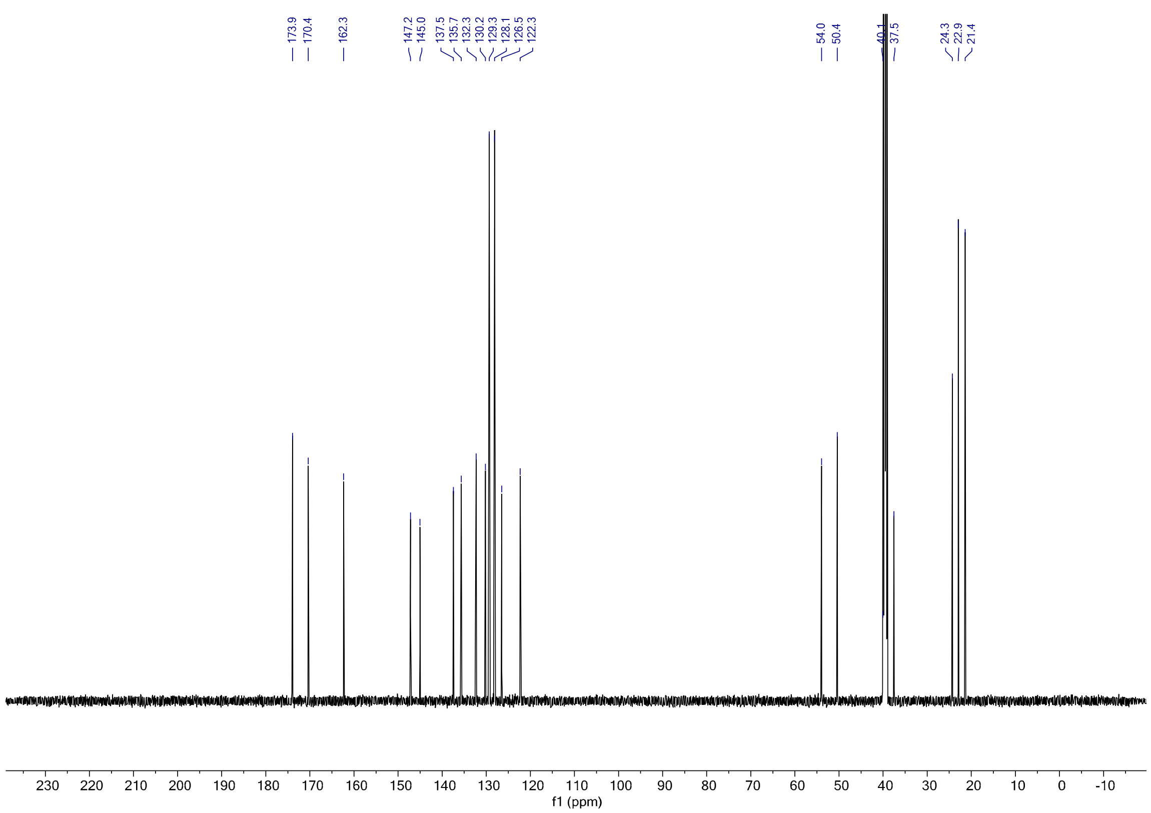

Spectrum 99: ^{13}C-NMR of **12**

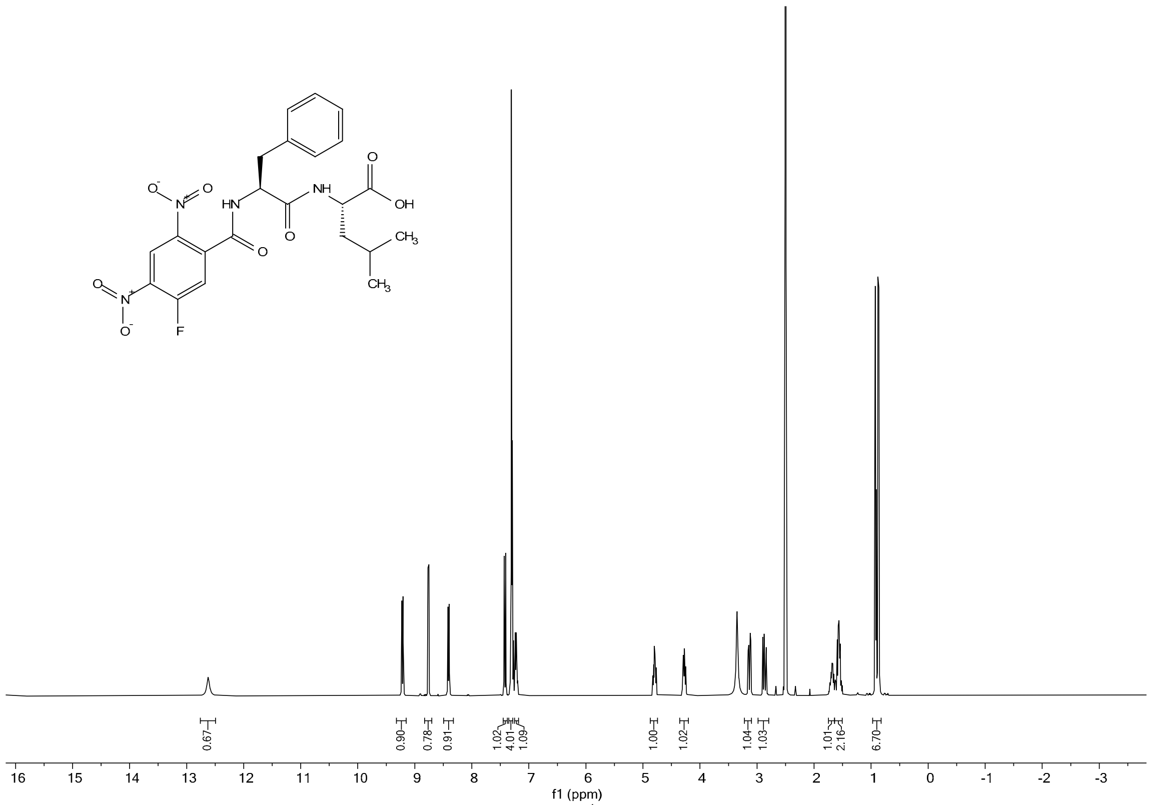

Spectrum 100: ^{1}H-NMR of **9**

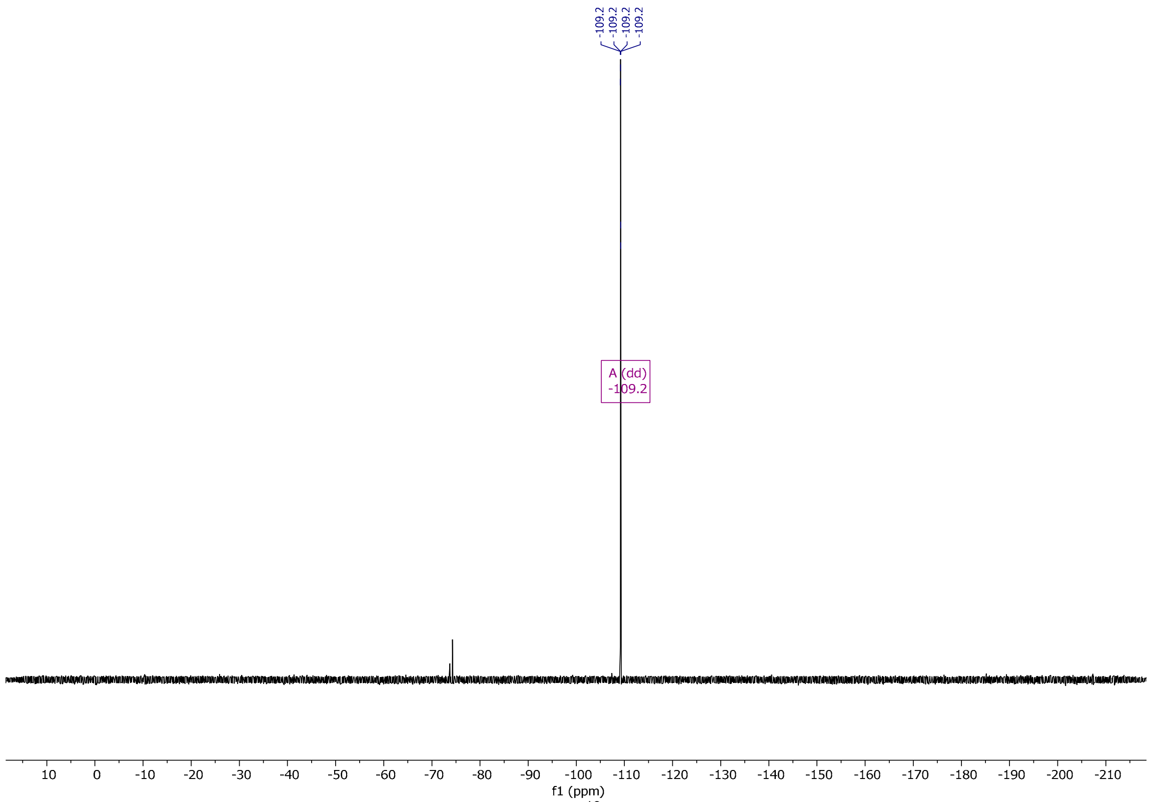

Spectrum 101: ^{19}F-NMR of **9**

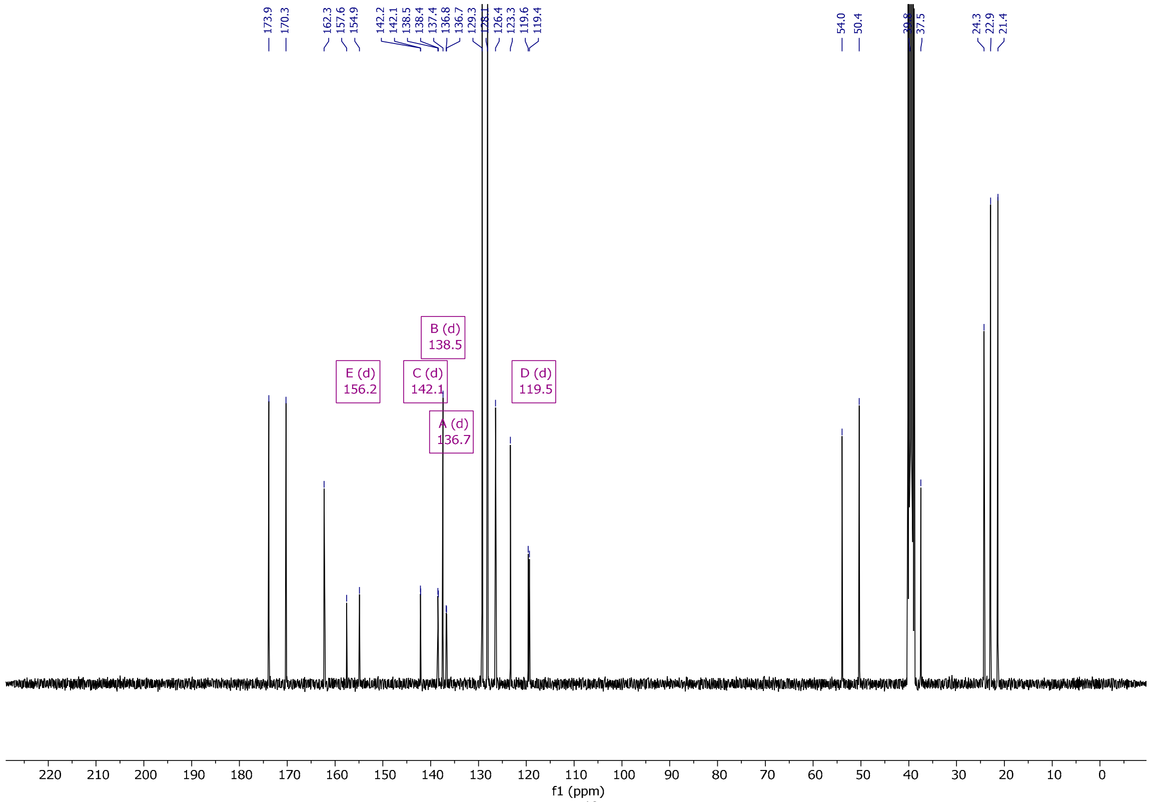

Spectrum 102: ^{13}C-NMR of **9**

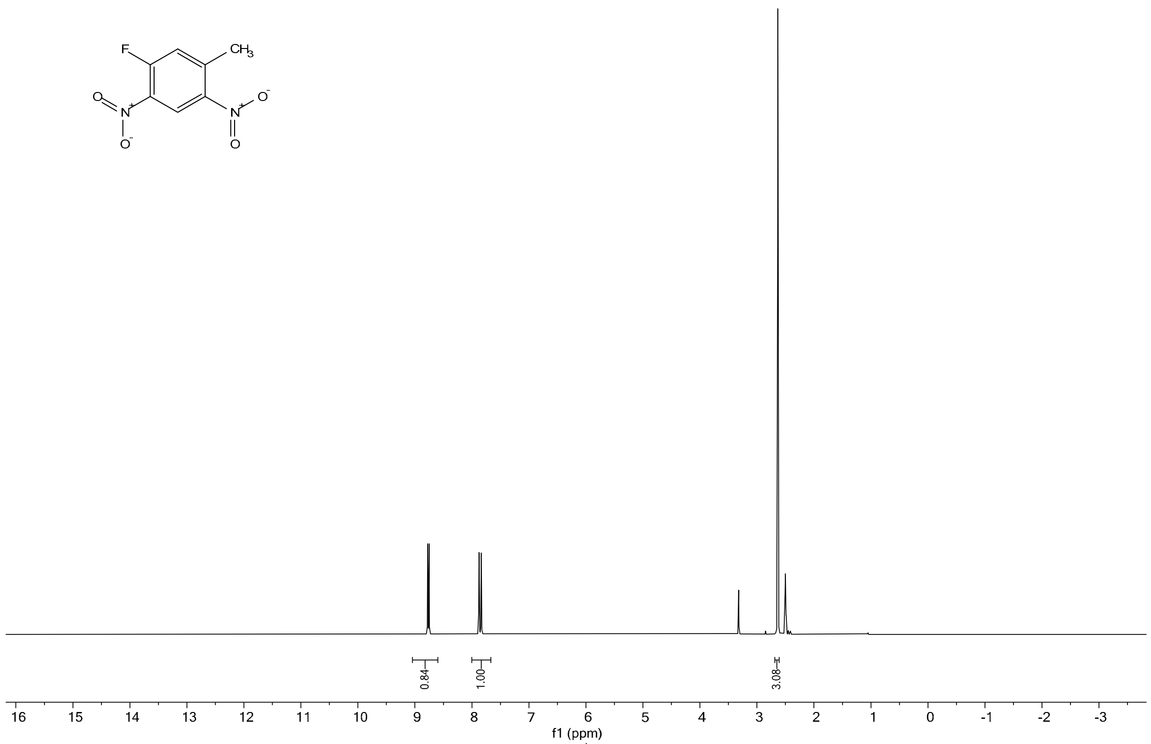

Spectrum 103: ^{1}H-NMR of **34**

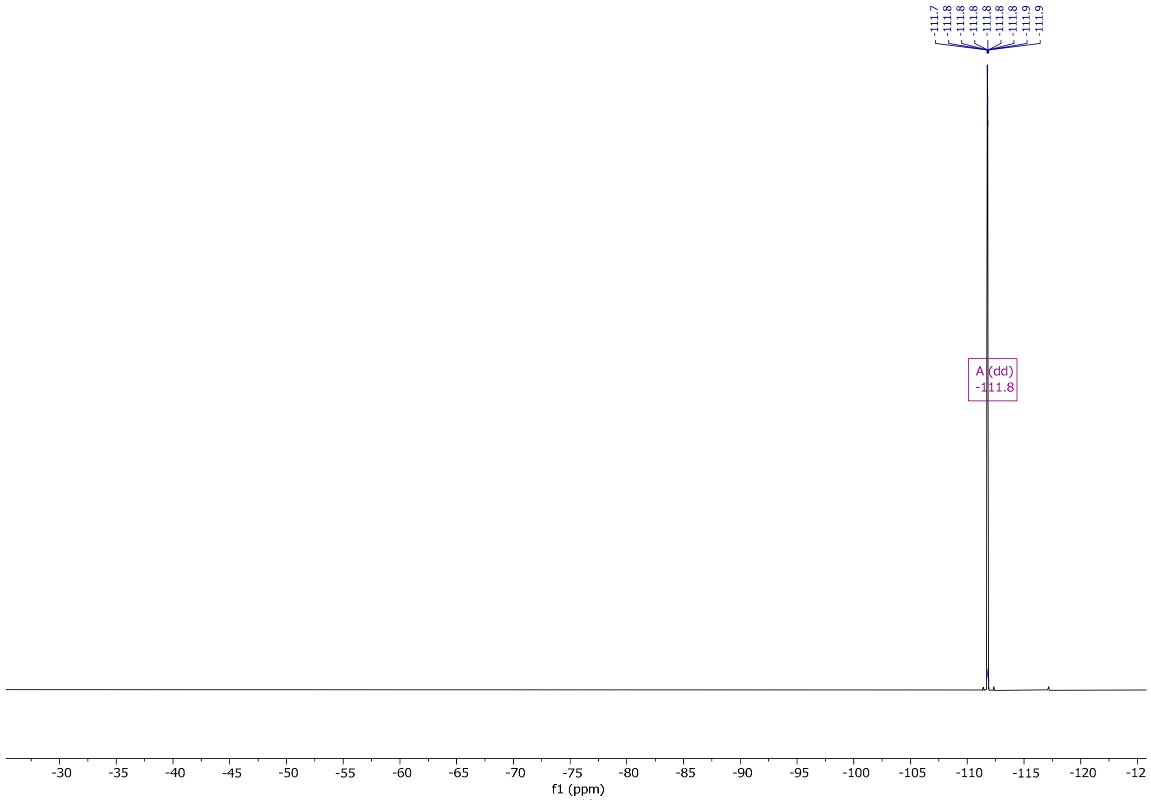

Spectrum 104: ^{19}F-NMR of **34**

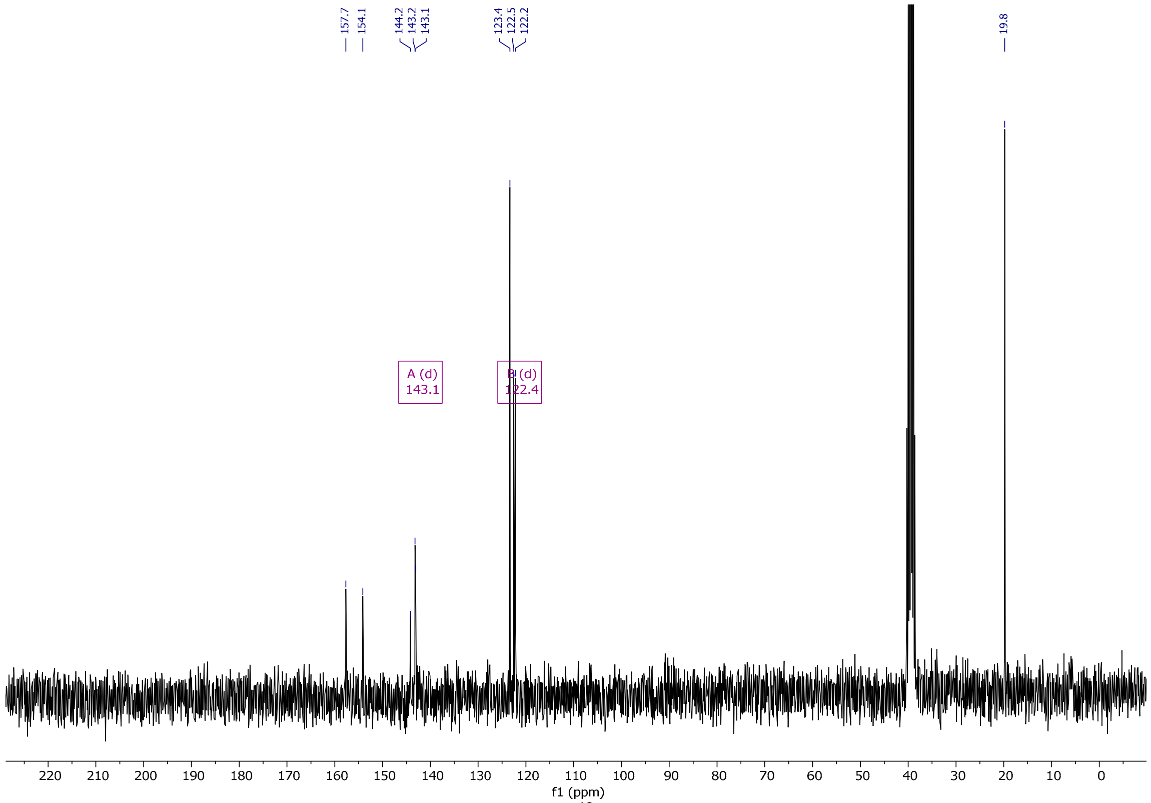

Spectrum 105: ^{13}C-NMR of **34**

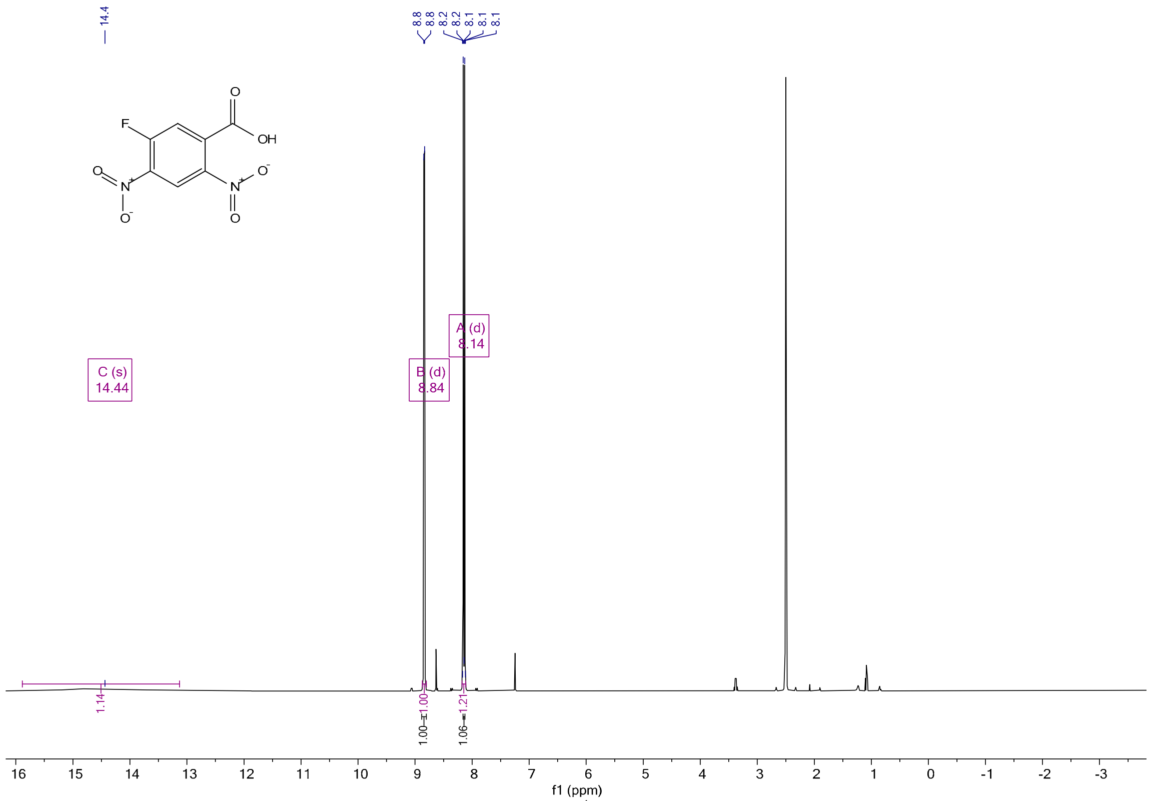

Spectrum 106: ^{1}H-NMR of **33**

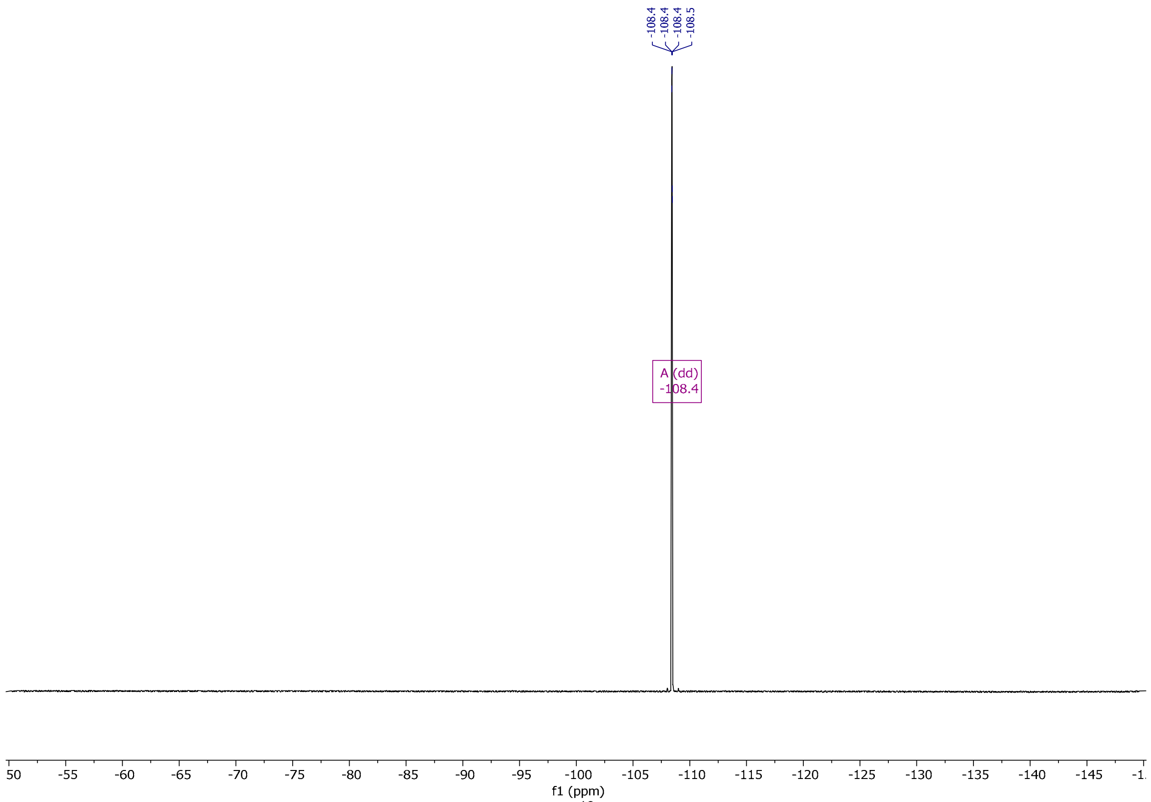

Spectrum 107: ^{19}F-NMR of **33**

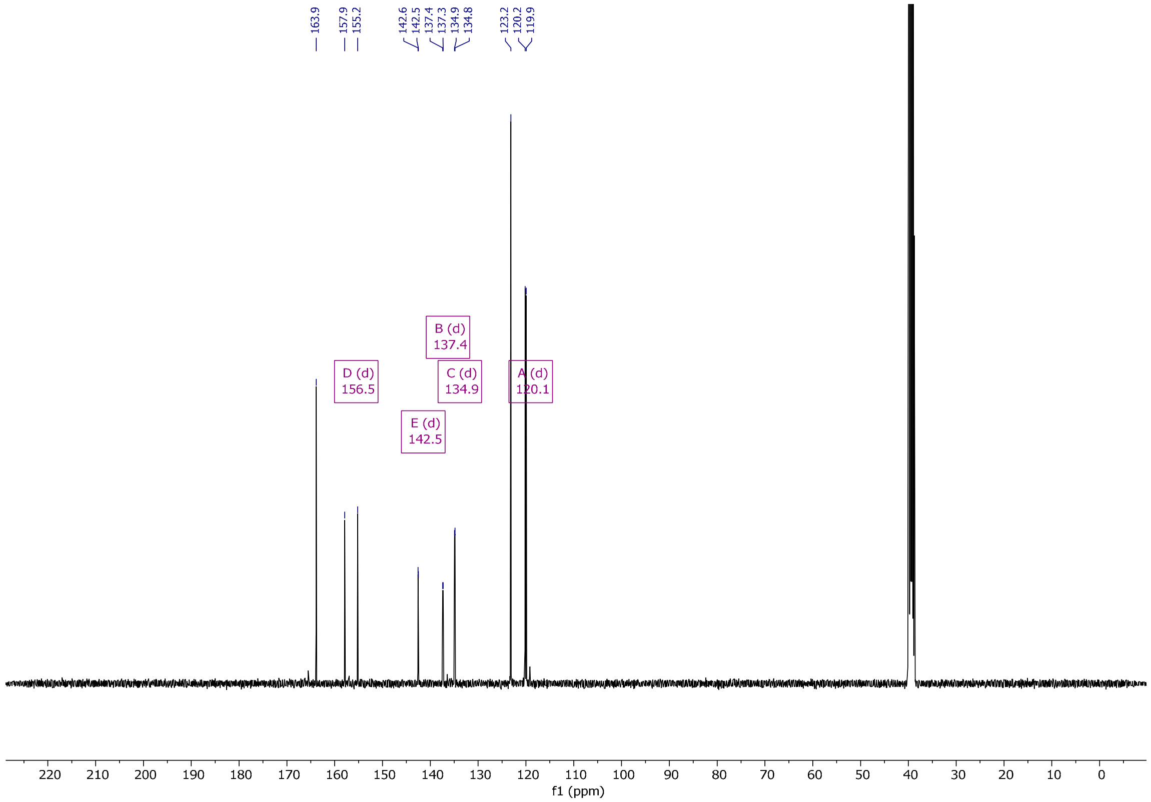

Spectrum 108: ^{13}C-NMR of **33**

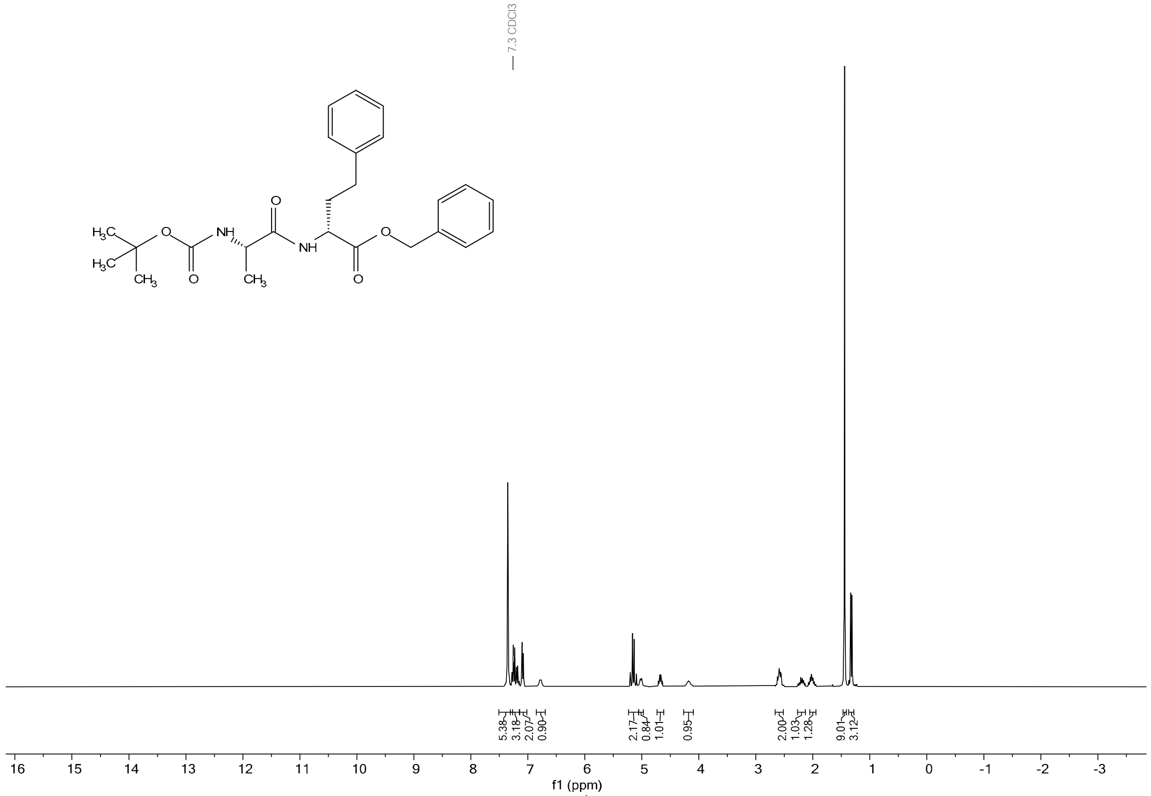

Spectrum 109: ^{1}H-NMR of **44**

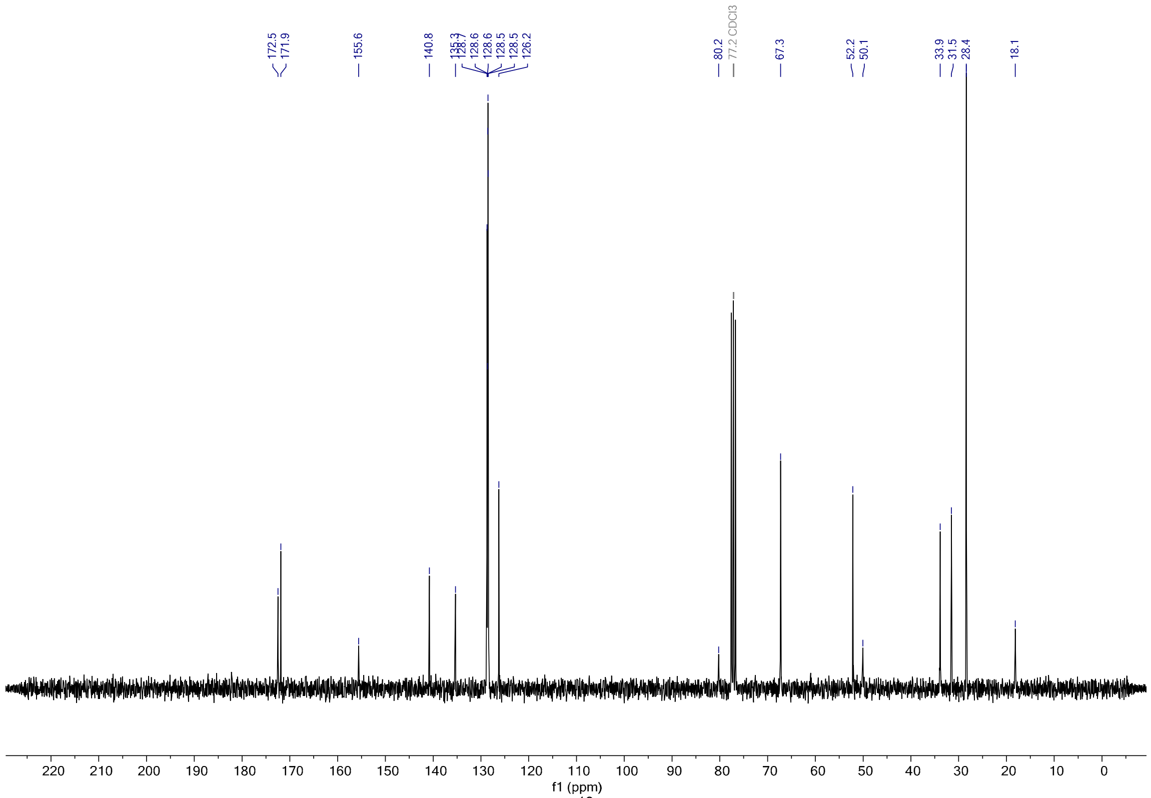

Spectrum 110: ^{13}C-NMR of **44**

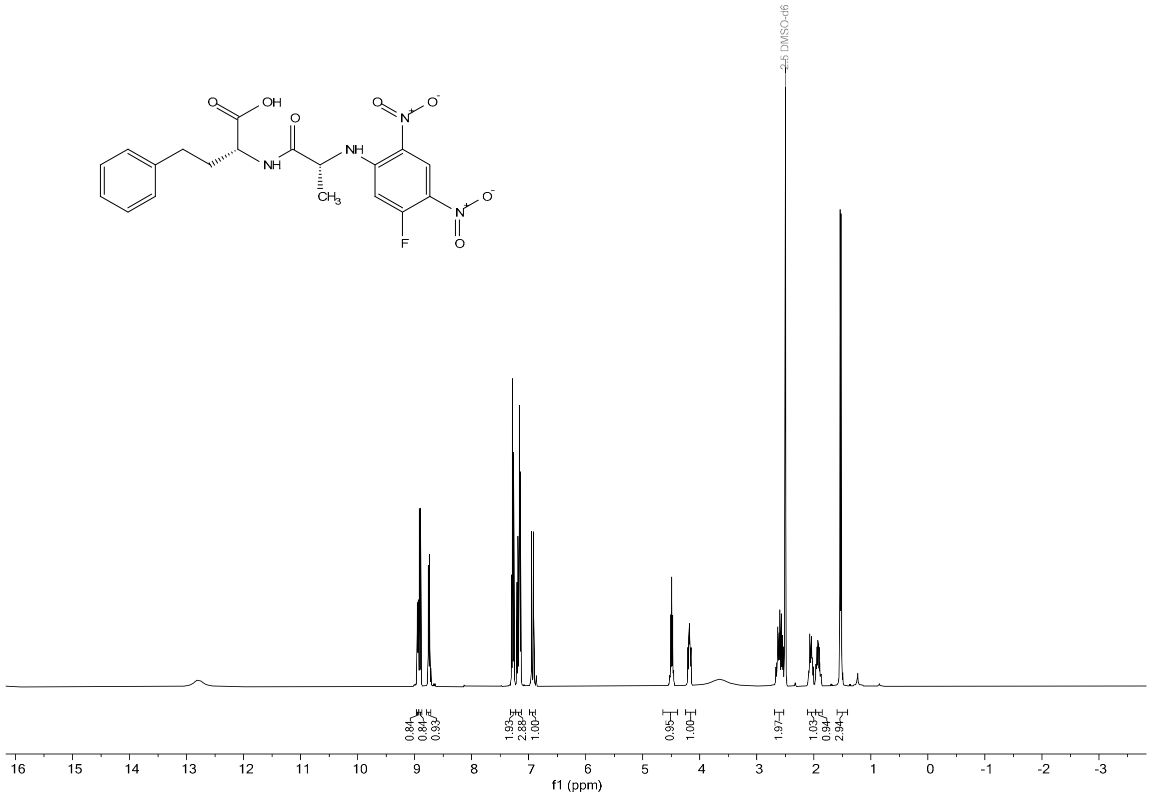

Spectrum 111: ^{1}H-NMR of **15**

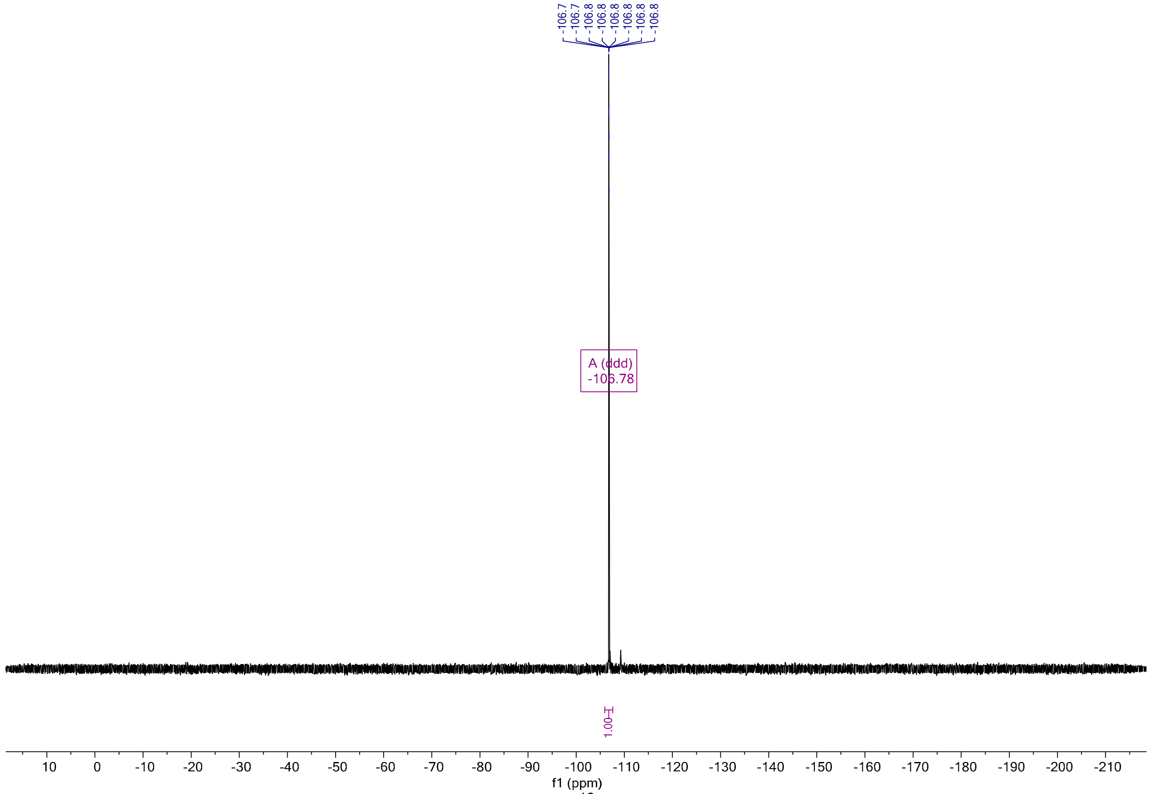

Spectrum 112: ^{19}F-NMR of **15**

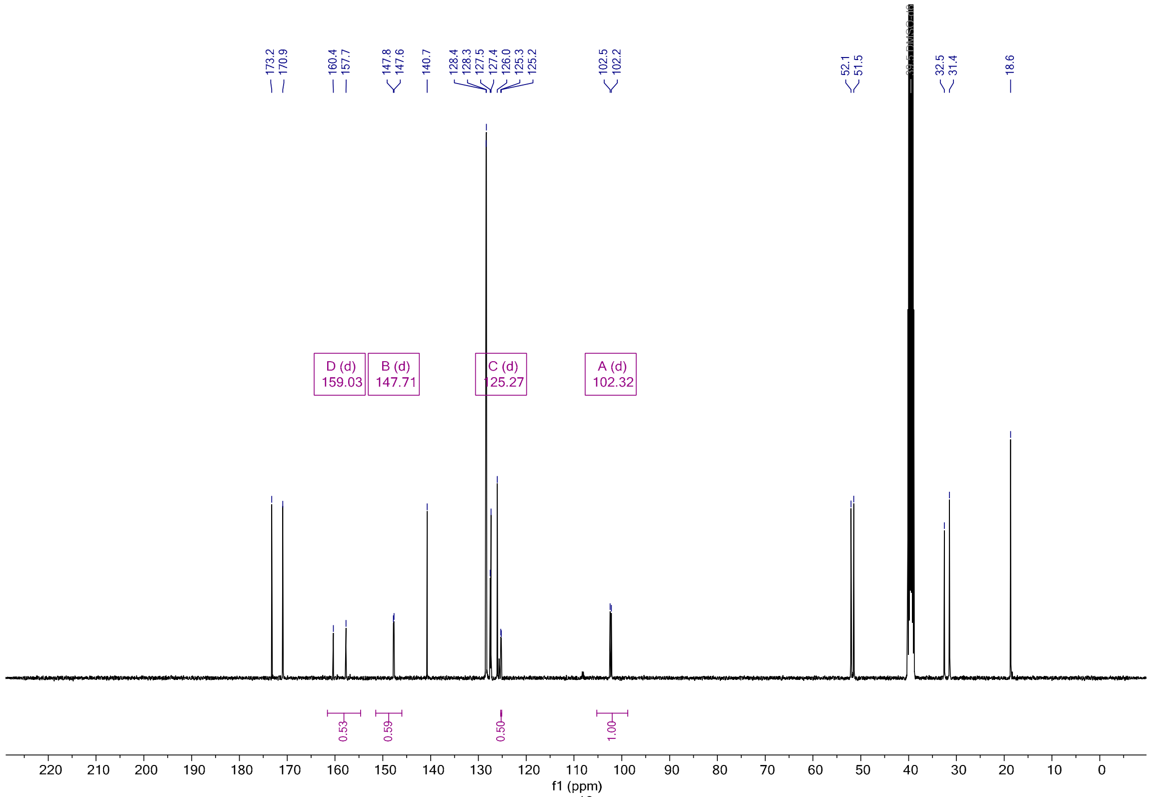

Spectrum 113: ^{13}C-NMR of **15**

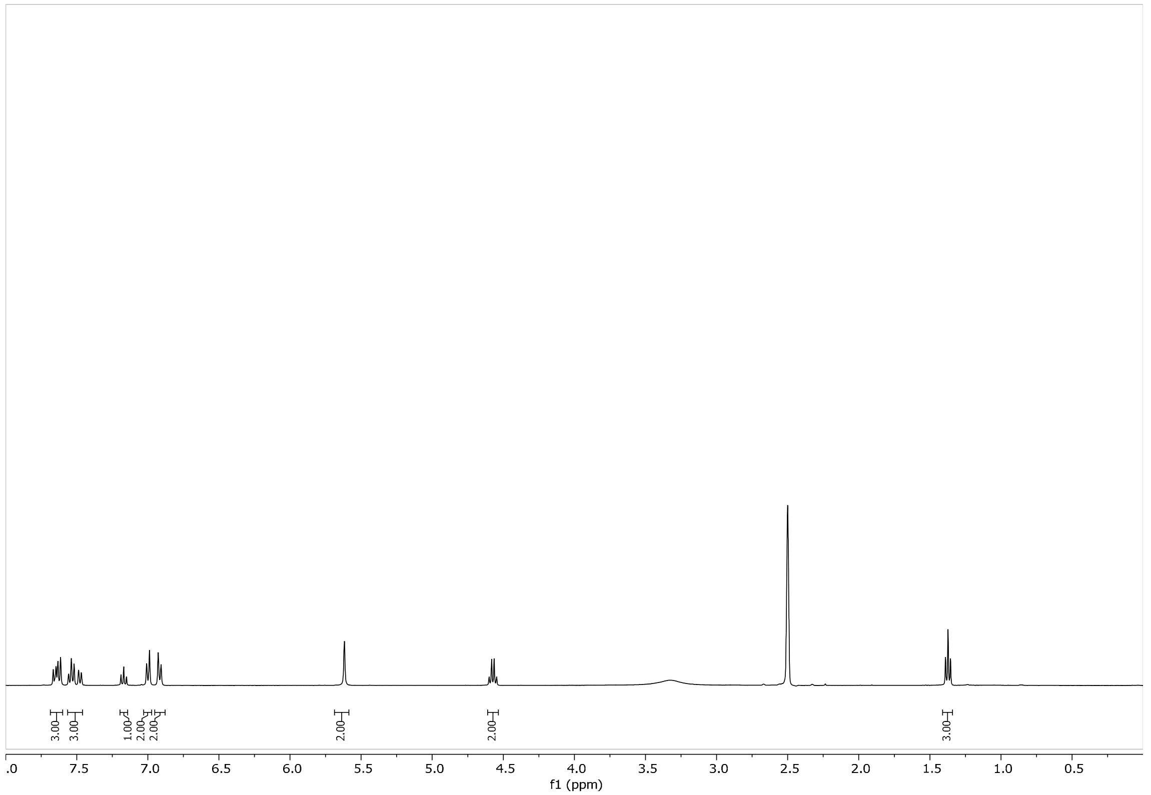

Spectrum 114: ^{1}H-NMR of candesartan

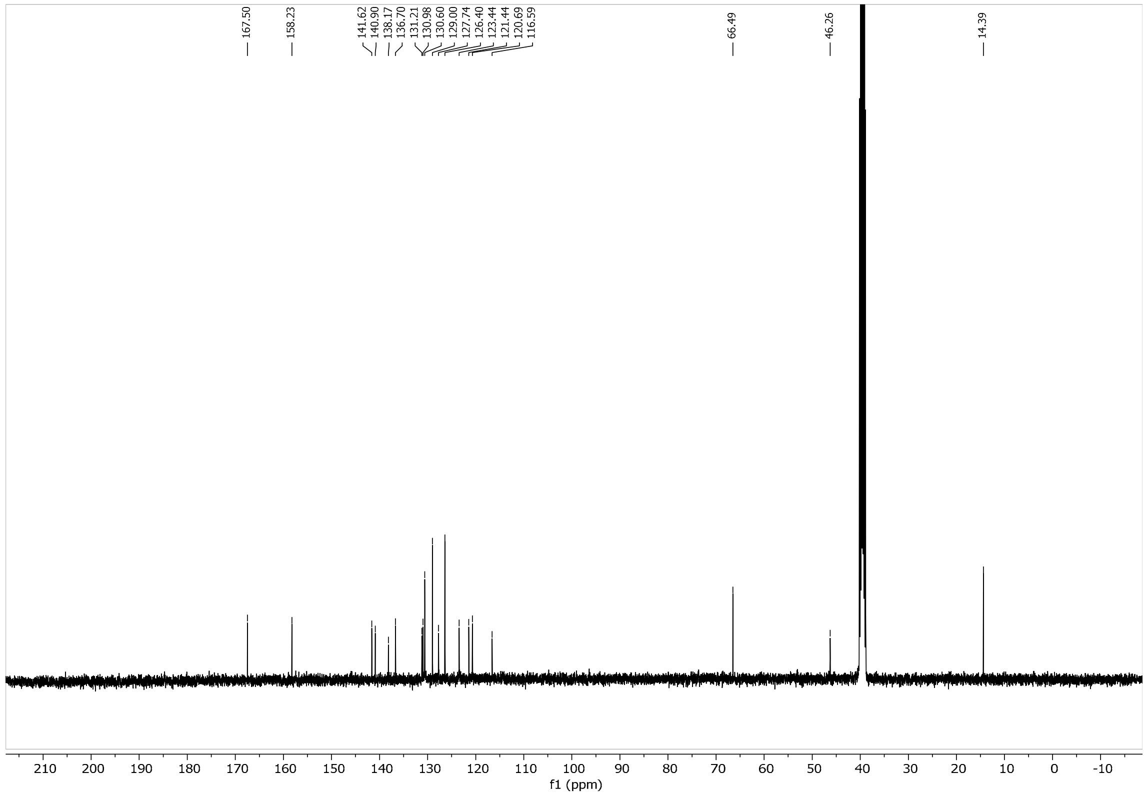

Spectrum 115: ^{13}C-NMR of candesartan
